# The Past, Present, and Future of Virtual and Augmented Reality Research: A Network and Cluster Analysis of the Literature

**DOI:** 10.3389/fpsyg.2018.02086

**Published:** 2018-11-06

**Authors:** Pietro Cipresso, Irene Alice Chicchi Giglioli, Mariano Alcañiz Raya, Giuseppe Riva

**Affiliations:** ^1^Applied Technology for Neuro-Psychology Lab, Istituto Auxologico Italiano, Milan, Italy; ^2^Department of Psychology, Catholic University of the Sacred Heart, Milan, Italy; ^3^Instituto de Investigación e Innovación en Bioingeniería, Universitat Politècnica de València, Valencia, Spain

**Keywords:** virtual reality, augmented reality, quantitative psychology, measurement, psychometrics, scientometrics, computational psychometrics, mathematical psychology

## Abstract

The recent appearance of low cost virtual reality (VR) technologies – like the Oculus Rift, the HTC Vive and the Sony PlayStation VR – and Mixed Reality Interfaces (MRITF) – like the Hololens – is attracting the attention of users and researchers suggesting it may be the next largest stepping stone in technological innovation. However, the history of VR technology is longer than it may seem: the concept of VR was formulated in the 1960s and the first commercial VR tools appeared in the late 1980s. For this reason, during the last 20 years, 100s of researchers explored the processes, effects, and applications of this technology producing 1000s of scientific papers. What is the outcome of this significant research work? This paper wants to provide an answer to this question by exploring, using advanced scientometric techniques, the existing research corpus in the field. We collected all the existent articles about VR in the Web of Science Core Collection scientific database, and the resultant dataset contained 21,667 records for VR and 9,944 for augmented reality (AR). The bibliographic record contained various fields, such as author, title, abstract, country, and all the references (needed for the citation analysis). The network and cluster analysis of the literature showed a composite panorama characterized by changes and evolutions over the time. Indeed, whether until 5 years ago, the main publication media on VR concerned both conference proceeding and journals, more recently journals constitute the main medium of communication. Similarly, if at first computer science was the leading research field, nowadays clinical areas have increased, as well as the number of countries involved in VR research. The present work discusses the evolution and changes over the time of the use of VR in the main areas of application with an emphasis on the future expected VR’s capacities, increases and challenges. We conclude considering the disruptive contribution that VR/AR/MRITF will be able to get in scientific fields, as well in human communication and interaction, as already happened with the advent of mobile phones by increasing the use and the development of scientific applications (e.g., in clinical areas) and by modifying the social communication and interaction among people.

## Introduction

In the last 5 years, virtual reality (VR) and augmented reality (AR) have attracted the interest of investors and the general public, especially after Mark Zuckerberg bought Oculus for two billion dollars (Luckerson, 2014; Castelvecchi, 2016). Currently, many other companies, such as Sony, Samsung, HTC, and Google are making huge investments in VR and AR (Korolov, 2014; Ebert, 2015; Castelvecchi, 2016). However, if VR has been used in research for more than 25 years, and now there are 1000s of papers and many researchers in the field, comprising a strong, interdisciplinary community, AR has a more recent application history (Burdea and Coiffet, 2003; Kim, 2005; Bohil et al., 2011; Cipresso and Serino, 2014; Wexelblat, 2014). The study of VR was initiated in the computer graphics field and has been extended to several disciplines (Sutherland, 1965, 1968; Mazuryk and Gervautz, 1996; Choi et al., 2015). Currently, videogames supported by VR tools are more popular than the past, and they represent valuables, work-related tools for neuroscientists, psychologists, biologists, and other researchers as well. Indeed, for example, one of the main research purposes lies from navigation studies that include complex experiments that could be done in a laboratory by using VR, whereas, without VR, the researchers would have to go directly into the field, possibly with limited use of intervention. The importance of navigation studies for the functional understanding of human memory in dementia has been a topic of significant interest for a long time, and, in 2014, the Nobel Prize in “Physiology or Medicine” was awarded to John M. O’Keefe, May-Britt Moser, and Edvard I. Moser for their discoveries of nerve cells in the brain that enable a sense of place and navigation. Journals and magazines have extended this knowledge by writing about “the brain GPS,” which gives a clear idea of the mechanism. A huge number of studies have been conducted in clinical settings by using VR (Bohil et al., 2011; Serino et al., 2014), and Nobel Prize winner, Edvard I. Moser commented about the use of VR (Minderer et al., 2016), highlighting its importance for research and clinical practice. Moreover, the availability of free tools for VR experimental and computational use has made it easy to access any field (Riva et al., 2011; Cipresso, 2015; Brown and Green, 2016; Cipresso et al., 2016).

Augmented reality is a more recent technology than VR and shows an interdisciplinary application framework, in which, nowadays, education and learning seem to be the most field of research. Indeed, AR allows supporting learning, for example increasing-on content understanding and memory preservation, as well as on learning motivation. However, if VR benefits from clear and more definite fields of application and research areas, AR is still emerging in the scientific scenarios.

In this article, we present a systematic and computational analysis of the emerging interdisciplinary VR and AR fields in terms of various co-citation networks in order to explore the evolution of the intellectual structure of this knowledge domain over time.

### Virtual Reality Concepts and Features

The concept of VR could be traced at the mid of 1960 when Ivan Sutherland in a pivotal manuscript attempted to describe VR as a window through which a user perceives the virtual world as if looked, felt, sounded real and in which the user could act realistically (Sutherland, 1965).

Since that time and in accordance with the application area, several definitions have been formulated: for example, Fuchs and Bishop (1992) defined VR as “real-time interactive graphics with 3D models, combined with a display technology that gives the user the immersion in the model world and direct manipulation” (Fuchs and Bishop, 1992); Gigante (1993) described VR as “The illusion of participation in a synthetic environment rather than external observation of such an environment. VR relies on a 3D, stereoscopic head-tracker displays, hand/body tracking and binaural sound. VR is an immersive, multi-sensory experience” (Gigante, 1993); and “Virtual reality refers to immersive, interactive, multi-sensory, viewer-centered, 3D computer generated environments and the combination of technologies required building environments” (Cruz-Neira, 1993).

As we can notice, these definitions, although different, highlight three common features of VR systems: immersion, perception to be present in an environment, and interaction with that environment (Biocca, 1997; Lombard and Ditton, 1997; Loomis et al., 1999; Heeter, 2000; Biocca et al., 2001; Bailenson et al., 2006; Skalski and Tamborini, 2007; Andersen and Thorpe, 2009; Slater, 2009; Sundar et al., 2010). Specifically, immersion concerns the amount of senses stimulated, interactions, and the reality’s similarity of the stimuli used to simulate environments. This feature can depend on the properties of the technological system used to isolate user from reality (Slater, 2009).

Higher or lower degrees of immersion can depend by three types of VR systems provided to the user:

• Non-immersive systems are the simplest and cheapest type of VR applications that use desktops to reproduce images of the world.
• Immersive systems provide a complete simulated experience due to the support of several sensory outputs devices such as head mounted displays (HMDs) for enhancing the stereoscopic view of the environment through the movement of the user’s head, as well as audio and haptic devices.
• Semi-immersive systems such as Fish Tank VR are between the two above. They provide a stereo image of a three dimensional (3D) scene viewed on a monitor using a perspective projection coupled to the head position of the observer (Ware et al., 1993). Higher technological immersive systems have showed a closest experience to reality, giving to the user the illusion of technological non-mediation and feeling him or her of “being in” or present in the virtual environment (Lombard and Ditton, 1997). Furthermore, higher immersive systems, than the other two systems, can give the possibility to add several sensory outputs allowing that the interaction and actions were perceived as real (Loomis et al., 1999; Heeter, 2000; Biocca et al., 2001).

Finally, the user’s VR experience could be disclosed by measuring presence, realism, and reality’s levels. Presence is a complex psychological feeling of “being there” in VR that involves the sensation and perception of physical presence, as well as the possibility to interact and react as if the user was in the real world (Heeter, 1992). Similarly, the realism’s level corresponds to the degree of expectation that the user has about of the stimuli and experience (Baños et al., 2000, 2009). If the presented stimuli are similar to reality, VR user’s expectation will be congruent with reality expectation, enhancing VR experience. In the same way, higher is the degree of reality in interaction with the virtual stimuli, higher would be the level of realism of the user’s behaviors (Baños et al., 2000, 2009).

### From Virtual to Augmented Reality

Looking chronologically on VR and AR developments, we can trace the first 3D immersive simulator in 1962, when Morton Heilig created Sensorama, a simulated experience of a motorcycle running through Brooklyn characterized by several sensory impressions, such as audio, olfactory, and haptic stimuli, including also wind to provide a realist experience (Heilig, 1962). In the same years, Ivan Sutherland developed The Ultimate Display that, more than sound, smell, and haptic feedback, included interactive graphics that Sensorama didn’t provide. Furthermore, Philco developed the first HMD that together with The Sword of Damocles of Sutherland was able to update the virtual images by tracking user’s head position and orientation (Sutherland, 1965). In the 70s, the University of North Carolina realized GROPE, the first system of force-feedback and Myron Krueger created VIDEOPLACE an Artificial Reality in which the users’ body figures were captured by cameras and projected on a screen (Krueger et al., 1985). In this way two or more users could interact in the 2D-virtual space. In 1982, the US’ Air Force created the first flight simulator [Visually Coupled Airbone System Simulator (VCASS)] in which the pilot through an HMD could control the pathway and the targets. Generally, the 80’s were the years in which the first commercial devices began to emerge: for example, in 1985 the VPL company commercialized the DataGlove, glove sensors’ equipped able to measure the flexion of fingers, orientation and position, and identify hand gestures. Another example is the Eyephone, created in 1988 by the VPL Company, an HMD system for completely immerging the user in a virtual world. At the end of 80’s, Fake Space Labs created a Binocular-Omni-Orientational Monitor (BOOM), a complex system composed by a stereoscopic-displaying device, providing a moving and broad virtual environment, and a mechanical arm tracking. Furthermore, BOOM offered a more stable image and giving more quickly responses to movements than the HMD devices. Thanks to BOOM and DataGlove, the NASA Ames Research Center developed the Virtual Wind Tunnel in order to research and manipulate airflow in a virtual airplane or space ship. In 1992, the Electronic Visualization Laboratory of the University of Illinois created the CAVE Automatic Virtual Environment, an immersive VR system composed by projectors directed on three or more walls of a room.

More recently, many videogames companies have improved the development and quality of VR devices, like Oculus Rift, or HTC Vive that provide a wider field of view and lower latency. In addition, the actual HMD’s devices can be now combined with other tracker system as eye-tracking systems (FOVE), and motion and orientation sensors (e.g., Razer Hydra, Oculus Touch, or HTC Vive).

Simultaneously, at the beginning of 90’, the Boing Corporation created the first prototype of AR system for showing to employees how set up a wiring tool (Carmigniani et al., 2011). At the same time, Rosenberg and Feiner developed an AR fixture for maintenance assistance, showing that the operator performance enhanced by added virtual information on the fixture to repair (Rosenberg, 1993). In 1993 Loomis and colleagues produced an AR GPS-based system for helping the blind in the assisted navigation through adding spatial audio information (Loomis et al., 1998). Always in the 1993 Julie Martin developed “Dancing in Cyberspace,” an AR theater in which actors interacted with virtual object in real time (Cathy, 2011). Few years later, Feiner et al. (1997) developed the first Mobile AR System (MARS) able to add virtual information about touristic buildings (Feiner et al., 1997). Since then, several applications have been developed: in Thomas et al. (2000), created ARQuake, a mobile AR video game; in 2008 was created Wikitude that through the mobile camera, internet, and GPS could add information about the user’s environments (Perry, 2008). In 2009 others AR applications, like AR Toolkit and SiteLens have been developed in order to add virtual information to the physical user’s surroundings. In 2011, Total Immersion developed D’Fusion, and AR system for designing projects (Maurugeon, 2011). Finally, in 2013 and 2015, Google developed Google Glass and Google HoloLens, and their usability have begun to test in several field of application.

### Virtual Reality Technologies

Technologically, the devices used in the virtual environments play an important role in the creation of successful virtual experiences. According to the literature, can be distinguished input and output devices (Burdea et al., 1996; Burdea and Coiffet, 2003). Input devices are the ones that allow the user to communicate with the virtual environment, which can range from a simple joystick or keyboard to a glove allowing capturing finger movements or a tracker able to capture postures. More in detail, keyboard, mouse, trackball, and joystick represent the desktop input devices easy to use, which allow the user to launch continuous and discrete commands or movements to the environment. Other input devices can be represented by tracking devices as bend-sensing gloves that capture hand movements, postures and gestures, or pinch gloves that detect the fingers movements, and trackers able to follow the user’s movements in the physical world and translate them in the virtual environment.

On the contrary, the output devices allow the user to see, hear, smell, or touch everything that happens in the virtual environment. As mentioned above, among the visual devices can be found a wide range of possibilities, from the simplest or least immersive (monitor of a computer) to the most immersive one such as VR glasses or helmets or HMD or CAVE systems.

Furthermore, auditory, speakers, as well as haptic output devices are able to stimulate body senses providing a more real virtual experience. For example, haptic devices can stimulate the touch feeling and force models in the user.

### Virtual Reality Applications

Since its appearance, VR has been used in different fields, as for gaming (Zyda, 2005; Meldrum et al., 2012), military training (Alexander et al., 2017), architectural design (Song et al., 2017), education (Englund et al., 2017), learning and social skills training (Schmidt et al., 2017), simulations of surgical procedures (Gallagher et al., 2005), assistance to the elderly or psychological treatments are other fields in which VR is bursting strongly (Freeman et al., 2017; Neri et al., 2017). A recent and extensive review of Slater and Sanchez-Vives (2016) reported the main VR application evidences, including weakness and advantages, in several research areas, such as science, education, training, physical training, as well as social phenomena, moral behaviors, and could be used in other fields, like travel, meetings, collaboration, industry, news, and entertainment. Furthermore, another review published this year by Freeman et al. (2017) focused on VR in mental health, showing the efficacy of VR in assessing and treating different psychological disorders as anxiety, schizophrenia, depression, and eating disorders.

There are many possibilities that allow the use of VR as a stimulus, replacing real stimuli, recreating experiences, which in the real world would be impossible, with a high realism. This is why VR is widely used in research on new ways of applying psychological treatment or training, for example, to problems arising from phobias (agoraphobia, phobia to fly, etc.) (Botella et al., 2017). Or, simply, it is used like improvement of the traditional systems of motor rehabilitation (Llorens et al., 2014; Borrego et al., 2016), developing games that ameliorate the tasks. More in detail, in psychological treatment, Virtual Reality Exposure Therapy (VRET) has showed its efficacy, allowing to patients to gradually face fear stimuli or stressed situations in a safe environment where the psychological and physiological reactions can be controlled by the therapist (Botella et al., 2017).

### Augmented Reality Concept

Milgram and Kishino (1994), conceptualized the Virtual-Reality Continuum that takes into consideration four systems: real environment, augmented reality (AR), augmented virtuality, and virtual environment. AR can be defined a newer technological system in which virtual objects are added to the real world in real-time during the user’s experience. Per Azuma et al. (2001) an AR system should: (1) combine real and virtual objects in a real environment; (2) run interactively and in real-time; (3) register real and virtual objects with each other. Furthermore, even if the AR experiences could seem different from VRs, the quality of AR experience could be considered similarly. Indeed, like in VR, feeling of presence, level of realism, and the degree of reality represent the main features that can be considered the indicators of the quality of AR experiences. Higher the experience is perceived as realistic, and there is congruence between the user’s expectation and the interaction inside the AR environments, higher would be the perception of “being there” physically, and at cognitive and emotional level. The feeling of presence, both in AR and VR environments, is important in acting behaviors like the real ones (Botella et al., 2005; Juan et al., 2005; Bretón-López et al., 2010; Wrzesien et al., 2013).

### Augmented Reality Technologies

Technologically, the AR systems, however various, present three common components, such as a geospatial datum for the virtual object, like a visual marker, a surface to project virtual elements to the user, and an adequate processing power for graphics, animation, and merging of images, like a pc and a monitor (Carmigniani et al., 2011). To run, an AR system must also include a camera able to track the user movement for merging the virtual objects, and a visual display, like glasses through that the user can see the virtual objects overlaying to the physical world. To date, two-display systems exist, a video see-through (VST) and an optical see-though (OST) AR systems (Botella et al., 2005; Juan et al., 2005, 2007). The first one, disclosures virtual objects to the user by capturing the real objects/scenes with a camera and overlaying virtual objects, projecting them on a video or a monitor, while the second one, merges the virtual object on a transparent surface, like glasses, through the user see the added elements. The main difference between the two systems is the latency: an OST system could require more time to display the virtual objects than a VST system, generating a time lag between user’s action and performance and the detection of them by the system.

### Augmented Reality Applications

Although AR is a more recent technology than VR, it has been investigated and used in several research areas such as architecture (Lin and Hsu, 2017), maintenance (Schwald and De Laval, 2003), entertainment (Ozbek et al., 2004), education (Nincarean et al., 2013; Bacca et al., 2014; Akçayır and Akçayır, 2017), medicine (De Buck et al., 2005), and psychological treatments (Juan et al., 2005; Botella et al., 2005, 2010; Bretón-López et al., 2010; Wrzesien et al., 2011a,b, 2013; see the review Chicchi Giglioli et al., 2015). More in detail, in education several AR applications have been developed in the last few years showing the positive effects of this technology in supporting learning, such as an increased-on content understanding and memory preservation, as well as on learning motivation (Radu, 2012, 2014). For example, Ibáñez et al. (2014) developed a AR application on electromagnetism concepts’ learning, in which students could use AR batteries, magnets, cables on real superficies, and the system gave a real-time feedback to students about the correctness of the performance, improving in this way the academic success and motivation (Di Serio et al., 2013). Deeply, AR system allows the possibility to learn visualizing and acting on composite phenomena that traditionally students study theoretically, without the possibility to see and test in real world (Chien et al., 2010; Chen et al., 2011).

As well in psychological health, the number of research about AR is increasing, showing its efficacy above all in the treatment of psychological disorder (see the reviews Baus and Bouchard, 2014; Chicchi Giglioli et al., 2015). For example, in the treatment of anxiety disorders, like phobias, AR exposure therapy (ARET) showed its efficacy in one-session treatment, maintaining the positive impact in a follow-up at 1 or 3 month after. As VRET, ARET provides a safety and an ecological environment where any kind of stimulus is possible, allowing to keep control over the situation experienced by the patients, gradually generating situations of fear or stress. Indeed, in situations of fear, like the phobias for small animals, AR applications allow, in accordance with the patient’s anxiety, to gradually expose patient to fear animals, adding new animals during the session or enlarging their or increasing the speed. The various studies showed that AR is able, at the beginning of the session, to activate patient’s anxiety, for reducing after 1 h of exposition. After the session, patients even more than to better manage animal’s fear and anxiety, ware able to approach, interact, and kill real feared animals.

## Materials and Methods

### Data Collection

The input data for the analyses were retrieved from the scientific database Web of Science Core Collection (Falagas et al., 2008) and the search terms used were “Virtual Reality” and “Augmented Reality” regarding papers published during the whole timespan covered.

*Web of science core collection is composed of:* Citation Indexes, Science Citation Index Expanded (SCI-EXPANDED) –1970-present, Social Sciences Citation Index (SSCI) –1970-present, Arts and Humanities Citation Index (A&HCI) –1975-present, Conference Proceedings Citation Index- Science (CPCI-S) –1990-present, Conference Proceedings Citation Index- Social Science & Humanities (CPCI-SSH) –1990-present, Book Citation Index– Science (BKCI-S) –2009-present, Book Citation Index– Social Sciences & Humanities (BKCI-SSH) –2009-present, Emerging Sources Citation Index (ESCI) –2015-present, Chemical Indexes, Current Chemical Reactions (CCR-EXPANDED) –2009-present (Includes Institut National de la Propriete Industrielle structure data back to 1840), Index Chemicus (IC) –2009-present.

The resultant dataset contained a total of 21,667 records for VR and 9,944 records for AR. The bibliographic record contained various fields, such as author, title, abstract, and all of the references (needed for the citation analysis). The research tool to visualize the networks was Cite space v.4.0.R5 SE (32 bit) (Chen, 2006) under Java Runtime v.8 update 91 (build 1.8.0_91-b15). Statistical analyses were conducted using Stata MP-Parallel Edition, Release 14.0, StataCorp LP. Additional information can be found in Supplementary Data Sheet 1.

The betweenness centrality of a node in a network measures the extent to which the node is part of paths that connect an arbitrary pair of nodes in the network (Freeman, 1977; Brandes, 2001; Chen, 2006).

Structural metrics include betweenness centrality, modularity, and silhouette. Temporal and hybrid metrics include citation burstness and novelty. All the algorithms are detailed (Chen et al., 2010).

## Results

The analysis of the literature on VR shows a complex panorama. At first sight, according to the document-type statistics from the Web of Science (WoS), proceedings papers were used extensively as outcomes of research, comprising almost 48% of the total (10,392 proceedings), with a similar number of articles on the subject amounting to about 47% of the total of 10, 199 articles. However, if we consider only the last 5 years (7,755 articles representing about 36% of the total), the situation changes with about 57% for articles (4,445) and about 33% for proceedings (2,578). Thus, it is clear that VR field has changed in areas other than at the technological level.

About the subject category, nodes and edges are computed as co-occurring subject categories from the Web of Science “Category” field in all the articles.

**FIGURE 1:**
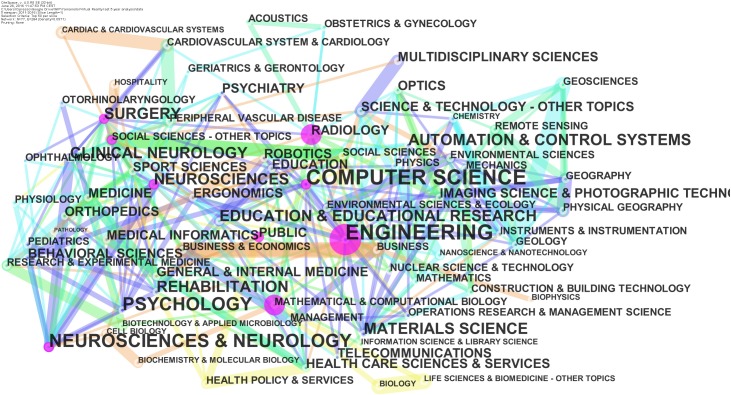
Category from the WoS: network for the last 5 years.

According to the subject category statistics from the WoS, computer science is the leading category, followed by engineering, and, together, they account for 15,341 articles, which make up about 71% of the total production. However, if we consider just the last 5 years, these categories reach only about 55%, with a total of 4,284 articles (Table 1 and Figure 1).

**Table 1:** Category statistics from the WoS for the entire period and the last 5 years.

%	Frequency	Subject category (for all the period)
42,15	9131	Computer Science, 1990–2016
28,66	6210	Engineering, 1990–2016
8,21	1779	Psychology, 1990–2016
7,15	1548	Neurosciences and Neurology, 1992–2016
6,55	1418	Surgery, 1992–2016
5,85	1267	Automation and Control Systems, 1993–2016
4,80	1040	Neurosciences, 1992–2016
4,74	1027	Imaging Science and Photographic Technology, 1992–2016
4,30	931	Education and Educational Research, 1993–2016
3,92	849	Robotics, 1992–2016

**%**	**Frequency**	**Subject category (for the last 5 years)**

29,80	2311	Computer Science, 2011–2016
25,44	1973	Engineering, 2011–2016
11,10	861	Neurosciences and Neurology, 2011–2016
9,32	723	Psychology, 2011–2016
7,70	597	Surgery, 2011–2016
7,53	584	Neurosciences, 2011–2016
6,02	467	Education and Educational Research, 2011–2016
5,54	430	Rehabilitation, 2011–2016
4,42	343	Clinical Neurology, 2011–2016
3,92	304	Materials Science, 2011–2016


The evidence is very interesting since it highlights that VR is doing very well as new technology with huge interest in hardware and software components. However, with respect to the past, we are witnessing increasing numbers of applications, especially in the medical area. In particular, note its inclusion in the top 10 list of rehabilitation and clinical neurology categories (about 10% of the total production in the last 5 years). It also is interesting that neuroscience and neurology, considered together, have shown an increase from about 12% to about 18.6% over the last 5 years. However, historic areas, such as automation and control systems, imaging science and photographic technology, and robotics, which had accounted for about 14.5% of the total articles ever produced were not even in the top 10 for the last 5 years, with each one accounting for less than 4%.

About the countries, nodes and edges are computed as networks of co-authors countries. Multiple occurrency of a country in the same paper are counted once.

**FIGURE 2:**
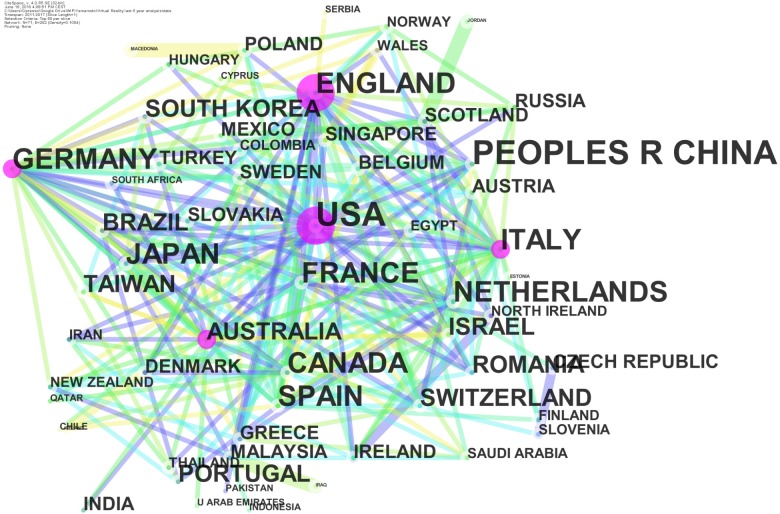
Country network (node dimension represents centrality).

The countries that were very involved in VR research have published for about 47% of the total (10,200 articles altogether). Of the 10,200 articles, the United States, China, England, and Germany published 4921, 2384, 1497, and 1398, respectively. The situation remains the same if we look at the articles published over the last 5 years. However, VR contributions also came from all over the globe, with Japan, Canada, Italy, France, Spain, South Korea, and Netherlands taking positions of prominence, as shown in Figure 2.

Network analysis was conducted to calculate and to represent the centrality index (Freeman, 1977; Brandes, 2001), i.e., the dimension of the node in Figure 2. The top-ranked country, with a centrality index of 0.26, was the United States (2011), and England was second, with a centrality index of 0.25. The third, fourth, and fifth countries were Germany, Italy, and Australia, with centrality indices of 0.15, 0.15, and 0.14, respectively.

**FIGURE 3:**
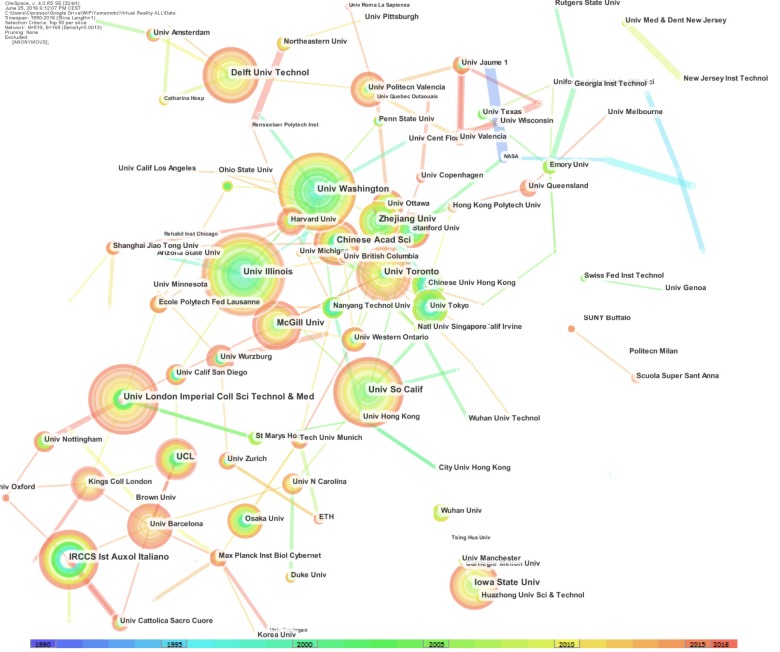
Network of institutions: the dimensions of the nodes represent centrality.

About the Institutions, nodes and edges are computed as networks of co-authors Institutions (Figure 3).

The top-level institutions in VR were in the United States, where three universities were ranked as the top three in the world for published articles; these universities were the University of Illinois (159), the University of South California (147), and the University of Washington (146). The United States also had the eighth-ranked university, which was Iowa State University (116). The second country in the ranking was Canada, with the University of Toronto, which was ranked fifth with 125 articles and McGill University, ranked 10^th^ with 103 articles.

Other countries in the top-ten list were Netherlands, with the Delft University of Technology ranked fourth with 129 articles; Italy, with IRCCS Istituto Auxologico Italiano, ranked sixth (with the same number of publication of the institution ranked fifth) with 125 published articles; England, which was ranked seventh with 125 articles from the University of London’s Imperial College of Science, Technology, and Medicine; and China with 104 publications, with the Chinese Academy of Science, ranked ninth. Italy’s Istituto Auxologico Italiano, which was ranked fifth, was the only non-university institution ranked in the top-10 list for VR research (Figure 3).

About the Journals, nodes, and edges are computed as journal co-citation networks among each journals in the corresponding field.

The top-ranked Journals for citations in VR are *Presence: Teleoperators & Virtual Environments* with 2689 citations and *CyberPsychology & Behavior* (Cyberpsychol BEHAV) with 1884 citations; however, looking at the last 5 years, the former had increased the citations, but the latter had a far more significant increase, from about 70% to about 90%, i.e., an increase from 1029 to 1147.

**FIGURE 4:**
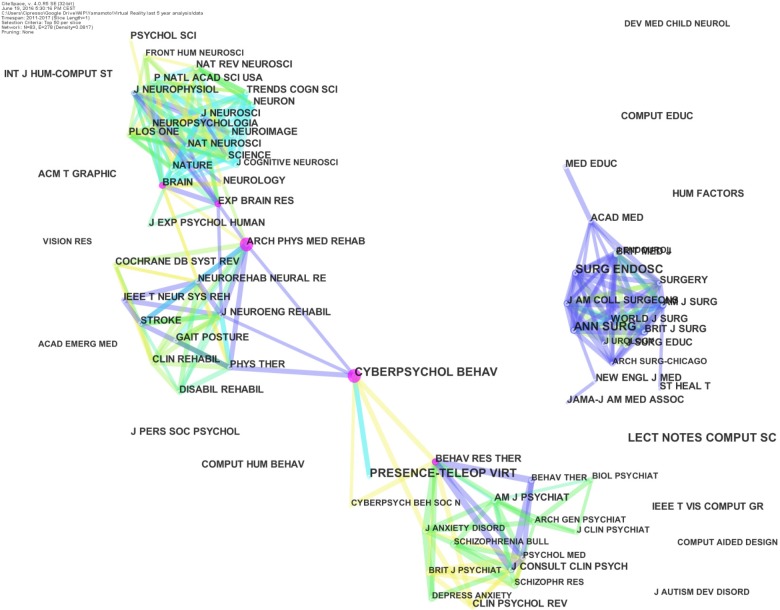
Co-citation network of journals: the dimensions of the nodes represent centrality. Full list of official abbreviations of WoS journals can be found here: https://images.webofknowledge.com/images/help/WOS/A_abrvjt.html.

Following the top two journals, IEEE Computer Graphics and Applications (*IEEE Comput Graph)* and Advanced Health Telematics and Telemedicine (*St HEAL T)* were both left out of the top-10 list based on the last 5 years. The data for the last 5 years also resulted in the inclusion of Experimental Brain Research (*Exp BRAIN RES)* (625 citations), Archives of Physical Medicine and Rehabilitation (*Arch PHYS MED REHAB)* (622 citations), and *Plos ONE* (619 citations) in the top-10 list of three journals, which highlighted the categories of rehabilitation and clinical neurology and neuroscience and neurology. Journal co-citation analysis is reported in Figure 4, which clearly shows four distinct clusters.

Network analysis was conducted to calculate and to represent the centrality index, i.e., the dimensions of the nodes in Figure 4. The top-ranked item by centrality was Cyberpsychol BEHAV, with a centrality index of 0.29. The second-ranked item was Arch PHYS MED REHAB, with a centrality index of 0.23. The third was Behaviour Research and Therapy (Behav RES THER), with a centrality index of 0.15. The fourth was BRAIN, with a centrality index of 0.14. The fifth was Exp BRAIN RES, with a centrality index of 0.11.

### Who’s Who in VR Research

**FIGURE 5:**
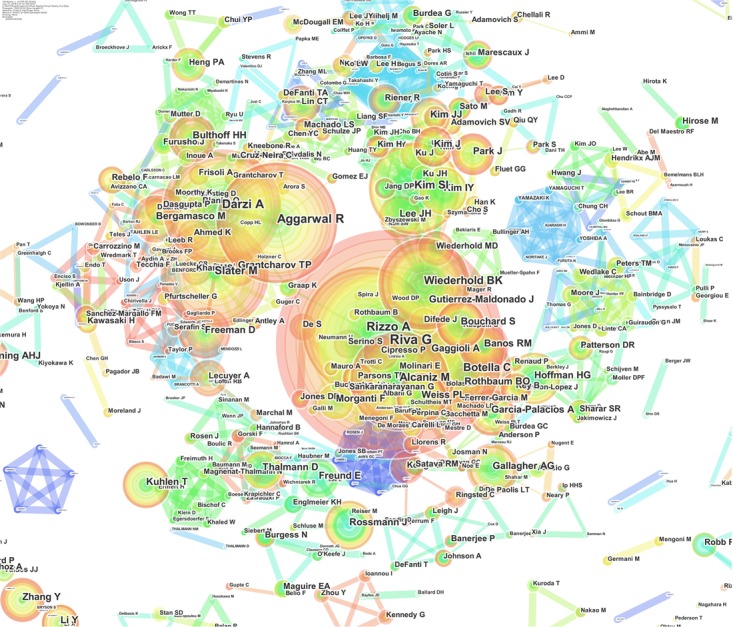
Network of authors’ numbers of publications: the dimensions of the nodes represent the centrality index, and the dimensions of the characters represent the author’s rank.

Authors are the heart and brain of research, and their roles in a field are to define the past, present, and future of disciplines and to make significant breakthroughs to make new ideas arise (Figure 5).

Virtual reality research is very young and changing with time, but the top-10 authors in this field have made fundamentally significant contributions as pioneers in VR and taking it beyond a mere technological development. The purpose of the following highlights is not to rank researchers; rather, the purpose is to identify the most active researchers in order to understand where the field is going and how they plan for it to get there.

**FIGURE 6:**
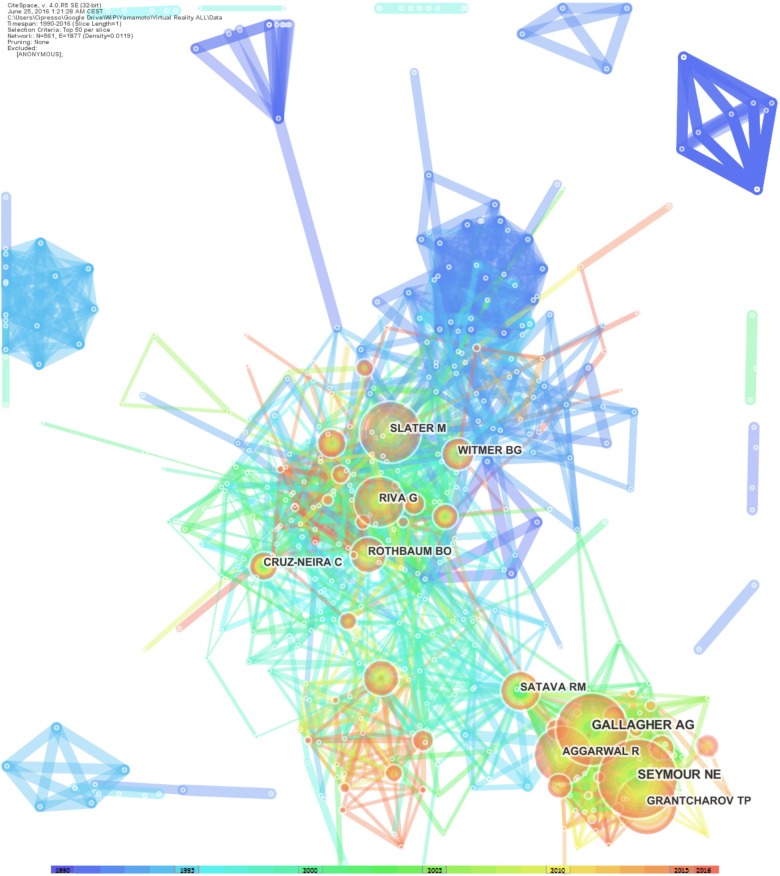
Authors’ co-citation network: the dimensions of the nodes represent centrality index, and the dimensions of the characters represent the author’s rank. The 10 authors that appear on the top-10 list are considered to be the pioneers of VR research.

The top-ranked author is Riva G, with 180 publications. The second-ranked author is Rizzo A, with 101 publications. The third is Darzi A, with 97 publications. The forth is Aggarwal R, with 94 publications. The six authors following these three are Slater M, Alcaniz M, Botella C, Wiederhold BK, Kim SI, and Gutierrez-Maldonado J with 90, 90, 85, 75, 59, and 54 publications, respectively (Figure 6).

Considering the last 5 years, the situation remains similar, with three new entries in the top-10 list, i.e., Muhlberger A, Cipresso P, and Ahmed K ranked 7th, 8th, and 10th, respectively.

The authors’ publications number network shows the most active authors in VR research. Another relevant analysis for our focus on VR research is to identify the most cited authors in the field.

For this purpose, the authors’ co-citation analysis highlights the authors in term of their impact on the literature considering the entire time span of the field (White and Griffith, 1981; González-Teruel et al., 2015; Bu et al., 2016). The idea is to focus on the dynamic nature of the community of authors who contribute to the research.

Normally, authors with higher numbers of citations tend to be the scholars who drive the fundamental research and who make the most meaningful impacts on the evolution and development of the field. In the following, we identified the most-cited pioneers in the field of VR Research.

The top-ranked author by citation count is Gallagher (2001), with 694 citations. Second is Seymour (2004), with 668 citations. Third is Slater (1999), with 649 citations. Fourth is Grantcharov (2003), with 563 citations. Fifth is Riva (1999), with 546 citations. Sixth is Aggarwal (2006), with 505 citations. Seventh is Satava (1994), with 477 citations. Eighth is Witmer (2002), with 454 citations. Ninth is Rothbaum (1996), with 448 citations. Tenth is Cruz-neira (1995), with 416 citations.

### Citation Network and Cluster Analyses for VR

Another analysis that can be used is the analysis of document co-citation, which allows us to focus on the highly-cited documents that generally are also the most influential in the domain (Small, 1973; González-Teruel et al., 2015; Orosz et al., 2016).

The top-ranked article by citation counts is Seymour (2002) in Cluster #0, with 317 citations. The second article is Grantcharov (2004) in Cluster #0, with 286 citations. The third is Holden (2005) in Cluster #2, with 179 citations. The 4th is Gallagher et al. (2005) in Cluster #0, with 171 citations. The 5th is Ahlberg (2007) in Cluster #0, with 142 citations. The 6th is Parsons (2008) in Cluster #4, with 136 citations. The 7th is Powers (2008) in Cluster #4, with 134 citations. The 8th is Aggarwal (2007) in Cluster #0, with 121 citations. The 9th is Reznick (2006) in Cluster #0, with 121 citations. The 10th is Munz (2004) in Cluster #0, with 117 citations.

**FIGURE 7:**
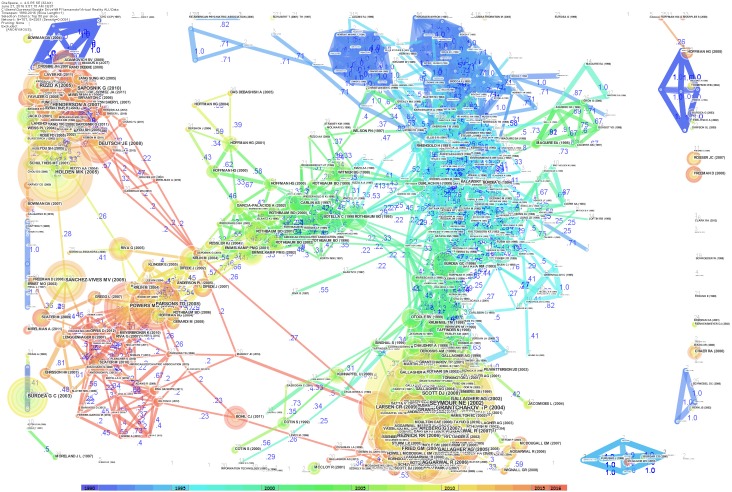
Network of document co-citations: the dimensions of the nodes represent centrality, the dimensions of the characters represent the rank of the article rank, and the numbers represent the strengths of the links. It is possible to identify four historical phases (colors: blue, green, yellow, and red) from the past VR research to the current research.

**FIGURE 8:**
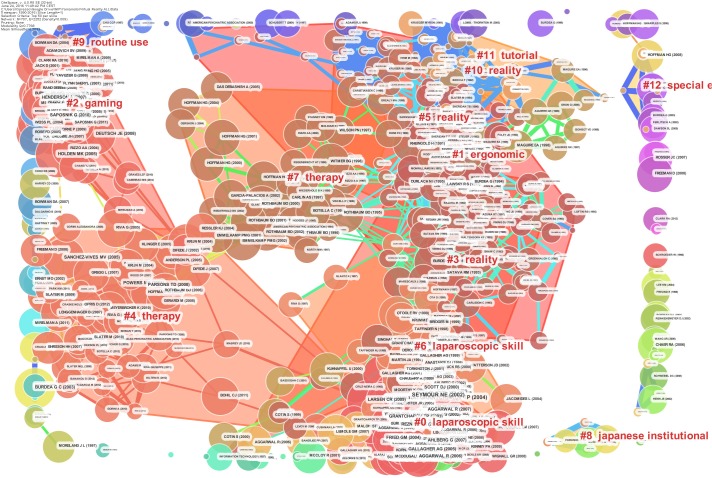
Document co-citation network by cluster: the dimensions of the nodes represent centrality, the dimensions of the characters represent the rank of the article rank and the red writing reports the name of the cluster with a short description that was produced with the mutual information algorithm; the clusters are identified with colored polygons.

The network of document co-citations is visually complex (Figure 7) because it includes 1000s of articles and the links among them. However, this analysis is very important because can be used to identify the possible conglomerate of knowledge in the area, and this is essential for a deep understanding of the area. Thus, for this purpose, a cluster analysis was conducted (Chen et al., 2010; González-Teruel et al., 2015; Klavans and Boyack, 2015). Figure 8 shows the clusters, which are identified with the two algorithms in Table 2.

**Table 2:** Cluster ID and silhouettes as identified with two algorithms (Chen et al., 2010).

ID	Size	Silho-uette	Mean (Citee Year)	Label (TFIDF, tf^∗^idf weighting algorithm)	Label (LLR, log-likelihood ratio, p-level)
0	84	0.812	2005	(25.82) laparoscopic skill; (25.01) proficiency; (24.5) basic laparoscopic skill; (24.14) trainer; (23.79) establishing validity	Training (143.21, 1.0E-4); performance (73.38, 1.0E-4); laparoscopic skill (72.93, 1.0E-4)
1	77	0.758	1992	(17.76) ergonomic; (17.66) reality; (16.83) virtual reality; (16.04) virtual environment; (15.76) assembly	Ergonomic (54.1, 1.0E-4); virtual reality interface (34.63, 1.0E-4); developing virtual environment (34.48, 1.0E-4)
2	62	0.992	2007	(24.5) gaming; (24.5) wii; (24.47) stroke; (23.07) rehabilitation; (22.38) cerebral palsy	Stroke (82.9, 1.0E-4); children (75.13, 1.0E-4); stroke rehabilitation (57.95, 1.0E-4)
3	61	0.758	1994	(15) reality; (14.66) virtual reality; (14.25) surgery; (14.1) telemedical information society; (13.73) chemistry	Telemedical information society (34.85, 1.0E-4); gaining insight (23.21, 1.0E-4); next decade (18.32, 1.0E-4)
4	56	0.934	2008	(25.4) therapy; (23.55) exposure therapy; (22.41) disorder; (21.63) virtual reality exposure therapy; (20.99) post-traumatic stress	Treatment (109.92, 1.0E-4); post-traumatic stress disorder (78.95, 1.0E-4); virtual reality exposure therapy (66.15, 1.0E-4)
5	49	0.885	1992	(16.03) reality; (15.31) virtual reality; (15.01) autistic children; (12.79) child; (12.79) children	Autistic children (29.81, 1.0E-4); possibilities (23.84, 1.0E-4); communication (22.08, 1.0E-4)
6	41	0.855	1998	(17.6) laparoscopic skill; (16.95) direct observation; (16.95) measuring operative performance; (16.95) videotape; (16.15) measuring	Laparoscopic skills training (52.73, 1.0E-4); measuring operative performance (40.97, 1.0E-4); videotape (40.97, 1.0E-4)
7	41	0.946	1998	(20.71) therapy; (18.76) exposure therapy; (17.85) exposure; (17.35) anxiety; (17.2) virtual reality exposure therapy	Virtual reality exposure therapy (32.01, 1.0E-4); spider phobia (27.67, 1.0E-4); ptsd vietnam veteran (22.12, 1.0E-4)
8	38	1	1989	(30.67) Japanese institutional mechanism; (30.67) systems perspective; (20.88) mechanism; (19.25) perspective; (17.97) system	Japanese institutional mechanism (615.45, 1.0E-4); systems perspective (615.45, 1.0E-4); virtual reality (16.28, 1.0E-4)
9	21	1	1987	(23.27) routine use; (23.27) current application; (23.27) behavioral-assessment; (23.27) obstacle; (23.27) future possibilities	Future possibilities (168.77, 1.0E-4); routine use (168.77, 1.0E-4); current application (168.77, 1.0E-4)
10	18	0.934	1991	(12.45) reality; (12.26) virtual-reality; (9.73) medicine; (9.07) virtual reality; (5.71) technology	Virtual-reality (88.95, 1.0E-4); medicine (34.87, 1.0E-4); pretty interface (9.63, 0.005)
11	16	0.937	1990	(13.37) tutorial; (12.45) reality; (11.98) virtual reality; (11.12) virtual reality technology; (10.78) technology	Tutorial (51.15, 1.0E-4); virtual reality technology (44.66, 1.0E-4); space (16.78, 1.0E-4)
12	12	1	1988	(20.05) special effect; (20.05) cyberspace; (13.65) space; (11.38) effect; (10.73) reality	Special effect (128.6, 1.0E-4); cyberspace (128.6, 1.0E-4); virtual reality (27.79, 1.0E-4)
13	8	0.995	1997	(14.88) neural substrate; (14.88) human spatial navigation; (14.88) cognitive map; (11.56) navigation; (10.64) cognitive	Neural substrate (72.6, 1.0E-4); human spatial navigation (66.58, 1.0E-4); cognitive map (66.58, 1.0E-4)
14	6	0.993	2008	(12.06) neurosurgery; (9.74) computer technology; (9.74) surgical application; (9.43) surgery; (8.55) teaching	Neurosurgery (28.72, 1.0E-4); computer technology (18.1, 1.0E-4); surgical application (18.1, 1.0E-4)


**FIGURE 9:**
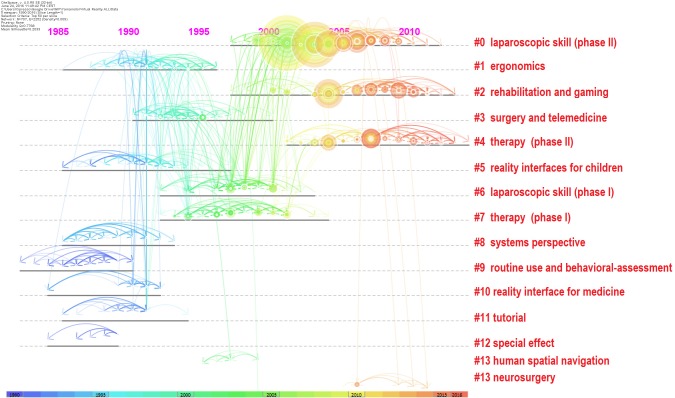
Network of document co-citation: the dimensions of the nodes represent centrality, the dimensions of the characters represent the rank of the article rank and the red writing on the right hand side reports the number of the cluster, such as in Table 2, with a short description that was extracted accordingly.

The identified clusters highlight clear parts of the literature of VR research, making clear and visible the interdisciplinary nature of this field. However, the dynamics to identify the past, present, and future of VR research cannot be clear yet. We analysed the relationships between these clusters and the temporal dimensions of each article. The results are synthesized in Figure 9. It is clear that cluster #0 (laparoscopic skill), cluster #2 (gaming and rehabilitation), cluster #4 (therapy), and cluster #14 (surgery) are the most popular areas of VR research. (See Figure 9 and Table 2 to identify the clusters.) From Figure 9, it also is possible to identify the first phase of laparoscopic skill (cluster #6) and therapy (cluster #7). More generally, it is possible to identify four historical phases (colors: blue, green, yellow, and red) from the past VR research to the current research.

We were able to identify the top 486 references that had the most citations by using burst citations algorithm. Citation burst is an indicator of a most active area of research. Citation burst is a detection of a burst event, which can last for multiple years as well as a single year. A citation burst provides evidence that a particular publication is associated with a surge of citations. The burst detection was based on Kleinberg’s algorithm (Kleinberg, 2002, 2003). The top-ranked document by bursts is Seymour (2002) in Cluster #0, with bursts of 88.93. The second is Grantcharov (2004) in Cluster #0, with bursts of 51.40. The third is Saposnik (2010) in Cluster #2, with bursts of 40.84. The fourth is Rothbaum (1995) in Cluster #7, with bursts of 38.94. The fifth is Holden (2005) in Cluster #2, with bursts of 37.52. The sixth is Scott (2000) in Cluster #0, with bursts of 33.39. The seventh is Saposnik (2011) in Cluster #2, with bursts of 33.33. The eighth is Burdea et al. (1996) in Cluster #3, with bursts of 32.42. The ninth is Burdea and Coiffet (2003) in Cluster #22, with bursts of 31.30. The 10th is Taffinder (1998) in Cluster #6, with bursts of 30.96 (Table 3).

**Table 3:** Cluster ID and references of burst article.

Cluster	Reference	Year	Strength	Begin	End	1990–2016
7	Rothbaum, 1995, Am J Psychiat, V152, P626	1995	38,94	1996	2003	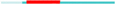
3	Burdea et al., 1996, Force Touch Feedback, V, P	1996	32,42	1997	2004	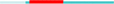
6	Taffinder, 1998, St Heal T, V50, P124	1998	30,96	2000	2006	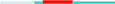
0	Scott, 2000, J Am Coll Surgeons, V191, P272, Doi	2000	33,39	2003	2008	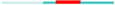
0	Seymour, 2002, Ann Surg, V236, P458, Doi	2002	88,93	2004	2010	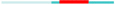
22	Burdea and Coiffet, 2003, Virtual Reality Tech, V, P	2003	31,30	2004	2010	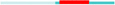
0	Grantcharov, 2004, Brit J Surg, V91, P146, Doi	2004	51,40	2005	2012	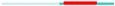
2	Holden, 2005, Cyberpsychol Behav, V8, P187, Doi	2005	37,52	2007	2013	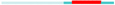
2	Saposnik, 2010, Stroke, V41, P1477, Doi	2010	40,84	2012	2016	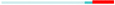
2	Saposnik, 2011, Stroke, V42, P1380, Doi	2011	33,33	2012	2016	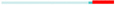


### Citation Network and Cluster Analyses for AR

Looking at Augmented Reality scenario, the top ranked item by citation counts is Azuma (1997) in Cluster #0, with citation counts of 231. The second one is Azuma et al. (2001) in Cluster #0, with citation counts of 220. The third is Van Krevelen (2010) in Cluster #5, with citation counts of 207. The 4th is Lowe (2004) in Cluster #1, with citation counts of 157. The 5th is Wu (2013) in Cluster #4, with citation counts of 144. The 6th is Dunleavy (2009) in Cluster #4, with citation counts of 122. The 7th is Zhou (2008) in Cluster #5, with citation counts of 118. The 8th is Bay (2008) in Cluster #1, with citation counts of 117. The 9th is Newcombe (2011) in Cluster #1, with citation counts of 109. The 10th is Carmigniani et al. (2011) in Cluster #5, with citation counts of 104.

**FIGURE 10:**
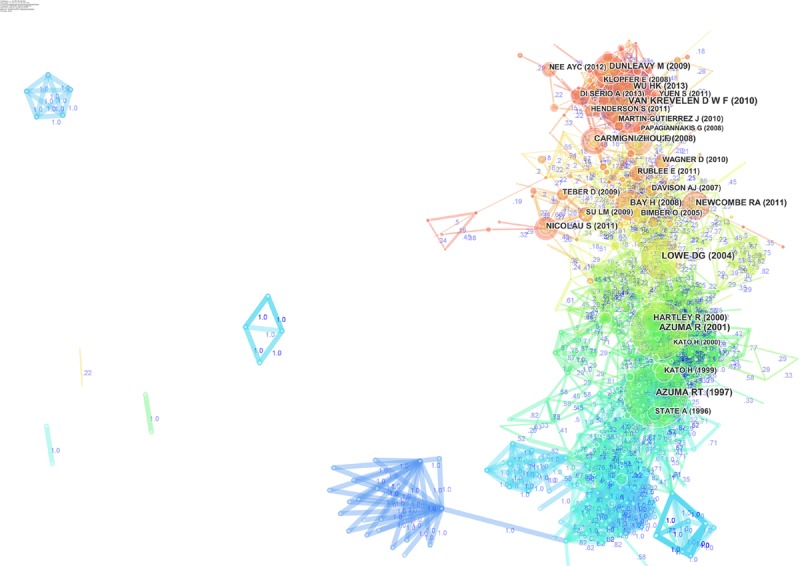
Network of document co-citations: the dimensions of the nodes represent centrality, the dimensions of the characters represent the rank of the article rank, and the numbers represent the strengths of the links. It is possible to identify four historical phases (colors: blue, green, yellow, and red) from the past AR research to the current research.

**FIGURE 11:**
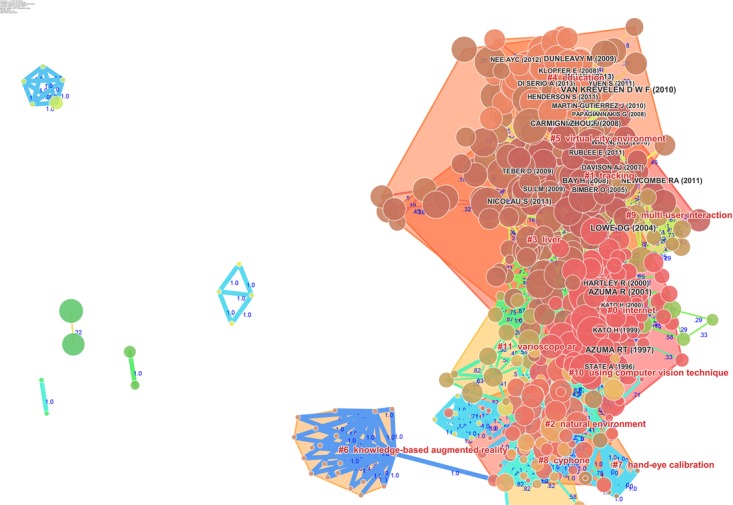
Document co-citation network by cluster: the dimensions of the nodes represent centrality, the dimensions of the characters represent the rank of the article rank and the red writing reports the name of the cluster with a short description that was produced with the mutual information algorithm; the clusters are identified with colored polygons.

The network of document co-citations is visually complex (Figure 10) because it includes 1000s of articles and the links among them. However, this analysis is very important because can be used to identify the possible conglomerate of knowledge in the area, and this is essential for a deep understanding of the area. Thus, for this purpose, a cluster analysis was conducted (Chen et al., 2010; González-Teruel et al., 2015; Klavans and Boyack, 2015). Figure 11 shows the clusters, which are identified with the two algorithms in Table 3.

**FIGURE 12:**
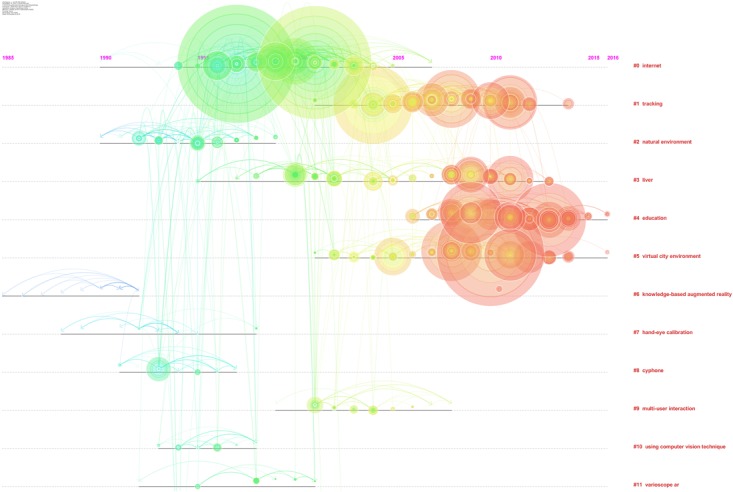
Network of document co-citation: the dimensions of the nodes represent centrality, the dimensions of the characters represent the rank of the article rank and the red writing on the right hand side reports the number of the cluster, such as in Table 2, with a short description that was extracted accordingly.

The identified clusters highlight clear parts of the literature of AR research, making clear and visible the interdisciplinary nature of this field. However, the dynamics to identify the past, present, and future of AR research cannot be clear yet. We analysed the relationships between these clusters and the temporal dimensions of each article. The results are synthesized in Figure 12. It is clear that cluster #1 (tracking), cluster #4 (education), and cluster #5 (virtual city environment) are the current areas of AR research. (See Figure 12 and Table 3 to identify the clusters.) It is possible to identify four historical phases (colors: blue, green, yellow, and red) from the past AR research to the current research.

We were able to identify the top 394 references that had the most citations by using burst citations algorithm. Citation burst is an indicator of a most active area of research. Citation burst is a detection of a burst event, which can last for multiple years as well as a single year. A citation burst provides evidence that a particular publication is associated with a surge of citations. The burst detection was based on Kleinberg’s algorithm (Kleinberg, 2002, 2003). The top ranked document by bursts is Azuma (1997) in Cluster #0, with bursts of 101.64. The second one is Azuma et al. (2001) in Cluster #0, with bursts of 84.23. The third is Lowe (2004) in Cluster #1, with bursts of 64.07. The 4th is Van Krevelen (2010) in Cluster #5, with bursts of 50.99. The 5th is Wu (2013) in Cluster #4, with bursts of 47.23. The 6th is Hartley (2000) in Cluster #0, with bursts of 37.71. The 7th is Dunleavy (2009) in Cluster #4, with bursts of 33.22. The 8th is Kato (1999) in Cluster #0, with bursts of 32.16. The 9th is Newcombe (2011) in Cluster #1, with bursts of 29.72. The 10th is Feiner (1993) in Cluster #8, with bursts of 29.46 (Table 4).

**Table 4:** Cluster ID and silhouettes as identified with two algorithms (Chen et al., 2010).

ID	Size	Silho-uette	Mean (Citee Year)	Label (TFIDF, tf^∗^idf weighting algorithm)	Label (LLR, log-likelihood ratio, p-level)
0	122	0.669	1999	(18.41) internet	Internet (39.96, 1.0E-4)
1	66	0.806	2007	(16.67) tracking	Mobile phone (47.52, 1.0E-4)
2	65	0.827	1994	(17.48) natural environment	Natural feature tracking (57.72, 1.0E-4)
3	56	0.89	2004	(17.33) liver	Laparoscopic surgery (30.43, 1.0E-4)
4	50	0.943	2011	(19.32) education	Education (64.26, 1.0E-4)
5	48	0.86	2007	(15.96) virtual city environment	Virtual city environment (32.68, 1.0E-4)
6	20	0.997	1989	(21.65) knowledge-based augmented reality	Knowledge-based augmented reality (250.67, 1.0E-4)
7	19	0.926	1992	(19.32) hand-eye calibration	Hand–eye calibration (104.98, 1.0E-4)


## Discussion

Our findings have profound implications for two reasons. At first the present work highlighted the evolution and development of VR and AR research and provided a clear perspective based on solid data and computational analyses. Secondly our findings on VR made it profoundly clear that the clinical dimension is one of the most investigated ever and seems to increase in quantitative and qualitative aspects, but also include technological development and article in computer science, engineer, and allied sciences.

Figure 9 clarifies the past, present, and future of VR research. The outset of VR research brought a clearly-identifiable development in interfaces for children and medicine, routine use and behavioral-assessment, special effects, systems perspectives, and tutorials. This pioneering era evolved in the period that we can identify as the development era, because it was the period in which VR was used in experiments associated with new technological impulses. Not surprisingly, this was exactly concomitant with the new economy era in which significant investments were made in information technology, and it also was the era of the so-called ‘dot-com bubble’ in the late 1990s. The confluence of pioneering techniques into ergonomic studies within this development era was used to develop the first effective clinical systems for surgery, telemedicine, human spatial navigation, and the first phase of the development of therapy and laparoscopic skills. With the new millennium, VR research switched strongly toward what we can call the clinical-VR era, with its strong emphasis on rehabilitation, neurosurgery, and a new phase of therapy and laparoscopic skills. The number of applications and articles that have been published in the last 5 years are in line with the new technological development that we are experiencing at the hardware level, for example, with so many new, HMDs, and at the software level with an increasing number of independent programmers and VR communities.

Finally, Figure 12 identifies clusters of the literature of AR research, making clear and visible the interdisciplinary nature of this field. The dynamics to identify the past, present, and future of AR research cannot be clear yet, but analyzing the relationships between these clusters and the temporal dimensions of each article tracking, education, and virtual city environment are the current areas of AR research. AR is a new technology that is showing its efficacy in different research fields, and providing a novel way to gather behavioral data and support learning, training, and clinical treatments.

Looking at scientific literature conducted in the last few years, it might appear that most developments in VR and AR studies have focused on clinical aspects. However, the reality is more complex; thus, this perception should be clarified. Although researchers publish studies on the use of VR in clinical settings, each study depends on the technologies available. Industrial development in VR and AR changed a lot in the last 10 years. In the past, the development involved mainly hardware solutions while nowadays, the main efforts pertain to the software when developing virtual solutions. Hardware became a commodity that is often available at low cost. On the other hand, software needs to be customized each time, per each experiment, and this requires huge efforts in term of development. Researchers in AR and VR today need to be able to adapt software in their labs.

Virtual reality and AR developments in this new clinical era rely on computer science and vice versa. The future of VR and AR is becoming more technological than before, and each day, new solutions and products are coming to the market. Both from software and hardware perspectives, the future of AR and VR depends on huge innovations in all fields. The gap between the past and the future of AR and VR research is about the “realism” that was the key aspect in the past versus the “interaction” that is the key aspect now. First 30 years of VR and AR consisted of a continuous research on better resolution and improved perception. Now, researchers already achieved a great resolution and need to focus on making the VR as realistic as possible, which is not simple. In fact, a real experience implies a realistic interaction and not just great resolution. Interactions can be improved in infinite ways through new developments at hardware and software levels.

Interaction in AR and VR is going to be “embodied,” with implication for neuroscientists that are thinking about new solutions to be implemented into the current systems (Blanke et al., 2015; Riva, 2018; Riva et al., 2018). For example, the use of hands with contactless device (i.e., without gloves) makes the interaction in virtual environments more natural. The Leap Motion device^1^ allows one to use of hands in VR without the use of gloves or markers. This simple and low-cost device allows the VR users to interact with virtual objects and related environments in a naturalistic way. When technology is able to be transparent, users can experience increased sense of being in the virtual environments (the so-called sense of presence).

Other forms of interactions are possible and have been developing continuously. For example, tactile and haptic device able to provide a continuous feedback to the users, intensifying their experience also by adding components, such as the feeling of touch and the physical weight of virtual objects, by using force feedback. Another technology available at low cost that facilitates interaction is the motion tracking system, such as Microsoft Kinect, for example. Such technology allows one to track the users’ bodies, allowing them to interact with the virtual environments using body movements, gestures, and interactions. Most HMDs use an embedded system to track HMD position and rotation as well as controllers that are generally placed into the user’s hands. This tracking allows a great degree of interaction and improves the overall virtual experience.

A final emerging approach is the use of digital technologies to simulate not only the external world but also the internal bodily signals (Azevedo et al., 2017; Riva et al., 2017): interoception, proprioception and vestibular input. For example, Riva et al. (2017) recently introduced the concept of “sonoception” (www.sonoception.com), a novel non-invasive technological paradigm based on wearable acoustic and vibrotactile transducers able to alter internal bodily signals. This approach allowed the development of an interoceptive stimulator that is both able to assess interoceptive time perception in clinical patients (Di Lernia et al., 2018b) and to enhance heart rate variability (the short-term vagally mediated component—rMSSD) through the modulation of the subjects’ parasympathetic system (Di Lernia et al., 2018a).

In this scenario, it is clear that the future of VR and AR research is not just in clinical applications, although the implications for the patients are huge. The continuous development of VR and AR technologies is the result of research in computer science, engineering, and allied sciences. The reasons for which from our analyses emerged a “clinical era” are threefold. First, all clinical research on VR and AR includes also technological developments, and new technological discoveries are being published in clinical or technological journals but with clinical samples as main subject. As noted in our research, main journals that publish numerous articles on technological developments tested with both healthy and patients include *Presence: Teleoperators & Virtual Environments, Cyberpsychology & Behavior* (Cyberpsychol BEHAV), and *IEEE Computer Graphics and Applications* (IEEE Comput Graph). It is clear that researchers in psychology, neuroscience, medicine, and behavioral sciences in general have been investigating whether the technological developments of VR and AR are effective for users, indicating that clinical behavioral research has been incorporating large parts of computer science and engineering. A second aspect to consider is the industrial development. In fact, once a new technology is envisioned and created it goes for a patent application. Once the patent is sent for registration the new technology may be made available for the market, and eventually for journal submission and publication. Moreover, most VR and AR research that that proposes the development of a technology moves directly from the presenting prototype to receiving the patent and introducing it to the market without publishing the findings in scientific paper. Hence, it is clear that if a new technology has been developed for industrial market or consumer, but not for clinical purpose, the research conducted to develop such technology may never be published in a scientific paper. Although our manuscript considered published researches, we have to acknowledge the existence of several researches that have not been published at all. The third reason for which our analyses highlighted a “clinical era” is that several articles on VR and AR have been considered within the Web of Knowledge database, that is our source of references. In this article, we referred to “research” as the one in the database considered. Of course, this is a limitation of our study, since there are several other databases that are of big value in the scientific community, such as IEEE Xplore Digital Library, ACM Digital Library, and many others. Generally, the most important articles in journals published in these databases are also included in the Web of Knowledge database; hence, we are convinced that our study considered the top-level publications in computer science or engineering. Accordingly, we believe that this limitation can be overcome by considering the large number of articles referenced in our research.

Considering all these aspects, it is clear that clinical applications, behavioral aspects, and technological developments in VR and AR research are parts of a more complex situation compared to the old platforms used before the huge diffusion of HMD and solutions. We think that this work might provide a clearer vision for stakeholders, providing evidence of the current research frontiers and the challenges that are expected in the future, highlighting all the connections and implications of the research in several fields, such as clinical, behavioral, industrial, entertainment, educational, and many others.

## Author Contributions

PC and GR conceived the idea. PC made data extraction and the computational analyses and wrote the first draft of the article. IG revised the introduction adding important information for the article. PC, IG, MR, and GR revised the article and approved the last version of the article after important input to the article rationale.

## Conflict of Interest Statement

The authors declare that the research was conducted in the absence of any commercial or financial relationships that could be construed as a potential conflict of interest. The reviewer GC declared a shared affiliation, with no collaboration, with the authors PC and GR to the handling Editor at the time of the review.

## Footnotes

1 https://www.leapmotion.com/
