# Supplementary material for: The Past, Present, and Future of Virtual and Augmented Reality Research: A Network and Cluster Analysis of the Literature
Source: Front Psychol. 2018 Nov 6;9:2086. doi: 10.3389/fpsyg.2018.02086 (PMC6232426; doi:10.3389/fpsyg.2018.02086)
Supplement: Supplementary file 1 [file Data_Sheet_1.ZIP › Cluster_Country.docx]

| **ClusterID** | **Size** | **Silhouette** | **mean(Year)** | **Label (TFIDF)** | **Label (LLR)** | **Label (MI)** |
| --- | --- | --- | --- | --- | --- | --- |
| 0 | 22 | 0.655 | 1998 | (8.95) assembly; (8.85) cybersickness; (8.85) virtual assembly; (8.26) reality; (8.18) ter | cybersickness (12.06, 0.001); research (9.03, 0.005); qtvr (8.03, 0.005); | approach |
| 1 | 20 | 0.67 | 1999 | (13.56) assessment; (13.49) future; (12.99) autism; (11.78) teaching; (11.78) history | effect (29.38, 1.0E-4); assessment (26.09, 1.0E-4); use (21.96, 1.0E-4); | approach |
| 2 | 19 | 0.643 | 1999 | (8.92) reality; (8.85) intelligent tutoring system; (8.59) virtual reality; (7.54) environment; (7.41) ter | intelligent tutoring system (12.42, 0.001); virtual reality application (9.43, 0.005); human locomotion (8.27, 0.005); | collaborative virtual environment |
| 3 | 18 | 0.618 | 1998 | (9.65) reality; (9.61) virtual reality; (9.19) plan; (9.09) response; (8.93) planning | planning (15.67, 1.0E-4); virtual workbench (11.75, 0.001); polish isaf soldier (11.75, 0.001); | dentition |
| 4 | 12 | 0.506 | 2002 | (7.99) reality; (7.88) virtual reality; (7.54) ter; (7.14) helmet; (7.12) com | helmet (8.95, 0.005); virtual design (4.47, 0.05); understanding (4.47, 0.05); | collaborative virtual environment |
| 5 | 12 | 0.665 | 1997 | (7.54) reality; (7.54) virtual reality; (7.14) society; (7.14) film; (7.12) ter | virtual heart model (5.18, 0.05); community (5.18, 0.05); head-related transfer function (5.18, 0.05); | dentition |
| 6 | 7 | 0.986 | 1997 | (8.85) natural user interface; (8.85) nui; (8.85) interaction technique; (8.85) cad system; (6.6) ter | natural user interface (20.9, 1.0E-4); video (20.9, 1.0E-4); nui (20.9, 1.0E-4); | 3d virtual reality system |
