# Supplementary material for: The Past, Present, and Future of Virtual and Augmented Reality Research: A Network and Cluster Analysis of the Literature
Source: Front Psychol. 2018 Nov 6;9:2086. doi: 10.3389/fpsyg.2018.02086 (PMC6232426; doi:10.3389/fpsyg.2018.02086)
Supplement: Supplementary file 1 [file Data_Sheet_1.ZIP › Cluster_Institutions.docx]

| **ClusterID** | **Size** | **Silhouette** | **mean(Year)** | **Label (TFIDF)** | **Label (LLR)** | **Label (MI)** |
| --- | --- | --- | --- | --- | --- | --- |
| 0 | 14 | 0.904 | 2006 | (15.35) ter; (15.35) virtual reality; (14.64) metric; (13.99) skill; (13.32) therapy | html5 (8.67, 0.005); using unpredictable trajectories (8.67, 0.005); laparoscopic skill (8.67, 0.005); | surgery |
| 1 | 14 | 0.876 | 2000 | (17.54) virtual reality; (14.64) assembly planning; (14.56) modeling; (14.31) ter; (13.18) planning | building (9.06, 0.005); assembly planning (9.06, 0.005); cerebral palsy (9.06, 0.005); | training system |
| 2 | 13 | 0.959 | 2007 | (16.39) shape; (16.38) size; (16.2) virtual reality; (14.64) fear; (14.01) influence | shape (27.59, 1.0E-4); size (18.34, 1.0E-4); fear (13.73, 0.001); | environment |
| 3 | 12 | 0.811 | 2003 | (16.57) virtual reality; (14.43) virtual environment; (14.31) environment; (13.61) surgery; (13.18) ergonomic | locomotion (8.81, 0.005); ergonomic (8.81, 0.005); efficacy (8.81, 0.005); | virtual-reality environment |
| 4 | 11 | 0.913 | 2005 | (14.98) people; (14.86) virtual reality; (14.64) photogrammetry; (13.69) ter; (13.69) system | people (19.02, 1.0E-4); ancient ayutthaya (9.48, 0.005); thailand (9.48, 0.005); | training system |
| 5 | 11 | 0.707 | 2003 | (17.54) ter; (15.35) virtual reality; (15.32) self; (13.32) perception; (12.34) interface | body ownership (9.48, 0.005); self (9.48, 0.005); brain-computer interface (5.86, 0.05); | virtual-reality environment |
| 6 | 10 | 0.945 | 1996 | (17.54) virtual reality; (12.13) ter; (11.81) surgical education; (11.71) virtual-reality; (11.09) system | surgical education (11.28, 0.001); virtual reality (7.98, 0.005); what (7.58, 0.01); | special section |
| 7 | 9 | 1 | 2006 | (19.02) virtual reality; (18.2) induction; (14.98) controlled trial; (14.98) mood; (14.95) treatment | balance rehabilitation (12.49, 0.001); patient (11.94, 0.001); pilot study (9.57, 0.005); | difference |
| 8 | 7 | 0.961 | 2002 | (14.98) virtual reality exposure; (13.69) ter; (13.69) virtual reality; (12.78) exposure; (11.96) skill | virtual reality exposure therapy (11.23, 0.001); ptsd vietnam veteran (11.23, 0.001); anxiety disorder (11.23, 0.001); | virtual basic laparoscopic skill trainer |
| 9 | 5 | 1 | 2001 | (12.98) virtual reality; (11.81) travel anxiety; (11.71) technology; (10.52) stroke; (7.87) environment | travel anxiety (6.86, 0.01); 3d synthetic environment construction (6.86, 0.01); using color database (6.86, 0.01); | using modern information technology |
