# Supplementary material for: The Past, Present, and Future of Virtual and Augmented Reality Research: A Network and Cluster Analysis of the Literature
Source: Front Psychol. 2018 Nov 6;9:2086. doi: 10.3389/fpsyg.2018.02086 (PMC6232426; doi:10.3389/fpsyg.2018.02086)
Supplement: Supplementary file 1 [file Data_Sheet_1.ZIP › Cluster_journals.docx]

| **ClusterID** | **Size** | **Silhouette** | **mean(Year)** | **Label (TFIDF)** | **Label (LLR)** | **Label (MI)** |
| --- | --- | --- | --- | --- | --- | --- |
| 0 | 56 | 0.853 | 1996 | (21.76) collision detection; (17.8) reality; (17.77) assembly; (17.59) ter; (17.46) manufacturing | computer-vision-enabled augmented reality (36.52, 1.0E-4); collision detection (35.99, 1.0E-4); using virtual reality (34.82, 1.0E-4); | engineering development |
| 1 | 47 | 0.872 | 1999 | (17.48) reality; (17.17) virtual reality; (16.45) ter; (16.31) adaptation; (16.17) parkinsons disease | parkinsons disease (60.82, 1.0E-4); human spatial navigation (42.63, 1.0E-4); neural substrate (42.63, 1.0E-4); | fmri study |
| 2 | 40 | 0.896 | 1997 | (16.83) routine use; (16.83) current application; (16.83) behavioral-assessment; (16.83) obstacle; (16.83) future possibilities | future possibilities (44.71, 1.0E-4); routine use (44.71, 1.0E-4); current application (44.71, 1.0E-4); | comparative research |
| 3 | 39 | 0.726 | 1993 | (18.58) reality; (18.18) virtual reality; (16.9) environment; (16.8) virtual environment; (16.79) ter | effect (42, 1.0E-4); virtual reality (35.39, 1.0E-4); augmented reality (34.62, 1.0E-4); | behavioural training |
| 4 | 33 | 0.87 | 1993 | (13.89) reality; (12.97) virtual reality; (12.56) policy; (12.43) ter; (11.98) environment | virtual-reality (35.86, 1.0E-4); policy (23.88, 1.0E-4); net (23.88, 1.0E-4); | psychology |
| 5 | 33 | 0.89 | 2001 | (16.83) developing suturing technique; (15.5) virtual university; (15.5) tele-manipulation; (15.5) telesurgery; (15.5) tele-education | measuring (42.88, 1.0E-4); other wizardry (42.88, 1.0E-4); developing suturing technique (42.88, 1.0E-4); | comparative research |
| 6 | 14 | 0.983 | 1994 | (16.21) japanese institutional mechanism; (16.21) systems perspective; (9.94) perspective; (9.94) mechanism; (9.42) system | japanese institutional mechanism (72.69, 1.0E-4); systems perspective (72.69, 1.0E-4); customer interface (17.95, 1.0E-4); | virtual reality |
| 7 | 11 | 0.977 | 2007 | (18.07) stroke; (16.8) rehabilitation; (16.79) reality; (16.68) virtual reality; (16.1) patient | stroke (55.95, 1.0E-4); children (55.19, 1.0E-4); cerebral palsy (42.67, 1.0E-4); | rehabilitation gaming |
| 8 | 5 | 0.996 | 1992 | (9.42) reality; (9.07) virtual reality; (6.78) performance; (6.49) avatar; (6.46) body | chapter (8.2, 0.005); black avatar (8.2, 0.005); affective state (8.2, 0.005); | future effect |
