# Supplementary material for: The Past, Present, and Future of Virtual and Augmented Reality Research: A Network and Cluster Analysis of the Literature
Source: Front Psychol. 2018 Nov 6;9:2086. doi: 10.3389/fpsyg.2018.02086 (PMC6232426; doi:10.3389/fpsyg.2018.02086)
Supplement: Supplementary file 1 [file Data_Sheet_1.ZIP › Cluster_Keywords.docx]

| **ClusterID** | **Size** | **Silhouette** | **mean(Year)** | **Label (TFIDF)** | **Label (LLR)** | **Label (MI)** |
| --- | --- | --- | --- | --- | --- | --- |
| 0 | 59 | 0.503 | 1996 | (11.01) visual illusion; (10.07) virtual environment use; (10.07) spatial knowledge; (10.07) physical ergonomic; (10.07) long-term retention | virtual environment (33.45, 1.0E-4); visual illusion (23.57, 1.0E-4); 3-d display (23.57, 1.0E-4); | computer-enhanced therapy |
| 1 | 47 | 0.659 | 1995 | (11.98) balance; (11.78) poster; (11.3) skill; (11.29) game; (11.14) neglect | research (41.42, 1.0E-4); virtual reality (31.77, 1.0E-4); design (27.89, 1.0E-4); | counsellor |
| 2 | 44 | 0.744 | 2000 | (15.76) skill; (15.04) technical skill; (14.7) instructor feedback; (14.24) laparoscopic skill; (13.56) acquisition | training (40.9, 1.0E-4); laparoscopic skill (40.01, 1.0E-4); instructor feedback (40.01, 1.0E-4); | information technology |
| 3 | 39 | 0.722 | 1994 | (10.07) retrieval; (9.71) telepresence; (8.85) critical analysis; (8.85) virtual library; (8.32) open technology | deepmatrix (21.51, 1.0E-4); virtual environment system (21.51, 1.0E-4); open technology (21.51, 1.0E-4); | geovr |
| 4 | 35 | 0.769 | 2008 | (14.6) balance; (12.92) balance training; (12.68) exposure therapy; (12.68) virtual reality exposure; (11.98) anxiety | postural control (37, 1.0E-4); multiple sclerosis patient (37, 1.0E-4); telerehabilitation program (37, 1.0E-4); | interactive computer play |
| 5 | 29 | 0.845 | 1993 | (8.85) electronic hardware; (8.85) ancient ayutthaya; (8.85) extensive computer support; (8.85) thailand; (8.85) digital building | building design (17.74, 1.0E-4); electronic hardware (17.74, 1.0E-4); ancient ayutthaya (17.74, 1.0E-4); | geovr |
| 6 | 20 | 0.969 | 1992 | (10.07) noncommand user interface; (8.85) current application; (8.85) behavioral-assessment; (8.85) routine use; (8.85) future possibilities | noncommand user interface (25.33, 1.0E-4); freezing (18.98, 1.0E-4); future possibilities (18.98, 1.0E-4); | virtual reality test |
| 7 | 19 | 0.858 | 1994 | (8.85) 3d graphical interface; (8.85) binary relational database; (8.85) triplespace; (7.14) japanese institutional mechanism; (7.14) systems perspective | triplespace (21.96, 1.0E-4); 3d graphical interface (21.96, 1.0E-4); binary relational database (21.96, 1.0E-4); | housebuilding industry |
| 8 | 10 | 0.928 | 1996 | (8.85) real doctor; (7.17) pain; (7.14) pain control; (6.37) dressing change; (6.37) judgment | judgment (19.71, 1.0E-4); real doctor (19.71, 1.0E-4); dressing change (19.71, 1.0E-4); | virtual reality test |
| 9 | 8 | 0.997 | 1992 | (12.43) virtual reality scotoma; (11.78) oculomotor adaptation; (7.5) adaptation; (5.06) motor; (4.03) reality | virtual reality scotoma (74.51, 1.0E-4); oculomotor adaptation (63.59, 1.0E-4); developing visual system (10.39, 0.005); | virtual reality |
| 10 | 5 | 1 | 1995 | (7.14) virtual-reality technology; (7.14) dedicated system; (5.9) cognitive deficit; (4.63) adult; (4.12) role | cognitive deficit (18.7, 1.0E-4); dedicated system (18.7, 1.0E-4); virtual-reality technology (18.7, 1.0E-4); | adolescence |
