# Supplementary material for: The Past, Present, and Future of Virtual and Augmented Reality Research: A Network and Cluster Analysis of the Literature
Source: Front Psychol. 2018 Nov 6;9:2086. doi: 10.3389/fpsyg.2018.02086 (PMC6232426; doi:10.3389/fpsyg.2018.02086)
Supplement: Supplementary file 1 [file Data_Sheet_1.ZIP › Clusters from AR.docx]

| 19 | 5.94 | 0.00 | 1.00 | 0.00 |  | Wang XY | 2008 | ***...*** | AUTOMAT CONSTR | V17 | P399 | 5 | 5 |
| --- | --- | --- | --- | --- | --- | --- | --- | --- | --- | --- | --- | --- | --- |
| 1 |  | 0.00 | 1.00 | 0.00 |  | Brooks FP | 1988 | ***...*** | CHI88 C P HUMAN FACT | V | P1 | 5 | 6 |
| 5 |  | 0.00 | 1.00 | 0.00 |  | Ploder O | 1995 | ***...*** | RADIOLOGE | V35 | P569 | 2 | 17 |
| 14 | 6.37 | 0.00 | 1.00 | 0.00 |  | Ohta Y | 1999 | ***...*** | MIXED REALITY MERGIN | V | P | 1 | 2 |
| 20 | 5.36 | 0.00 | 1.00 | 0.00 |  | El Sayed NAM | 2011 | ***...*** | COMPUT EDUC | V56 | P1045 | 3 | 4 |
| 2 |  | 0.00 | 1.00 | 0.00 |  | Drascic D | 1993 | ***...*** | Scientific Computing & Automation | V9 | P31 | 4 | 2 |
| 11 | 5.22 | 0.00 | 1.00 | 0.00 |  | Burdea GC | 2003 | ***...*** | VIRTUAL REALITY TECH | V | P | 7 | 5 |
| 4 |  | 0.00 | 1.00 | 0.00 |  | Ajune W | 2008 | ***...*** | ADV COMPUTER GRAPHIC | V | P87 | 1 | 9 |
| 38 | 10.53 | 0.00 | 1.00 | 0.00 |  | Klein G | 2009 | ***...*** | INT SYM MIX AUGMENT | V | P83 | 2 | 1 |
| 18 | 6.70 | 0.00 | 1.00 | 0.00 |  | Chai L | 2002 | ***...*** | PRESENCE-TELEOP VIRT | V11 | P474 | 2 | 0 |
| 40 | 12.51 | 0.00 | 1.00 | 0.00 |  | Lepetit V | 2006 | ***...*** | IEEE T PATTERN ANAL | V28 | P1465 | 2 | 1 |
| 9 | 3.92 | 0.00 | 1.00 | 0.00 |  | Cheok AD | 2003 | ***...*** | P 2 WORKSH NETW SYST | V | P106 | 7 | 5 |
| 1 |  | 0.00 | 1.00 | 0.00 |  | *im GUID TECHN INC | 1997 | ***...*** | FLASHP MOD 5000 3D L | V | P | 1 | 2 |

**References of clustering**

1. 0.07 Azuma,, R (2001) [recent advances in augmented reality](http://dx.doi.org/10.1109/38.963459)
2. 0.07 Azuma,, R (1999) [tracking in unprepared environments for augmented reality systems](http://dx.doi.org/10.1016/S0097-8493(99)00104-1)
3. 0.06 Neumann,, U (1999) augmented reality tracking in natural environments
4. 0.06 Zhu,, P (2002) an internet oriented platform for civil engineering applications: towards disaster mitigation in metropolises
5. 0.05 Azuma,, RT (1999) the challenge of making augmented reality work outdoors
6. 0.05 Klinker,, G (1999) augmented reality: a balancing act between high quality and real-time constraints
7. 0.05 Yokoya,, N (1999) passive range sensing techniques: depth from images
8. 0.05 Yokoya,, N (1999) stereo vision based video see-through mixed reality
9. 0.05 You,, SY (1999) [orientation tracking for outdoor augmented reality registration](http://dx.doi.org/10.1109/38.799738)
10. 0.04 Azuma,, R (1999) [a motion-stabilized outdoor augmented reality system](http://dx.doi.org/10.1109/VR.1999.756959)
11. 0.04 Feiner,, S (1999) wearing it out: first steps toward mobile augmented reality systems
12. 0.04 Fuhrmann,, A (1999) [occlusion in collaborative augmented environments](http://dx.doi.org/10.1016/S0097-8493(99)00107-7)
13. 0.04 Stricker,, D (1999) a fast and robust line-based optical tracker for augmented reality applications
14. 0.03 Azuma,, RT (1999) making augmented reality work outdoors requires hybrid tracking
15. 0.03 Kanbara,, M (1999) real-time composition of stereo images for video see-through augmented reality
16. 0.03 Pasman,, W (1999) [accurate overlaying for mobile augmented reality](http://dx.doi.org/10.1016/S0097-8493(99)00118-1)
17. 0.03 Poupyrev,, I (2001) tiles: a mixed reality authoring interface
18. 0.03 You,, S (1999) [hybrid inertial and vision tracking for augmented reality registration](http://dx.doi.org/10.1109/VR.1999.756960)
19. 0.02 Ansar,, A (2001) [visual and haptic collaborative tele-presence](http://dx.doi.org/10.1016/S0097-8493(01)00121-2)
20. 0.02 Behringer,, R (1999) a novel interface for device diagnostics using speech recognition, augmented reality visualization, and 3d audio auralization
21. 0.02 Behringer,, R (2002) [model-based visual tracking for outdoor augmented reality applications](http://dx.doi.org/10.1109/ISMAR.2002.1115111)
22. 0.02 Billinghurst,, M (1999) collaborative mixed reality
23. 0.02 Chung,, KH (1999) [an application of augmented reality to thickness inspection](http://dx.doi.org/10.1002/(SICI)1520-6564(199923)9:4%3c331::AID-HFM1%3e3.0.CO;2-3)
24. 0.02 Dorfmuller,, K (1999) [robust tracking for augmented reality using retroreflective markers](http://dx.doi.org/10.1016/S0097-8493(99)00105-3)
25. 0.02 Gemeiner,, P (2007) [simultaneous motion and structure estimation by fusion of inertial and vision data](http://dx.doi.org/10.1177/0278364907080058)
26. 0.02 Hoff,, WA (1999) fusion of data from head-mounted and fixed sensors
27. 0.02 Luo,, B (2011) [sensor fusion based head pose tracking for lightweight flight cockpit systems](http://dx.doi.org/10.1007/s11042-010-0468-4)
28. 0.02 Neumann,, U (1999) [natural feature tracking for augmented reality](http://dx.doi.org/10.1109/6046.748171)
29. 0.02 Park,, H (2010) [invisible marker-based augmented reality](http://dx.doi.org/10.1080/10447318.2010.496335)
30. 0.02 Park,, J (1999) natural feature tracking for extendible robust augmented realities
31. 0.02 Raskar,, R (1999) spatially augmented reality
32. 0.02 Reiners,, D (1999) augmented reality for construction tasks: doorlock assembly
33. 0.02 Sato,, I (1999) [acquiring a radiance distribution to superimpose virtual objects onto a real scene](http://dx.doi.org/10.1109/2945.764865)
34. 0.02 Sundareswaran,, V (1999) visual servoing-based augmented reality
35. 0.02 Tamura,, H (1999) steps toward seamless mixed reality
36. 0.02 Xu,, K (2002) [visual registration for geographical labeling in wearable computing](http://dx.doi.org/10.1109/ISWC.2002.1167225)
37. 0.02 Yu,, DG (2010) [a useful visualization technique: a literature review for augmented reality and its application, limitation & future direction](http://dx.doi.org/10.1007/978-1-4419-0312-9_21)
38. 0.02 Zhang,, J (2010) [a multi-regional computation scheme in an ar-assisted in situ cnc simulation environment](http://dx.doi.org/10.1016/j.cad.2010.06.007)
39. 0.02 Anderson,, EF (2010) [developing serious games for cultural heritage: a state-of-the-art review](http://dx.doi.org/10.1007/s10055-010-0177-3)
40. 0.02 Auer,, T (1999) [the integration of optical and magnetic tracking for multi-user augmented reality](http://dx.doi.org/10.1016/S0097-8493(99)00106-5)
41. 0.02 Baber,, C (1999) [contrasting paradigms for the development of wearable computers](http://dx.doi.org/10.1147/sj.384.0551)
42. 0.02 Behringer,, R (1999) [registration for an augmented reality system enhancing the situational awareness in an outdoor scenario](http://dx.doi.org/10.1117/12.354425)
43. 0.02 Behringer,, R (1999) [registration for outdoor augmented reality applications using computer vision techniques and hybrid sensors](http://dx.doi.org/10.1109/VR.1999.756958)
44. 0.02 Behringer,, R (2000) [a wearable augmented reality testbed for navigation and control, built solely with commercial-off-the-shelf (cots) hardware](http://dx.doi.org/10.1109/ISAR.2000.880918)
45. 0.02 Behringer,, R (2000) [two wearable testbeds for augmented reality: itwarns and wimmis](http://dx.doi.org/10.1109/ISWC.2000.888495)
46. 0.02 Billinghurst,, M (2002) collaborative augmented reality
47. 0.02 Bimber,, O (2002) [merging fossil specimens with computer-generated information](http://dx.doi.org/10.1109/MC.2002.1033024)
48. 0.02 Brantner,, S (1999) real-time optical edge and corner tracking at subpixel accuracy
49. 0.02 Corke,, P (2007) [an introduction to inertial and visual sensing](http://dx.doi.org/10.1177/0278364907079279)
50. 0.02 Dubois,, E (1999) classification space for augmented surgery, an augmented reality case study
51. 0.02 Fjeld,, M (2001) design and evaluation of four ar navigation tools using scene and viewpoint handling
52. 0.02 Fuchs,, H (1999) displays for augmented reality: historical remarks and future prospects
53. 0.02 Gemeiner,, P (2007) [real-time slam with a high-speed cmos camera](http://dx.doi.org/10.1109/ICIAP.2007.4362795)
54. 0.02 Guan,, T (2010) [recovering pose and occlusion consistencies in augmented reality systems using affine properties](http://dx.doi.org/10.1108/02602281011022751)
55. 0.02 Hoff,, B (2000) [autocalibration of an electronic compass in an outdoor augmented reality system](http://dx.doi.org/10.1109/ISAR.2000.880939)
56. 0.02 Hollerer,, T (1999) [exploring mars: developing indoor and outdoor user interfaces to a mobile augmented reality system](http://dx.doi.org/10.1016/S0097-8493(99)00103-X)
57. 0.02 Hollerer,, T (2001) [user interface management techniques for collaborative mobile augmented reality](http://dx.doi.org/10.1016/S0097-8493(01)00122-4)
58. 0.02 Iwata,, H (1999) feel-through: augmented reality with force feedback
59. 0.02 Jang,, B (1999) an outdoor augmented reality system for gis applications
60. 0.02 Kato,, H (2000) [virtual object manipulation on a table-top ar environment](http://dx.doi.org/10.1109/ISAR.2000.880934)
61. 0.02 Kim,, S (2010) [ar interfacing with prototype 3d applications based on user-centered interactivity](http://dx.doi.org/10.1016/j.cad.2008.10.009)
62. 0.02 Kiyokawa,, K (1999) seamlessdesign: a face-to-face collaborative virtual/augmented environment for rapid prototyping of geometrically constrained 3-d objects
63. 0.02 Lehikoinen,, J (2001) an evaluation of augmented reality navigational maps in head-worn displays
64. 0.02 Lu,, CY (1999) virtual and augmented reality technologies for product realization
65. 0.02 Maidi,, M (2010) [handling occlusions for robust augmented reality systems](http://dx.doi.org/10.1155/2010/146123)
66. 0.02 Marchand,, T (2002) virtual visual servoing: a framework for real-time augmented reality
67. 0.02 Martins,, JF (2010) a facade tracking system for outdoor augmented reality
68. 0.02 Milgram,, P (1999) a taxonomy of real and virtual world display integration
69. 0.02 Navab,, N (2003) [industrial augmented reality(iar): challenges in design and commercialization of killer apps](http://dx.doi.org/10.1109/ISMAR.2003.1240682)
70. 0.02 Park,, H (2010) [automatic confidence adjustment of visual cues in model-based camera tracking](http://dx.doi.org/10.1002/cav.321)
71. 0.02 Pressigout,, M (2004) [model-free augmented reality by virtual visual servoing](http://dx.doi.org/10.1109/ICPR.2004.1334401)
72. 0.02 Regenbrecht,, HT (2000) [a mobile passive augmented reality device - mpard](http://dx.doi.org/10.1109/ISAR.2000.880926)
73. 0.02 Reitmayr,, G (2001) [mobile collaborative augmented reality](http://dx.doi.org/10.1109/ISAR.2001.970521)
74. 0.02 Satoh,, K (1999) case studies of see-through augmentation in mixed reality project
75. 0.02 Schmalstieg,, D (2000) [bridging multiple user interface dimensions with augmented reality](http://dx.doi.org/10.1109/ISAR.2000.880919)
76. 0.02 Simon,, G (2000) [markerless tracking using planar structures in the scene](http://dx.doi.org/10.1109/ISAR.2000.880935)
77. 0.02 State,, A (2001) [dynamic virtual convergence for video see-through head-mounted displays: maintaining maximum stereo overlap throughout a close-range work space](http://dx.doi.org/10.1109/ISAR.2001.970523)
78. 0.02 Turunen,, T (2000) [a wearable computer for mobile augmented reality based controlling of an intelligent robot](http://dx.doi.org/10.1117/12.403786)
79. 0.02 Uchiyama,, S (2002) [mr platform: a basic body on which mixed reality applications are built](http://dx.doi.org/10.1109/ISMAR.2002.1115095)
80. 0.02 Vallino,, J (1999) haptics in augmented reality
81. 0.02 Wientapper,, F (2011) [composing the feature map retrieval process for robust and ready-to-use monocular tracking](http://dx.doi.org/10.1016/j.cag.2011.04.008)
82. 0.01 Ababsa,, F (2007) robust circular fiducials tracking and camera pose estimation using particle filtering
83. 0.01 Ababsa,, F (2010) real-time camera tracking for structured environment using an iterated particle filter
84. 0.01 Ababsa,, FE (2007) [hybrid three-dimensional camera pose estimation using particle filter sensor fusion](http://dx.doi.org/10.1163/156855307779293689)
85. 0.01 Akhloufi,, MA (1999) virtual view synthesis from uncalibrated stereo cameras
86. 0.01 Alaghehbandian,, A (2003) an internet oriented platform for structural health monitoring
87. 0.01 Alaghehbandian,, A (2003) [developing an internet oriented platform for earthquake engineering application and web-based simulation system for seismic hazards: towards disaster mitigation in metropolises](http://dx.doi.org/10.1109/IV.2003.1217982)
88. 0.01 Alaghehbandian,, A (2003) developing an internet oriented platform for earthquake engineering application and web-based virtual reality simulation system for seismic hazards: towards disaster mitigation in metropolises
89. 0.01 Ansar,, A (2001) linear solutions for visual augmented reality registration
90. 0.01 Azuma,, RT (1997) [a survey of augmented reality](http://dx.doi.org/10.1162/pres.1997.6.4.355)
91. 0.01 Baillot,, Y (2003) [a tracker alignment framework for augmented reality](http://dx.doi.org/10.1109/ISMAR.2003.1240697)
92. 0.01 Balcisoy,, S (2000) [augmented reality for real and virtual humans](http://dx.doi.org/10.1109/CGI.2000.852346)
93. 0.01 Baratoff,, G (2002) [interactive multi-marker calibration for augmented reality applications](http://dx.doi.org/10.1109/ISMAR.2002.1115079)
94. 0.01 Bayerl,, PAJ (2002) an interactive vision-based tool for model-based scene calibration of augmented reality environments
95. 0.01 Behringer,, R (1999) [a distributed device diagnostics system utilizing augmented reality and 3d audio](http://dx.doi.org/10.1016/S0097-8493(99)00108-9)
96. 0.01 Behringer,, R (1999) improving registration precision through visual horizon silhouette matching
97. 0.01 Behringer,, R (1999) international workshop on augmented reality 1998 - overview and summary
98. 0.01 Berger,, MO (1999) [mixing synthetic and video images of an outdoor urban environment](http://dx.doi.org/10.1007/s001380050098)
99. 0.01 Bimber,, O (2001) [the extended virtual table: an optical extension for table-like projection systems](http://dx.doi.org/10.1162/105474601753272862)
100. 0.01 Brogni,, A (1999) [technological approach for cultural heritage: augmented reality](http://dx.doi.org/10.1109/ROMAN.1999.900341)
101. 0.01 Caruso,, G (2010) [interactive augmented reality system for product design review](http://dx.doi.org/10.1117/12.840261)
102. 0.01 Cheng,, YM (2001) the reality gap: pragmatic boundaries of context awareness
103. 0.01 Cheok,, AD (2002) [touch-space: mixed reality game space based on ubiquitous, tangible, and social computing](http://dx.doi.org/10.1007/s007790200047)
104. 0.01 Chinthammit,, W (2002) [unique shared-aperture display with head or target tracking](http://dx.doi.org/10.1109/VR.2002.996527)
105. 0.01 Cho,, Y (1999) a multi-ring fiducial system and an intensity-invariant detection method for scalable augmented reality
106. 0.01 Chroust,, SG (2003) fusion of vision and inertia date for motion and structure estimation
107. 0.01 Dorfmuller-Ulhaas,, K (2001) finger tracking for interaction in augmented environments
108. 0.01 Dornaika,, F (2007) real-time vehicle ego-motion using stereo pairs and particle filters
109. 0.01 Duchesne,, C (2000) a point-based approach to the interposition problem in augmented reality
110. 0.01 Duchesne,, C (2000) comparison of matching criteria for the interposition problem in augmented reality
111. 0.01 Dutoit,, AH (2001) [architectural issues in mobile augmented reality systems: a prototyping case study](http://dx.doi.org/10.1109/APSEC.2001.991499)
112. 0.01 Feiner,, SK (2002) [augmented reality: a new way of seeing](http://dx.doi.org/10.1038/scientificamerican0402-48)
113. 0.01 Feng,, Y (2011) [some research on the fusion of virtual scene and video image](http://dx.doi.org/10.4028/www.scientific.net/AMM.58-60.1)
114. 0.01 Fuhrmann,, AL (2001) comprehensive calibration and registration procedures for augmented reality
115. 0.01 Furmanski,, C (2002) [augmented-reality visualizations guided by cognition: perceptual heuristics for combining visible and obscured information](http://dx.doi.org/10.1109/ISMAR.2002.1115091)
116. 0.01 Gabbard,, JL (2002) [usability engineering: domain analysis activities for augmented reality systems](http://dx.doi.org/10.1117/12.468073)
117. 0.01 Gee,, AP (2011) [a topometric system for wide area augmented reality](http://dx.doi.org/10.1016/j.cag.2011.04.006)
118. 0.01 Genc,, Y (2000) [optical see-through hmd calibration: a stereo method validated with a video see-through system](http://dx.doi.org/10.1109/ISAR.2000.880940)
119. 0.01 Genc,, Y (2001) [optical see-through calibration with vision-based trackers: propagation of projection matrices](http://dx.doi.org/10.1109/ISAR.2001.970524)
120. 0.01 Gonzalez-Garcia,, G (2010) optimisation of focal length using a stereoscopic operating microscope for augmented reality surgical guidance
121. 0.01 Goose,, S (2003) [speech-enabled augmented reality supporting mobile industrial maintenance](http://dx.doi.org/10.1109/MPRV.2003.1186727)
122. 0.01 Gordon,, G (2002) [the use of dense stereo range data in augmented reality](http://dx.doi.org/10.1109/ISMAR.2002.1115063)
123. 0.01 Guttentag,, DA (2010) [virtual reality: applications and implications for tourism](http://dx.doi.org/10.1016/j.tourman.2009.07.003)
124. 0.01 Harasaki,, S (2001) [vision based overlay of a virtual object into real scene for designing room interior](http://dx.doi.org/10.1117/12.444225)
125. 0.01 Hedley,, NR (2002) [explorations in the use of augmented reality for geographic visualization](http://dx.doi.org/10.1162/1054746021470577)
126. 0.01 Herve,, JY (2000) dynamic registration for augmented reality in telerobotics applications
127. 0.01 Hutson,, M (2011) [janusvf: accurate navigation using scaat and virtual fiducials](http://dx.doi.org/10.1109/TVCG.2010.91)
128. 0.01 Inami,, M (2001) ["talking head" system using object-oriented display](http://dx.doi.org/10.1117/12.430846)
129. 0.01 Ioannidis,, N (2002) [hybrid image processing techniques for the reconstruction of lost worlds and the revival of ancient life](http://dx.doi.org/10.1109/ICDSP.2002.1027827)
130. 0.01 Julier,, S (2000) [information filtering for mobile augmented reality](http://dx.doi.org/10.1109/ISAR.2000.880917)
131. 0.01 Julier,, S (2001) mobile augmented reality: a complex human-centered system
132. 0.01 Kanbara,, M (2000) a stereo vision-based augmented reality system with a wide range of registration
133. 0.01 Kanbara,, M (2000) [a stereo vision-based augmented reality system with an inertial sensor](http://dx.doi.org/10.1109/ISAR.2000.880931)
134. 0.01 Kanbara,, M (2001) [a stereo vision-based augmented reality system with marker and natural feature tracking](http://dx.doi.org/10.1109/VSMM.2001.969700)
135. 0.01 Keitler,, P (2010) [management of tracking for mixed and augmented reality systems](http://dx.doi.org/10.1007/978-1-84882-733-2_13)
136. 0.01 Kim,, H (1999) [a birds-eye view system using augmented reality](http://dx.doi.org/10.1109/SIMSYM.1999.766463)
137. 0.01 Kim,, J (2002) development of video-based interactive content system for internet
138. 0.01 Kim,, JS (2007) [a recursive camera resectioning technique for off-line video-based augmented reality](http://dx.doi.org/10.1016/j.patrec.2006.11.012)
139. 0.01 Kim,, T (2001) improving ar using shadows arising from natural illumination distribution in video sequences
140. 0.01 Kiyokawa,, K (2001) [an optical see-through display for mutual occlusion with a real-time stereovision system](http://dx.doi.org/10.1016/S0097-8493(01)00119-4)
141. 0.01 Klinker,, G (2000) [distributed user tracking concepts for augmented reality applications](http://dx.doi.org/10.1109/ISAR.2000.880921)
142. 0.01 Klinker,, G (2001) augmented maintenance of powerplants: a prototyping case study of a mobile ar system
143. 0.01 Konomi,, S (1999) passage: physical transportation of digital information in cooperative buildings
144. 0.01 Kourogi,, M (2000) [improvement of panorama-based annotation overlay using omnidirectional vision and inertial sensors](http://dx.doi.org/10.1109/ISWC.2000.888492)
145. 0.01 Kourogi,, M (2003) [personal positioning based on walking locomotion analysis with self-contained sensors and a wearable camera](http://dx.doi.org/10.1109/ISMAR.2003.1240693)
146. 0.01 Kuroda,, Y (2010) error reduction in 3d gaze point estimation for advanced medical annotations
147. 0.01 Lambrecht,, J (2012) spatial programming for industrial robots based on gestures and augmented reality
148. 0.01 Lawson,, SW (2002) [augmented reality as a tool to aid the telerobotic exploration and characterization of remote environments](http://dx.doi.org/10.1162/105474602760204273)
149. 0.01 Lee,, JW (1999) [rule-based segmentation for intensity-adaptive fiducial detection](http://dx.doi.org/10.1117/12.348425)
150. 0.01 Lee,, SL (2010) [from medical images to minimally invasive intervention: computer assistance for robotic surgery](http://dx.doi.org/10.1016/j.compmedimag.2009.07.007)
151. 0.01 Lepetit,, V (2000) a semi-automatic method for resolving occlusion in augmented reality
152. 0.01 Lepetit,, V (2000) [handling occlusion in augmented reality systems: a semi-automatic method](http://dx.doi.org/10.1109/ISAR.2000.880937)
153. 0.01 Lepetit,, V (2003) [fully automated and stable registration for augmented reality applications](http://dx.doi.org/10.1109/ISMAR.2003.1240692)
154. 0.01 Loscos,, C (2000) [interactive virtual relighting of real scenes](http://dx.doi.org/10.1109/2945.895874)
155. 0.01 Maidi,, M (2010) [a performance study for camera pose estimation using visual marker based tracking](http://dx.doi.org/10.1007/s00138-008-0170-y)
156. 0.01 Malik,, S (2002) [hand tracking for interactive pattern-based augmented reality](http://dx.doi.org/10.1109/ISMAR.2002.1115080)
157. 0.01 Malkawi,, A (2004) [interactive, immersive visualization for indoor environments: use of augmented reality, human-computer interaction and building simulation](http://dx.doi.org/10.1109/IV.2004.1320237)
158. 0.01 McDonald,, C (2002) hand-based interaction in augmented reality
159. 0.01 McGarrity,, E (2001) [a new system for online quantitative evaluation of optical see-through augmentation](http://dx.doi.org/10.1109/ISAR.2001.970525)
160. 0.01 Mendelsohn,, J (1999) constrained self-calibration for augmented reality registration
161. 0.01 Molineros,, J (1999) areas: augmented reality for evaluating assembly sequences
162. 0.01 Naimark,, L (2002) [circular data matrix fiducial system and robust image processing for a wearable vision-inertial self-tracker](http://dx.doi.org/10.1109/ISMAR.2002.1115065)
163. 0.01 Navab,, N (2010) [camera augmented mobile c-arm (camc): calibration, accuracy study, and clinical applications](http://dx.doi.org/10.1109/TMI.2009.2021947)
164. 0.01 Nilsson,, J (2010) [performance evaluation method for mobile computer vision systems using augmented reality](http://dx.doi.org/10.1109/VR.2010.5444821)
165. 0.01 Niquin,, C (2010) [a point cloud based pipeline for depth reconstruction from auto-stereoscopic sets](http://dx.doi.org/10.1117/12.838844)
166. 0.01 Nishi,, T (2001) snaplink: interactive object registration and recognition for augmented desk interface
167. 0.01 Okuma,, T (2001) real-time camera parameter estimation for 3-d annotation on a wearable vision system
168. 0.01 Ono,, Y (2010) [relative posture estimation using high frequency markers](http://dx.doi.org/10.1109/IROS.2010.5652134)
169. 0.01 Pasmore,, PJ (2001) [effects of viewing and orientation on path following in a medical teleoperation environment](http://dx.doi.org/10.1109/VR.2001.913788)
170. 0.01 Persa,, S (1999) on positioning for augmented reality systems
171. 0.01 Piekarski,, W (2001) [tinmith-metro: new outdoor techniques for creating city models with an augmented reality wearable computer](http://dx.doi.org/10.1109/ISWC.2001.962093)
172. 0.01 Piekarski,, W (2002) [bread crumbs: a technique for modelling large outdoor ground features](http://dx.doi.org/10.1109/ISMAR.2002.1115107)
173. 0.01 Pretlove,, J (1999) integrating augmented reality and telepresence for telerobotics in hostile environments
174. 0.01 Prince,, S (2002) [3d live: real time captured content for mixed reality](http://dx.doi.org/10.1109/ISMAR.2002.1115062)
175. 0.01 Prince,, SJD (2002) [augmented reality camera tracking with homographies](http://dx.doi.org/10.1109/MCG.2002.1046627)
176. 0.01 Pustka,, D (2011) [automatic configuration of pervasive sensor networks for augmented reality](http://dx.doi.org/10.1109/MPRV.2010.50)
177. 0.01 Regenbrecht,, H (2001) [a tangible ar desktop environment](http://dx.doi.org/10.1016/S0097-8493(01)00118-2)
178. 0.01 Ribo,, M (2001) a new optical tracking system for virtual and augmented reality applications
179. 0.01 Ribo,, M (2002) [hybrid tracking for outdoor augmented reality applications](http://dx.doi.org/10.1109/MCG.2002.1046629)
180. 0.01 Ribo,, M (2003) a flexible software architecture for hybrid hacking
181. 0.01 Rueckert,, D (2002) [automated camera calibration for image-guided surgery using intensity-based registration](http://dx.doi.org/10.1117/12.466951)
182. 0.01 Sargent,, D (2010) [endoscope-magnetic tracker calibration via trust region optimization](http://dx.doi.org/10.1117/12.843595)
183. 0.01 Shimoda,, M (2010) study of the visual support for nuclear power plant dismantlement work using augmented reality technology
184. 0.01 Simon,, G (2002) [pose estimation for planar structures](http://dx.doi.org/10.1109/MCG.2002.1046628)
185. 0.01 Soferman,, Z (1998) [advanced graphics behind medical virtual reality: evolution of algorithms, hardware, and software interfaces](http://dx.doi.org/10.1109/5.662878)
186. 0.01 Suomela,, R (2001) a system for evaluating augmented reality user interfaces in wearable computers
187. 0.01 Tarumi,, H (1999) spacetag: an overlaid virtual system and its applications
188. 0.01 Teber,, D (2010) [in-vitro evaluation of a soft-tissue navigation system for laparoscopic prostatectomy](http://dx.doi.org/10.1089/end.2009.0289)
189. 0.01 Tenmoku,, R (2003) [a wearable augmented reality system for navigation using positioning infrastructures and a pedometer](http://dx.doi.org/10.1109/ISMAR.2003.1240752)
190. 0.01 Tenmoku,, R (2003) [a wearable augmented reality system using positioning infrastructures and a pedometer](http://dx.doi.org/10.1109/ISWC.2003.1241400)
191. 0.01 Tenmoku,, R (2003) [a wearable augmented reality system using-an irda device and a passometer](http://dx.doi.org/10.1117/12.474072)
192. 0.01 Terashima,, M (1999) a human-robot interface using an extended digital desk
193. 0.01 Thomas,, B (2000) [arquake: an outdoor/indoor augmented reality first person application](http://dx.doi.org/10.1109/ISWC.2000.888480)
194. 0.01 Tian,, Y (2010) [real-time occlusion handling in augmented reality based on an object tracking approach](http://dx.doi.org/10.3390/s100402885)
195. 0.01 Tian,, YA (2010) [an automatic occlusion handling method in augmented reality](http://dx.doi.org/10.1108/02602281011051399)
196. 0.01 Torre,, R (2000) interaction between real and virtual humans: playing checkers
197. 0.01 Tuceryan,, M (2000) [single point active alignment method (spaam) for optical see-through hmd calibration for ar](http://dx.doi.org/10.1109/ISAR.2000.880938)
198. 0.01 Tuceryan,, M (2002) [single-point active alignment method (spaam) for optical see-through hmd calibration for augmented reality](http://dx.doi.org/10.1162/105474602317473213)
199. 0.01 Uva,, AE (2010) [distributed design review using tangible augmented technical drawings](http://dx.doi.org/10.1016/j.cad.2008.10.015)
200. 0.01 Vacchetti,, L (2003) [stable real-time interaction between virtual humans and real scenes](http://dx.doi.org/10.1109/IM.2003.1240281)
201. 0.11 Guan,, T (2010) [fast scene recognition and camera relocalisation for wide area augmented reality systems](http://dx.doi.org/10.3390/s100606017)
202. 0.11 Wagner,, D (2010) [real-time detection and tracking for augmented reality on mobile phones](http://dx.doi.org/10.1109/TVCG.2009.99)
203. 0.09 Yu,, DG (2010) [a useful visualization technique: a literature review for augmented reality and its application, limitation & future direction](http://dx.doi.org/10.1007/978-1-4419-0312-9_21)
204. 0.08 Barandiaran,, I (2010) [real-time optical markerless tracking for augmented reality applications](http://dx.doi.org/10.1007/s11554-009-0140-2)
205. 0.08 Wagner,, D (2010) [real-time panoramic mapping and tracking on mobile phones](http://dx.doi.org/10.1109/VR.2010.5444786)
206. 0.06 Guan,, T (2010) [recovering pose and occlusion consistencies in augmented reality systems using affine properties](http://dx.doi.org/10.1108/02602281011022751)
207. 0.06 Shen,, SH (2010) [monocular 3d tracking of deformable surfaces using sequential second order cone programming](http://dx.doi.org/10.1016/j.patcog.2009.06.016)
208. 0.06 Uchiyama,, H (2012) recent trends on visual tracking for augmented reality
209. 0.06 Wientapper,, F (2011) [composing the feature map retrieval process for robust and ready-to-use monocular tracking](http://dx.doi.org/10.1016/j.cag.2011.04.008)
210. 0.05 Audet,, S (2010) [direct image alignment of projector-camera systems with planar surfaces](http://dx.doi.org/10.1109/CVPR.2010.5540199)
211. 0.05 Castle,, RO (2010) [combining monoslam with object recognition for scene augmentation using a wearable camera](http://dx.doi.org/10.1016/j.imavis.2010.03.009)
212. 0.05 Kim,, K (2010) [scalable real-time planar targets tracking for digilog books](http://dx.doi.org/10.1007/s00371-010-0490-6)
213. 0.05 Klopschitz,, M (2010) visual tracking for augmented reality
214. 0.05 Li,, SQ (2011) [efficient lookup table based camera pose estimation for augmented reality](http://dx.doi.org/10.1002/cav.385)
215. 0.05 Murphy-Chutorian,, E (2010) [head pose estimation and augmented reality tracking: an integrated system and evaluation for monitoring driver awareness](http://dx.doi.org/10.1109/TITS.2010.2044241)
216. 0.05 Pilet,, J (2010) [virtually augmenting hundreds of real pictures: an approach based on learning, retrieval, and tracking](http://dx.doi.org/10.1109/VR.2010.5444811)
217. 0.03 Banwell,, T (2010) combining absolute positioning and vision for wide area augmented reality
218. 0.03 Billinghurst,, M (2011) the future of augmented reality in our everyday life
219. 0.03 Castle,, RO (2011) [keyframe-based recognition and localization during video-rate parallel tracking and mapping](http://dx.doi.org/10.1016/j.imavis.2011.05.002)
220. 0.03 Castle,, RO (2011) [wide-area augmented reality using camera tracking and mapping in multiple regions](http://dx.doi.org/10.1016/j.cviu.2011.02.007)
221. 0.03 Cho,, K (2010) multiple page recognition and tracking for augmented books
222. 0.03 El, Choubassi, M (2010) an augmented reality tourist guide on your mobile devices
223. 0.03 Fiala,, M (2010) [designing highly reliable fiducial markers](http://dx.doi.org/10.1109/TPAMI.2009.146)
224. 0.03 Kim,, YM (2010) marker detection by using affine-sift matching points for marker occlusion of augmented reality
225. 0.03 Martins,, JF (2010) a facade tracking system for outdoor augmented reality
226. 0.03 Pomares,, J (2010) [visual control of robots using range images](http://dx.doi.org/10.3390/s100807303)
227. 0.03 Sugimoto,, M (2010) [image overlay navigation by markerless surface registration in gastrointestinal, hepatobiliary and pancreatic surgery](http://dx.doi.org/10.1007/s00534-009-0199-y)
228. 0.03 Taketomi,, T (2011) [real-time and accurate extrinsic camera parameter estimation using feature landmark database for augmented reality](http://dx.doi.org/10.1016/j.cag.2011.04.007)
229. 0.03 Wither,, J (2011) [indirect augmented reality](http://dx.doi.org/10.1016/j.cag.2011.04.010)
230. 0.03 Yii,, W (2012) distributed visual processing for augmented reality
231. 0.03 Zhang,, J (2010) [a multi-regional computation scheme in an ar-assisted in situ cnc simulation environment](http://dx.doi.org/10.1016/j.cad.2010.06.007)
232. 0.02 Ababsa,, F (2010) real-time camera tracking for structured environment using an iterated particle filter
233. 0.02 Ajanki,, A (2011) [an augmented reality interface to contextual information](http://dx.doi.org/10.1007/s10055-010-0183-5)
234. 0.02 Al, Delail, B (2013) indoor localization and navigation using smartphones augmented reality and inertial tracking
235. 0.02 Aleotti,, J (2010) object manipulation in visuo-haptic augmented reality with physics-based animation
236. 0.02 Alvarez,, H (2011) providing guidance for maintenance operations using automatic markerless augmented reality system
237. 0.02 Aracena-Pizarro,, D (2010) museum guide through annotations using augmented reality
238. 0.02 Arth,, C (2011) real-time self-localization from panoramic images on mobile devices
239. 0.02 Arvanitis,, P (2012) augmented reality in second language teaching and learning ?
240. 0.02 Azuma,, R (2001) [recent advances in augmented reality](http://dx.doi.org/10.1109/38.963459)
241. 0.02 Bae,, H (2015) [image-based localization and content authoring in structure-from-motion point cloud models for real-time field reporting applications](http://dx.doi.org/10.1061/(ASCE)CP.1943-5487.0000392)
242. 0.02 Behr,, J (2011) instantreality - a framework for industrial augmented and virtual reality applications
243. 0.02 Belhaoua,, A (2014) accuracy analysis of an augmented reality system
244. 0.02 Bernier,, E (2012) the mobilar robot, ubiquitous, unobtrusive, augmented reality device
245. 0.02 Caruso,, G (2010) ar-mote: a wireless device for augmented reality environment
246. 0.02 Chen,, J (2010) [augmented reality registration algorithm based on nature feature recognition](http://dx.doi.org/10.1007/s11432-010-4026-5)
247. 0.02 Cho,, K (2011) [real-time recognition and tracking for augmented reality books](http://dx.doi.org/10.1002/cav.431)
248. 0.02 Cleary,, K (2010) [image-guided interventions: technology review and clinical applications](http://dx.doi.org/10.1146/annurev-bioeng-070909-105249)
249. 0.02 Corradini,, A (2010) natural multimodal interaction in collaborative visualization
250. 0.02 Csongei,, M (2012) clonar: rapid redesign of real-world objects
251. 0.02 Dalla, Mura, M (2012) [augmented reality: fusing the real and synthetic worlds](http://dx.doi.org/10.1109/IGARSS.2012.6351610)
252. 0.02 De, Crescenzio, F (2011) [augmented reality for aircraft maintenance training and operations support](http://dx.doi.org/10.1109/MCG.2011.4)
253. 0.02 De, Lucia, A (2010) smartbuilding: a people-to-people-to-geographical-places mobile system based on augmented reality
254. 0.02 Debenham,, P (2011) evolutionary augmented reality at the natural history museum
255. 0.02 Dong,, SY (2010) [robust mobile computing framework for visualization of simulated processes in augmented reality](http://dx.doi.org/10.1109/WSC.2010.5679004)
256. 0.02 Dornaika,, F (2010) [real-time object detection and tracking in video sequences](http://dx.doi.org/10.1117/12.838820)
257. 0.02 Ercan,, AO (2011) on sensor fusion for head tracking in augmented reality applications
258. 0.02 Estevez,, D (2015) [robot devastation: using diy low-cost platforms for multiplayer interaction in an augmented reality game](http://dx.doi.org/10.4108/icst.intetain.2015.259753)
259. 0.02 Fukuda,, T (2015) integration of cfd, vr, ar and bim for design feedback in a design process an experimental study
260. 0.02 Gee,, AP (2011) [a topometric system for wide area augmented reality](http://dx.doi.org/10.1016/j.cag.2011.04.006)
261. 0.02 Golparvar-Fard,, M (2011) [integrated sequential as-built and as-planned representation with d(4)ar tools in support of decision-making tasks in the aec/fm industry](http://dx.doi.org/10.1061/(ASCE)CO.1943-7862.0000371)
262. 0.02 Gonzalez,, FCJ (2014) [smart multi-level tool for remote patient monitoring based on a wireless sensor network and mobile augmented reality](http://dx.doi.org/10.3390/s140917212)
263. 0.02 Gonzalez-Gonzalez,, CS (2011) natural interaction without marks
264. 0.02 Graf,, H (2011) lifecycle building card: toward paperless and visual lifecycle management tools
265. 0.02 Gruber,, L (2012) real-time photometric registration from arbitrary geometry
266. 0.02 Hartl,, A (2012) rectangular target extraction for mobile augmented reality applications
267. 0.02 Henderson,, S (2010) [opportunistic tangible user interfaces for augmented reality](http://dx.doi.org/10.1109/TVCG.2009.91)
268. 0.02 Hutson,, M (2011) [janusvf: accurate navigation using scaat and virtual fiducials](http://dx.doi.org/10.1109/TVCG.2010.91)
269. 0.02 Ikeda,, S (2011) augmented reality system for visualizing 3-d region of interest in unknown environment
270. 0.02 Jachnik,, J (2012) real-time surface light-field capture for augmentation of planar specular surfaces
271. 0.02 Jang,, Y (2013) unified visual perception model for context-aware wearable ar
272. 0.02 Jin,, D (2011) development of real-time marker less augmented reality system using multi-thread design patterns
273. 0.02 Jo,, H (2011) [aroundplot: focus plus context interface for off-screen objects in 3d environments](http://dx.doi.org/10.1016/j.cag.2011.04.005)
274. 0.02 Jorissen,, L (2014) [robust global tracking using a seamless structured pattern of dots](http://dx.doi.org/10.1007/978-3-319-13969-2_17)
275. 0.02 Jung,, S (2010) a study on marker overlapping control for m2m-based augmented reality multiple object loading using bresenham algorithm
276. 0.02 Kahn,, S (2010) time-of-flight based scene reconstruction with a mesh processing tool for model based camera tracking
277. 0.02 Karlekar,, J (2010) [model-based localization and drift-free user tracking for outdoor augmented reality](http://dx.doi.org/10.1109/ICME.2010.5583850)
278. 0.02 Kauko,, J (2011) bidirectional pose estimation
279. 0.02 Kersten-Oertel,, M (2012) [dvv: a taxonomy for mixed reality visualization in image guided surgery](http://dx.doi.org/10.1109/TVCG.2011.50)
280. 0.02 Kim,, D (2012) non-marker based mobile augmented reality and its applications using object recognition
281. 0.02 Kim,, S (2010) [ar interfacing with prototype 3d applications based on user-centered interactivity](http://dx.doi.org/10.1016/j.cad.2008.10.009)
282. 0.02 Klein,, G (2010) [simulating low-cost cameras for augmented reality compositing](http://dx.doi.org/10.1109/TVCG.2009.210)
283. 0.02 Kobayashi,, T (2013) [novel keypoint registration for fast and robust pose detection on mobile phones](http://dx.doi.org/10.1109/ACPR.2013.67)
284. 0.02 Kyrki,, V (2011) [tracking rigid objects using integration of model-based and model-free cues](http://dx.doi.org/10.1007/s00138-009-0214-y)
285. 0.02 Langlotz,, T (2011) [robust detection and tracking of annotations for outdoor augmented reality browsing](http://dx.doi.org/10.1016/j.cag.2011.04.004)
286. 0.02 Lensing,, P (2012) instant indirect illumination for dynamic mixed reality scenes
287. 0.02 Lieberknecht,, S (2011) [benchmarking template-based tracking algorithms](http://dx.doi.org/10.1007/s10055-010-0185-3)
288. 0.02 Lieberknecht,, S (2011) [evolution of a tracking system](http://dx.doi.org/10.1007/978-1-4614-0064-6_17)
289. 0.02 Lin,, FY (2010) augmented reality with human body interaction based on monocular 3d pose estimation
290. 0.02 Liu,, DSM (2010) a cross-platform framework for physics-based collaborative augmented reality
291. 0.02 Lopez-Moreno,, J (2010) [compositing images through light source detection](http://dx.doi.org/10.1016/j.cag.2010.08.004)
292. 0.02 Lu,, YZ (2010) [augmented reality e-commerce system: a case study](http://dx.doi.org/10.1115/1.3385795)
293. 0.02 MacIntyre,, B (2011) the argon ar web browser and standards-based ar application environment
294. 0.02 Makita,, K (2013) photo-shoot localization of a mobile camera based on registered frame data of virtualized reality models
295. 0.02 Mirota,, DJ (2011) [vision-based navigation in image-guided interventions](http://dx.doi.org/10.1146/annurev-bioeng-071910-124757)
296. 0.02 Misker,, JMV (2010) [authoring immersive mixed reality experiences](http://dx.doi.org/10.1007/978-1-84882-733-2_14)
297. 0.02 Miyata,, K (2011) [applying ar technology with a projector-camera system in a history museum](http://dx.doi.org/10.1117/12.872179)
298. 0.02 Morrison,, A (2011) [collaborative use of mobile augmented reality with paper maps](http://dx.doi.org/10.1016/j.cag.2011.04.009)
299. 0.02 Nakashima,, Y (2015) [ar image generation using view-dependent geometry modification and texture mapping](http://dx.doi.org/10.1007/s10055-015-0259-3)
300. 0.02 Navab,, N (2012) [first deployments of augmented reality in operating rooms](http://dx.doi.org/10.1109/MC.2012.75)
301. 0.02 Newcombe,, RA (2010) [live dense reconstruction with a single moving camera](http://dx.doi.org/10.1109/CVPR.2010.5539794)
302. 0.02 Oberhofer,, C (2012) natural feature tracking in javascript
303. 0.02 Oda,, O (2012) 3d referencing techniques for physical objects in shared augmented reality
304. 0.02 Oh,, S (2010) [synthetic vision-based perceptual attention for augmented reality agents](http://dx.doi.org/10.1002/cav.368)
305. 0.02 Okada,, N (2015) [manseibashi reminiscent window: on-site ar exhibition system using mobile devices](http://dx.doi.org/10.1007/978-3-319-20804-6_32)
306. 0.02 Olmedo,, H (2013) [virtuality continuum's state of the art](http://dx.doi.org/10.1016/j.procs.2013.11.032)
307. 0.02 Omercevic,, D (2011) [hyperlinking reality via camera phones](http://dx.doi.org/10.1007/s00138-010-0285-9)
308. 0.02 Pan,, Q (2011) rapid scene reconstruction on mobile phones from panoramic images
309. 0.02 Park,, H (2010) [automatic confidence adjustment of visual cues in model-based camera tracking](http://dx.doi.org/10.1002/cav.321)
310. 0.02 Park,, Y (2011) [extended keyframe detection with stable tracking for multiple 3d object tracking](http://dx.doi.org/10.1109/TVCG.2010.262)
311. 0.02 Pastor-Moreno,, D (2015) optical flow localisation and appearance mapping (oflaam) for long-term navigation
312. 0.02 Pokric,, B (2015) engaging citizen communities in smart cities using iot, serious gaming and fast markerless augmented reality
313. 0.02 Prochazka,, D (2011) mobile augmented reality applications
314. 0.02 Pustka,, D (2011) [automatic configuration of pervasive sensor networks for augmented reality](http://dx.doi.org/10.1109/MPRV.2010.50)
315. 0.02 Ramadasan,, D (2015) dcslam: a dynamically constrained real-time slam
316. 0.02 Rolland,, JP (2012) [see-through head worn display (hwd) architectures](http://dx.doi.org/10.1007/978-3-540-79567-4_10.4.1)
317. 0.02 Rosenthal,, S (2010) augmenting on-screen instructions with micro-projected guides: when it works, and when it fails
318. 0.02 Ryu,, NH (2011) implementation of augmented reality system using the infrared rays led marker based on hybrid tracking
319. 0.02 Saaski,, J (2010) augmented reality in factory environments - system usability and human factors studies
320. 0.02 Scheggi,, S (2010) shape and weight rendering for haptic augmented reality
321. 0.02 Seo,, BK (2011) 3-d visual tracking for mobile augmented reality applications
322. 0.02 Seo,, BK (2011) augmented reality-based on-site tour guide: a study in gyeongbokgung
323. 0.02 Seo,, BK (2012) [real-time visual tracking of less textured three-dimensional objects on mobile platforms](http://dx.doi.org/10.1117/1.OE.51.12.127202)
324. 0.02 Shen,, Y (2011) [vision-based hand interaction in augmented reality environment](http://dx.doi.org/10.1080/10447318.2011.555297)
325. 0.02 Stork,, S (2010) [human cognition in manual assembly: theories and applications](http://dx.doi.org/10.1016/j.aei.2010.05.010)
326. 0.02 Sukan,, M (2012) quick viewpoint switching for manipulating virtual objects in hand-held augmented reality using stored snapshots
327. 0.02 Sweeney,, C (2013) improved outdoor augmented reality through "globalization"
328. 0.02 Takacs,, G (2010) [unified real-time tracking and recognition with rotation-invariant fast features](http://dx.doi.org/10.1109/CVPR.2010.5540116)
329. 0.02 Teng,, CH (2012) [an augmented reality environment for learning opengl programming](http://dx.doi.org/10.1109/UIC-ATC.2012.57)
330. 0.02 Teng,, CH (2012) [developing qr code based augmented reality using sift features](http://dx.doi.org/10.1109/UIC-ATC.2012.56)
331. 0.02 Tian,, Y (2010) [real-time occlusion handling in augmented reality based on an object tracking approach](http://dx.doi.org/10.3390/s100402885)
332. 0.02 Tian,, YA (2010) [an automatic occlusion handling method in augmented reality](http://dx.doi.org/10.1108/02602281011051399)
333. 0.02 Tsai,, SS (2010) [fast geometric re-ranking for image-based retrieval](http://dx.doi.org/10.1109/ICIP.2010.5648942)
334. 0.02 Uva,, AE (2010) [distributed design review using tangible augmented technical drawings](http://dx.doi.org/10.1016/j.cad.2008.10.015)
335. 0.02 Vazquez-Martin,, R (2012) [unified framework for recognition, localization and mapping using wearable cameras](http://dx.doi.org/10.1007/s10339-012-0496-2)
336. 0.02 Verbelen,, T (2011) [dynamic deployment and quality adaptation for mobile augmented reality applications](http://dx.doi.org/10.1016/j.jss.2011.06.063)
337. 0.02 Vidal,, NR (2010) [augmented reality systems for weed economic thresholds applications](http://dx.doi.org/10.1590/S0100-83582010000200025)
338. 0.02 Wang,, H (2015) [harco: hierarchical fiducial markers for pose estimation in helicopter landing tasks](http://dx.doi.org/10.1109/SMC.2015.343)
339. 0.02 Wang,, J (2012) augmented reality during angiography: integration of a virtual mirror for improved 2d/3d visualization
340. 0.02 Wang,, XW (2010) [endoscopic video texture mapping on pre-built 3-d anatomical objects without camera tracking](http://dx.doi.org/10.1109/TMI.2009.2028341)
341. 0.02 Wang,, YT (2011) [prototyping a mobile ar based multi-user guide system for yuanmingyuan garden](http://dx.doi.org/10.1007/978-1-4419-9845-3_10)
342. 0.02 Wei,, BC (2015) [wide area localization and tracking on camera phones for mobile augmented reality systems](http://dx.doi.org/10.1007/s00530-014-0364-2)
343. 0.02 Wetzstein,, G (2010) [optical image processing using light modulation displays](http://dx.doi.org/10.1111/j.1467-8659.2010.01660.x)
344. 0.02 Yip,, MC (2012) [tissue tracking and registration for image-guided surgery](http://dx.doi.org/10.1109/TMI.2012.2212718)
345. 0.02 Yoo,, J (2010) online scene modeling for interactive ar applications
346. 0.02 Zhang,, J (2011) [rfid-assisted assembly guidance system in an augmented reality environment](http://dx.doi.org/10.1080/00207543.2010.492802)
347. 0.02 Zheng,, ZR (2010) [design and fabrication of an off-axis see-through head-mounted display with an x-y polynomial surface](http://dx.doi.org/10.1364/AO.49.003661)
348. 0.02 de, Sorbier, F (2012) violin pedagogy for finger and bow placement using augmented reality
349. 0.02 van, den Berg, NS (2013) [sentinel lymph node biopsy for prostate cancer: a hybrid approach](http://dx.doi.org/10.2967/jnumed.112.113746)
350. 0.57 Behringer,, R (1999) [registration for an augmented reality system enhancing the situational awareness in an outdoor scenario](http://dx.doi.org/10.1117/12.354425)
351. 0.57 Behringer,, R (1999) [registration for outdoor augmented reality applications using computer vision techniques and hybrid sensors](http://dx.doi.org/10.1109/VR.1999.756958)
352. 0.5 Berger,, JW (1999) computer-vision-enabled ophthalmic augmented reality: a pc-based prototype
353. 0.5 Berger,, JW (1999) [image-guided macular laser therapy: design considerations and progress towards implementation](http://dx.doi.org/10.1117/12.350586)
354. 0.43 Berger,, JW (1999) [computer-vision-enabled augmented reality fundus biomicroscopy](http://dx.doi.org/10.1016/S0161-6420(99)90404-9)
355. 0.36 Behringer,, R (1999) a novel interface for device diagnostics using speech recognition, augmented reality visualization, and 3d audio auralization
356. 0.21 Milgram,, P (1999) a taxonomy of real and virtual world display integration
357. 0.14 Behringer,, R (1999) [a distributed device diagnostics system utilizing augmented reality and 3d audio](http://dx.doi.org/10.1016/S0097-8493(99)00108-9)
358. 0.14 Klinker,, G (1999) augmented reality: a balancing act between high quality and real-time constraints
359. 0.07 Azuma,, RT (1999) the challenge of making augmented reality work outdoors
360. 0.07 Behringer,, R (1999) international workshop on augmented reality 1998 - overview and summary
361. 0.07 Blackwell,, M (2000) [an image overlay system for medical data visualization](http://dx.doi.org/10.1016/S1361-8415(00)00007-4)
362. 0.07 Hariprasad,, R (1999) an intelligent, interactive platform for ophthalmic teaching, telemedicine, and telecollaboration: design considerations and prototype construction
363. 0.07 Jang,, B (1999) an outdoor augmented reality system for gis applications
364. 0.07 Kim,, H (1999) [a birds-eye view system using augmented reality](http://dx.doi.org/10.1109/SIMSYM.1999.766463)
365. 0.07 Raghavan,, V (1999) [interactive evaluation of assembly sequences using augmented reality](http://dx.doi.org/10.1109/70.768177)
366. 0.07 Raskar,, R (1999) spatially augmented reality
367. 0.07 Spohrer,, JC (1999) [information in places](http://dx.doi.org/10.1147/sj.384.0602)
368. 0.07 Sundareswaran,, V (1999) visual servoing-based augmented reality
369. 0.07 Yokoya,, N (1999) stereo vision based video see-through mixed reality
370. 0.23 Birkfellner,, W (2000) the varioscope ar - a head-mounted operating microscope for augmented reality
371. 0.23 Figl,, M (2002) [pc-based control unit for a head mounted operating microscope for augmented reality visualization in surgical navigation](http://dx.doi.org/10.1117/12.466983)
372. 0.23 Wanschitz,, F (2002) [computer-enhanced stereoscopic vision in a head-mounted display for oral implant surgery](http://dx.doi.org/10.1034/j.1600-0501.2002.130606.x)
373. 0.15 Birkfellner,, W (2000) [development of the varioscope ar a see-through hmd for computer- aided surgery](http://dx.doi.org/10.1109/ISAR.2000.880923)
374. 0.15 Birkfellner,, W (2001) [calibration of a head-mounted operating microscope for augmented reality visualization in cas](http://dx.doi.org/10.1016/S0531-5131(01)00034-6)
375. 0.15 Birkfellner,, W (2002) [a head-mounted operating binocular for augmented reality visualization in medicine - design and initial evaluation](http://dx.doi.org/10.1109/TMI.2002.803099)
376. 0.15 Birkfellner,, W (2002) [stereoscopic visualization in the varioscope ar: a see-through head mounted display for surgical navigation](http://dx.doi.org/10.1117/12.466947)
377. 0.15 Weidenbach,, M (2000) [augmented reality simulator for training in two-dimensional echocardiography](http://dx.doi.org/10.1006/cbmr.1999.1527)
378. 0.08 Berger,, JW (1999) [computer-vision-enabled augmented reality fundus biomicroscopy](http://dx.doi.org/10.1016/S0161-6420(99)90404-9)
379. 0.08 Berger,, JW (1999) [image-guided macular laser therapy: design considerations and progress towards implementation](http://dx.doi.org/10.1117/12.350586)
380. 0.08 Berger,, JW (2001) [augmented reality fundus biomicroscopy - a working clinical prototype](http://dx.doi.org/10.1001/archopht.119.12.1815)
381. 0.08 Birkfellner,, W (2001) [calibration of projection parameters in the varioscope ar, a head-mounted display for augmented reality visualization in image-guided therapy](http://dx.doi.org/10.1117/12.428089)
382. 0.08 Edwards,, PJE (1999) stereo augmented reality in the surgical microscope
383. 0.08 Figl,, M (2001) [current status of the varioscope ar, a head-mounted operating microscope for computer-aided surgery](http://dx.doi.org/10.1109/ISAR.2001.970512)
384. 0.08 King,, AP (1999) [a system for microscope-assisted guided interventions](http://dx.doi.org/10.1159/000029708)
385. 0.08 Madjarov,, B (2001) [video injection for slitlamp augmented reality](http://dx.doi.org/10.1117/12.429270)
386. 0.08 Maurer,, CR (2001) augmented reality visualization of brain structures with stereo and kinetic depth cues: system description and initial evaluation with head phantom
387. 0.08 Rolland,, JP (2000) [optical versus video see-through mead-mounted displays in medical visualization](http://dx.doi.org/10.1162/105474600566808)
388. 0.08 Rosenthal,, M (2002) [augmented reality guidance for needle biopsies: an initial randomized, controlled trial in phantoms](http://dx.doi.org/10.1016/S1361-8415(02)00088-9)
389. 0.08 Salb,, T (2000) [intraoperative presentation of surgical planning and simulation results using a stereoscopic see-through head-mounted display](http://dx.doi.org/10.1117/12.384431)
390. 0.08 Weidenbach,, M (2000) [augmented reality in echocardiography - a new method of computer supported training and image processing using virtual and real three-dimensional data sets](http://dx.doi.org/10.1007/s003920050463)
391. 0.91 BAUDEL,, T (1993) [charade - remote-control of objects using free-hand gestures](http://dx.doi.org/10.1145/159544.159562)
392. 0.09 Spohrer,, JC (1999) [information in places](http://dx.doi.org/10.1147/sj.384.0602)
393. 0.88 LAVALLEE,, S (1995) [building a hybrid patients model for augmented reality in surgery - a registration problem](http://dx.doi.org/10.1016/0010-4825(95)00004-N)
394. 0.12 Herve,, JY (2000) dynamic registration for augmented reality in telerobotics applications
395. 0.12 Tonet,, O (2000) an augmented reality navigation system for computer assisted arthroscopic surgery of the knee
396. 0.88 Breen,, DE (1996) interactive occlusion and automatic object placement for augmented reality
397. 0.12 Klinker,, G (1999) augmented reality: a balancing act between high quality and real-time constraints
398. 0.86 Li,, GY (2003) manipulating nano scale biological specimen in liquid
399. 0.86 Li,, GY (2003) modeling of 3-d interactive forces in nanomanipulation
400. 0.71 Li,, GY (2003) augmented reality system for real-time nanomanipulation
401. 0.71 Li,, GY (2003) manipulation of living cells by atomic force microscopy
402. 0.14 Marchi,, F (2010) an augmented reality nanomanipulator for learning nanophysics: the "nanolearner" platform
403. 1 Fuchs,, H (1996) towards performing ultrasound-guided needle biopsies from within a head-mounted display
404. 0.67 WAGNER,, A (1995) [virtual image-guided navigation in tumor surgery - technical innovation](http://dx.doi.org/10.1016/S1010-5182(05)80155-6)
405. 0.17 Birkfellner,, W (1999) computer-aided implant dentistry - an early report
406. 0.17 Wagner,, A (1996) [image guided surgery](http://dx.doi.org/10.1016/S0901-5027(96)80062-2)
407. 0.8 Turunen,, T (2001) performance evaluation of service connectivity in mobile augmented reality
408. 0.8 Turunen,, T (2001) performance of a service connectivity architecture for mobile augmented reality
409. 0.8 Turunen,, T (2001) [service connectivity architecture for mobile augmented reality](http://dx.doi.org/10.1117/12.430839)
410. 0.2 Pyssysalo,, T (2000) [adaptive streaming protocol for mobile augmented reality-based concurrent engineering](http://dx.doi.org/10.1117/12.384468)
411. 0.6 Berger,, MO (1999) [mixing synthetic and video images of an outdoor urban environment](http://dx.doi.org/10.1007/s001380050098)
412. 0.2 Berger,, MO (1996) compositing computer and video image sequences: robust algorithms for the reconstruction of the camera parameters
413. 0.2 Du,, W (1999) viewpoint computation from the perspective projection of a rectangle
414. 0.23 Neumann,, U (1999) augmented reality tracking in natural environments
415. 0.18 Neumann,, U (1999) [natural feature tracking for augmented reality](http://dx.doi.org/10.1109/6046.748171)
416. 0.15 You,, S (1999) [hybrid inertial and vision tracking for augmented reality registration](http://dx.doi.org/10.1109/VR.1999.756960)
417. 0.12 Park,, J (1999) natural feature tracking for extendible robust augmented realities
418. 0.11 Cho,, Y (1999) a multi-ring fiducial system and an intensity-invariant detection method for scalable augmented reality
419. 0.11 Stricker,, D (1999) a fast and robust line-based optical tracker for augmented reality applications
420. 0.09 Kanbara,, M (1999) real-time composition of stereo images for video see-through augmented reality
421. 0.09 TUCERYAN,, M (1995) [calibration requirements and procedures for a monitor-based augmented reality system](http://dx.doi.org/10.1109/2945.466720)
422. 0.09 WHITAKER,, RT (1995) [object calibration for augmented reality](http://dx.doi.org/10.1111/1467-8659.1430015)
423. 0.09 Yokoya,, N (1999) stereo vision based video see-through mixed reality
424. 0.08 Raghavan,, V (1999) [interactive evaluation of assembly sequences using augmented reality](http://dx.doi.org/10.1109/70.768177)
425. 0.08 Tuceryan,, M (2000) [single point active alignment method (spaam) for optical see-through hmd calibration for ar](http://dx.doi.org/10.1109/ISAR.2000.880938)
426. 0.06 Berger,, JW (1999) computer-vision-enabled ophthalmic augmented reality: a pc-based prototype
427. 0.06 Chung,, KH (1999) [an application of augmented reality to thickness inspection](http://dx.doi.org/10.1002/(SICI)1520-6564(199923)9:4%3c331::AID-HFM1%3e3.0.CO;2-3)
428. 0.06 Jang,, B (1999) an outdoor augmented reality system for gis applications
429. 0.06 Yokoya,, N (1999) passive range sensing techniques: depth from images
430. 0.05 Akhloufi,, MA (1999) virtual view synthesis from uncalibrated stereo cameras
431. 0.05 Azuma,, R (1999) [tracking in unprepared environments for augmented reality systems](http://dx.doi.org/10.1016/S0097-8493(99)00104-1)
432. 0.05 BAJURA,, M (1995) [dynamic registration correction in video-based augmented reality systems](http://dx.doi.org/10.1109/38.403828)
433. 0.05 Berger,, JW (1999) [computer-vision-enabled augmented reality fundus biomicroscopy](http://dx.doi.org/10.1016/S0161-6420(99)90404-9)
434. 0.05 Berger,, JW (1999) [image-guided macular laser therapy: design considerations and progress towards implementation](http://dx.doi.org/10.1117/12.350586)
435. 0.05 Feiner,, S (1999) wearing it out: first steps toward mobile augmented reality systems
436. 0.05 Klinker,, G (1999) augmented reality: a balancing act between high quality and real-time constraints
437. 0.05 Lee,, JW (1999) [rule-based segmentation for intensity-adaptive fiducial detection](http://dx.doi.org/10.1117/12.348425)
438. 0.05 Rolland,, JP (2000) [optical versus video see-through mead-mounted displays in medical visualization](http://dx.doi.org/10.1162/105474600566808)
439. 0.05 Sato,, I (1999) [acquiring a radiance distribution to superimpose virtual objects onto a real scene](http://dx.doi.org/10.1109/2945.764865)
440. 0.05 Satoh,, K (1999) case studies of see-through augmentation in mixed reality project
441. 0.05 Sundareswaran,, V (1999) visual servoing-based augmented reality
442. 0.05 Terashima,, M (1999) a human-robot interface using an extended digital desk
443. 0.03 Azuma,, RT (1997) [a survey of augmented reality](http://dx.doi.org/10.1162/pres.1997.6.4.355)
444. 0.03 Behringer,, R (1999) a novel interface for device diagnostics using speech recognition, augmented reality visualization, and 3d audio auralization
445. 0.03 Behringer,, R (1999) improving registration precision through visual horizon silhouette matching
446. 0.03 Curtis,, D (1999) several devils in the details: making an ar application work in the airplane factory
447. 0.03 Dorfmuller,, K (1999) [robust tracking for augmented reality using retroreflective markers](http://dx.doi.org/10.1016/S0097-8493(99)00105-3)
448. 0.03 FEINER,, SK (1995) [architectural anatomy](http://dx.doi.org/10.1162/pres.1995.4.3.318)
449. 0.03 Kutulakos,, KN (1998) [calibration-free augmented reality](http://dx.doi.org/10.1109/2945.675647)
450. 0.03 Madritsch,, F (1996) ccd-camera based optical beacon tracking for virtual and augmented reality
451. 0.03 Mendelsohn,, J (1999) constrained self-calibration for augmented reality registration
452. 0.03 Milgram,, P (1999) a framework for relating head-mounted displays to mixed reality displays
453. 0.03 Molineros,, J (1999) areas: augmented reality for evaluating assembly sequences
454. 0.03 Reiners,, D (1999) augmented reality for construction tasks: doorlock assembly
455. 0.03 Spohrer,, JC (1999) [information in places](http://dx.doi.org/10.1147/sj.384.0602)
456. 0.03 Tamura,, H (1999) steps toward seamless mixed reality
457. 0.03 Vallino,, J (1999) haptics in augmented reality
458. 0.03 You,, SY (1999) [orientation tracking for outdoor augmented reality registration](http://dx.doi.org/10.1109/38.799738)
459. 0.03 Young,, AL (1999) the potential of augmented reality technology for training support systems.
460. 0.02 Azuma,, R (1999) [a motion-stabilized outdoor augmented reality system](http://dx.doi.org/10.1109/VR.1999.756959)
461. 0.02 Azuma,, RT (1999) the challenge of making augmented reality work outdoors
462. 0.02 Behringer,, R (1999) [a distributed device diagnostics system utilizing augmented reality and 3d audio](http://dx.doi.org/10.1016/S0097-8493(99)00108-9)
463. 0.02 Behringer,, R (1999) international workshop on augmented reality 1998 - overview and summary
464. 0.02 Behringer,, R (1999) [registration for an augmented reality system enhancing the situational awareness in an outdoor scenario](http://dx.doi.org/10.1117/12.354425)
465. 0.02 Berger,, MO (1999) [mixing synthetic and video images of an outdoor urban environment](http://dx.doi.org/10.1007/s001380050098)
466. 0.02 Chen,, WC (2000) [toward a compelling sensation of telepresence: demonstrating a portal to a distant (static) office](http://dx.doi.org/10.1109/VISUAL.2000.885712)
467. 0.02 Dubois,, E (1999) classification space for augmented surgery, an augmented reality case study
468. 0.02 Faugeras,, O (1998) [3-d reconstruction of urban scenes from image sequences](http://dx.doi.org/10.1006/cviu.1998.0665)
469. 0.02 Fuchs,, H (1998) augmented reality visualization for laparoscopic surgery
470. 0.02 Genc,, Y (2000) [optical see-through hmd calibration: a stereo method validated with a video see-through system](http://dx.doi.org/10.1109/ISAR.2000.880940)
471. 0.02 Haniff,, D (1999) augmented reality and computer-assisted learning
472. 0.02 Hollerer,, T (1999) [exploring mars: developing indoor and outdoor user interfaces to a mobile augmented reality system](http://dx.doi.org/10.1016/S0097-8493(99)00103-X)
473. 0.02 Holloway,, RL (1997) [registration error analysis for augmented reality](http://dx.doi.org/10.1162/pres.1997.6.4.413)
474. 0.02 Hoshino,, J (2001) [a match moving technique for merging cg cloth and human movie sequences](http://dx.doi.org/10.1002/vis.242)
475. 0.02 Hoshino,, J (2001) interactive virtual fashion simulator
476. 0.02 Kim,, H (1999) [a birds-eye view system using augmented reality](http://dx.doi.org/10.1109/SIMSYM.1999.766463)
477. 0.02 Kuijper,, F (1999) [tracking systems and the value of inertial technology](http://dx.doi.org/10.1117/12.349403)
478. 0.02 Lu,, CY (1999) virtual and augmented reality technologies for product realization
479. 0.02 MacIntyre,, B (2000) [adapting to dynamic registration errors using level of error (loe) filtering](http://dx.doi.org/10.1109/ISAR.2000.880927)
480. 0.02 Maurer,, CR (2001) augmented reality visualization of brain structures with stereo and kinetic depth cues: system description and initial evaluation with head phantom
481. 0.02 Milgram,, P (1999) a taxonomy of real and virtual world display integration
482. 0.02 Pasman,, W (1999) [accurate overlaying for mobile augmented reality](http://dx.doi.org/10.1016/S0097-8493(99)00118-1)
483. 0.02 Plesniak,, W (1999) spatial interaction with haptic holograms
484. 0.02 Saito,, H (2001) a match moving technique for merging cg and human video sequences
485. 0.02 Sauer,, F (2000) [augmented workspace: designing an ar testbed](http://dx.doi.org/10.1109/ISAR.2000.880922)
486. 0.02 Seo,, Y (2000) [calibration-free augmented reality in perspective](http://dx.doi.org/10.1109/2945.895879)
487. 0.02 Seo,, Y (2000) [weakly calibrated video-based augmented reality: embedding and rendering through virtual camera](http://dx.doi.org/10.1109/ISAR.2000.880936)
488. 0.02 Sharma,, R (1997) [computer vision-based augmented reality for guiding manual assembly](http://dx.doi.org/10.1162/pres.1997.6.3.292)
489. 0.02 Tang,, SL (1998) [augmented reality systems for medical applications](http://dx.doi.org/10.1109/51.677169)
490. 0.8 AHLERS,, KH (1995) [distributed augmented reality for collaborative design applications](http://dx.doi.org/10.1111/1467-8659.1430003)
491. 0.2 Billinghurst,, M (1999) collaborative mixed reality
492. 0.2 Schmalstieg,, D (1999) [sewing worlds together with seams: a mechanism to construct complex virtual environments](http://dx.doi.org/10.1162/105474699566332)
493. 1 Ciulli,, N (1998) [a cooperative environment based on augmented reality: from telepresence to performance issues](http://dx.doi.org/10.1016/S0169-7552(98)00165-2)
494. 0.09 Figl,, M (2010) [image guidance for robotic minimally invasive coronary artery bypass](http://dx.doi.org/10.1016/j.compmedimag.2009.08.002)
495. 0.09 Hansen,, C (2010) [illustrative visualization of 3d planning models for augmented reality in liver surgery](http://dx.doi.org/10.1007/s11548-009-0365-3)
496. 0.09 Navab,, N (2010) [camera augmented mobile c-arm (camc): calibration, accuracy study, and clinical applications](http://dx.doi.org/10.1109/TMI.2009.2021947)
497. 0.09 Sugimoto,, M (2010) [image overlay navigation by markerless surface registration in gastrointestinal, hepatobiliary and pancreatic surgery](http://dx.doi.org/10.1007/s00534-009-0199-y)
498. 0.09 Ukimura,, O (2010) [image-guided surgery in minimally invasive urology](http://dx.doi.org/10.1097/MOU.0b013e3283362610)
499. 0.09 Yu,, DG (2010) [a useful visualization technique: a literature review for augmented reality and its application, limitation & future direction](http://dx.doi.org/10.1007/978-1-4419-0312-9_21)
500. 0.07 Ukimura,, O (2010) [augmented reality for image-guided surgery in urology](http://dx.doi.org/10.1007/978-1-84882-178-1_25)
501. 0.07 Ukimura,, O (2010) [evolution of precise and multimodal mri and trus in detection and management of early prostate cancer](http://dx.doi.org/10.1586/ERD.10.24)
502. 0.05 Cleary,, K (2010) [image-guided interventions: technology review and clinical applications](http://dx.doi.org/10.1146/annurev-bioeng-070909-105249)
503. 0.05 Lamata,, P (2010) simulation, planning and navigation in laparoscopic surgery: current status and challenges
504. 0.05 Liao,, HE (2010) [3-d augmented reality for mri-guided surgery using integral videography autostereoscopic image overlay](http://dx.doi.org/10.1109/TBME.2010.2040278)
505. 0.05 Pratt,, P (2010) dynamic guidance for robotic surgery using image-constrained biomechanical models
506. 0.05 Shekhar,, R (2010) [live augmented reality: a new visualization method for laparoscopic surgery using continuous volumetric computed tomography](http://dx.doi.org/10.1007/s00464-010-0890-8)
507. 0.05 Yip,, MC (2010) 3d ultrasound to stereoscopic camera registration through an air-tissue boundary
508. 0.04 Azuma,, R (2001) [recent advances in augmented reality](http://dx.doi.org/10.1109/38.963459)
509. 0.04 Cheung,, CL (2010) fused video and ultrasound images for minimally invasive partial nephrectomy: a phantom study
510. 0.04 Cohen,, D (2010) augmented reality image guidance in minimally invasive prostatectomy
511. 0.04 Hua,, H (2002) [calibration of a head-mounted projective display for augmented reality systems](http://dx.doi.org/10.1109/ISMAR.2002.1115087)
512. 0.04 Hua,, H (2002) [design of an ultra-light head-mounted projective display (hmpd) and its applications in augmented collaborative environments](http://dx.doi.org/10.1117/12.468067)
513. 0.04 Hua,, H (2002) [study of the imaging properties of retro-reflective materials used in head-mounted projective displays (hmpds)](http://dx.doi.org/10.1117/12.478871)
514. 0.04 Lee,, JD (2010) [fast-micp for frameless image-guided surgery](http://dx.doi.org/10.1118/1.3470097)
515. 0.04 Lee,, SL (2010) [from medical images to minimally invasive intervention: computer assistance for robotic surgery](http://dx.doi.org/10.1016/j.compmedimag.2009.07.007)
516. 0.04 Maurer,, CR (2001) augmented reality visualization of brain structures with stereo and kinetic depth cues: system description and initial evaluation with head phantom
517. 0.04 Mountney,, P (2010) motion compensated slam for image guided surgery
518. 0.04 Nakamoto,, M (2012) [current progress on augmented reality visualization in endoscopic surgery](http://dx.doi.org/10.1097/MOU.0b013e3283501774)
519. 0.04 Nakamura,, K (2010) [surgical navigation using three-dimensional computed tomography images fused intraoperatively with live video](http://dx.doi.org/10.1089/end.2009.0365)
520. 0.04 Nicolau,, SA (2010) augmented reality systems for medical interventions: current limits
521. 0.04 Park,, A (2010) computed tomography guided laparoscopy: proof of concept
522. 0.04 Roche,, B (2010) [future treatment](http://dx.doi.org/10.1007/978-88-470-1542-5_47)
523. 0.04 Rolland,, JP (2000) [optical versus video see-through mead-mounted displays in medical visualization](http://dx.doi.org/10.1162/105474600566808)
524. 0.04 Sauer,, F (2001) [augmented reality visualization of ultrasound images: system description, calibration, and features](http://dx.doi.org/10.1109/ISAR.2001.970513)
525. 0.04 Teber,, D (2010) [in-vitro evaluation of a soft-tissue navigation system for laparoscopic prostatectomy](http://dx.doi.org/10.1089/end.2009.0289)
526. 0.04 Vemuri,, AS (2012) [deformable three-dimensional model architecture for interactive augmented reality in minimally invasive surgery](http://dx.doi.org/10.1007/s00464-012-2395-0)
527. 0.04 Vogt,, S (2002) [single camera tracking of marker clusters: multiparameter cluster optimization and experimental verification](http://dx.doi.org/10.1109/ISMAR.2002.1115082)
528. 0.04 Wu,, C (2010) [a full geometric and photometric calibration method for oblique-viewing endoscopes](http://dx.doi.org/10.3109/10929081003718758)
529. 0.02 Argotti,, Y (2001) [dynamic superimposition of synthetic objects on rigid and simple-deformable real objects](http://dx.doi.org/10.1109/ISAR.2001.970510)
530. 0.02 Argotti,, Y (2002) [dynamic superimposition of synthetic objects on rigid and simple-deformable real objects](http://dx.doi.org/10.1016/S0097-8493(02)00180-2)
531. 0.02 Audet,, S (2010) [direct image alignment of projector-camera systems with planar surfaces](http://dx.doi.org/10.1109/CVPR.2010.5540199)
532. 0.02 Baumhauer,, M (2010) [the mitk image guided therapy toolkit and its application for augmented reality in laparoscopic prostate surgery](http://dx.doi.org/10.1117/12.844667)
533. 0.02 Bianch,, G (2006) high-fidelity visuo-haptic interaction with virtual objects in multi-modal ar systems
534. 0.02 Bimber,, O (2002) [merging fossil specimens with computer-generated information](http://dx.doi.org/10.1109/MC.2002.1033024)
535. 0.02 Birkfellner,, W (1999) computer-aided implant dentistry - an early report
536. 0.02 Botella,, C (2010) [treating cockroach phobia with augmented reality](http://dx.doi.org/10.1016/j.beth.2009.07.002)
537. 0.02 Chien,, CH (2010) an interactive augmented reality system for learning anatomy structure
538. 0.02 Davis,, L (2002) [application of augmented reality to visualizing anatomical airways](http://dx.doi.org/10.1117/12.478890)
539. 0.02 Davis,, L (2002) augmented reality and training for airway management procedures
540. 0.02 Djaghloul,, H (2010) calibration-free markerless augmented reality in monocular laparoscopic cholecystectomy
541. 0.02 Edgcumbe,, P (2014) pico lantern: a pick-up projector for augmented reality in laparoscopic surgery
542. 0.02 Fichtinger,, G (2005) [image overlay guidance for needle insertion in ct scanner](http://dx.doi.org/10.1109/TBME.2005.851493)
543. 0.02 Figl,, M (2002) [pc-based control unit for a head mounted operating microscope for augmented reality visualization in surgical navigation](http://dx.doi.org/10.1117/12.466983)
544. 0.02 Fritz,, J (2012) [augmented reality visualization with use of image overlay technology for mr imaging-guided interventions: assessment of performance in cadaveric shoulder and hip arthrography at 1.5 t](http://dx.doi.org/10.1148/radiol.12112640)
545. 0.02 Furmanski,, C (2002) [augmented-reality visualizations guided by cognition: perceptual heuristics for combining visible and obscured information](http://dx.doi.org/10.1109/ISMAR.2002.1115091)
546. 0.02 Gavaghan,, KA (2011) [a portable image overlay projection device for computer-aided open liver surgery](http://dx.doi.org/10.1109/TBME.2011.2126572)
547. 0.02 Grasa,, OG (2014) [visual slam for handheld monocular endoscope](http://dx.doi.org/10.1109/TMI.2013.2282997)
548. 0.02 Grobelski,, B (2010) [new ways of visualization in laparoscopic surgery](http://dx.doi.org/10.5114/wiitm.2010.16425)
549. 0.02 Halic,, T (2010) [mixed reality simulation of rasping procedure in artificial cervical disc replacement (acdr) surgery](http://dx.doi.org/10.1186/1471-2105-11-S6-S11)
550. 0.02 Haouchine,, N (2014) towards an accurate tracking of liver tumors for augmented reality in robotic assisted surgery
551. 0.02 Hung,, AJ (2012) [robotic transrectal ultrasonography during robot-assisted radical prostatectomy](http://dx.doi.org/10.1016/j.eururo.2012.04.032)
552. 0.02 Ito,, E (2010) [magnetically guided 3-dimensional virtual neuronavigation for neuroendoscopic surgery: technique and clinical experience](http://dx.doi.org/10.1227/01.NEU.0000369659.19479.AF)
553. 0.02 Kersten-Oertel,, M (2013) volume visualization for neurovascular augmented reality surgery
554. 0.02 Khamene,, A (2003) an augmented reality system for mri-guided needle biopsies
555. 0.02 Khamene,, A (2003) local 3d reconstruction and augmented reality visualization of free-hand ultrasound for needle biopsy procedures
556. 0.02 Liang,, JT (2012) [a fluorolaser navigation system to guide linear surgical tool insertion](http://dx.doi.org/10.1007/s11548-012-0743-0)
557. 0.02 Liao,, HE (2011) [3d medical imaging and augmented reality for image-guided surgery](http://dx.doi.org/10.1007/978-1-4614-0064-6_27)
558. 0.02 Liu,, W (2010) [an efficient zoom tracking method for pan-tilt-zoom camera](http://dx.doi.org/10.1109/ICCSIT.2010.5564441)
559. 0.02 Malik,, S (2002) [hand tracking for interactive pattern-based augmented reality](http://dx.doi.org/10.1109/ISMAR.2002.1115080)
560. 0.02 Mansoux,, B (2005) the mini-screen: an innovative device for computer assisted surgery systems
561. 0.02 McDonald,, C (2002) hand-based interaction in augmented reality
562. 0.02 Mezzana,, P (2011) [augmented reality in oculoplastic surgery: first iphone application](http://dx.doi.org/10.1097/PRS.0b013e31820632eb)
563. 0.02 Mutter,, D (2010) [recent advances in liver imaging](http://dx.doi.org/10.1586/EGH.10.57)
564. 0.02 Nicolau,, S (2011) [augmented reality in laparoscopic surgical oncology](http://dx.doi.org/10.1016/j.suronc.2011.07.002)
565. 0.02 Nicolau,, SA (2005) a complete augmented reality guidance system for liver punctures: first clinical evaluation
566. 0.02 Olbrich,, B (2005) [respiratory motion analysis: towards gated augmentation of the liver](http://dx.doi.org/10.1016/j.ics.2005.03.285)
567. 0.02 Rassweiler,, JJ (2012) [ipad-assisted percutaneous access to the kidney using marker-based navigation: initial clinical experience](http://dx.doi.org/10.1016/j.eururo.2011.12.024)
568. 0.02 Rolland,, J (2002) [head-mounted projective displays for creating distributed collaborative environments](http://dx.doi.org/10.1117/12.478889)
569. 0.02 Rolland,, J (2002) merging augmented reality and anatomicaly correct 3d models in the development of a training tool for endotracheal intubation
570. 0.02 Rosenthal,, M (2002) [augmented reality guidance for needle biopsies: an initial randomized, controlled trial in phantoms](http://dx.doi.org/10.1016/S1361-8415(02)00088-9)
571. 0.02 Sauer,, F (2000) [augmented workspace: designing an ar testbed](http://dx.doi.org/10.1109/ISAR.2000.880922)
572. 0.02 Sauer,, F (2002) [augmented reality visualization in imri operating room: system description and pre-clinical testing](http://dx.doi.org/10.1117/12.466949)
573. 0.02 Sauer,, F (2003) [augmented reality system for ct-guided interventions: system description and initial phantom trials](http://dx.doi.org/10.1117/12.480383)
574. 0.02 Sauer,, F (2005) [image registration: enabling technology for image guided surgery and therapy](http://dx.doi.org/10.1109/IEMBS.2005.1616182)
575. 0.02 Schwald,, B (2002) [a flexible tracking concept applied to medical scenarios using an ar window](http://dx.doi.org/10.1109/ISMAR.2002.1115102)
576. 0.02 Soler,, L (2010) [computer-assisted digestive surgery](http://dx.doi.org/10.1007/978-1-4419-1123-0_8)
577. 0.02 Spallek,, H (2010) [paradigm shift or annoying distraction emerging implications of web 2.0 for clinical practice](http://dx.doi.org/10.4338/ACI-2010-01-CR-0003)
578. 0.02 State,, A (2001) [dynamic virtual convergence for video see-through head-mounted displays: maintaining maximum stereo overlap throughout a close-range work space](http://dx.doi.org/10.1109/ISAR.2001.970523)
579. 0.02 Tran,, HH (2011) augmented reality system for oral surgery using 3d auto stereoscopic visualization
580. 0.02 Vaissie,, L (1999) [analysis of eyepoint locations and accuracy of rendered depth in binocular head-mounted displays](http://dx.doi.org/10.1117/12.349415)
581. 0.02 Vosburgh,, KG (2007) natural orifice transluminal endoscopic surgery (notes): an opportunity for augmented reality guidance
582. 0.02 Wacker,, FK (2005) [mr image-guided needle biopsies with a combination of augmented reality and mri: a pilot study in phantoms and animals](http://dx.doi.org/10.1016/j.ics.2005.03.300)
583. 0.02 Wagner,, A (1999) [clinical experience with interactive teleconsultation and teleassistance in craniomaxillofacial surgical procedures](http://dx.doi.org/10.1016/S0278-2391(99)90722-X)
584. 0.02 Weidert,, S (2012) [intraoperative augmented reality visualization. current state of development and initial experiences with the camc](http://dx.doi.org/10.1007/s00113-011-2121-8)
585. 0.12 Bacca,, J (2014) augmented reality trends in education: a systematic review of research and applications
586. 0.08 Fombona,, J (2015) mobile augmented reality interaction: an approach to the phenomenon
587. 0.06 Botella,, C (2010) [treating cockroach phobia with augmented reality](http://dx.doi.org/10.1016/j.beth.2009.07.002)
588. 0.06 Chen,, CM (2012) [interactive augmented reality system for enhancing library instruction in elementary schools](http://dx.doi.org/10.1016/j.compedu.2012.03.001)
589. 0.06 Cheng,, KH (2014) [children and parents' reading of an augmented reality picture book: analyses of behavioral patterns and cognitive attainment](http://dx.doi.org/10.1016/j.compedu.2013.12.003)
590. 0.06 Kose,, U (2013) [an augmented reality based mobile software to support learning experiences in computer science courses](http://dx.doi.org/10.1016/j.procs.2013.11.045)
591. 0.06 Sommerauer,, P (2014) [augmented reality in informal learning environments: a field experiment in a mathematics exhibition](http://dx.doi.org/10.1016/j.compedu.2014.07.013)
592. 0.04 Almenara,, JC (2016) productions of learning objects production in agumented reality: the experience of sav of the university of seville
593. 0.04 Bower,, M (2013) augmented reality in education - cases, places, and potentials
594. 0.04 Breton-Lopez,, J (2010) [an augmented reality system validation for the treatment of cockroach phobia](http://dx.doi.org/10.1089/cyber.2009.0170)
595. 0.04 Camba,, J (2014) desktop vs. mobile: a comparative study of augmented reality systems for engineering visualizations in education
596. 0.04 Chen,, DR (2013) [developing a mobile learning system in augmented reality context](http://dx.doi.org/10.1155/2013/594627)
597. 0.04 Fleck,, S (2013) an augmented reality environment for astronomy learning in elementary grades: an exploratory study
598. 0.04 Iordache,, DD (2012) influence of specific ar capabilities on the learning effectiveness and efficiency
599. 0.04 Lin,, TJ (2013) [an investigation of learners' collaborative knowledge construction performances and behavior patterns in an augmented reality simulation system](http://dx.doi.org/10.1016/j.compedu.2013.05.011)
600. 0.04 Martin-Gutierrez,, J (2011) proposal of methodology for learning of standard mechanical elements using augmented reality
601. 0.04 Mathews,, JM (2010) augmented reality gaming and game design as a new literacy practice
602. 0.04 Nincarean,, D (2013) [mobile augmented reality: the potential for education](http://dx.doi.org/10.1016/j.sbspro.2013.10.385)
603. 0.04 Salmi,, H (2012) [towards an open learning environment via augmented reality (ar): visualising the invisible in science centres and schools for teacher education](http://dx.doi.org/10.1016/j.sbspro.2012.06.565)
604. 0.04 Yoon,, SA (2013) [scaffolding informal learning in science museums: how much is too much?](http://dx.doi.org/10.1002/sce.21079)
605. 0.04 Yu,, DG (2010) [a useful visualization technique: a literature review for augmented reality and its application, limitation & future direction](http://dx.doi.org/10.1007/978-1-4419-0312-9_21)
606. 0.04 Zhang,, J (2015) [using augmented reality to promote homogeneity in learning achievement](http://dx.doi.org/10.1109/ISMAR-MASHD.2015.17)
607. 0.02 Abd, Majid, NA (2013) application of mobile augmented reality in a computer science course
608. 0.02 Agusta,, GM (2012) qr code augmented reality tracking with merging on conventional marker based backpropagation neural network
609. 0.02 Akaike,, Y (2014) [ar go-kon: a system for facilitating a smooth communication in the first meeting](http://dx.doi.org/10.1109/UIC-ATC-ScalCom.2014.130)
610. 0.02 Al-Khalifa,, AS (2012) [developing interactive quizzes using layar (tm) augmented reality: lessons learned](http://dx.doi.org/10.1109/NGMAST.2012.16)
611. 0.02 Alrashidi,, M (2013) [viewpoint: an augmented reality tool for viewing and understanding deep technology](http://dx.doi.org/10.3233/978-1-61499-286-8-252)
612. 0.02 Alrashidi,, M (2014) [an object-oriented pedagogical model for mixed reality teaching and learning](http://dx.doi.org/10.1109/IE.2014.37)
613. 0.02 Antonaci,, A (2015) [towards design patterns for augmented reality serious games](http://dx.doi.org/10.1007/978-3-319-25684-9_20)
614. 0.02 Bacca,, J (2015) [mobile augmented reality in vocational education and training](http://dx.doi.org/10.1016/j.procs.2015.12.203)
615. 0.02 Cadavieco,, JF (2013) [geolocation for interactive mobile devices, a new relationship between people and things](http://dx.doi.org/10.5209/rev_HICS.2013.v18.44007)
616. 0.02 Cai,, S (2014) [a case study of augmented reality simulation system application in a chemistry course](http://dx.doi.org/10.1016/j.chb.2014.04.018)
617. 0.02 Camba,, JD (2016) application of low-cost 3d scanning technologies to the development of educational augmented reality content
618. 0.02 Carmigniani,, J (2011) [augmented reality technologies, systems and applications](http://dx.doi.org/10.1007/s11042-010-0660-6)
619. 0.02 Carmigniani,, J (2011) [augmented reality: an overview](http://dx.doi.org/10.1007/978-1-4614-0064-6_1)
620. 0.02 Carter,, BW (2013) digital humanities: current perspective, practices, and research
621. 0.02 Castillo,, RIB (2015) [a pilot study on the use of mobile augmented reality for interactive experimentation in quadratic equations](http://dx.doi.org/10.1155/2015/946034)
622. 0.02 Chang,, HY (2013) [integrating a mobile augmented reality activity to contextualize student learning of a socioscientific issue](http://dx.doi.org/10.1111/j.1467-8535.2012.01379.x)
623. 0.02 Cifuentes,, SC (2016) [augmented reality experiences in therapeutic pedagogy: a study with special needs students](http://dx.doi.org/10.1109/ICALT.2016.23)
624. 0.02 Contero,, M (2012) development of an augmented reality based remedial course to improve the spatial ability of engineering students
625. 0.02 Dede,, C (2011) emerging technologies, ubiquitous learning, and educational transformation
626. 0.02 Delic,, A (2014) augeo: a geolocation-based augmented reality application for vocational geodesy education
627. 0.02 Diaz,, DMS (2015) creating educational content with augmented reality applying principles of the cognitive theory of multimedia learning comparative study to teach how to fly a drone (quadcopter)
628. 0.02 Diaz,, VM (2016) possibilities of use of augmented reality in inclusive education. case study
629. 0.02 Fonseca,, AM (2013) the potential of miio with augmented reality in genetics teaching
630. 0.02 Fonseca,, D (2014) engineering teaching methods using hybrid technologies based on the motivation and assessment of student's profiles
631. 0.02 Fonseca,, D (2014) motivation assessment in engineering students using hybrid technologies for 3d visualization
632. 0.02 Gimeno,, J (2011) multiuser augmented reality system for indoor exhibitions
633. 0.02 Gomez,, JE (2015) interactive architecture as support of situated learning for teaching of engineering
634. 0.02 Gonzalez-Rogado,, AB (2013) [augmented safety in the laboratory with mobile technology](http://dx.doi.org/10.1145/2536536.2536601)
635. 0.02 Gopalan,, V (2015) evaluation of e-star: an enhanced science textbook using augmented reality among lower secondary school students
636. 0.02 Guttentag,, DA (2010) [virtual reality: applications and implications for tourism](http://dx.doi.org/10.1016/j.tourman.2009.07.003)
637. 0.02 Hobert,, S (2015) supporting situated learning on the job in industrial production facilities using augmented reality learning on wearable computers
638. 0.02 Holotescu,, C (2013) educational augmented reality and location-based applications. case study: microblogging
639. 0.02 Hsu,, CH (2012) augmented reality on a dot matrix hologram
640. 0.02 Huang,, YT (2011) [augmented reality in exhibition and entertainment for the public](http://dx.doi.org/10.1007/978-1-4614-0064-6_32)
641. 0.02 Ibanez,, MB (2014) [experimenting with electromagnetism using augmented reality: impact on flow student experience and educational effectiveness](http://dx.doi.org/10.1016/j.compedu.2013.09.004)
642. 0.02 Juarez-Urquijo,, F (2013) [paper and its multimedia possibilities in the library](http://dx.doi.org/10.3145/epi.2013.ene.08)
643. 0.02 Jurane,, I (2015) [opportunities of graphical education's improvement using computer game in distance education](http://dx.doi.org/10.17770/sie2015vol4.401)
644. 0.02 Kawai,, J (2015) [tsunami evacuation drill system using smart glasses](http://dx.doi.org/10.1016/j.procs.2015.12.147)
645. 0.02 Kirner,, C (2013) development of online educational games with augmented reality
646. 0.02 Koutromanos,, G (2015) "the buildings speak about our city": a location based augmented reality game
647. 0.02 Koutromanos,, G (2015) [the use of augmented reality games in education: a review of the literature](http://dx.doi.org/10.1080/09523987.2015.1125988)
648. 0.02 Kucuk,, S (2014) augmented reality applications attitude scale in secondary schools: validity and reliability study
649. 0.02 Kucuk,, S (2014) augmented reality for learning english: achievement, attitude and cognitive load levels of students
650. 0.02 Liao,, T (2012) a framework for debating augmented futures: classifying the visions, promises and ideographs advanced about augmented reality
651. 0.02 Lorenzo,, G (2011) immersive learning using virtual reality and augmented reality based on visual servoing: application to students with special educational needs
652. 0.02 Mahmoudi,, MT (2015) assessing the role of ar-based content in improving learning performance considering felder-silverman learning style
653. 0.02 Martin-Gutierrez,, J (2011) mixed reality for development of spatial skills of first-year engineering students
654. 0.02 Martinez,, NMM (2016) a formative experience in reality augmented with students of master's in secondary education teacher training at the university of malaga
655. 0.02 Martinez,, NMM (2016) robotics, 3d modeling and augmented reality in education for development of multiple intelligences
656. 0.02 Martinez-Grana,, AM (2014) [3d virtual itinerary for education using google earth as a tool for the recovery of the geological heritage of natural areas: application in the "las batuecas valley" nature park (salamanca, spain)](http://dx.doi.org/10.3390/su6128567)
657. 0.02 Metcalf,, D (2011) [fundamental design elements of pervasive games for blended learning](http://dx.doi.org/10.4018/978-1-60960-479-0.ch009)
658. 0.02 Murillo,, AR (2014) improving kinematic lab practices by image processing
659. 0.02 Navarro,, I (2013) [augmented reality uses in educational research projects: the "falcones project", a case study applying technology in the humanities framework at high school level](http://dx.doi.org/10.1145/2536536.2536599)
660. 0.02 Nifakos,, S (2014) [combining physical and virtual contexts through augmented reality: design and evaluation of a prototype using a drug box as a marker for antibiotic training](http://dx.doi.org/10.7717/peerj.697)
661. 0.02 OShea,, P (2011) lessons learned about designing augmented realities
662. 0.02 Panadero,, CF (2014) phymel-ws: physically experiencing the virtual world. insights into mixed reality and flow state on board a wheelchair simulator
663. 0.02 Petrucco,, C (2015) walled cities of veneto region: promoting cultural heritage in education using augmented reality tools
664. 0.02 Quintero,, E (2015) [augmented reality app for calculus: a proposal for the development of spatial visualization](http://dx.doi.org/10.1016/j.procs.2015.12.251)
665. 0.02 Redondo,, E (2013) [new strategies using handheld augmented reality and mobile learning-teaching methodologies, in architecture and building engineering degrees](http://dx.doi.org/10.1016/j.procs.2013.11.007)
666. 0.02 Riera,, AS (2013) [construction processes using mobile augmented reality: a study case in building engineering degree](http://dx.doi.org/10.1007/978-3-642-36981-0_100)
667. 0.02 Squire,, K (2010) from information to experience: place-based augmented reality games as a model for learning in a globally networked society
668. 0.02 Tan,, CT (2011) augmented reality games: a review
669. 0.02 Tobar-Munoz,, H (2014) using a videogame with augmented reality for an inclusive logical skills learning session
670. 0.02 Tsang,, AHC (2011) a context-aware and reality-based learning system
671. 0.02 Valentini,, PP (2010) virtual engineering in augmented reality
672. 0.02 Wang,, CH (2012) applying augmented reality in teaching fundamental earth science in junior high schools
673. 0.02 Wu,, HK (2013) [current status, opportunities and challenges of augmented reality in education](http://dx.doi.org/10.1016/j.compedu.2012.10.024)
674. 0.02 Yamaguchi,, S (2011) intuitive surgical navigation system for dental implantology by using retinal imaging display
675. 0.02 Yen,, JC (2013) [augmented reality in the higher education: students' science concept learning and academic achievement in astronomy](http://dx.doi.org/10.1016/j.sbspro.2013.10.322)
676. 0.02 Yusoff,, Z (2013) mobile based learning: an integrated framework to support learning engagement through augmented reality environment
677. 0.02 Yuviler-Gavish,, N (2011) [learning in multimodal training: visual guidance can be both appealing and disadvantageous in spatial tasks](http://dx.doi.org/10.1016/j.ijhcs.2010.11.005)
678. 0.02 Zaman,, HB (2013) evaluation of augmented reality remedial worksheet based on avctp algorithm for negative numbers (ar(2)wn(2))
679. 0.02 Zhang,, J (2014) [the development and evaluation of an augmented reality-based armillary sphere for astronomical observation instruction](http://dx.doi.org/10.1016/j.compedu.2014.01.003)
680. 0.02 Zhang,, J (2014) uare: using reality-virtually-reality (rvr) models to construct ubiquitous ar environment for e-learning context
681. 0.02 [Anonymous] (2013) [special issue on the applications of augmented reality in architecture, engineering, and construction preface](http://dx.doi.org/10.1016/j.autcon.2013.05.003)
682. 0.15 Portales,, C (2010) [augmented reality and photogrammetry: a synergy to visualize physical and virtual city environments](http://dx.doi.org/10.1016/j.isprsjprs.2009.10.001)
683. 0.15 Yu,, DG (2010) [a useful visualization technique: a literature review for augmented reality and its application, limitation & future direction](http://dx.doi.org/10.1007/978-1-4419-0312-9_21)
684. 0.1 Bruno,, F (2010) mixed prototyping for products usability evaluation
685. 0.1 Bruno,, F (2010) product behaviour simulation in mixed reality
686. 0.1 Caruso,, G (2010) [interactive augmented reality system for product design review](http://dx.doi.org/10.1117/12.840261)
687. 0.1 Santana-Fernandez,, J (2010) [design and implementation of a gps guidance system for agricultural tractors using augmented reality technology](http://dx.doi.org/10.3390/s101110435)
688. 0.08 Vlada,, M (2010) the potential of collaborative augmented reality in education
689. 0.06 Lee,, JY (2010) [hand gesture-based tangible interactions for manipulating virtual objects in a mixed reality environment](http://dx.doi.org/10.1007/s00170-010-2671-x)
690. 0.06 Lee,, S (2010) effects of viewing conditions and rotation methods in a collaborative tabletop ar environment
691. 0.06 Valentini,, PP (2010) virtual engineering in augmented reality
692. 0.04 Botella,, C (2010) [treating cockroach phobia with augmented reality](http://dx.doi.org/10.1016/j.beth.2009.07.002)
693. 0.04 Breton-Lopez,, J (2010) [an augmented reality system validation for the treatment of cockroach phobia](http://dx.doi.org/10.1089/cyber.2009.0170)
694. 0.04 Corradini,, A (2010) natural multimodal interaction in collaborative visualization
695. 0.04 Dong,, SY (2010) [robust mobile computing framework for visualization of simulated processes in augmented reality](http://dx.doi.org/10.1109/WSC.2010.5679004)
696. 0.04 Gu,, N (2010) applying augmented reality for data interaction and collaboration in bim
697. 0.04 Januszka,, M (2010) [augmented reality for machinery systems design and development](http://dx.doi.org/10.1007/978-0-85729-024-3_10)
698. 0.04 Juan,, MC (2010) [using augmented and virtual reality for the development of acrophobic scenarios. comparison of the levels of presence and anxiety](http://dx.doi.org/10.1016/j.cag.2010.08.001)
699. 0.04 Kim,, S (2010) [ar interfacing with prototype 3d applications based on user-centered interactivity](http://dx.doi.org/10.1016/j.cad.2008.10.009)
700. 0.04 Kroeker,, KL (2010) [mainstreaming augmented reality](http://dx.doi.org/10.1145/1785414.1785422)
701. 0.04 Martin-Gutierrez,, J (2010) [design and validation of an augmented book for spatial abilities development in engineering students](http://dx.doi.org/10.1016/j.cag.2009.11.003)
702. 0.04 Ong,, SK (2011) [augmented reality in assistive technology and rehabilitation engineering](http://dx.doi.org/10.1007/978-1-4614-0064-6_28)
703. 0.04 Portales,, C (2010) [ar-immersive cinema at the aula natura visitors center](http://dx.doi.org/10.1109/MMUL.2010.72)
704. 0.04 Riva,, G (2010) [interreality in practice: bridging virtual and real worlds in the treatment of posttraumatic stress disorders](http://dx.doi.org/10.1089/cyber.2009.0320)
705. 0.04 Schnadelbach,, H (2010) [embedded mixed reality environments](http://dx.doi.org/10.1007/978-1-84882-733-2_4)
706. 0.04 Tedjokusumo,, J (2010) [immersive multiplayer games with tangible and physical interaction](http://dx.doi.org/10.1109/TSMCA.2009.2028432)
707. 0.04 Zhu,, JJ (2010) [handling occlusions in video-based augmented reality using depth information](http://dx.doi.org/10.1002/cav.326)
708. 0.02 Aleotti,, J (2010) object manipulation in visuo-haptic augmented reality with physics-based animation
709. 0.02 Ashdown,, M (2003) the escritoire: a personal projected display
710. 0.02 Audet,, S (2010) [direct image alignment of projector-camera systems with planar surfaces](http://dx.doi.org/10.1109/CVPR.2010.5540199)
711. 0.02 Baumeister,, J (2017) [cognitive cost of using augmented reality displays](http://dx.doi.org/10.1109/TVCG.2017.2735098)
712. 0.02 Behzadan,, AH (2010) [scalable algorithm for resolving incorrect occlusion in dynamic augmented reality engineering environments](http://dx.doi.org/10.1111/j.1467-8667.2009.00601.x)
713. 0.02 Bin, Tomi, A (2011) a conceptual design for augmented reality games using motion detection as user interface and interaction
714. 0.02 Botella,, C (2011) [treating cockroach phobia using a serious game on a mobile phone and augmented reality exposure: a single case study](http://dx.doi.org/10.1016/j.chb.2010.07.043)
715. 0.02 Canepa-Talamas,, D (2017) [innovative framework for immersive metrology](http://dx.doi.org/10.1016/j.procir.2017.02.028)
716. 0.02 Caruso,, G (2010) ar-mote: a wireless device for augmented reality environment
717. 0.02 Chang,, MML (2017) [ar-guided product disassembly for maintenance and remanufacturing](http://dx.doi.org/10.1016/j.procir.2016.11.194)
718. 0.02 Chang,, YJ (2011) investigating students perceived satisfaction, behavioral intention, and effectiveness of english learning using augmented reality
719. 0.02 Chen,, SN (2010) implementation and application of an augmented reality based ecology e-learning platform
720. 0.02 Cheok,, AD (2002) [touch-space: mixed reality game space based on ubiquitous, tangible, and social computing](http://dx.doi.org/10.1007/s007790200047)
721. 0.02 Chimienti,, V (2010) guidelines for implementing augmented reality procedures in assisting assembly operations
722. 0.02 Cobzas,, D (2010) [wavelet-based inverse light and reflectance from images of a known object](http://dx.doi.org/10.1007/978-3-642-12392-4_10)
723. 0.02 Darling,, BA (2010) [tangible display systems: direct interfaces for computer-based studies of surface appearance](http://dx.doi.org/10.1117/12.845182)
724. 0.02 De, Lucia, A (2010) smartbuilding: a people-to-people-to-geographical-places mobile system based on augmented reality
725. 0.02 De, Marchi, L (2013) [augmented reality to support on-field post-impact maintenance operations on thin structures](http://dx.doi.org/10.1155/2013/619570)
726. 0.02 Dominguez,, ER (2010) investigation case: the recovery of gerona jew neighbourhood road section using augmented reality
727. 0.02 Duarte,, E (2010) [virtual reality and its potential for evaluating warning compliance](http://dx.doi.org/10.1002/hfm.20242)
728. 0.02 Erkoyuncu,, JA (2017) [improving efficiency of industrial maintenance with context aware adaptive authoring in augmented reality](http://dx.doi.org/10.1016/j.cirp.2017.04.006)
729. 0.02 Evans,, G (2017) [evaluating the microsoft hololens through an augmented reality assembly application](http://dx.doi.org/10.1117/12.2262626)
730. 0.02 Furtado,, H (2010) [a system for visualization and automatic placement of the endoclamp balloon catheter](http://dx.doi.org/10.1117/12.844100)
731. 0.02 Gabbard,, JL (2010) [more than meets the eye: an engineering study to empirically examine the blending of real and virtual color spaces](http://dx.doi.org/10.1109/VR.2010.5444808)
732. 0.02 Galambos,, P (2012) virca net: a case study for collaboration in shared virtual space
733. 0.02 Gao,, QK (2016) [monocular 3d see-through head-mounted display via complex amplitude modulation](http://dx.doi.org/10.1364/OE.24.017372)
734. 0.02 Gimeno,, J (2010) a new approach to the management of the setting out in construction based on augmented reality techniques
735. 0.02 Guan,, T (2010) [recovering pose and occlusion consistencies in augmented reality systems using affine properties](http://dx.doi.org/10.1108/02602281011022751)
736. 0.02 Guttentag,, DA (2010) [virtual reality: applications and implications for tourism](http://dx.doi.org/10.1016/j.tourman.2009.07.003)
737. 0.02 Hachaj,, T (2010) augmented reality interface for visualization of volumetric medical data
738. 0.02 Henderson,, SJ (2011) augmented reality in the psychomotor phase of a procedural task
739. 0.02 Holm,, M (2017) [adaptive instructions to novice shop-floor operators using augmented reality](http://dx.doi.org/10.1080/21681015.2017.1320592)
740. 0.02 Hong,, K (2014) [two-dimensional and three-dimensional transparent screens based on lens-array holographic optical elements](http://dx.doi.org/10.1364/OE.22.014363)
741. 0.02 Horvath,, S (2014) [refocusing a scanned laser projector for small and bright images: simultaneously controlling the profile of the laser beam and the boundary of the image](http://dx.doi.org/10.1364/AO.53.005421)
742. 0.02 Hou,, L (2017) [a framework of innovative learning for skill development in complex operational tasks](http://dx.doi.org/10.1016/j.autcon.2017.07.001)
743. 0.02 Hua,, H (2014) [past and future of wearable augmented reality displays and their applications](http://dx.doi.org/10.1117/12.2063946)
744. 0.02 Huang,, JY (2010) the research of a multiplayer mobile augmented reality (mimar) system and its applications
745. 0.02 Inami,, M (2010) [active tangible interactions](http://dx.doi.org/10.1007/978-1-84996-113-4_8)
746. 0.02 Kim,, SK (2017) [augmented-reality survey: from concept to application](http://dx.doi.org/10.3837/tiis.2017.02.019)
747. 0.02 Knorlein,, B (2010) enhanced visual depth cues for collocated visuo-haptic augmented reality
748. 0.02 Lamberti,, F (2014) [challenges, opportunities, and future trends of emerging techniques for augmented reality-based maintenance](http://dx.doi.org/10.1109/TETC.2014.2368833)
749. 0.02 Lei,, J (2011) real-time object tracking on mobile phones
750. 0.02 Lin,, CY (2010) [study on augmented reality as a teaching aid for handicapped children](http://dx.doi.org/10.4028/www.scientific.net/KEM.439-440.1253)
751. 0.02 Lin,, FY (2010) augmented reality with human body interaction based on monocular 3d pose estimation
752. 0.02 Liu,, YL (2010) [a new approach to outdoor illumination estimation based on statistical analysis for augmented reality](http://dx.doi.org/10.1002/cav.357)
753. 0.02 Livatino,, S (2010) augmented reality stereoscopic visualization for intuitive robot teleguide
754. 0.02 Manders,, C (2010) [a gesture control system for intuitive 3d interaction with virtual objects](http://dx.doi.org/10.1002/cav.324)
755. 0.02 Marner,, MR (2010) augmented foam sculpting for capturing 3d models
756. 0.02 Martin-Gutierrez,, J (2010) evaluating the usability of an augmented reality based educational application
757. 0.02 Martins,, VF (2013) usability test for augmented reality applications
758. 0.02 Murphy,, DJ (2010) [an augmented reality view on mirror world content, with image space](http://dx.doi.org/10.1109/VR.2010.5444761)
759. 0.02 Nilsson,, S (2010) [a holistic approach to design and evaluation of mixed reality systems](http://dx.doi.org/10.1007/978-1-84882-733-2_3)
760. 0.02 Olivieri,, D (2011) ar-based virtual musical instruments using smc tracking
761. 0.02 Olsson,, T (2011) online user survey on current mobile augmented reality applications
762. 0.02 Ong,, SK (2011) [augmented reality in product development and manufacturing](http://dx.doi.org/10.1007/978-1-4614-0064-6_30)
763. 0.02 Ono,, Y (2010) [relative posture estimation using high frequency markers](http://dx.doi.org/10.1109/IROS.2010.5652134)
764. 0.02 Placitelli,, AP (2011) 3d point cloud sensors for low-cost medical in-situ visualization
765. 0.02 Rakkolainen,, I (2010) feasible mid-air virtual reality with the immaterial projection screen technology
766. 0.02 Scheggi,, S (2010) shape and weight rendering for haptic augmented reality
767. 0.02 Schmalstieg,, D (2002) [the studierstube augmented reality project](http://dx.doi.org/10.1162/105474602317343640)
768. 0.02 Shen,, Y (2010) [augmented reality for collaborative product design and development](http://dx.doi.org/10.1016/j.destud.2009.11.001)
769. 0.02 Sherstyuk,, A (2010) [toward natural selection in virtual reality](http://dx.doi.org/10.1109/MCG.2010.34)
770. 0.02 Shin,, DH (2010) [technology development needs for advancing augmented reality-based inspection](http://dx.doi.org/10.1016/j.autcon.2009.11.001)
771. 0.02 Syberfeldt,, A (2017) [augmented reality smart glasses in the smart factory: product evaluation guidelines and review of available products](http://dx.doi.org/10.1109/ACCESS.2017.2703952)
772. 0.02 Valentini,, PP (2010) interactive multibody simulation in augmented reality
773. 0.02 Vasilijevic,, A (2011) augmented reality in marine applications
774. 0.02 Vidal,, NR (2010) [augmented reality systems for weed economic thresholds applications](http://dx.doi.org/10.1590/S0100-83582010000200025)
775. 0.02 Wang,, XY (2011) [comparative effectiveness of mixed reality-based virtual environments in collaborative design](http://dx.doi.org/10.1109/TSMCC.2010.2093573)
776. 0.02 Wegerich,, A (2010) a context-aware adaptation system for spatial augmented reality projections
777. 0.02 Wetzstein,, G (2010) [optical image processing using light modulation displays](http://dx.doi.org/10.1111/j.1467-8659.2010.01660.x)
778. 0.02 Wrzesien,, M (2011) how technology influences the therapeutic process: a comparative field evaluation of augmented reality and in vivo exposure therapy for phobia of small animals
779. 0.02 Yin,, XY (2014) [vr&ar combined manual operation instruction system on industry products: a case study](http://dx.doi.org/10.1109/ICVRV.2014.55)
780. 0.02 Zhang,, J (2010) [a multi-regional computation scheme in an ar-assisted in situ cnc simulation environment](http://dx.doi.org/10.1016/j.cad.2010.06.007)
781. 0.02 Zheng,, ZR (2010) [design and fabrication of an off-axis see-through head-mounted display with an x-y polynomial surface](http://dx.doi.org/10.1364/AO.49.003661)
782. 0.02 Zhu,, ZW (2014) ar-mentor: augmented reality based mentoring system
783. 0.02 [Anonymous] (2010) [human pacman: a mobile augmented reality entertainment system based on physical, social, and ubiquitous computing](http://dx.doi.org/10.1007/978-1-84996-137-0_2)
784. 0.02 [Anonymous] (2013) [special issue on the applications of augmented reality in architecture, engineering, and construction preface](http://dx.doi.org/10.1016/j.autcon.2013.05.003)
785. 0.02 de, Lima, ES (2011) draw your own story: paper and pencil interactive storytelling
786. 1 FEINER,, S (1993) [knowledge-based augmented reality](http://dx.doi.org/10.1145/159544.159587)
787. 0.68 Lu,, CP (1996) [online computation of exterior orientation with application to hand-eye calibration](http://dx.doi.org/10.1016/0895-7177(96)00118-5)
788. 0.11 Yokoya,, N (1999) stereo vision based video see-through mixed reality
789. 0.05 BAJURA,, M (1995) [dynamic registration correction in video-based augmented reality systems](http://dx.doi.org/10.1109/38.403828)
790. 0.05 Billinghurst,, M (1999) collaborative mixed reality
791. 0.05 Hoff,, W (2000) [analysis of head pose accuracy in augmented reality](http://dx.doi.org/10.1109/2945.895877)
792. 0.05 Kanbara,, M (1999) real-time composition of stereo images for video see-through augmented reality
793. 0.05 Molineros,, J (1999) areas: augmented reality for evaluating assembly sequences
794. 0.05 Sato,, I (1999) [acquiring a radiance distribution to superimpose virtual objects onto a real scene](http://dx.doi.org/10.1109/2945.764865)
795. 0.05 Yokoya,, N (1999) passive range sensing techniques: depth from images
796. 0.22 Pulli,, P (1998) cyphone - mobile multimodal personal augmented reality
797. 0.22 Pulli,, P (1998) cyphone - mobile telepresence and augmented reality for 3rd generation cellular phone
798. 0.11 Baber,, C (1999) [contrasting paradigms for the development of wearable computers](http://dx.doi.org/10.1147/sj.384.0551)
799. 0.11 Billinghurst,, M (1999) collaborative mixed reality
800. 0.11 Bruns,, WF (1999) complex construction kits for coupled real and virtual engineering workspaces
801. 0.11 Feiner,, S (1999) wearing it out: first steps toward mobile augmented reality systems
802. 0.11 Spohrer,, JC (1999) [information in places](http://dx.doi.org/10.1147/sj.384.0602)
803. 0.11 Tarumi,, H (1999) spacetag: an overlaid virtual system and its applications
804. 0.06 AHLERS,, KH (1995) [distributed augmented reality for collaborative design applications](http://dx.doi.org/10.1111/1467-8659.1430003)
805. 0.06 Azuma,, RT (1997) [a survey of augmented reality](http://dx.doi.org/10.1162/pres.1997.6.4.355)
806. 0.06 Berger,, JW (1999) [computer-vision-enabled augmented reality fundus biomicroscopy](http://dx.doi.org/10.1016/S0161-6420(99)90404-9)
807. 0.06 Berger,, JW (1999) computer-vision-enabled ophthalmic augmented reality: a pc-based prototype
808. 0.06 Berger,, JW (1999) [image-guided macular laser therapy: design considerations and progress towards implementation](http://dx.doi.org/10.1117/12.350586)
809. 0.06 Bimber,, O (2000) augmented reality with back-projection systems using transflective surfaces
810. 0.06 Dubois,, E (1999) classification space for augmented surgery, an augmented reality case study
811. 0.06 Falk,, J (1999) amplifying reality
812. 0.06 Fjeld,, M (1999) exploring brick-based navigation and composition in an augmented reality
813. 0.06 Fuhrmann,, A (1998) [collaborative visualization in augmented reality](http://dx.doi.org/10.1109/38.689665)
814. 0.06 Fuhrmann,, A (1999) [occlusion in collaborative augmented environments](http://dx.doi.org/10.1016/S0097-8493(99)00107-7)
815. 0.06 Fuhrmann,, A (2000) practical calibration procedures for augmented reality
816. 0.06 Jang,, B (1999) an outdoor augmented reality system for gis applications
817. 0.06 Julier,, S (2000) [information filtering for mobile augmented reality](http://dx.doi.org/10.1109/ISAR.2000.880917)
818. 0.06 Kim,, H (1999) [a birds-eye view system using augmented reality](http://dx.doi.org/10.1109/SIMSYM.1999.766463)
819. 0.06 Kiyokawa,, K (1999) seamlessdesign: a face-to-face collaborative virtual/augmented environment for rapid prototyping of geometrically constrained 3-d objects
820. 0.06 Klinker,, G (1999) augmented reality: a balancing act between high quality and real-time constraints
821. 0.06 Konomi,, S (1999) passage: physical transportation of digital information in cooperative buildings
822. 0.06 MacIntyre,, B (2000) [adapting to dynamic registration errors using level of error (loe) filtering](http://dx.doi.org/10.1109/ISAR.2000.880927)
823. 0.06 Molineros,, J (1999) areas: augmented reality for evaluating assembly sequences
824. 0.06 Pyssysalo,, T (2000) [adaptive streaming protocol for mobile augmented reality-based concurrent engineering](http://dx.doi.org/10.1117/12.384468)
825. 0.06 Reiners,, D (1999) augmented reality for construction tasks: doorlock assembly
826. 0.06 Rekimoto,, J (1997) [navicam: a magnifying glass approach to augmented reality](http://dx.doi.org/10.1162/pres.1997.6.4.399)
827. 0.06 TUCERYAN,, M (1995) [calibration requirements and procedures for a monitor-based augmented reality system](http://dx.doi.org/10.1109/2945.466720)
828. 0.06 Terashima,, M (1999) a human-robot interface using an extended digital desk
829. 0.06 Turunen,, T (2001) performance evaluation of service connectivity in mobile augmented reality
830. 0.06 WHITAKER,, RT (1995) [object calibration for augmented reality](http://dx.doi.org/10.1111/1467-8659.1430015)
831. 0.06 You,, S (1999) [hybrid inertial and vision tracking for augmented reality registration](http://dx.doi.org/10.1109/VR.1999.756960)
832. 0.06 Young,, AL (1999) the potential of augmented reality technology for training support systems.
833. 0.18 Ismail,, AW (2009) [collaborative augmented reality approach for multi-user interaction in urban simulation](http://dx.doi.org/10.1109/ICIMT.2009.68)
834. 0.18 Ismail,, AW (2009) collaborative augmented reality: multi-user interaction in urban simulation
835. 0.18 Ismail,, AW (2009) [multi-user interaction in collaborative augmented reality for urban simulation](http://dx.doi.org/10.1109/ICMV.2009.40)
836. 0.12 Ismail,, AW (2009) [survey on collaborative ar for multi-user in urban studies and planning](http://dx.doi.org/10.1007/978-3-642-03364-3_53)
837. 0.12 Noh,, Z (2009) exploring the potential of using augmented reality approach in cultural heritage system
838. 0.06 Azuma,, R (2001) [recent advances in augmented reality](http://dx.doi.org/10.1109/38.963459)
839. 0.06 Behzadan,, AH (2008) [general-purpose modular hardware and software framework for mobile outdoor augmented reality applications in engineering](http://dx.doi.org/10.1016/j.aei.2007.08.005)
840. 0.06 Corradini,, A (2010) natural multimodal interaction in collaborative visualization
841. 0.06 Filippi,, S (2011) an augmented reality based application for furnishing configuration and evaluation
842. 0.06 Graf,, H (2011) lifecycle building card: toward paperless and visual lifecycle management tools
843. 0.06 Ha,, T (2010) an empirical evaluation of virtual hand techniques for 3d object manipulation in a tangible augmented reality environment
844. 0.06 Hagbi,, N (2011) [shape recognition and pose estimation for mobile augmented reality](http://dx.doi.org/10.1109/TVCG.2010.241)
845. 0.06 Koceski,, S (2011) collaborative augmented reality (ar) mobile phone game for co-located players
846. 0.06 Maamar,, HR (2010) [mosaic - a mobile peer-to-peer networks-based 3d streaming supplying partner protocol](http://dx.doi.org/10.1109/DS-RT.2010.16)
847. 0.06 MacIntyre,, B (2001) [augmented reality as a new media experience](http://dx.doi.org/10.1109/ISAR.2001.970538)
848. 0.06 Martin-Gutierrez,, J (2010) evaluating the usability of an augmented reality based educational application
849. 0.06 Noh,, Z (2009) [a review on shadow techniques in augmented reality](http://dx.doi.org/10.1109/ICMV.2009.41)
850. 0.06 Paelke,, V (2010) [augmented paper maps: exploring the design space of a mixed reality system](http://dx.doi.org/10.1016/j.isprsjprs.2009.05.006)
851. 0.06 Pang,, Y (2006) [assembly feature design in an augmented reality environment](http://dx.doi.org/10.1108/01445150610645648)
852. 0.06 Park,, JS (2011) [ar-room: a rapid prototyping framework for augmented reality applications](http://dx.doi.org/10.1007/s11042-010-0592-1)
853. 0.06 Penn,, A (2004) augmented reality meeting table: a novel multi-user interface for architectural design
854. 0.06 Regenbrecht,, H (2004) [using augmented virtuality for remote collaboration](http://dx.doi.org/10.1162/1054746041422334)
855. 0.06 Seo,, BK (2011) augmented reality-based on-site tour guide: a study in gyeongbokgung
856. 0.06 St-Aubin,, B (2010) a 3d collaborative geospatial augmented reality system for urban design and planning purposes
857. 0.06 Tedjokusumo,, J (2010) [immersive multiplayer games with tangible and physical interaction](http://dx.doi.org/10.1109/TSMCA.2009.2028432)
858. 0.06 Wagner,, D (2010) [real-time detection and tracking for augmented reality on mobile phones](http://dx.doi.org/10.1109/TVCG.2009.99)
859. 0.06 Wither,, J (2004) [evaluating techniques for interaction at a distance](http://dx.doi.org/10.1109/ISWC.2004.18)
860. 0.06 Yu,, DG (2010) [a useful visualization technique: a literature review for augmented reality and its application, limitation & future direction](http://dx.doi.org/10.1007/978-1-4419-0312-9_21)
861. 0.06 [Anonymous] (2010) [human pacman: a mobile augmented reality entertainment system based on physical, social, and ubiquitous computing](http://dx.doi.org/10.1007/978-1-84996-137-0_2)
