# Supplementary material for: The Past, Present, and Future of Virtual and Augmented Reality Research: A Network and Cluster Analysis of the Literature
Source: Front Psychol. 2018 Nov 6;9:2086. doi: 10.3389/fpsyg.2018.02086 (PMC6232426; doi:10.3389/fpsyg.2018.02086)
Supplement: Supplementary file 1 [file Data_Sheet_1.ZIP › Clusters semplificato from citations.docx]

**Cluster summary**

| **ID** | **Size** | **Silho-uette** | **Mean (Citee Year)** | **Label (TFIDF)** | **Label (LLR)** |
| --- | --- | --- | --- | --- | --- |
| 0 | 84 | 0.812 | 2005 | (25.82) laparoscopic skill; (25.01) proficiency; (24.5) basic laparoscopic skill; (24.14) trainer; (23.79) establishing validity | training (143.21, 1.0E-4); performance (73.38, 1.0E-4); laparoscopic skill (72.93, 1.0E-4); |
| 1 | 77 | 0.758 | 1992 | (17.76) ergonomic; (17.66) reality; (16.83) virtual reality; (16.04) virtual environment; (15.76) assembly | ergonomic (54.1, 1.0E-4); virtual reality interface (34.63, 1.0E-4); developing virtual environment (34.48, 1.0E-4); |
| 2 | 62 | 0.992 | 2007 | (24.5) gaming; (24.5) wii; (24.47) stroke; (23.07) rehabilitation; (22.38) cerebral palsy | stroke (82.9, 1.0E-4); children (75.13, 1.0E-4); stroke rehabilitation (57.95, 1.0E-4); |
| 3 | 61 | 0.758 | 1994 | (15) reality; (14.66) virtual reality; (14.25) surgery; (14.1) telemedical information society; (13.73) chemistry | telemedical information society (34.85, 1.0E-4); gaining insight (23.21, 1.0E-4); next decade (18.32, 1.0E-4); |
| 4 | 56 | 0.934 | 2008 | (25.4) therapy; (23.55) exposure therapy; (22.41) disorder; (21.63) virtual reality exposure therapy; (20.99) posttraumatic stress | treatment (109.92, 1.0E-4); posttraumatic stress disorder (78.95, 1.0E-4); virtual reality exposure therapy (66.15, 1.0E-4); |
| 5 | 49 | 0.885 | 1992 | (16.03) reality; (15.31) virtual reality; (15.01) autistic children; (12.79) child; (12.79) children | autistic children (29.81, 1.0E-4); possibilities (23.84, 1.0E-4); communication (22.08, 1.0E-4); |
| 6 | 41 | 0.855 | 1998 | (17.6) laparoscopic skill; (16.95) direct observation; (16.95) measuring operative performance; (16.95) videotape; (16.15) measuring | laparoscopic skills training (52.73, 1.0E-4); measuring operative performance (40.97, 1.0E-4); videotape (40.97, 1.0E-4); |
| 7 | 41 | 0.946 | 1998 | (20.71) therapy; (18.76) exposure therapy; (17.85) exposure; (17.35) anxiety; (17.2) virtual reality exposure therapy | virtual reality exposure therapy (32.01, 1.0E-4); spider phobia (27.67, 1.0E-4); ptsd vietnam veteran (22.12, 1.0E-4); |
| 8 | 38 | 1 | 1989 | (30.67) japanese institutional mechanism; (30.67) systems perspective; (20.88) mechanism; (19.25) perspective; (17.97) system | japanese institutional mechanism (615.45, 1.0E-4); systems perspective (615.45, 1.0E-4); virtual reality (16.28, 1.0E-4); |
| 9 | 21 | 1 | 1987 | (23.27) routine use; (23.27) current application; (23.27) behavioral-assessment; (23.27) obstacle; (23.27) future possibilities | future possibilities (168.77, 1.0E-4); routine use (168.77, 1.0E-4); current application (168.77, 1.0E-4); |
| 10 | 18 | 0.934 | 1991 | (12.45) reality; (12.26) virtual-reality; (9.73) medicine; (9.07) virtual reality; (5.71) technology | virtual-reality (88.95, 1.0E-4); medicine (34.87, 1.0E-4); pretty interface (9.63, 0.005); |
| 11 | 16 | 0.937 | 1990 | (13.37) tutorial; (12.45) reality; (11.98) virtual reality; (11.12) virtual reality technology; (10.78) technology | tutorial (51.15, 1.0E-4); virtual reality technology (44.66, 1.0E-4); space (16.78, 1.0E-4); |
| 12 | 12 | 1 | 1988 | (20.05) special effect; (20.05) cyberspace; (13.65) space; (11.38) effect; (10.73) reality | special effect (128.6, 1.0E-4); cyberspace (128.6, 1.0E-4); virtual reality (27.79, 1.0E-4); |
| 13 | 8 | 0.995 | 1997 | (14.88) neural substrate; (14.88) human spatial navigation; (14.88) cognitive map; (11.56) navigation; (10.64) cognitive | neural substrate (72.6, 1.0E-4); human spatial navigation (66.58, 1.0E-4); cognitive map (66.58, 1.0E-4); |
| 14 | 6 | 0.993 | 2008 | (12.06) neurosurgery; (9.74) computer technology; (9.74) surgical application; (9.43) surgery; (8.55) teaching | neurosurgery (28.72, 1.0E-4); computer technology (18.1, 1.0E-4); surgical application (18.1, 1.0E-4); |

**Cluster 4**

| 4 | 56 | 0.934 | 2008 | (25.4) therapy; (23.55) exposure therapy; (22.41) disorder; (21.63) virtual reality exposure therapy; (20.99) posttraumatic stress | treatment (109.92, 1.0E-4); posttraumatic stress disorder (78.95, 1.0E-4); virtual reality exposure therapy (66.15, 1.0E-4); | approache |
| --- | --- | --- | --- | --- | --- | --- |

| 136 |  | 0.00 | 1.00 | 0.00 |  | Parsons TD | 2008 | ***...*** | J BEHAV THER EXP PSY | V39 | P250 | 1 | 4 |
| --- | --- | --- | --- | --- | --- | --- | --- | --- | --- | --- | --- | --- | --- |
| 134 |  | 0.00 | 1.00 | 0.00 |  | Powers MB | 2008 | ***...*** | J ANXIETY DISORD | V22 | P561 | 1 | 4 |
| 110 |  | 0.00 | 1.00 | 0.00 |  | Sanchez-vives MV | 2005 | ***...*** | NAT REV NEUROSCI | V6 | P332 | 3 | 4 |
| 72 |  | 0.00 | 1.00 | 0.00 |  | Krijn M | 2004 | ***...*** | CLIN PSYCHOL REV | V24 | P259 | 1 | 4 |
| 70 |  | 0.00 | 1.00 | 0.00 |  | Bohil CJ | 2011 | ***...*** | NAT REV NEUROSCI | V12 | P752 | 3 | 4 |
| 68 |  | 0.00 | 1.00 | 0.00 |  | Riva G | 2005 | ***...*** | CYBERPSYCHOL BEHAV | V8 | P220 | 2 | 4 |
| 60 |  | 0.00 | 1.00 | 0.00 |  | Slater M | 2010 | ***...*** | PLOS ONE | V5 | P | 3 | 4 |
| 58 |  | 0.00 | 1.00 | 0.00 |  | Slater M | 2009 | ***...*** | PHILOS T R SOC B | V364 | P3549 | 4 | 4 |
| 57 |  | 0.00 | 1.00 | 0.00 |  | Lenggenhager B | 2007 | ***...*** | SCIENCE | V317 | P1096 | 6 | 4 |
| 55 |  | 0.00 | 1.00 | 0.00 |  | Difede J | 2007 | ***...*** | J CLIN PSYCHIAT | V68 | P1639 | 2 | 4 |
| 54 |  | 0.00 | 1.00 | 0.00 |  | Rothbaum BO | 2006 | ***...*** | BEHAV THER | V37 | P80 | 5 | 4 |
| 54 |  | 0.00 | 1.00 | 0.00 |  | Gregg L | 2007 | ***...*** | SOC PSYCH PSYCH EPID | V42 | P343 | 4 | 4 |
| 50 |  | 0.00 | 1.00 | 0.00 |  | Krijn M | 2004 | ***...*** | BEHAV RES THER | V42 | P229 | 7 | 4 |
| 49 |  | 0.00 | 1.00 | 0.00 |  | Riva G | 2007 | ***...*** | CYBERPSYCHOL BEHAV | V10 | P45 | 8 | 4 |
| 49 |  | 0.00 | 1.00 | 0.00 |  | Hoffman HG | 2004 | ***...*** | NEUROREPORT | V15 | P1245 | 6 | 4 |

1. 0.18 De, Carvalho, MR (2010) [virtual reality as a mechanism for exposure therapy](http://dx.doi.org/10.3109/15622970802575985)
2. 0.16 Meyerbroker,, K (2010) [virtual reality exposure therapy in anxiety disorders: a systematic review of process-and-outcome studies](http://dx.doi.org/10.1002/da.20734)
3. 0.16 Riva,, G (2010) [interreality in practice: bridging virtual and real worlds in the treatment of posttraumatic stress disorders](http://dx.doi.org/10.1089/cyber.2009.0320)
4. 0.14 Rizzo,, A (2010) [development and early evaluation of the virtual iraq/afghanistan exposure therapy system for combat-related ptsd](http://dx.doi.org/10.1111/j.1749-6632.2010.05755.x)
5. 0.12 Gerardi,, M (2010) [virtual reality exposure therapy for post-traumatic stress disorder and other anxiety disorders](http://dx.doi.org/10.1007/s11920-010-0128-4)
6. 0.11 Brinkman,, WP (2010) [the therapist user interface of a virtual reality exposure therapy system in the treatment of fear of flying](http://dx.doi.org/10.1016/j.intcom.2010.03.005)
7. 0.11 Newman,, MG (2010) anxiety disorders
8. 0.11 Rothbaurn,, BO (2010) [virtual reality exposure therapy for combat-related posttraumatic stress disorder](http://dx.doi.org/10.1111/j.1749-6632.2010.05691.x)
9. 0.11 de, la Pena, N (2010) [immersive journalism: immersive virtual reality for the first-person experience of news](http://dx.doi.org/10.1162/PRES_a_00005)
10. 0.09 Slater,, M (2010) [first person experience of body transfer in virtual reality](http://dx.doi.org/10.1371/journal.pone.0010564)
11. 0.07 Botella,, C (2010) [an adaptive display for the treatment of diverse trauma ptsd victims](http://dx.doi.org/10.1089/cyber.2009.0353)
12. 0.07 Cosic,, K (2010) [physiology-driven adaptive virtual reality stimulation for prevention and treatment of stress related disorders](http://dx.doi.org/10.1089/cyber.2009.0260)
13. 0.07 Gonzalez-Franco,, M (2010) the contribution of real-time mirror reflections of motor actions on virtual body ownership in an immersive virtual environment
14. 0.07 Norrholm,, SD (2010) the optimization of research and clinical applications for combat-related posttraumatic stress disorder (ptsd): progress through modern translational methodologies
15. 0.07 Ready,, DJ (2010) [comparing virtual reality exposure therapy to present-centered therapy with 11 us vietnam veterans with ptsd](http://dx.doi.org/10.1089/cyber.2009.0239)
16. 0.05 Alsina-Jurnet,, I (2010) [influence of personality and individual abilities on the sense of presence experienced in anxiety triggering virtual environments](http://dx.doi.org/10.1016/j.ijhcs.2010.07.001)
17. 0.05 Botella,, C (2010) treating cockroach phobia with augmented reality
18. 0.05 Bruck,, S (2010) [accessible virtual reality therapy using portable media devices](http://dx.doi.org/10.3233/978-1-60750-561-7-87)
19. 0.05 Choi,, DC (2010) [pharmacological enhancement of behavioral therapy: focus on posttraumatic stress disorder](http://dx.doi.org/10.1007/7854_2009_10)
20. 0.05 Courtney,, CG (2010) [better than the real thing: eliciting fear with moving and static computer-generated stimuli](http://dx.doi.org/10.1016/j.ijpsycho.2010.06.028)
21. 0.05 Diemer,, J (2010) [therapy-refractory panic: current research areas as possible perspectives in the treatment of anxiety](http://dx.doi.org/10.1007/s00406-010-0143-9)
22. 0.05 Freedman,, SA (2010) [prolonged exposure and virtual reality-enhanced imaginal exposure for ptsd following a terrorist bulldozer attack: a case study](http://dx.doi.org/10.1089/cyber.2009.0271)
23. 0.05 Lambrey,, S (2010) [virtual reality therapies in the treatment of phobic disorders](http://dx.doi.org/10.1016/j.amp.2009.10.003)
24. 0.05 Lau,, WC (2010) [a virtual psychiatric ward for orientating patients admitted for the first time](http://dx.doi.org/10.1089/cyber.2009.0107)
25. 0.05 McLean,, CP (2010) posttraumatic stress disorder
26. 0.05 Rey,, B (2010) [brain activity and presence: a preliminary study in different immersive conditions using transcranial doppler monitoring](http://dx.doi.org/10.1007/s10055-009-0141-2)
27. 0.05 Waterworth,, JA (2010) on feeling (the) present an evolutionary account of the sense of presence in physical and electronically-mediated environments
28. 0.05 Wieser,, MJ (2010) [virtual social interactions in social anxiety-the impact of sex, gaze, and interpersonal distance](http://dx.doi.org/10.1089/cyber.2009.0432)
29. 0.05 Wood,, DP (2010) [cybertherapy for combat related posttraumatic stress disorder, pain management and physical rehabilitation following stroke](http://dx.doi.org/10.3233/978-1-60750-571-6-169)
30. 0.05 Wood,, DP (2010) [lessons learned from 350 virtual-reality sessions with warriors diagnosed with combat-related posttraumatic stress disorder](http://dx.doi.org/10.1089/cyber.2009.0396)
31. 0.05 Young,, G (2010) [virtually real emotions and the paradox of fiction: implications for the use of virtual environments in psychological research](http://dx.doi.org/10.1080/09515080903532274)
32. 0.04 Breton-Lopez,, J (2010) [an augmented reality system validation for the treatment of cockroach phobia](http://dx.doi.org/10.1089/cyber.2009.0170)
33. 0.04 Freeman,, D (2010) [cognitive and social processes in psychosis: recent developments](http://dx.doi.org/10.1007/978-1-4419-0913-8_15)
34. 0.04 Freeman,, D (2010) [testing the continuum of delusional beliefs: an experimental study using virtual reality](http://dx.doi.org/10.1037/a0017514)
35. 0.04 Gamito,, P (2010) [ptsd elderly war veterans: a clinical controlled pilot study](http://dx.doi.org/10.1089/cyber.2009.0237)
36. 0.04 Garcia-Betances,, RI (2015) [a succinct overview of virtual reality technology use in alzheimer's disease](http://dx.doi.org/10.3389/fnagi.2015.00080)
37. 0.04 Goncalves,, R (2012) [efficacy of virtual reality exposure therapy in the treatment of ptsd: a systematic review](http://dx.doi.org/10.1371/journal.pone.0048469)
38. 0.04 Gorini,, A (2010) [assessment of the emotional responses produced by exposure to real food, virtual food and photographs of food in patients affected by eating disorders](http://dx.doi.org/10.1186/1744-859X-9-30)
39. 0.04 Jackson,, RE (2010) [reducing the presence of navigation risk eliminates strong environmental illusions](http://dx.doi.org/10.1167/10.5.9)
40. 0.04 Juan,, MC (2010) [using augmented and virtual reality for the development of acrophobic scenarios. comparison of the levels of presence and anxiety](http://dx.doi.org/10.1016/j.cag.2010.08.001)
41. 0.04 Kannape,, OA (2010) [the limits of agency in walking humans](http://dx.doi.org/10.1016/j.neuropsychologia.2010.02.005)
42. 0.04 Morina,, N (2014) [sense of presence and anxiety during virtual social interactions between a human and virtual humans](http://dx.doi.org/10.7717/peerj.337)
43. 0.04 Paping,, C (2010) [an explorative study into a tele-delivered multi-patient virtual reality exposure therapy system](http://dx.doi.org/10.3233/978-1-60750-571-6-203)
44. 0.04 Plancher,, G (2010) [age effect on components of episodic memory and feature binding: a virtual reality study](http://dx.doi.org/10.1037/a0018680)
45. 0.04 Pomes,, A (2013) [drift and ownership toward a distant virtual body](http://dx.doi.org/10.3389/fnhum.2013.00908)
46. 0.04 Rief,, W (2015) [expectancies as core features of mental disorders](http://dx.doi.org/10.1097/YCO.0000000000000184)
47. 0.04 Riva,, G (2012) [virtual reality in the treatment of body image disturbances after bariatric surgery: a clinical case](http://dx.doi.org/10.3233/978-1-61499-121-2-278)
48. 0.04 Spurgeon,, JA (2010) [computer-assisted cognitive-behavioral therapy](http://dx.doi.org/10.1007/s11920-010-0152-4)
49. 0.04 Stinson,, K (2010) [cognitive triggers of auditory hallucinations: an experimental investigation](http://dx.doi.org/10.1016/j.jbtep.2009.12.003)
50. 0.04 Vignais,, N (2010) [influence of the graphical levels of detail of a virtual thrower on the perception of the movement](http://dx.doi.org/10.1162/pres.19.3.243)
51. 0.04 Vignais,, N (2010) [virtual thrower versus real goalkeeper: the influence of different visual conditions on performance](http://dx.doi.org/10.1162/PRES_a_00003)
52. 0.04 Wu,, DR (2010) [optimal arousal identification and classification for affective computing using physiological signals: virtual reality stroop task](http://dx.doi.org/10.1109/T-AFFC.2010.12)
53. 0.02 Anderson,, PL (2013) [virtual reality exposure therapy for social anxiety disorder: a randomized controlled trial](http://dx.doi.org/10.1037/a0033559)
54. 0.02 Andreatta,, PB (2010) [virtual reality triage training provides a viable solution for disaster-preparedness](http://dx.doi.org/10.1111/j.1553-2712.2010.00728.x)
55. 0.02 Annett,, MK (2010) [investigating the application of virtual reality systems to psychology and cognitive neuroscience research](http://dx.doi.org/10.1162/pres.19.2.131)
56. 0.02 Atesok,, K (2012) [surgical simulation in orthopaedic skills training](http://dx.doi.org/10.5435/JAAOS-20-06-410)
57. 0.02 Banakou,, D (2013) [illusory ownership of a virtual child body causes overestimation of object sizes and implicit attitude changes](http://dx.doi.org/10.1073/pnas.1306779110)
58. 0.02 Banos,, RM (2012) [positive mood induction procedures for virtual environments designed for elderly people](http://dx.doi.org/10.1016/j.intcom.2012.04.002)
59. 0.02 Beck,, L (2010) [evaluation of spatial processing in virtual reality using functional magnetic resonance imaging (fmri)](http://dx.doi.org/10.1089/cyber.2008.0343)
60. 0.02 Borckardt,, JJ (2010) pain management
61. 0.02 Boulic,, R (2010) spatial awareness in full-body immersive interactions: where do we stand?
62. 0.02 Brown,, EC (2012) [potential therapeutic avenues to tackle social cognition problems in schizophrenia](http://dx.doi.org/10.1586/ERN.11.183)
63. 0.02 Buhle,, J (2010) [performance-dependent inhibition of pain by an executive working memory task](http://dx.doi.org/10.1016/j.pain.2009.10.027)
64. 0.02 Campbell,, CM (2010) [catastrophizing delays the analgesic effect of distraction](http://dx.doi.org/10.1016/j.pain.2009.11.012)
65. 0.02 Cartreine,, JA (2010) [a roadmap to computer-based psychotherapy in the united states](http://dx.doi.org/10.3109/10673221003707702)
66. 0.02 Cesa,, GL (2013) [virtual reality for enhancing the cognitive behavioral treatment of obesity with binge eating disorder: randomized controlled study with one-year follow-up](http://dx.doi.org/10.2196/jmir.2441)
67. 0.02 Chavarriaga,, R (2012) anticipation- and error-related eeg signals during realistic human-machine interaction: a study on visual and tactile feedback
68. 0.02 Chittaro,, L (2010) the persuasive power of virtual reality: effects of simulated human distress on attitudes towards fire safety
69. 0.02 Choi,, SH (2010) [deficits in eye gaze during negative social interactions in patients with schizophrenia](http://dx.doi.org/10.1097/NMD.0b013e3181f97c0d)
70. 0.02 Clemente,, M (2010) [contributions of functional magnetic resonance in the field of psychological treatments with virtual reality](http://dx.doi.org/10.3233/978-1-60750-561-7-197)
71. 0.02 Dessing,, JC (2010) [bending it like beckham: how to visually fool the goalkeeper](http://dx.doi.org/10.1371/journal.pone.0013161)
72. 0.02 Diemer,, J (2015) [the impact of perception and presence on emotional reactions: a review of research in virtual reality](http://dx.doi.org/10.3389/fpsyg.2015.00026)
73. 0.02 Doherty,, G (2010) [special issue supportive interaction: computer interventions for mental health introduction](http://dx.doi.org/10.1016/j.intcom.2010.03.002)
74. 0.02 Dyck,, M (2010) [virtual faces as a tool to study emotion recognition deficits in schizophrenia](http://dx.doi.org/10.1016/j.psychres.2009.11.004)
75. 0.02 Ekeland,, AG (2010) [effectiveness of telemedicine: a systematic review of reviews](http://dx.doi.org/10.1016/j.ijmedinf.2010.08.006)
76. 0.02 Emmelkamp,, PMG (2011) [effectiveness of cybertherapy in mental health: a critical appraisal](http://dx.doi.org/10.3233/978-1-60750-766-6-3)
77. 0.02 Ferrer-Garcia,, M (2010) [efficacy of virtual reality in triggering the craving to smoke: its relation to level of presence and nicotine dependence](http://dx.doi.org/10.3233/978-1-60750-561-7-123)
78. 0.02 Ferrer-Garcia,, M (2011) [virtual reality exposure in patients with eating disorders: influence of symptom severity and presence](http://dx.doi.org/10.3233/978-1-60750-766-6-80)
79. 0.02 Ferrer-Garcia,, M (2012) [the use of virtual reality in the treatment of eating disorders](http://dx.doi.org/10.3233/978-1-61499-121-2-17)
80. 0.02 Gamito,, P (2010) [training presence: the importance of virtual reality experience on the "sense of being there"](http://dx.doi.org/10.3233/978-1-60750-561-7-128)
81. 0.02 Gervasi,, O (2010) [nu!rehavr: virtual reality in neuro tele-rehabilitation of patients with traumatic brain injury and stroke](http://dx.doi.org/10.1007/s10055-009-0149-7)
82. 0.02 Giner-Bartolome,, C (2015) [can an intervention based on a serious videogame prior to cognitive behavioral therapy be helpful in bulimia nervosa? a clinical case study](http://dx.doi.org/10.3389/fpsyg.2015.00982)
83. 0.02 Gopher,, D (2010) skill training in multimodal virtual environments
84. 0.02 Groenegress,, C (2010) [the physiological mirror-a system for unconscious control of a virtual environment through physiological activity](http://dx.doi.org/10.1007/s00371-010-0471-9)
85. 0.02 Gruzelier,, J (2010) [acting performance and flow state enhanced with sensory-motor rhythm neurofeedback comparing ecologically valid immersive vr and training screen scenarios](http://dx.doi.org/10.1016/j.neulet.2010.06.019)
86. 0.02 Guardia,, D (2012) [imagining one's own and someone else's body actions: dissociation in anorexia nervosa](http://dx.doi.org/10.1371/journal.pone.0043241)
87. 0.02 Guttentag,, DA (2010) [virtual reality: applications and implications for tourism](http://dx.doi.org/10.1016/j.tourman.2009.07.003)
88. 0.02 Han,, K (2012) [assessment of cognitive flexibility in real life using virtual reality: a comparison of healthy individuals and schizophrenia patients](http://dx.doi.org/10.1016/j.compbiomed.2012.06.007)
89. 0.02 Han,, K (2012) [the effect of simulated auditory hallucinations on daily activities in schizophrenia patients](http://dx.doi.org/10.1159/000337264)
90. 0.02 Hancock,, AB (2010) [public speaking attitudes: does curriculum make a difference?](http://dx.doi.org/10.1016/j.jvoice.2008.09.007)
91. 0.02 Handouzi,, W (2014) [objective model assessment for short-term anxiety recognition from blood volume pulse signal](http://dx.doi.org/10.1016/j.bspc.2014.07.008)
92. 0.02 Hartanto,, D (2014) [controlling social stress in virtual reality environments](http://dx.doi.org/10.1371/journal.pone.0092804)
93. 0.02 Head,, D (2010) [age effects on wayfinding and route learning skills](http://dx.doi.org/10.1016/j.bbr.2010.01.012)
94. 0.02 Jahn,, K (2010) [functional imaging of locomotion and navigation. physiology and neurodegeneration](http://dx.doi.org/10.1007/s00115-010-3103-8)
95. 0.02 Johnson,, S (2014) [stereotype confirmation concerns predict dropout from cognitive behavioral therapy for social anxiety disorder](http://dx.doi.org/10.1186/s12888-014-0233-8)
96. 0.02 Johnson,, SB (2014) [stereotype confirmation concern and fear of negative evaluation among african americans and caucasians with social anxiety disorder](http://dx.doi.org/10.1016/j.janxdis.2014.03.003)
97. 0.02 Jun,, Y (2012) [anatomic basis 3-d surgical simulation system for custom fit knee replacement](http://dx.doi.org/10.1007/s12541-012-0092-5)
98. 0.02 Kalyanaraman,, S (2010) [the virtual doppelganger effects of a virtual reality simulator on perceptions of schizophrenia](http://dx.doi.org/10.1097/NMD.0b013e3181e07d66)
99. 0.02 Kartiko,, I (2010) [learning science in a virtual reality application: the impacts of animated-virtual actors' visual complexity](http://dx.doi.org/10.1016/j.compedu.2010.03.019)
100. 0.02 Katzman,, MA (2014) [canadian clinical practice guidelines for the management of anxiety, posttraumatic stress and obsessive-compulsive disorders](http://dx.doi.org/10.1186/1471-244X-14-S1-S1)
101. 0.02 Kilteni,, K (2012) the sense of embodiment in virtual reality
102. 0.02 Kilteni,, K (2013) drumming in immersive virtual reality: the body shapes the way we play
103. 0.02 Kramer,, TL (2010) clinician perceptions of virtual reality to assess and treat returning veterans
104. 0.02 La, Paglia, F (2012) [assessment of executive functions in patients with obsessive compulsive disorder by neurovr](http://dx.doi.org/10.3233/978-1-61499-121-2-98)
105. 0.02 Lange,, B (2012) [designing informed game-based rehabilitation tasks leveraging advances in virtual reality](http://dx.doi.org/10.3109/09638288.2012.670029)
106. 0.02 Ling,, Y (2014) [a meta-analysis on the relationship between self-reported presence and anxiety in virtual reality exposure therapy for anxiety disorders](http://dx.doi.org/10.1371/journal.pone.0096144)
107. 0.02 Linkenauger,, SA (2013) [welcome to wonderland: the influence of the size and shape of a virtual hand on the perceived size and shape of virtual objects](http://dx.doi.org/10.1371/journal.pone.0068594)
108. 0.02 Lipsitz,, JD (2014) specific phobia
109. 0.02 Llobera,, J (2013) [the relationship between virtual body ownership and temperature sensitivity](http://dx.doi.org/10.1098/rsif.2013.0300)
110. 0.02 Londero,, A (2010) [auditory and visual 3d virtual reality therapy for chronic subjective tinnitus: theoretical framework](http://dx.doi.org/10.1007/s10055-009-0135-0)
111. 0.02 Lorenzo,, MG (2011) efficacy of virtual reality exposure therapy combined with two pharmacotherapies in the treatment of agoraphobia
112. 0.02 Martini,, M (2013) [what color is my arm? changes in skin color of an embodied virtual arm modulates pain threshold](http://dx.doi.org/10.3389/fnhum.2013.00438)
113. 0.02 Maselli,, A (2013) [the building blocks of the full body ownership illusion](http://dx.doi.org/10.3389/fnhum.2013.00083)
114. 0.02 McLay,, RN (2010) [exposure therapy with and without virtual reality to treat ptsd while in the combat theater: a parallel case series](http://dx.doi.org/10.1089/cyber.2009.0346)
115. 0.02 Meyerbroker,, K (2011) [virtual reality exposure treatment of agoraphobia: a comparison of computer automatic virtual environment and head-mounted display](http://dx.doi.org/10.3233/978-1-60750-766-6-51)
116. 0.02 Miller,, K (2010) [multi-modal distraction. using technology to combat pain in young children with burn injuries](http://dx.doi.org/10.1016/j.burns.2009.06.199)
117. 0.02 Miyahira,, SD (2010) [effectiveness of brief vr treatment for ptsd in war-fighters: a case study](http://dx.doi.org/10.3233/978-1-60750-561-7-214)
118. 0.02 Miyahira,, SD (2012) [the effectiveness of vr exposure therapy for ptsd in returning warfighters](http://dx.doi.org/10.3233/978-1-61499-121-2-128)
119. 0.02 Morgan,, JR (2014) [cognitive processes as mediators of the relation between mindfulness and change in social anxiety symptoms following cognitive behavioral treatment](http://dx.doi.org/10.1080/10615806.2013.839988)
120. 0.02 Morgan,, JR (2014) [discrepancies in therapist and client ratings of global improvement following cognitive behavioral therapy for social phobia and their differential relations with symptom improvement at post-treatment and 12-month follow-up](http://dx.doi.org/10.1080/10503307.2013.852268)
121. 0.02 Morganti,, F (2013) [from allo- to egocentric spatial ability in early alzheimer's disease: a study with virtual reality spatial tasks](http://dx.doi.org/10.1080/17588928.2013.854762)
122. 0.02 Morris,, LD (2010) [feasibility and potential effect of a low-cost virtual reality system on reducing pain and anxiety in adult burn injury patients during physiotherapy in a developing country](http://dx.doi.org/10.1016/j.burns.2009.09.005)
123. 0.02 Mosig,, C (2014) [enhanced discriminative fear learning of phobia-irrelevant stimuli in spider-fearful individuals](http://dx.doi.org/10.3389/fnbeh.2014.00328)
124. 0.02 Notzon,, S (2015) [psychophysiological effects of an itbs modulated virtual reality challenge including participants with spider phobia](http://dx.doi.org/10.1016/j.biopsycho.2015.10.003)
125. 0.02 Oakes,, M (2010) [the psychology of fear of flying (part ii): a critical evaluation of current perspectives on approaches to treatment](http://dx.doi.org/10.1016/j.tmaid.2010.10.002)
126. 0.02 Optale,, G (2010) [controlling memory impairment in elderly adults using virtual reality memory training: a randomized controlled pilot study](http://dx.doi.org/10.1177/1545968309353328)
127. 0.02 Paul,, LA (2012) [technological advances in the treatment of trauma: a review of promising practices](http://dx.doi.org/10.1177/0145445512450733)
128. 0.02 Peck,, TC (2013) [putting yourself in the skin of a black avatar reduces implicit racial bias](http://dx.doi.org/10.1016/j.concog.2013.04.016)
129. 0.02 Peperkorn,, HM (2015) [temporal dynamics in the relation between presence and fear in virtual reality](http://dx.doi.org/10.1016/j.chb.2015.02.028)
130. 0.02 Perez-Ara,, MA (2010) [virtual reality interoceptive exposure for the treatment of panic disorder and agoraphobia](http://dx.doi.org/10.3233/978-1-60750-561-7-77)
131. 0.02 Picard,, L (2011) virtual reality: an ecological method to assess multifaceted episodic memory development
132. 0.02 Plancher,, G (2013) [the influence of action on episodic memory: a virtual reality study](http://dx.doi.org/10.1080/17470218.2012.722657)
133. 0.02 Portnoy,, DB (2010) [perceiving interpersonally-mediated risk in virtual environments](http://dx.doi.org/10.1007/s10055-009-0120-7)
134. 0.02 Powell,, V (2010) [visual properties of an object affect time to target in vr reaching tasks](http://dx.doi.org/10.3233/978-1-60750-561-7-180)
135. 0.02 Price,, M (2011) [the impact of cognitive behavioral therapy on post event processing among those with social anxiety disorder](http://dx.doi.org/10.1016/j.brat.2010.11.006)
136. 0.02 Raskin,, SA (2010) [current approaches to cognitive rehabilitation](http://dx.doi.org/10.1007/978-1-4419-1364-7_28)
137. 0.02 Risbrough,, V (2010) [behavioral correlates of anxiety](http://dx.doi.org/10.1007/7854_2009_11)
138. 0.02 Riva,, G (2012) [allocentric lock in anorexia nervosa: new evidences from neuroimaging studies](http://dx.doi.org/10.1016/j.mehy.2012.03.036)
139. 0.02 Robillard,, G (2010) [using virtual humans to alleviate social anxiety: preliminary report from a comparative outcome study](http://dx.doi.org/10.3233/978-1-60750-561-7-57)
140. 0.02 Rosenberg,, RS (2013) [virtual superheroes: using superpowers in virtual reality to encourage prosocial behavior](http://dx.doi.org/10.1371/journal.pone.0055003)
141. 0.02 Roy,, MJ (2010) [improvement in cerebral function with treatment of posttraumatic stress disorder](http://dx.doi.org/10.1111/j.1749-6632.2010.05689.x)
142. 0.02 Rus-Calafell,, M (2012) [improving social behaviour in schizophrenia patients using an integrated virtual reality programme: a case study](http://dx.doi.org/10.3233/978-1-61499-121-2-283)
143. 0.02 Salamin,, P (2010) [quantifying effects of exposure to the third and first-person perspectives in virtual-reality-based training](http://dx.doi.org/10.1109/TLT.2010.13)
144. 0.02 Sato,, K (2010) nonimmersive virtual reality mirror visual feedback therapy and its application for the treatment of complex regional pain syndrome: an open-label pilot study
145. 0.02 Schultze,, U (2010) [embodiment and presence in virtual worlds: a review](http://dx.doi.org/10.1057/jit.2010.25)
146. 0.02 Serino,, S (2014) [the role of egocentric and allocentric abilities in alzheimer's disease: a systematic review](http://dx.doi.org/10.1016/j.arr.2014.04.004)
147. 0.02 Serino,, S (2015) [out of body, out of space: impaired reference frame processing in eating disorders](http://dx.doi.org/10.1016/j.psychres.2015.10.025)
148. 0.02 Smith,, JW (2015) [immersive virtual environment technology to supplement environmental perception, preference and behavior research: a review with applications](http://dx.doi.org/10.3390/ijerph120911486)
149. 0.02 Smith,, MJ (2014) [virtual reality job interview training in adults with autism spectrum disorder](http://dx.doi.org/10.1007/s10803-014-2113-y)
150. 0.02 Smith,, ST (2010) agent-based monitoring of functional rehabilitation using video games
151. 0.02 Steinicke,, F (2010) [gradual transitions and their effects on presence and distance estimation](http://dx.doi.org/10.1016/j.cag.2009.12.003)
152. 0.02 Sutton,, JE (2010) [spinning in the scanner: neural correlates of virtual reorientation](http://dx.doi.org/10.1037/a0019938)
153. 0.02 Suzuki,, K (2012) [substitutional reality system: a novel experimental platform for experiencing alternative reality](http://dx.doi.org/10.1038/srep00459)
154. 0.02 Turner,, WA (2014) [outcomes associated with virtual reality in psychological interventions: where are we now?](http://dx.doi.org/10.1016/j.cpr.2014.10.003)
155. 0.02 Vakili,, V (2014) [characteristics of successful technological interventions in mental resilience training](http://dx.doi.org/10.1007/s10916-014-0113-2)
156. 0.02 Van, Damme, S (2010) [keeping pain in mind: a motivational account of attention to pain](http://dx.doi.org/10.1016/j.neubiorev.2009.01.005)
157. 0.02 Vecchiato,, G (2015) [electroencephalographic correlates of sensorimotor integration and embodiment during the appreciation of virtual architectural environments](http://dx.doi.org/10.3389/fpsyg.2015.01944)
158. 0.02 Wallach,, HS (2010) [personality variables and presence](http://dx.doi.org/10.1007/s10055-009-0124-3)
159. 0.02 Wang,, Z (2010) influence of vision and haptics on plausibility of social interaction in virtual reality scenarios
160. 0.02 Waterlander,, WE (2015) [using a 3d virtual supermarket to measure food purchase behavior: a validation study](http://dx.doi.org/10.2196/jmir.3774)
161. 0.02 Wiederhold,, B (2012) [a brief review of positive technology in europe and the usa](http://dx.doi.org/10.3233/978-1-61499-121-2-46)
162. 0.02 Wiederhold,, BK (2010) [virtual reality treatment of posttraumatic stress disorder due to motor vehicle accident](http://dx.doi.org/10.1089/cyber.2009.0394)
163. 0.02 Yamada,, J (2010) audience effects on stuttering: a japanese case study
164. 0.02 Yuan,, Y (2010) is the rubber hand illusion induced by immersive virtual reality?
165. 0.02 Zaalberg,, R (2010) human responses to climate change flooding experiences in the netherlands
166. 0.02 Zschaler,, S (2010) [increased pain through psychological therapy?](http://dx.doi.org/10.1007/s00482-010-0981-4)
167. 0.02 de, Bruin, ED (2010) [use of virtual reality technique for the training of motor control in the elderly some theoretical considerations](http://dx.doi.org/10.1007/s00391-010-0124-7)
