# Supplementary material for: The Past, Present, and Future of Virtual and Augmented Reality Research: A Network and Cluster Analysis of the Literature
Source: Front Psychol. 2018 Nov 6;9:2086. doi: 10.3389/fpsyg.2018.02086 (PMC6232426; doi:10.3389/fpsyg.2018.02086)
Supplement: Supplementary file 1 [file Data_Sheet_1.ZIP › NARRATIVES - Authors Publications.docx]

**NARRATIVES**

**MAJOR CLUSTERS**

The network is divided into **29** co-citation clusters. These clusters are labeled by index terms from their own citers. The largest **11** clusters are summarized.

**Table 1. Summary of the largest 11 clusters.**

| **ClusterID** | **Size** | **Silhouette** | **Label (TFIDF)** | **Label (LLR)** | **Label (MI)** | **mean(Citee Year)** |
| --- | --- | --- | --- | --- | --- | --- |
| 0 | 44 | 0.956 | (24.13) eating disorder | eating disorder (38.46, 1.0E-4) | human factor | 2007 |
| 1 | 33 | 0.988 | (18.53) soldier suffering | soldier suffering (23.97, 1.0E-4) | virtual space | 2004 |
| 2 | 20 | 0.955 | (19.73) combat | virtual reality exposure therapy (34.19, 1.0E-4) | future | 2006 |
| 3 | 20 | 1 | (19.83) content | face (38.82, 1.0E-4) | surgery | 2009 |
| 4 | 13 | 1 | (24.75) brain-computer interface | brain-computer interface (72.93, 1.0E-4) | investigation | 2007 |
| 5 | 10 | 1 | (19.18) report | report (75.25, 1.0E-4) | future | 1991 |
| 6 | 10 | 1 | (19.83) way | work (29.65, 1.0E-4) | safety | 2008 |
| 7 | 10 | 1 | (26.34) hyper hospital | hyper hospital (101.51, 1.0E-4) | home | 1995 |
| 8 | 10 | 1 | (25.32) nedo project | 3-d rehabilitation system (84.62, 1.0E-4) | research | 2005 |
| 9 | 9 | 1 | (24.13) medical-care system | virtual reality network (55.27, 1.0E-4) | virtual-reality system | 1994 |
| 10 | 8 | 1 | (18.53) cardiac intervention | cardiac intervention (42.55, 1.0E-4) | guidance | 2007 |

The largest cluster (#0) has 44 members and a silhouette value of 0.956. It is labeled as ***eating disorder*** by both LLR and TFIDF, and as *human factor* by MI. The most active citer to the cluster is 0.09Albani,, G (2010) [executive functions in a virtual world: a study in parkinson's disease](http://dx.doi.org/10.3233/978-1-60750-561-7-92).

The second largest cluster (#1) has 33 members and a silhouette value of 0.988. It is labeled as ***soldier suffering*** by both LLR and TFIDF, and as *virtual space* by MI. The most active citer to the cluster is0.09 Ahn,, HB (2001) the development of virtual reality driving simulator for rehabilitaion.

The third largest cluster (#2) has 20 members and a silhouette value of 0.955. It is labeled as *virtual reality exposure therapy* by LLR, *combat* by TFIDF, and *future* by MI. The most active citer to the cluster is 0.1 Gerardi,, M (2010) [virtual reality exposure therapy for post-traumatic stress disorder and other anxiety disorders](http://dx.doi.org/10.1007/s11920-010-0128-4).

The 4th largest cluster (#3) has 20 members and a silhouette value of 1. It is labeled as *face* by LLR, *content* by TFIDF, and *surgery* by MI. The most active citer to the cluster is 0.15 Ahmed,, K (2010)[assessment and maintenance of competence in urology](http://dx.doi.org/10.1038/nrurol.2010.81).

The 5th largest cluster (#4) has 13 members and a silhouette value of 1. It is labeled as ***brain-computer interface*** by both LLR and TFIDF, and as *investigation* by MI. The most active citer to the cluster is0.23 Usoh,, M (1999) walking > walking-in-place > flying, in virtual environments.

The 6th largest cluster (#5) has 10 members and a silhouette value of 1. It is labeled as ***report*** by both LLR and TFIDF, and as *future* by MI. The most active citer to the cluster is 0.9 LANIER,, J (1991) applications of virtual reality - reports from the field.

The 7th largest cluster (#6) has 10 members and a silhouette value of 1. It is labeled as *work* by LLR, *way* by TFIDF, and *safety* by MI. The most active citer to the cluster is 0.4 Evangelista,, C (1999) picture transposition in an immersive virtual representation for work of art fruition in a traditional museum.

The 8th largest cluster (#7) has 10 members and a silhouette value of 1. It is labeled as ***hyper hospital*** by both LLR and TFIDF, and as *home* by MI. The most active citer to the cluster is 0.7 Sugioka,, Y (1995) a controlled study of the safety features of a virtual reality system for the development of the hyper hospital.

The 9th largest cluster (#8) has 10 members and a silhouette value of 1. It is labeled as *3-d rehabilitation system* by LLR, *nedo project* by TFIDF, and *research* by MI. The most active citer to the cluster is 0.4Furusho,, J (2005) a 3-d exercise machine for upper-limb rehabilitation using er actuators with high safety.

The 10th largest cluster (#9) has 9 members and a silhouette value of 1. It is labeled as *virtual reality network* by LLR, *medical-care system* by TFIDF, and *virtual-reality system* by MI. The most active citer to the cluster is 0.89 YAMAGUCHI,, T (1994) the hyper-hospital - a networked reality based medical-care system.

The 11th largest cluster (#10) has 8 members and a silhouette value of 1. It is labeled as ***cardiac intervention*** by both LLR and TFIDF, and as *guidance* by MI. The most active citer to the cluster is 0.5Moore,, JT (2010) [integration of trans-esophageal echocardiography with magnetic tracking technology for cardiac interventions](http://dx.doi.org/10.1117/12.844273).

**CITATION COUNTS**

The top ranked item by citation counts is Riva G (1998) in Cluster #0, with citation counts of **180**. The second one is Darzi A (2001) in Cluster #3, with citation counts of **97**. The third is Aggarwal R (2005) in Cluster #3, with citation counts of **94**. The 4th is Slater M (1999) in Cluster #4, with citation counts of **90**. The 5th is Alcaniz M (2000) in Cluster #0, with citation counts of **89**. The 6th is [Anonymous] (1991) in Cluster #286, with citation counts of **88**. The 7th is Botella C (2000) in Cluster #0, with citation counts of **85**. The 8th is Wiederhold BK (2000) in Cluster #1, with citation counts of **75**. The 9th is Kim SI (2001) in Cluster #1, with citation counts of **74**. The 10th is Rizzo A (2005) in Cluster #2, with citation counts of **59**.

| **citation counts** | **references** | **cluster #** |
| --- | --- | --- |
| 180 | Riva G, 1998, SO, V, P | 0 |
| 97 | Darzi A, 2001, SO, V, P | 3 |
| 94 | Aggarwal R, 2005, SO, V, P | 3 |
| 90 | Slater M, 1999, SO, V, P | 4 |
| 89 | Alcaniz M, 2000, SO, V, P | 0 |
| 88 | [Anonymous], 1991, SO, V, P | 286 |
| 85 | Botella C, 2000, SO, V, P | 0 |
| 75 | Wiederhold BK, 2000, SO, V, P | 1 |
| 74 | Kim SI, 2001, SO, V, P | 1 |
| 59 | Rizzo A, 2005, SO, V, P | 2 |

**BURSTS**

| **bursts** | **references** | **cluster #** |
| --- | --- | --- |

**CENTRALITY**

The top ranked item by centrality is Riva G (1998) in Cluster #0, with centrality of **0.00**. The second one is Darzi A (2001) in Cluster #3, with centrality of **0.00**. The third is Aggarwal R (2005) in Cluster #3, with centrality of **0.00**. The 4th is Slater M (1999) in Cluster #4, with centrality of **0.00**. The 5th is Alcaniz M (2000) in Cluster #0, with centrality of **0.00**. The 6th is [Anonymous] (1991) in Cluster #286, with centrality of **0.00**. The 7th is Botella C (2000) in Cluster #0, with centrality of **0.00**. The 8th is Wiederhold BK (2000) in Cluster #1, with centrality of **0.00**. The 9th is Kim SI (2001) in Cluster #1, with centrality of **0.00**. The 10th is Rizzo A (2005) in Cluster #2, with centrality of **0.00**.

| **centrality** | **references** | **cluster #** |
| --- | --- | --- |
| 0.00 | Riva G, 1998, SO, V, P | 0 |
| 0.00 | Darzi A, 2001, SO, V, P | 3 |
| 0.00 | Aggarwal R, 2005, SO, V, P | 3 |
| 0.00 | Slater M, 1999, SO, V, P | 4 |
| 0.00 | Alcaniz M, 2000, SO, V, P | 0 |
| 0.00 | [Anonymous], 1991, SO, V, P | 286 |
| 0.00 | Botella C, 2000, SO, V, P | 0 |
| 0.00 | Wiederhold BK, 2000, SO, V, P | 1 |
| 0.00 | Kim SI, 2001, SO, V, P | 1 |
| 0.00 | Rizzo A, 2005, SO, V, P | 2 |

**SIGMA**

The top ranked item by sigma is Riva G (1998) in Cluster #0, with sigma of **1.00**. The second one is Darzi A (2001) in Cluster #3, with sigma of **1.00**. The third is Aggarwal R (2005) in Cluster #3, with sigma of **1.00**. The 4th is Slater M (1999) in Cluster #4, with sigma of **1.00**. The 5th is Alcaniz M (2000) in Cluster #0, with sigma of **1.00**. The 6th is [Anonymous] (1991) in Cluster #286, with sigma of **1.00**. The 7th is Botella C (2000) in Cluster #0, with sigma of **1.00**. The 8th is Wiederhold BK (2000) in Cluster #1, with sigma of **1.00**. The 9th is Kim SI (2001) in Cluster #1, with sigma of **1.00**. The 10th is Rizzo A (2005) in Cluster #2, with sigma of **1.00**.

| **sigma** | **references** | **cluster #** |
| --- | --- | --- |
| 1.00 | Riva G, 1998, SO, V, P | 0 |
| 1.00 | Darzi A, 2001, SO, V, P | 3 |
| 1.00 | Aggarwal R, 2005, SO, V, P | 3 |
| 1.00 | Slater M, 1999, SO, V, P | 4 |
| 1.00 | Alcaniz M, 2000, SO, V, P | 0 |
| 1.00 | [Anonymous], 1991, SO, V, P | 286 |
| 1.00 | Botella C, 2000, SO, V, P | 0 |
| 1.00 | Wiederhold BK, 2000, SO, V, P | 1 |
| 1.00 | Kim SI, 2001, SO, V, P | 1 |
| 1.00 | Rizzo A, 2005, SO, V, P | 2 |
