# Supplementary material for: The Past, Present, and Future of Virtual and Augmented Reality Research: A Network and Cluster Analysis of the Literature
Source: Front Psychol. 2018 Nov 6;9:2086. doi: 10.3389/fpsyg.2018.02086 (PMC6232426; doi:10.3389/fpsyg.2018.02086)
Supplement: Supplementary file 1 [file Data_Sheet_1.ZIP › NARRATIVES - Category_WoS.docx]

**NARRATIVES**

**MAJOR CLUSTERS**

The network is divided into **6** co-citation clusters. These clusters are labeled by index terms from their own citers. The largest **2** clusters are summarized.

**Table 1. Summary of the largest 2 clusters.**

| **ClusterID** | **Size** | **Silhouette** | **Label (TFIDF)** | **Label (LLR)** | **Label (MI)** | **mean(Citee Year)** |
| --- | --- | --- | --- | --- | --- | --- |
| 0 | 27 | 0.523 | (9.73) robotic system | virtual reality application (26.67, 1.0E-4) | advanced open source | 1996 |
| 1 | 26 | 0.656 | (10.17) feasibility | patient (53.32, 1.0E-4) | accuracy | 1997 |

The largest cluster (#0) has 27 members and a silhouette value of 0.523. It is labeled as *virtual reality application* by LLR, *robotic system* by TFIDF, and *advanced open source* by MI. The most active citer to the cluster is 0.15 Boulianne,, M (1999) virtual reality applied to user interfaces for digital photogrammetric workstations.

The second largest cluster (#1) has 26 members and a silhouette value of 0.656. It is labeled as *patient* by LLR, *feasibility* by TFIDF, and *accuracy* by MI. The most active citer to the cluster is 0.12Klobucka,, S (2013) the effect of virtual reality environment during robotic-assisted locomotor training on gross motor functions in patients with cerebral palsy.

**CITATION COUNTS**

The top ranked item by citation counts is COMPUTER SCIENCE (1990) in Cluster #4, with citation counts of **9131**. The second one is ENGINEERING (1990) in Cluster #0, with citation counts of **6210**. The third is PSYCHOLOGY (1990) in Cluster #3, with citation counts of **1779**. The 4th is NEUROSCIENCES & NEUROLOGY (1992) in Cluster #1, with citation counts of **1548**. The 5th is SURGERY (1992) in Cluster #1, with citation counts of **1418**. The 6th is AUTOMATION & CONTROL SYSTEMS (1993) in Cluster #0, with citation counts of **1267**. The 7th is NEUROSCIENCES (1992) in Cluster #1, with citation counts of **1040**. The 8th is IMAGING SCIENCE & PHOTOGRAPHIC TECHNOLOGY (1992) in Cluster #0, with citation counts of **1027**. The 9th is EDUCATION & EDUCATIONAL RESEARCH (1993) in Cluster #3, with citation counts of **931**. The 10th is ROBOTICS (1992) in Cluster #0, with citation counts of **849**.

| **citation counts** | **references** | **cluster #** |
| --- | --- | --- |
| 9131 | COMPUTER SCIENCE, 1990, SO, V, P | 4 |
| 6210 | ENGINEERING, 1990, SO, V, P | 0 |
| 1779 | PSYCHOLOGY, 1990, SO, V, P | 3 |
| 1548 | NEUROSCIENCES & NEUROLOGY, 1992, SO, V, P | 1 |
| 1418 | SURGERY, 1992, SO, V, P | 1 |
| 1267 | AUTOMATION & CONTROL SYSTEMS, 1993, SO, V, P | 0 |
| 1040 | NEUROSCIENCES, 1992, SO, V, P | 1 |
| 1027 | IMAGING SCIENCE & PHOTOGRAPHIC TECHNOLOGY, 1992, SO, V, P | 0 |
| 931 | EDUCATION & EDUCATIONAL RESEARCH, 1993, SO, V, P | 3 |
| 849 | ROBOTICS, 1992, SO, V, P | 0 |

**BURSTS**

The top ranked item by bursts is OPTICS (1991) in Cluster #0, with bursts of **110.63**. The second one is NEUROSCIENCES & NEUROLOGY (1992) in Cluster #1, with bursts of **91.27**. The third is NEUROSCIENCES (1992) in Cluster #1, with bursts of **77.71**. The 4th is COMMUNICATION (1992) in Cluster #2, with bursts of **75.75**. The 5th is IMAGING SCIENCE & PHOTOGRAPHIC TECHNOLOGY (1992) in Cluster #0, with bursts of **67.42**. The 6th is INFORMATION SCIENCE & LIBRARY SCIENCE (1991) in Cluster #2, with bursts of **55.53**. The 7th is MATERIALS SCIENCE (1994) in Cluster #0, with bursts of **48.77**. The 8th is RESEARCH & EXPERIMENTAL MEDICINE (2003) in Cluster #1, with bursts of **39.02**. The 9th is RADIOLOGY (1994) in Cluster #1, with bursts of**37.17**. The 10th is AUTOMATION & CONTROL SYSTEMS (1993) in Cluster #0, with bursts of **33.57**.

| **bursts** | **references** | **cluster #** |
| --- | --- | --- |
| 110.63 | OPTICS, 1991, SO, V, P | 0 |
| 91.27 | NEUROSCIENCES & NEUROLOGY, 1992, SO, V, P | 1 |
| 77.71 | NEUROSCIENCES, 1992, SO, V, P | 1 |
| 75.75 | COMMUNICATION, 1992, SO, V, P | 2 |
| 67.42 | IMAGING SCIENCE & PHOTOGRAPHIC TECHNOLOGY, 1992, SO, V, P | 0 |
| 55.53 | INFORMATION SCIENCE & LIBRARY SCIENCE, 1991, SO, V, P | 2 |
| 48.77 | MATERIALS SCIENCE, 1994, SO, V, P | 0 |
| 39.02 | RESEARCH & EXPERIMENTAL MEDICINE, 2003, SO, V, P | 1 |
| 37.17 | RADIOLOGY, 1994, SO, V, P | 1 |
| 33.57 | AUTOMATION & CONTROL SYSTEMS, 1993, SO, V, P | 0 |

**CENTRALITY**

The top ranked item by centrality is COMPUTER SCIENCE (1990) in Cluster #4, with centrality of **0.50**. The second one is ENGINEERING (1990) in Cluster #0, with centrality of **0.31**. The third is EDUCATION & EDUCATIONAL RESEARCH (1993) in Cluster #3, with centrality of **0.18**. The 4th is RADIOLOGY (1994) in Cluster #1, with centrality of **0.13**. The 5th is NEUROSCIENCES & NEUROLOGY (1992) in Cluster #1, with centrality of **0.11**. The 6th is ENVIRONMENTAL SCIENCES & ECOLOGY (1992) in Cluster #2, with centrality of **0.09**. The 7th is NEUROSCIENCES (1992) in Cluster #1, with centrality of **0.08**. The 8th is SCIENCE & TECHNOLOGY - OTHER TOPICS (1990) in Cluster #5, with centrality of **0.08**. The 9th is SURGERY (1992) in Cluster #1, with centrality of**0.07**. The 10th is BUSINESS & ECONOMICS (1991) in Cluster #2, with centrality of **0.06**.

| **centrality** | **references** | **cluster #** |
| --- | --- | --- |
| 0.50 | COMPUTER SCIENCE, 1990, SO, V, P | 4 |
| 0.31 | ENGINEERING, 1990, SO, V, P | 0 |
| 0.18 | EDUCATION & EDUCATIONAL RESEARCH, 1993, SO, V, P | 3 |
| 0.13 | RADIOLOGY, 1994, SO, V, P | 1 |
| 0.11 | NEUROSCIENCES & NEUROLOGY, 1992, SO, V, P | 1 |
| 0.09 | ENVIRONMENTAL SCIENCES & ECOLOGY, 1992, SO, V, P | 2 |
| 0.08 | NEUROSCIENCES, 1992, SO, V, P | 1 |
| 0.08 | SCIENCE & TECHNOLOGY - OTHER TOPICS, 1990, SO, V, P | 5 |
| 0.07 | SURGERY, 1992, SO, V, P | 1 |
| 0.06 | BUSINESS & ECONOMICS, 1991, SO, V, P | 2 |

**SIGMA**

The top ranked item by sigma is NEUROSCIENCES & NEUROLOGY (1992) in Cluster #1, with sigma of **13701.82**. The second one is NEUROSCIENCES (1992) in Cluster #1, with sigma of **453.17**. The third is RADIOLOGY (1994) in Cluster #1, with sigma of **105.68**. The 4th is OPTICS (1991) in Cluster #0, with sigma of **15.26**. The 5th is MATERIALS SCIENCE (1994) in Cluster #0, with sigma of **11.68**. The 6th is COMMUNICATION (1992) in Cluster #2, with sigma of **11.68**. The 7th is INFORMATION SCIENCE & LIBRARY SCIENCE (1991) in Cluster #2, with sigma of **4.54**. The 8th is SCIENCE & TECHNOLOGY - OTHER TOPICS (1990) in Cluster #5, with sigma of **4.15**. The 9th is IMAGING SCIENCE & PHOTOGRAPHIC TECHNOLOGY (1992) in Cluster #0, with sigma of **3.61**. The 10th is RESEARCH & EXPERIMENTAL MEDICINE (2003) in Cluster #1, with sigma of **2.84**.

| **sigma** | **references** | **cluster #** |
| --- | --- | --- |
| 13701.82 | NEUROSCIENCES & NEUROLOGY, 1992, SO, V, P | 1 |
| 453.17 | NEUROSCIENCES, 1992, SO, V, P | 1 |
| 105.68 | RADIOLOGY, 1994, SO, V, P | 1 |
| 15.26 | OPTICS, 1991, SO, V, P | 0 |
| 11.68 | MATERIALS SCIENCE, 1994, SO, V, P | 0 |
| 11.68 | COMMUNICATION, 1992, SO, V, P | 2 |
| 4.54 | INFORMATION SCIENCE & LIBRARY SCIENCE, 1991, SO, V, P | 2 |
| 4.15 | SCIENCE & TECHNOLOGY - OTHER TOPICS, 1990, SO, V, P | 5 |
| 3.61 | IMAGING SCIENCE & PHOTOGRAPHIC TECHNOLOGY, 1992, SO, V, P | 0 |
| 2.84 | RESEARCH & EXPERIMENTAL MEDICINE, 2003, SO, V, P | 1 |
