# Supplementary material for: The Past, Present, and Future of Virtual and Augmented Reality Research: A Network and Cluster Analysis of the Literature
Source: Front Psychol. 2018 Nov 6;9:2086. doi: 10.3389/fpsyg.2018.02086 (PMC6232426; doi:10.3389/fpsyg.2018.02086)
Supplement: Supplementary file 1 [file Data_Sheet_1.ZIP › NARRATIVES - Citations clusters AR.docx]

**MAJOR CLUSTERS**

The network is divided into **22** co-citation clusters. These clusters are labeled by index terms from their own citers. The largest **8** clusters are summarized.

**Table 1. Summary of the largest 8 clusters.**

| **ClusterID** | **Size** | **Silhouette** | **Label (TFIDF)** | **Label (LLR)** | **Label (MI)** | **mean(Citee Year)** |
| --- | --- | --- | --- | --- | --- | --- |
| 0 | 122 | 0.669 | (18.41) internet | internet (39.96, 1.0E-4) | automatic confidence adjustment | 1999 |
| 1 | 66 | 0.806 | (16.67) tracking | mobile phone (47.52, 1.0E-4) | gyeongbokgung | 2007 |
| 2 | 65 | 0.827 | (17.48) natural environment | natural feature tracking (57.72, 1.0E-4) | dynamic registration error | 1994 |
| 3 | 56 | 0.89 | (17.33) liver | laparoscopic surgery (30.43, 1.0E-4) | analysis | 2004 |
| 4 | 50 | 0.943 | (19.32) education | education (64.26, 1.0E-4) | assessment | 2011 |
| 5 | 48 | 0.86 | (15.96) virtual city environment | virtual city environment (32.68, 1.0E-4) | educational application | 2007 |
| 6 | 20 | 0.997 | (21.65) knowledge-based augmented reality | knowledge-based augmented reality (250.67, 1.0E-4) | ... | 1989 |
| 7 | 19 | 0.926 | (19.32) hand-eye calibration | hand-eye calibration (104.98, 1.0E-4) | analysis | 1992 |

The largest cluster (#0) has 122 members and a silhouette value of 0.669. It is labeled as ***internet*** by both LLR and TFIDF, and as *automatic confidence adjustment* by MI. The most active citer to the cluster is 0.07Azuma,, R (2001) [recent advances in augmented reality](http://dx.doi.org/10.1109/38.963459).

The second largest cluster (#1) has 66 members and a silhouette value of 0.806. It is labeled as *mobile phone* by LLR, *tracking* by TFIDF, and *gyeongbokgung* by MI. The most active citer to the cluster is 0.11 Guan,, T (2010) [fast scene recognition and camera relocalisation for wide area augmented reality systems](http://dx.doi.org/10.3390/s100606017).

The third largest cluster (#2) has 65 members and a silhouette value of 0.827. It is labeled as *natural feature tracking* by LLR, *natural environment* by TFIDF, and *dynamic registration error* by MI. The most active citer to the cluster is 0.23 Neumann,, U (1999) augmented reality tracking in natural environments.

The 4th largest cluster (#3) has 56 members and a silhouette value of 0.89. It is labeled as *laparoscopic surgery* by LLR, *liver* by TFIDF, and *analysis* by MI. The most active citer to the cluster is 0.09 Figl,, M (2010) [image guidance for robotic minimally invasive coronary artery bypass](http://dx.doi.org/10.1016/j.compmedimag.2009.08.002).

The 5th largest cluster (#4) has 50 members and a silhouette value of 0.943. It is labeled as ***education*** by both LLR and TFIDF, and as *assessment* by MI. The most active citer to the cluster is 0.12 Bacca,, J (2014) augmented reality trends in education: a systematic review of research and applications.

The 6th largest cluster (#5) has 48 members and a silhouette value of 0.86. It is labeled as ***virtual city environment*** by both LLR and TFIDF, and as *educational application* by MI. The most active citer to the cluster is 0.15 Portales,, C (2010) [augmented reality and photogrammetry: a synergy to visualize physical and virtual city environments](http://dx.doi.org/10.1016/j.isprsjprs.2009.10.001).

The 7th largest cluster (#6) has 20 members and a silhouette value of 0.997. It is labeled as ***knowledge-based augmented reality*** by both LLR and TFIDF, and as *...* by MI. The most active citer to the cluster is 1FEINER,, S (1993) [knowledge-based augmented reality](http://dx.doi.org/10.1145/159544.159587).

The 8th largest cluster (#7) has 19 members and a silhouette value of 0.926. It is labeled as ***hand-eye calibration*** by both LLR and TFIDF, and as *analysis* by MI. The most active citer to the cluster is 0.68 Lu,, CP (1996) [online computation of exterior orientation with application to hand-eye calibration](http://dx.doi.org/10.1016/0895-7177(96)00118-5).

**CITATION COUNTS**

The top ranked item by citation counts is Azuma RT (1997) in Cluster #0, with citation counts of **231**. The second one is Azuma R (2001) in Cluster #0, with citation counts of **220**. The third is Van Krevelen D W F (2010) in Cluster #5, with citation counts of **207**. The 4th is Lowe DG (2004) in Cluster #1, with citation counts of **157**. The 5th is Wu HK (2013) in Cluster #4, with citation counts of **144**. The 6th is Dunleavy M (2009) in Cluster #4, with citation counts of **122**. The 7th is Zhou F (2008) in Cluster #5, with citation counts of **118**. The 8th is Bay H (2008) in Cluster #1, with citation counts of **117**. The 9th is Newcombe RA (2011) in Cluster #1, with citation counts of **109**. The 10th is Carmigniani J (2011) in Cluster #5, with citation counts of **104**.

| **citation counts** | **references** | **cluster #** |
| --- | --- | --- |
| 231 | Azuma RT, 1997, PRESENCE-TELEOP VIRT, V6, P355 | 0 |
| 220 | Azuma R, 2001, IEEE COMPUT GRAPH, V21, P34 | 0 |
| 207 | Van Krevelen D W F, 2010, INT J VIRTUAL REALIT, V9, P1 | 5 |
| 157 | Lowe DG, 2004, INT J COMPUT VISION, V60, P91 | 1 |
| 144 | Wu HK, 2013, COMPUT EDUC, V62, P41 | 4 |
| 122 | Dunleavy M, 2009, J SCI EDUC TECHNOL, V18, P7 | 4 |
| 118 | Zhou F, 2008, INT SYM MIX AUGMENT, V, P193 | 5 |
| 117 | Bay H, 2008, COMPUT VIS IMAGE UND, V110, P346 | 1 |
| 109 | Newcombe RA, 2011, INT SYM MIX AUGMENT, V, P127 | 1 |
| 104 | Carmigniani J, 2011, MULTIMED TOOLS APPL, V51, P341 | 5 |

**BURSTS**

The top ranked item by bursts is Azuma RT (1997) in Cluster #0, with bursts of **101.64**. The second one is Azuma R (2001) in Cluster #0, with bursts of **84.23**. The third is Lowe DG (2004) in Cluster #1, with bursts of **64.07**. The 4th is Van Krevelen D W F (2010) in Cluster #5, with bursts of **50.99**. The 5th is Wu HK (2013) in Cluster #4, with bursts of **47.23**. The 6th is Hartley R (2000) in Cluster #0, with bursts of **37.71**. The 7th is Dunleavy M (2009) in Cluster #4, with bursts of **33.22**. The 8th is Kato H (1999) in Cluster #0, with bursts of **32.16**. The 9th is Newcombe RA (2011) in Cluster #1, with bursts of **29.72**. The 10th is Feiner S (1993) in Cluster #8, with bursts of **29.46**.

| **bursts** | **references** | **cluster #** |
| --- | --- | --- |
| 101.64 | Azuma RT, 1997, PRESENCE-TELEOP VIRT, V6, P355 | 0 |
| 84.23 | Azuma R, 2001, IEEE COMPUT GRAPH, V21, P34 | 0 |
| 64.07 | Lowe DG, 2004, INT J COMPUT VISION, V60, P91 | 1 |
| 50.99 | Van Krevelen D W F, 2010, INT J VIRTUAL REALIT, V9, P1 | 5 |
| 47.23 | Wu HK, 2013, COMPUT EDUC, V62, P41 | 4 |
| 37.71 | Hartley R, 2000, MULTIPLE VIEW GEOMET, V, P | 0 |
| 33.22 | Dunleavy M, 2009, J SCI EDUC TECHNOL, V18, P7 | 4 |
| 32.16 | Kato H, 1999, Proceedings 2nd IEEE and ACM International Workshop on Augmented Reality (IWAR99), V, P85 | 0 |
| 29.72 | Newcombe RA, 2011, INT SYM MIX AUGMENT, V, P127 | 1 |
| 29.46 | Feiner S, 1993, COMMUN ACM, V36, P53 | 8 |

**CENTRALITY**

The top ranked item by centrality is Azuma RT (1997) in Cluster #0, with centrality of **0.00**. The second one is Azuma R (2001) in Cluster #0, with centrality of **0.00**. The third is Lowe DG (2004) in Cluster #1, with centrality of **0.00**. The 4th is Van Krevelen D W F (2010) in Cluster #5, with centrality of **0.00**. The 5th is Wu HK (2013) in Cluster #4, with centrality of **0.00**. The 6th is Hartley R (2000) in Cluster #0, with centrality of **0.00**. The 7th is Dunleavy M (2009) in Cluster #4, with centrality of **0.00**. The 8th is Kato H (1999) in Cluster #0, with centrality of **0.00**. The 9th is Newcombe RA (2011) in Cluster #1, with centrality of **0.00**. The 10th is Feiner S (1993) in Cluster #8, with centrality of **0.00**.

| **centrality** | **references** | **cluster #** |
| --- | --- | --- |
| 0.00 | Azuma RT, 1997, PRESENCE-TELEOP VIRT, V6, P355 | 0 |
| 0.00 | Azuma R, 2001, IEEE COMPUT GRAPH, V21, P34 | 0 |
| 0.00 | Lowe DG, 2004, INT J COMPUT VISION, V60, P91 | 1 |
| 0.00 | Van Krevelen D W F, 2010, INT J VIRTUAL REALIT, V9, P1 | 5 |
| 0.00 | Wu HK, 2013, COMPUT EDUC, V62, P41 | 4 |
| 0.00 | Hartley R, 2000, MULTIPLE VIEW GEOMET, V, P | 0 |
| 0.00 | Dunleavy M, 2009, J SCI EDUC TECHNOL, V18, P7 | 4 |
| 0.00 | Kato H, 1999, Proceedings 2nd IEEE and ACM International Workshop on Augmented Reality (IWAR99), V, P85 | 0 |
| 0.00 | Newcombe RA, 2011, INT SYM MIX AUGMENT, V, P127 | 1 |
| 0.00 | Feiner S, 1993, COMMUN ACM, V36, P53 | 8 |

**SIGMA**

The top ranked item by sigma is Azuma RT (1997) in Cluster #0, with sigma of **1.00**. The second one is Azuma R (2001) in Cluster #0, with sigma of **1.00**. The third is Lowe DG (2004) in Cluster #1, with sigma of **1.00**. The 4th is Van Krevelen D W F (2010) in Cluster #5, with sigma of **1.00**. The 5th is Wu HK (2013) in Cluster #4, with sigma of **1.00**. The 6th is Hartley R (2000) in Cluster #0, with sigma of **1.00**. The 7th is Dunleavy M (2009) in Cluster #4, with sigma of **1.00**. The 8th is Kato H (1999) in Cluster #0, with sigma of **1.00**. The 9th is Newcombe RA (2011) in Cluster #1, with sigma of **1.00**. The 10th is Feiner S (1993) in Cluster #8, with sigma of **1.00**.

| **sigma** | **references** | **cluster #** |
| --- | --- | --- |
| 1.00 | Azuma RT, 1997, PRESENCE-TELEOP VIRT, V6, P355 | 0 |
| 1.00 | Azuma R, 2001, IEEE COMPUT GRAPH, V21, P34 | 0 |
| 1.00 | Lowe DG, 2004, INT J COMPUT VISION, V60, P91 | 1 |
| 1.00 | Van Krevelen D W F, 2010, INT J VIRTUAL REALIT, V9, P1 | 5 |
| 1.00 | Wu HK, 2013, COMPUT EDUC, V62, P41 | 4 |
| 1.00 | Hartley R, 2000, MULTIPLE VIEW GEOMET, V, P | 0 |
| 1.00 | Dunleavy M, 2009, J SCI EDUC TECHNOL, V18, P7 | 4 |
| 1.00 | Kato H, 1999, Proceedings 2nd IEEE and ACM International Workshop on Augmented Reality (IWAR99), V, P85 | 0 |
| 1.00 | Newcombe RA, 2011, INT SYM MIX AUGMENT, V, P127 | 1 |
| 1.00 | Feiner S, 1993, COMMUN ACM, V36, P53 | 8 |
