# Supplementary material for: The Past, Present, and Future of Virtual and Augmented Reality Research: A Network and Cluster Analysis of the Literature
Source: Front Psychol. 2018 Nov 6;9:2086. doi: 10.3389/fpsyg.2018.02086 (PMC6232426; doi:10.3389/fpsyg.2018.02086)
Supplement: Supplementary file 1 [file Data_Sheet_1.ZIP › NARRATIVES - Citations clusters VR.docx]

**NARRATIVES**

**MAJOR CLUSTERS**

The network is divided into **15** co-citation clusters. These clusters are labeled by index terms from their own citers. The largest **6** clusters are summarized.

**Table 1. Summary of the largest 6 clusters.**

| **ClusterID** | **Size** | **Silhouette** | **Label (TFIDF)** | **Label (LLR)** | **Label (MI)** | **mean(Citee Year)** |
| --- | --- | --- | --- | --- | --- | --- |
| 0 | 84 | 0.812 | (25.82) laparoscopic skill | training (143.21, 1.0E-4) | analysis | 2005 |
| 1 | 77 | 0.758 | (17.76) ergonomic | ergonomic (54.1, 1.0E-4) | defining virtual reality | 1992 |
| 2 | 62 | 0.992 | (24.5) gaming | stroke (82.9, 1.0E-4) | utility | 2007 |
| 3 | 61 | 0.758 | (15) reality | telemedical information society (34.85, 1.0E-4) | microstructure | 1994 |
| 4 | 56 | 0.934 | (25.4) therapy | treatment (109.92, 1.0E-4) | approache | 2008 |
| 5 | 49 | 0.885 | (16.03) reality | autistic children (29.81, 1.0E-4) | microstructure | 1992 |

The largest cluster (#0) has 84 members and a silhouette value of 0.812. It is labeled as *training* by LLR, *laparoscopic skill* by TFIDF, and *analysis* by MI. The most active citer to the cluster is 0.32 Palter,, VN (2010) [simulation in surgical education](http://dx.doi.org/10.1503/cmaj.091743).

The second largest cluster (#1) has 77 members and a silhouette value of 0.758. It is labeled as ***ergonomic*** by both LLR and TFIDF, and as *defining virtual reality* by MI. The most active citer to the cluster is0.12 Riva,, G (1999) [from technology to communication: psycho-social issues in developing virtual environments](http://dx.doi.org/10.1006/jvlc.1998.0110).

The third largest cluster (#2) has 62 members and a silhouette value of 0.992. It is labeled as *stroke* by LLR, *gaming* by TFIDF, and *utility* by MI. The most active citer to the cluster is 0.11 Lange,, BS (2010)[the potential of virtual reality and gaming to assist successful aging with disability](http://dx.doi.org/10.1016/j.pmr.2009.12.007).

The 4th largest cluster (#3) has 61 members and a silhouette value of 0.758. It is labeled as *telemedical information society* by LLR, *reality* by TFIDF, and *microstructure* by MI. The most active citer to the cluster is 0.13 Gorman,, PJ (1999) [simulation and virtual reality in surgical education - real or unreal?](http://dx.doi.org/10.1001/archsurg.134.11.1203).

The 5th largest cluster (#4) has 56 members and a silhouette value of 0.934. It is labeled as *treatment* by LLR, *therapy* by TFIDF, and *approache* by MI. The most active citer to the cluster is 0.18 De, Carvalho, MR (2010) [virtual reality as a mechanism for exposure therapy](http://dx.doi.org/10.3109/15622970802575985).

The 6th largest cluster (#5) has 49 members and a silhouette value of 0.885. It is labeled as *autistic children* by LLR, *reality* by TFIDF, and *microstructure* by MI. The most active citer to the cluster is 0.1BIOCCA,, F (1992) [communication within virtual reality - creating a space for research](http://dx.doi.org/10.1111/j.1460-2466.1992.tb00810.x).

**CITATION COUNTS**

The top ranked item by citation counts is Seymour NE (2002) in Cluster #0, with citation counts of **317**. The second one is Grantcharov TP (2004) in Cluster #0, with citation counts of **286**. The third is Holden MK (2005) in Cluster #2, with citation counts of **179**. The 4th is Gallagher AG (2005) in Cluster #0, with citation counts of **171**. The 5th is Ahlberg G (2007) in Cluster #0, with citation counts of **142**. The 6th is Parsons TD (2008) in Cluster #4, with citation counts of **136**. The 7th is Powers MB (2008) in Cluster #4, with citation counts of **134**. The 8th is Aggarwal R (2007) in Cluster #0, with citation counts of **121**. The 9th is Reznick RK (2006) in Cluster #0, with citation counts of **121**. The 10th is Munz Y (2004) in Cluster #0, with citation counts of **117**.

| **citation counts** | **references** | **cluster #** |
| --- | --- | --- |
| 317 | Seymour NE, 2002, ANN SURG, V236, P458 | 0 |
| 286 | Grantcharov TP, 2004, BRIT J SURG, V91, P146 | 0 |
| 179 | Holden MK, 2005, CYBERPSYCHOL BEHAV, V8, P187 | 2 |
| 171 | Gallagher AG, 2005, ANN SURG, V241, P364 | 0 |
| 142 | Ahlberg G, 2007, AM J SURG, V193, P797 | 0 |
| 136 | Parsons TD, 2008, J BEHAV THER EXP PSY, V39, P250 | 4 |
| 134 | Powers MB, 2008, J ANXIETY DISORD, V22, P561 | 4 |
| 121 | Aggarwal R, 2007, ANN SURG, V246, P771 | 0 |
| 121 | Reznick RK, 2006, NEW ENGL J MED, V355, P2664 | 0 |
| 117 | Munz Y, 2004, SURG ENDOSC, V18, P485 | 0 |

**BURSTS**

The top ranked item by bursts is Seymour NE (2002) in Cluster #0, with bursts of **88.93**. The second one is Grantcharov TP (2004) in Cluster #0, with bursts of **51.40**. The third is Saposnik G (2010) in Cluster #2, with bursts of **40.84**. The 4th is Rothbaum BO (1995) in Cluster #7, with bursts of **38.94**. The 5th is Holden MK (2005) in Cluster #2, with bursts of **37.52**. The 6th is Scott DJ (2000) in Cluster #0, with bursts of **33.39**. The 7th is Saposnik G (2011) in Cluster #2, with bursts of **33.33**. The 8th is Burdea GC (1996) in Cluster #3, with bursts of **32.42**. The 9th is Burdea G C (2003) in Cluster #22, with bursts of **31.30**. The 10th is Taffinder N (1998) in Cluster #6, with bursts of **30.96**.

| **bursts** | **references** | **cluster #** |
| --- | --- | --- |
| 88.93 | Seymour NE, 2002, ANN SURG, V236, P458 | 0 |
| 51.40 | Grantcharov TP, 2004, BRIT J SURG, V91, P146 | 0 |
| 40.84 | Saposnik G, 2010, STROKE, V41, P1477 | 2 |
| 38.94 | Rothbaum BO, 1995, AM J PSYCHIAT, V152, P626 | 7 |
| 37.52 | Holden MK, 2005, CYBERPSYCHOL BEHAV, V8, P187 | 2 |
| 33.39 | Scott DJ, 2000, J AM COLL SURGEONS, V191, P272 | 0 |
| 33.33 | Saposnik G, 2011, STROKE, V42, P1380 | 2 |
| 32.42 | Burdea GC, 1996, FORCE TOUCH FEEDBACK, V, P | 3 |
| 31.30 | Burdea G C, 2003, VIRTUAL REALITY TECH, V, P | 22 |
| 30.96 | Taffinder N, 1998, ST HEAL T, V50, P124 | 6 |

**CENTRALITY**

The top ranked item by centrality is Seymour NE (2002) in Cluster #0, with centrality of **0.00**. The second one is Grantcharov TP (2004) in Cluster #0, with centrality of **0.00**. The third is Saposnik G (2010) in Cluster #2, with centrality of **0.00**. The 4th is Rothbaum BO (1995) in Cluster #7, with centrality of **0.00**. The 5th is Holden MK (2005) in Cluster #2, with centrality of **0.00**. The 6th is Scott DJ (2000) in Cluster #0, with centrality of **0.00**. The 7th is Saposnik G (2011) in Cluster #2, with centrality of **0.00**. The 8th is Burdea GC (1996) in Cluster #3, with centrality of **0.00**. The 9th is Burdea G C (2003) in Cluster #22, with centrality of **0.00**. The 10th is Taffinder N (1998) in Cluster #6, with centrality of **0.00**.

| **centrality** | **references** | **cluster #** |
| --- | --- | --- |
| 0.00 | Seymour NE, 2002, ANN SURG, V236, P458 | 0 |
| 0.00 | Grantcharov TP, 2004, BRIT J SURG, V91, P146 | 0 |
| 0.00 | Saposnik G, 2010, STROKE, V41, P1477 | 2 |
| 0.00 | Rothbaum BO, 1995, AM J PSYCHIAT, V152, P626 | 7 |
| 0.00 | Holden MK, 2005, CYBERPSYCHOL BEHAV, V8, P187 | 2 |
| 0.00 | Scott DJ, 2000, J AM COLL SURGEONS, V191, P272 | 0 |
| 0.00 | Saposnik G, 2011, STROKE, V42, P1380 | 2 |
| 0.00 | Burdea GC, 1996, FORCE TOUCH FEEDBACK, V, P | 3 |
| 0.00 | Burdea G C, 2003, VIRTUAL REALITY TECH, V, P | 22 |
| 0.00 | Taffinder N, 1998, ST HEAL T, V50, P124 | 6 |

**SIGMA**

The top ranked item by sigma is Seymour NE (2002) in Cluster #0, with sigma of **1.00**. The second one is Grantcharov TP (2004) in Cluster #0, with sigma of **1.00**. The third is Saposnik G (2010) in Cluster #2, with sigma of **1.00**. The 4th is Rothbaum BO (1995) in Cluster #7, with sigma of **1.00**. The 5th is Holden MK (2005) in Cluster #2, with sigma of **1.00**. The 6th is Scott DJ (2000) in Cluster #0, with sigma of **1.00**. The 7th is Saposnik G (2011) in Cluster #2, with sigma of **1.00**. The 8th is Burdea GC (1996) in Cluster #3, with sigma of **1.00**. The 9th is Burdea G C (2003) in Cluster #22, with sigma of **1.00**. The 10th is Taffinder N (1998) in Cluster #6, with sigma of **1.00**.

| **sigma** | **references** | **cluster #** |
| --- | --- | --- |
| 1.00 | Seymour NE, 2002, ANN SURG, V236, P458 | 0 |
| 1.00 | Grantcharov TP, 2004, BRIT J SURG, V91, P146 | 0 |
| 1.00 | Saposnik G, 2010, STROKE, V41, P1477 | 2 |
| 1.00 | Rothbaum BO, 1995, AM J PSYCHIAT, V152, P626 | 7 |
| 1.00 | Holden MK, 2005, CYBERPSYCHOL BEHAV, V8, P187 | 2 |
| 1.00 | Scott DJ, 2000, J AM COLL SURGEONS, V191, P272 | 0 |
| 1.00 | Saposnik G, 2011, STROKE, V42, P1380 | 2 |
| 1.00 | Burdea GC, 1996, FORCE TOUCH FEEDBACK, V, P | 3 |
| 1.00 | Burdea G C, 2003, VIRTUAL REALITY TECH, V, P | 22 |
| 1.00 | Taffinder N, 1998, ST HEAL T, V50, P124 | 6 |
