# Supplementary material for: The Past, Present, and Future of Virtual and Augmented Reality Research: A Network and Cluster Analysis of the Literature
Source: Front Psychol. 2018 Nov 6;9:2086. doi: 10.3389/fpsyg.2018.02086 (PMC6232426; doi:10.3389/fpsyg.2018.02086)
Supplement: Supplementary file 1 [file Data_Sheet_1.ZIP › NARRATIVES - Cited Authors.docx]

**NARRATIVES**

**MAJOR CLUSTERS**

The network is divided into **12** co-citation clusters. These clusters are labeled by index terms from their own citers. The largest **4** clusters are summarized.

**Table 1. Summary of the largest 4 clusters.**

| **ClusterID** | **Size** | **Silhouette** | **Label (TFIDF)** | **Label (LLR)** | **Label (MI)** | **mean(Citee Year)** |
| --- | --- | --- | --- | --- | --- | --- |
| 0 | 82 | 0.844 | (19.46) volume | model deformation (51, 1.0E-4) | direct virtual-hand interface | 1997 |
| 1 | 79 | 0.698 | (20.05) reality | synergy (38.64, 1.0E-4) | 3-d tactile display | 1996 |
| 2 | 68 | 0.775 | (24.29) exposure therapy | treatment (130.16, 1.0E-4) | anticipating consequence | 2006 |
| 3 | 59 | 0.846 | (16.94) reality | virtual-reality (28.89, 1.0E-4) | direct virtual-hand interface | 1992 |

The largest cluster (#0) has 82 members and a silhouette value of 0.844. It is labeled as *model deformation* by LLR, *volume* by TFIDF, and *direct virtual-hand interface* by MI. The most active citer to the cluster is 0.09 Gorman,, PJ (1999) [simulation and virtual reality in surgical education - real or unreal?](http://dx.doi.org/10.1001/archsurg.134.11.1203).

The second largest cluster (#1) has 79 members and a silhouette value of 0.698. It is labeled as *synergy* by LLR, *reality* by TFIDF, and *3-d tactile display* by MI. The most active citer to the cluster is 0.11Burdea,, GC (1999) [invited review: the synergy between virtual reality and robotics](http://dx.doi.org/10.1109/70.768174).

The third largest cluster (#2) has 68 members and a silhouette value of 0.775. It is labeled as *treatment* by LLR, *exposure therapy* by TFIDF, and *anticipating consequence* by MI. The most active citer to the cluster is 0.12 Malbos,, E (2013) [virtual reality in the treatment of mental disorders](http://dx.doi.org/10.1016/j.lpm.2013.01.065).

The 4th largest cluster (#3) has 59 members and a silhouette value of 0.846. It is labeled as *virtual-reality* by LLR, *reality* by TFIDF, and *direct virtual-hand interface* by MI. The most active citer to the cluster is 0.05 BIOCCA,, F (1992) [virtual reality technology - a tutorial](http://dx.doi.org/10.1111/j.1460-2466.1992.tb00811.x).

**CITATION COUNTS**

The top ranked item by citation counts is Gallagher AG (2001) in Cluster #4, with citation counts of **694**. The second one is Seymour NE (2004) in Cluster #4, with citation counts of **668**. The third is Slater M (1999) in Cluster #2, with citation counts of **649**. The 4th is Grantcharov TP (2003) in Cluster #4, with citation counts of **563**. The 5th is Riva G (1999) in Cluster #2, with citation counts of **546**. The 6th is Aggarwal R (2006) in Cluster #4, with citation counts of **505**. The 7th is Satava RM (1994) in Cluster #0, with citation counts of **477**. The 8th is Witmer BG (2002) in Cluster #2, with citation counts of **454**. The 9th is Rothbaum BO (1996) in Cluster #2, with citation counts of **448**. The 10th is Cruz-neira C (1995) in Cluster #1, with citation counts of **416**.

| **citation counts** | **references** | **cluster #** |
| --- | --- | --- |
| 694 | Gallagher AG, 2001, SO, V, P | 4 |
| 668 | Seymour NE, 2004, SO, V, P | 4 |
| 649 | Slater M, 1999, SO, V, P | 2 |
| 563 | Grantcharov TP, 2003, SO, V, P | 4 |
| 546 | Riva G, 1999, SO, V, P | 2 |
| 505 | Aggarwal R, 2006, SO, V, P | 4 |
| 477 | Satava RM, 1994, SO, V, P | 0 |
| 454 | Witmer BG, 2002, SO, V, P | 2 |
| 448 | Rothbaum BO, 1996, SO, V, P | 2 |
| 416 | Cruz-neira C, 1995, SO, V, P | 1 |

**BURSTS**

The top ranked item by bursts is Saposnik G (2012) in Cluster #6, with bursts of **56.38**. The second one is Rheingold H (1991) in Cluster #1, with bursts of **33.18**. The third is Burdea G C (2006) in Cluster #55, with bursts of **33.17**. The 4th is Laver KE (2014) in Cluster #6, with bursts of **31.29**. The 5th is Satava RM (1994) in Cluster #0, with bursts of **30.65**. The 6th is Lange B (2014) in Cluster #6, with bursts of **30.20**. The 7th is Laver K (2015) in Cluster #6, with bursts of **29.52**. The 8th is Parsons TD (2009) in Cluster #2, with bursts of **29.22**. The 9th is Bohil CJ (2016) in Cluster #2, with bursts of **27.01**. The 10th is Larsen CR (2010) in Cluster #4, with bursts of **26.90**.

| **bursts** | **references** | **cluster #** |
| --- | --- | --- |
| 56.38 | Saposnik G, 2012, SO, V, P | 6 |
| 33.18 | Rheingold H, 1991, SO, V, P | 1 |
| 33.17 | Burdea G C, 2006, SO, V, P | 55 |
| 31.29 | Laver KE, 2014, SO, V, P | 6 |
| 30.65 | Satava RM, 1994, SO, V, P | 0 |
| 30.20 | Lange B, 2014, SO, V, P | 6 |
| 29.52 | Laver K, 2015, SO, V, P | 6 |
| 29.22 | Parsons TD, 2009, SO, V, P | 2 |
| 27.01 | Bohil CJ, 2016, SO, V, P | 2 |
| 26.90 | Larsen CR, 2010, SO, V, P | 4 |

**CENTRALITY**

The top ranked item by centrality is Saposnik G (2012) in Cluster #6, with centrality of **0.00**. The second one is Rheingold H (1991) in Cluster #1, with centrality of **0.00**. The third is Burdea G C (2006) in Cluster #55, with centrality of **0.00**. The 4th is Laver KE (2014) in Cluster #6, with centrality of **0.00**. The 5th is Satava RM (1994) in Cluster #0, with centrality of **0.00**. The 6th is Lange B (2014) in Cluster #6, with centrality of **0.00**. The 7th is Laver K (2015) in Cluster #6, with centrality of **0.00**. The 8th is Parsons TD (2009) in Cluster #2, with centrality of **0.00**. The 9th is Bohil CJ (2016) in Cluster #2, with centrality of **0.00**. The 10th is Larsen CR (2010) in Cluster #4, with centrality of **0.00**.

| **centrality** | **references** | **cluster #** |
| --- | --- | --- |
| 0.00 | Saposnik G, 2012, SO, V, P | 6 |
| 0.00 | Rheingold H, 1991, SO, V, P | 1 |
| 0.00 | Burdea G C, 2006, SO, V, P | 55 |
| 0.00 | Laver KE, 2014, SO, V, P | 6 |
| 0.00 | Satava RM, 1994, SO, V, P | 0 |
| 0.00 | Lange B, 2014, SO, V, P | 6 |
| 0.00 | Laver K, 2015, SO, V, P | 6 |
| 0.00 | Parsons TD, 2009, SO, V, P | 2 |
| 0.00 | Bohil CJ, 2016, SO, V, P | 2 |
| 0.00 | Larsen CR, 2010, SO, V, P | 4 |

**SIGMA**

The top ranked item by sigma is Saposnik G (2012) in Cluster #6, with sigma of **1.00**. The second one is Rheingold H (1991) in Cluster #1, with sigma of **1.00**. The third is Burdea G C (2006) in Cluster #55, with sigma of **1.00**. The 4th is Laver KE (2014) in Cluster #6, with sigma of **1.00**. The 5th is Satava RM (1994) in Cluster #0, with sigma of **1.00**. The 6th is Lange B (2014) in Cluster #6, with sigma of **1.00**. The 7th is Laver K (2015) in Cluster #6, with sigma of **1.00**. The 8th is Parsons TD (2009) in Cluster #2, with sigma of **1.00**. The 9th is Bohil CJ (2016) in Cluster #2, with sigma of **1.00**. The 10th is Larsen CR (2010) in Cluster #4, with sigma of **1.00**.

| **sigma** | **references** | **cluster #** |
| --- | --- | --- |
| 1.00 | Saposnik G, 2012, SO, V, P | 6 |
| 1.00 | Rheingold H, 1991, SO, V, P | 1 |
| 1.00 | Burdea G C, 2006, SO, V, P | 55 |
| 1.00 | Laver KE, 2014, SO, V, P | 6 |
| 1.00 | Satava RM, 1994, SO, V, P | 0 |
| 1.00 | Lange B, 2014, SO, V, P | 6 |
| 1.00 | Laver K, 2015, SO, V, P | 6 |
| 1.00 | Parsons TD, 2009, SO, V, P | 2 |
| 1.00 | Bohil CJ, 2016, SO, V, P | 2 |
| 1.00 | Larsen CR, 2010, SO, V, P | 4 |
