# Supplementary material for: The Past, Present, and Future of Virtual and Augmented Reality Research: A Network and Cluster Analysis of the Literature
Source: Front Psychol. 2018 Nov 6;9:2086. doi: 10.3389/fpsyg.2018.02086 (PMC6232426; doi:10.3389/fpsyg.2018.02086)
Supplement: Supplementary file 1 [file Data_Sheet_1.ZIP › NARRATIVES - Country.docx]

**NARRATIVES**

**MAJOR CLUSTERS**

The network is divided into **7** co-citation clusters. These clusters are labeled by index terms from their own citers. The largest **2** clusters are summarized.

**Table 1. Summary of the largest 2 clusters.**

| **ClusterID** | **Size** | **Silhouette** | **Label (TFIDF)** | **Label (LLR)** | **Label (MI)** | **mean(Citee Year)** |
| --- | --- | --- | --- | --- | --- | --- |
| 0 | 22 | 0.655 | (8.95) assembly | cybersickness (12.06, 0.001) | approach | 1998 |
| 1 | 20 | 0.67 | (13.56) assessment | effect (29.38, 1.0E-4) | approach | 1999 |

The largest cluster (#0) has 22 members and a silhouette value of 0.655. It is labeled as *cybersickness* by LLR, *assembly* by TFIDF, and *approach* by MI. The most active citer to the cluster is 0.09 Kosel,, J (2010) [anatomical study of the radius and center of curvature of the distal femoral condyle](http://dx.doi.org/10.1115/1.4002061).

The second largest cluster (#1) has 20 members and a silhouette value of 0.67. It is labeled as *effect* by LLR, *assessment* by TFIDF, and *approach* by MI. The most active citer to the cluster is 0.1Samaraweera,, G (2013) latency and avatars in virtual environments and the effects on gait for persons with mobility impairments.

**CITATION COUNTS**

The top ranked item by citation counts is USA (1992) in Cluster #1, with citation counts of **4921**. The second one is PEOPLES R CHINA (1996) in Cluster #0, with citation counts of **2384**. The third is ENGLAND (1993) in Cluster #4, with citation counts of **1497**. The 4th is GERMANY (1993) in Cluster #3, with citation counts of **1398**. The 5th is JAPAN (1994) in Cluster #3, with citation counts of **1106**. The 6th is CANADA (1993) in Cluster #0, with citation counts of **985**. The 7th is ITALY (1994) in Cluster #2, with citation counts of **934**. The 8th is FRANCE (1994) in Cluster #2, with citation counts of**875**. The 9th is SPAIN (1996) in Cluster #2, with citation counts of **740**. The 10th is SOUTH KOREA (1997) in Cluster #0, with citation counts of **595**.

| **citation counts** | **references** | **cluster #** |
| --- | --- | --- |
| 4921 | USA, 1992, SO, V, P | 1 |
| 2384 | PEOPLES R CHINA, 1996, SO, V, P | 0 |
| 1497 | ENGLAND, 1993, SO, V, P | 4 |
| 1398 | GERMANY, 1993, SO, V, P | 3 |
| 1106 | JAPAN, 1994, SO, V, P | 3 |
| 985 | CANADA, 1993, SO, V, P | 0 |
| 934 | ITALY, 1994, SO, V, P | 2 |
| 875 | FRANCE, 1994, SO, V, P | 2 |
| 740 | SPAIN, 1996, SO, V, P | 2 |
| 595 | SOUTH KOREA, 1997, SO, V, P | 0 |

**BURSTS**

| **bursts** | **references** | **cluster #** |
| --- | --- | --- |

**CENTRALITY**

The top ranked item by centrality is USA (1992) in Cluster #1, with centrality of **0.46**. The second one is ENGLAND (1993) in Cluster #4, with centrality of **0.31**. The third is GERMANY (1993) in Cluster #3, with centrality of **0.19**. The 4th is SWITZERLAND (1994) in Cluster #6, with centrality of **0.16**. The 5th is AUSTRALIA (1993) in Cluster #0, with centrality of **0.13**. The 6th is FRANCE (1994) in Cluster #2, with centrality of **0.11**. The 7th is NETHERLANDS (1993) in Cluster #5, with centrality of **0.11**. The 8th is SPAIN (1996) in Cluster #2, with centrality of **0.10**. The 9th is JAPAN (1994) in Cluster #3, with centrality of **0.09**. The 10th is CANADA (1993) in Cluster #0, with centrality of **0.08**.

| **centrality** | **references** | **cluster #** |
| --- | --- | --- |
| 0.46 | USA, 1992, SO, V, P | 1 |
| 0.31 | ENGLAND, 1993, SO, V, P | 4 |
| 0.19 | GERMANY, 1993, SO, V, P | 3 |
| 0.16 | SWITZERLAND, 1994, SO, V, P | 6 |
| 0.13 | AUSTRALIA, 1993, SO, V, P | 0 |
| 0.11 | FRANCE, 1994, SO, V, P | 2 |
| 0.11 | NETHERLANDS, 1993, SO, V, P | 5 |
| 0.10 | SPAIN, 1996, SO, V, P | 2 |
| 0.09 | JAPAN, 1994, SO, V, P | 3 |
| 0.08 | CANADA, 1993, SO, V, P | 0 |

**SIGMA**

The top ranked item by sigma is USA (1992) in Cluster #1, with sigma of **1.00**. The second one is ENGLAND (1993) in Cluster #4, with sigma of **1.00**. The third is GERMANY (1993) in Cluster #3, with sigma of **1.00**. The 4th is SWITZERLAND (1994) in Cluster #6, with sigma of **1.00**. The 5th is AUSTRALIA (1993) in Cluster #0, with sigma of **1.00**. The 6th is FRANCE (1994) in Cluster #2, with sigma of **1.00**. The 7th is NETHERLANDS (1993) in Cluster #5, with sigma of **1.00**. The 8th is SPAIN (1996) in Cluster #2, with sigma of **1.00**. The 9th is JAPAN (1994) in Cluster #3, with sigma of **1.00**. The 10th is CANADA (1993) in Cluster #0, with sigma of **1.00**.

| **sigma** | **references** | **cluster #** |
| --- | --- | --- |
| 1.00 | USA, 1992, SO, V, P | 1 |
| 1.00 | ENGLAND, 1993, SO, V, P | 4 |
| 1.00 | GERMANY, 1993, SO, V, P | 3 |
| 1.00 | SWITZERLAND, 1994, SO, V, P | 6 |
| 1.00 | AUSTRALIA, 1993, SO, V, P | 0 |
| 1.00 | FRANCE, 1994, SO, V, P | 2 |
| 1.00 | NETHERLANDS, 1993, SO, V, P | 5 |
| 1.00 | SPAIN, 1996, SO, V, P | 2 |
| 1.00 | JAPAN, 1994, SO, V, P | 3 |
| 1.00 | CANADA, 1993, SO, V, P | 0 |
