# Supplementary material for: The Past, Present, and Future of Virtual and Augmented Reality Research: A Network and Cluster Analysis of the Literature
Source: Front Psychol. 2018 Nov 6;9:2086. doi: 10.3389/fpsyg.2018.02086 (PMC6232426; doi:10.3389/fpsyg.2018.02086)
Supplement: Supplementary file 1 [file Data_Sheet_1.ZIP › NARRATIVES - Institutions.docx]

**NARRATIVES**

**MAJOR CLUSTERS**

The network is divided into **10** co-citation clusters. These clusters are labeled by index terms from their own citers. The largest **4** clusters are summarized.

**Table 1. Summary of the largest 4 clusters.**

| **ClusterID** | **Size** | **Silhouette** | **Label (TFIDF)** | **Label (LLR)** | **Label (MI)** | **mean(Citee Year)** |
| --- | --- | --- | --- | --- | --- | --- |
| 0 | 14 | 0.904 | (15.35) ter | html5 (8.67, 0.005) | surgery | 2006 |
| 1 | 14 | 0.876 | (17.54) virtual reality | building (9.06, 0.005) | training system | 2000 |
| 2 | 13 | 0.959 | (16.39) shape | shape (27.59, 1.0E-4) | environment | 2007 |
| 3 | 12 | 0.811 | (16.57) virtual reality | locomotion (8.81, 0.005) | virtual-reality environment | 2003 |

The largest cluster (#0) has 14 members and a silhouette value of 0.904. It is labeled as *html5* by LLR, *ter* by TFIDF, and *surgery* by MI. The most active citer to the cluster is 0.14 Shaffer,, DM (2013) [chasin' choppers: using unpredictable trajectories to test theories of object interception](http://dx.doi.org/10.3758/s13414-013-0500-7).

The second largest cluster (#1) has 14 members and a silhouette value of 0.876. It is labeled as *building* by LLR, *virtual reality* by TFIDF, and *training system* by MI. The most active citer to the cluster is 0.14Ellis,, CD (1999) qualitative spatial representation for situational awareness and spatial decision support.

The third largest cluster (#2) has 13 members and a silhouette value of 0.959. It is labeled as ***shape*** by both LLR and TFIDF, and as *environment* by MI. The most active citer to the cluster is 0.15Linkenauger,, SA (2013) [welcome to wonderland: the influence of the size and shape of a virtual hand on the perceived size and shape of virtual objects](http://dx.doi.org/10.1371/journal.pone.0068594).

The 4th largest cluster (#3) has 12 members and a silhouette value of 0.811. It is labeled as *locomotion* by LLR, *virtual reality* by TFIDF, and *virtual-reality environment* by MI. The most active citer to the cluster is 0.08 Abboudi,, H (2013) [current status of validation for robotic surgery simulators a systematic review](http://dx.doi.org/10.1111/j.1464-410X.2012.11270.x).

**CITATION COUNTS**

The top ranked item by citation counts is Univ Illinois (1998) in Cluster #1, with citation counts of **159**. The second one is Univ So Calif (1999) in Cluster #6, with citation counts of **147**. The third is Univ Washington (1997) in Cluster #1, with citation counts of **146**. The 4th is Delft Univ Technol (1999) in Cluster #7, with citation counts of **129**. The 5th is Univ Toronto (2002) in Cluster #0, with citation counts of **125**. The 6th is IRCCS Ist Auxol Italiano (1999) in Cluster #3, with citation counts of **125**. The 7th is Univ London Imperial Coll Sci Technol & Med (2001) in Cluster #3, with citation counts of **125**. The 8th is Iowa State Univ (1998) in Cluster #189, with citation counts of **116**. The 9th is Chinese Acad Sci (2000) in Cluster #4, with citation counts of **104**. The 10th is McGill Univ (1998) in Cluster #0, with citation counts of **103**.

| **citation counts** | **references** | **cluster #** |
| --- | --- | --- |
| 159 | Univ Illinois, 1998, SO, V, P | 1 |
| 147 | Univ So Calif, 1999, SO, V, P | 6 |
| 146 | Univ Washington, 1997, SO, V, P | 1 |
| 129 | Delft Univ Technol, 1999, SO, V, P | 7 |
| 125 | Univ Toronto, 2002, SO, V, P | 0 |
| 125 | IRCCS Ist Auxol Italiano, 1999, SO, V, P | 3 |
| 125 | Univ London Imperial Coll Sci Technol & Med, 2001, SO, V, P | 3 |
| 116 | Iowa State Univ, 1998, SO, V, P | 189 |
| 104 | Chinese Acad Sci, 2000, SO, V, P | 4 |
| 103 | McGill Univ, 1998, SO, V, P | 0 |

**BURSTS**

The top ranked item by bursts is Univ Barcelona (2010) in Cluster #3, with bursts of **18.71**. The second one is Univ Illinois (1998) in Cluster #1, with bursts of **14.56**. The third is Univ Texas (2000) in Cluster #8, with bursts of **12.22**. The 4th is Univ Dortmund (1998) in Cluster #84, with bursts of **11.23**. The 5th is Univ Wurzburg (2012) in Cluster #2, with bursts of **11.07**. The 6th is Hong Kong Univ Sci & Technol (1997) in Cluster #209, with bursts of **10.66**. The 7th is Univ Washington (1997) in Cluster #1, with bursts of **10.31**. The 8th is Stanford Univ (1999) in Cluster #6, with bursts of **9.90**. The 9th is Tsing Hua Univ (1997) in Cluster #195, with bursts of **9.57**. The 10th is Wuhan Univ (2006) in Cluster #198, with bursts of **9.54**.

| **bursts** | **references** | **cluster #** |
| --- | --- | --- |
| 18.71 | Univ Barcelona, 2010, SO, V, P | 3 |
| 14.56 | Univ Illinois, 1998, SO, V, P | 1 |
| 12.22 | Univ Texas, 2000, SO, V, P | 8 |
| 11.23 | Univ Dortmund, 1998, SO, V, P | 84 |
| 11.07 | Univ Wurzburg, 2012, SO, V, P | 2 |
| 10.66 | Hong Kong Univ Sci & Technol, 1997, SO, V, P | 209 |
| 10.31 | Univ Washington, 1997, SO, V, P | 1 |
| 9.90 | Stanford Univ, 1999, SO, V, P | 6 |
| 9.57 | Tsing Hua Univ, 1997, SO, V, P | 195 |
| 9.54 | Wuhan Univ, 2006, SO, V, P | 198 |

**CENTRALITY**

The top ranked item by centrality is Univ Barcelona (2010) in Cluster #3, with centrality of **0.00**. The second one is Univ Illinois (1998) in Cluster #1, with centrality of **0.00**. The third is Univ Texas (2000) in Cluster #8, with centrality of **0.00**. The 4th is Univ Dortmund (1998) in Cluster #84, with centrality of **0.00**. The 5th is Univ Wurzburg (2012) in Cluster #2, with centrality of **0.00**. The 6th is Hong Kong Univ Sci & Technol (1997) in Cluster #209, with centrality of **0.00**. The 7th is Univ Washington (1997) in Cluster #1, with centrality of **0.00**. The 8th is Stanford Univ (1999) in Cluster #6, with centrality of **0.00**. The 9th is Tsing Hua Univ (1997) in Cluster #195, with centrality of **0.00**. The 10th is Wuhan Univ (2006) in Cluster #198, with centrality of **0.00**.

| **centrality** | **references** | **cluster #** |
| --- | --- | --- |
| 0.00 | Univ Barcelona, 2010, SO, V, P | 3 |
| 0.00 | Univ Illinois, 1998, SO, V, P | 1 |
| 0.00 | Univ Texas, 2000, SO, V, P | 8 |
| 0.00 | Univ Dortmund, 1998, SO, V, P | 84 |
| 0.00 | Univ Wurzburg, 2012, SO, V, P | 2 |
| 0.00 | Hong Kong Univ Sci & Technol, 1997, SO, V, P | 209 |
| 0.00 | Univ Washington, 1997, SO, V, P | 1 |
| 0.00 | Stanford Univ, 1999, SO, V, P | 6 |
| 0.00 | Tsing Hua Univ, 1997, SO, V, P | 195 |
| 0.00 | Wuhan Univ, 2006, SO, V, P | 198 |

**SIGMA**

The top ranked item by sigma is Univ Barcelona (2010) in Cluster #3, with sigma of **1.00**. The second one is Univ Illinois (1998) in Cluster #1, with sigma of **1.00**. The third is Univ Texas (2000) in Cluster #8, with sigma of **1.00**. The 4th is Univ Dortmund (1998) in Cluster #84, with sigma of **1.00**. The 5th is Univ Wurzburg (2012) in Cluster #2, with sigma of **1.00**. The 6th is Hong Kong Univ Sci & Technol (1997) in Cluster #209, with sigma of **1.00**. The 7th is Univ Washington (1997) in Cluster #1, with sigma of **1.00**. The 8th is Stanford Univ (1999) in Cluster #6, with sigma of **1.00**. The 9th is Tsing Hua Univ (1997) in Cluster #195, with sigma of **1.00**. The 10th is Wuhan Univ (2006) in Cluster #198, with sigma of **1.00**.

| **sigma** | **references** | **cluster #** |
| --- | --- | --- |
| 1.00 | Univ Barcelona, 2010, SO, V, P | 3 |
| 1.00 | Univ Illinois, 1998, SO, V, P | 1 |
| 1.00 | Univ Texas, 2000, SO, V, P | 8 |
| 1.00 | Univ Dortmund, 1998, SO, V, P | 84 |
| 1.00 | Univ Wurzburg, 2012, SO, V, P | 2 |
| 1.00 | Hong Kong Univ Sci & Technol, 1997, SO, V, P | 209 |
| 1.00 | Univ Washington, 1997, SO, V, P | 1 |
| 1.00 | Stanford Univ, 1999, SO, V, P | 6 |
| 1.00 | Tsing Hua Univ, 1997, SO, V, P | 195 |
| 1.00 | Wuhan Univ, 2006, SO, V, P | 198 |
