# Supplementary material for: The Past, Present, and Future of Virtual and Augmented Reality Research: A Network and Cluster Analysis of the Literature
Source: Front Psychol. 2018 Nov 6;9:2086. doi: 10.3389/fpsyg.2018.02086 (PMC6232426; doi:10.3389/fpsyg.2018.02086)
Supplement: Supplementary file 1 [file Data_Sheet_1.ZIP › NARRATIVES - Journals.docx]

**NARRATIVES**

**MAJOR CLUSTERS**

The network is divided into **9** co-citation clusters. These clusters are labeled by index terms from their own citers. The largest **3** clusters are summarized.

**Table 1. Summary of the largest 3 clusters.**

| **ClusterID** | **Size** | **Silhouette** | **Label (TFIDF)** | **Label (LLR)** | **Label (MI)** | **mean(Citee Year)** |
| --- | --- | --- | --- | --- | --- | --- |
| 0 | 56 | 0.853 | (21.76) collision detection | computer-vision-enabled augmented reality (36.52, 1.0E-4) | engineering development | 1996 |
| 1 | 47 | 0.872 | (17.48) reality | parkinsons disease (60.82, 1.0E-4) | fmri study | 1999 |
| 2 | 40 | 0.896 | (16.83) routine use | future possibilities (44.71, 1.0E-4) | comparative research | 1997 |

The largest cluster (#0) has 56 members and a silhouette value of 0.853. It is labeled as *computer-vision-enabled augmented reality* by LLR, *collision detection* by TFIDF, and *engineering development* by MI. The most active citer to the cluster is 0.21 Berger,, JW (1999) [computer-vision-enabled augmented reality fundus biomicroscopy](http://dx.doi.org/10.1016/S0161-6420(99)90404-9).

The second largest cluster (#1) has 47 members and a silhouette value of 0.872. It is labeled as *parkinsons disease* by LLR, *reality* by TFIDF, and *fmri study* by MI. The most active citer to the cluster is 0.26Maguire,, EA (1999) [human spatial navigation: cognitive maps, sexual dimorphism, and neural substrates](http://dx.doi.org/10.1016/S0959-4388(99)80023-3).

The third largest cluster (#2) has 40 members and a silhouette value of 0.896. It is labeled as *future possibilities* by LLR, *routine use* by TFIDF, and *comparative research* by MI. The most active citer to the cluster is 0.22 FARRELL,, AD (1991) computers and behavioral-assessment - current applications, future possibilities, and obstacles to routine use.

**CITATION COUNTS**

The top ranked item by citation counts is Presence-teleop VIRT (1992) in Cluster #3, with citation counts of **2689**. The second one is Cyberpsychol Behav (2000) in Cluster #2, with citation counts of **1884**. The third is Ieee COMPUT GRAPH (1993) in Cluster #0, with citation counts of **1873**. The 4th is St HEAL T (1997) in Cluster #5, with citation counts of **1371**. The 5th is Surg ENDOSC (2003) in Cluster #5, with citation counts of **1369**. The 6th is Ann SURG (2000) in Cluster #5, with citation counts of **1325**. The 7th is Lect NOTES COMPUT SC (1998) in Cluster #0, with citation counts of **1311**. The 8th is SCIENCE (1994) in Cluster #1, with citation counts of **1249**. The 9th is Am J SURG (2000) in Cluster #5, with citation counts of **1233**. The 10th is NATURE (1995) in Cluster #1, with citation counts of**1122**.

| **citation counts** | **references** | **cluster #** |
| --- | --- | --- |
| 2689 | Presence-teleop VIRT, 1992, PRESENCE-TELEOP VIRT, V, P | 3 |
| 1884 | Cyberpsychol Behav, 2000, Cyberpsychol Behav, V, P | 2 |
| 1873 | Ieee COMPUT GRAPH, 1993, IEEE COMPUT GRAPH, V, P | 0 |
| 1371 | St HEAL T, 1997, ST HEAL T, V, P | 5 |
| 1369 | Surg ENDOSC, 2003, SURG ENDOSC, V, P | 5 |
| 1325 | Ann SURG, 2000, ANN SURG, V, P | 5 |
| 1311 | Lect NOTES COMPUT SC, 1998, LECT NOTES COMPUT SC, V, P | 0 |
| 1249 | SCIENCE, 1994, SCIENCE, V, P | 1 |
| 1233 | Am J SURG, 2000, AM J SURG, V, P | 5 |
| 1122 | NATURE, 1995, NATURE, V, P | 1 |

**BURSTS**

| **bursts** | **references** | **cluster #** |
| --- | --- | --- |

**CENTRALITY**

The top ranked item by centrality is Presence-teleop VIRT (1992) in Cluster #3, with centrality of **0.00**. The second one is Cyberpsychol Behav (2000) in Cluster #2, with centrality of **0.00**. The third is Ieee COMPUT GRAPH (1993) in Cluster #0, with centrality of **0.00**. The 4th is St HEAL T (1997) in Cluster #5, with centrality of **0.00**. The 5th is Surg ENDOSC (2003) in Cluster #5, with centrality of **0.00**. The 6th is Ann SURG (2000) in Cluster #5, with centrality of **0.00**. The 7th is Lect NOTES COMPUT SC (1998) in Cluster #0, with centrality of **0.00**. The 8th is SCIENCE (1994) in Cluster #1, with centrality of**0.00**. The 9th is Am J SURG (2000) in Cluster #5, with centrality of **0.00**. The 10th is NATURE (1995) in Cluster #1, with centrality of **0.00**.

| **centrality** | **references** | **cluster #** |
| --- | --- | --- |
| 0.00 | Presence-teleop VIRT, 1992, PRESENCE-TELEOP VIRT, V, P | 3 |
| 0.00 | Cyberpsychol Behav, 2000, Cyberpsychol Behav, V, P | 2 |
| 0.00 | Ieee COMPUT GRAPH, 1993, IEEE COMPUT GRAPH, V, P | 0 |
| 0.00 | St HEAL T, 1997, ST HEAL T, V, P | 5 |
| 0.00 | Surg ENDOSC, 2003, SURG ENDOSC, V, P | 5 |
| 0.00 | Ann SURG, 2000, ANN SURG, V, P | 5 |
| 0.00 | Lect NOTES COMPUT SC, 1998, LECT NOTES COMPUT SC, V, P | 0 |
| 0.00 | SCIENCE, 1994, SCIENCE, V, P | 1 |
| 0.00 | Am J SURG, 2000, AM J SURG, V, P | 5 |
| 0.00 | NATURE, 1995, NATURE, V, P | 1 |

**SIGMA**

The top ranked item by sigma is Presence-teleop VIRT (1992) in Cluster #3, with sigma of **1.00**. The second one is Cyberpsychol Behav (2000) in Cluster #2, with sigma of **1.00**. The third is Ieee COMPUT GRAPH (1993) in Cluster #0, with sigma of **1.00**. The 4th is St HEAL T (1997) in Cluster #5, with sigma of **1.00**. The 5th is Surg ENDOSC (2003) in Cluster #5, with sigma of **1.00**. The 6th is Ann SURG (2000) in Cluster #5, with sigma of **1.00**. The 7th is Lect NOTES COMPUT SC (1998) in Cluster #0, with sigma of **1.00**. The 8th is SCIENCE (1994) in Cluster #1, with sigma of **1.00**. The 9th is Am J SURG (2000) in Cluster #5, with sigma of **1.00**. The 10th is NATURE (1995) in Cluster #1, with sigma of **1.00**.

| **sigma** | **references** | **cluster #** |
| --- | --- | --- |
| 1.00 | Presence-teleop VIRT, 1992, PRESENCE-TELEOP VIRT, V, P | 3 |
| 1.00 | Cyberpsychol Behav, 2000, Cyberpsychol Behav, V, P | 2 |
| 1.00 | Ieee COMPUT GRAPH, 1993, IEEE COMPUT GRAPH, V, P | 0 |
| 1.00 | St HEAL T, 1997, ST HEAL T, V, P | 5 |
| 1.00 | Surg ENDOSC, 2003, SURG ENDOSC, V, P | 5 |
| 1.00 | Ann SURG, 2000, ANN SURG, V, P | 5 |
| 1.00 | Lect NOTES COMPUT SC, 1998, LECT NOTES COMPUT SC, V, P | 0 |
| 1.00 | SCIENCE, 1994, SCIENCE, V, P | 1 |
| 1.00 | Am J SURG, 2000, AM J SURG, V, P | 5 |
| 1.00 | NATURE, 1995, NATURE, V, P | 1 |
