# Supplementary material for: The Past, Present, and Future of Virtual and Augmented Reality Research: A Network and Cluster Analysis of the Literature
Source: Front Psychol. 2018 Nov 6;9:2086. doi: 10.3389/fpsyg.2018.02086 (PMC6232426; doi:10.3389/fpsyg.2018.02086)
Supplement: Supplementary file 1 [file Data_Sheet_1.ZIP › NARRATIVES - Keywords.docx]

**NARRATIVES**

**MAJOR CLUSTERS**

The network is divided into **11** co-citation clusters. These clusters are labeled by index terms from their own citers. The largest **4** clusters are summarized.

**Table 1. Summary of the largest 4 clusters.**

| **ClusterID** | **Size** | **Silhouette** | **Label (TFIDF)** | **Label (LLR)** | **Label (MI)** | **mean(Citee Year)** |
| --- | --- | --- | --- | --- | --- | --- |
| 0 | 59 | 0.503 | (11.01) visual illusion | virtual environment (33.45, 1.0E-4) | computer-enhanced therapy | 1996 |
| 1 | 47 | 0.659 | (11.98) balance | research (41.42, 1.0E-4) | counsellor | 1995 |
| 2 | 44 | 0.744 | (15.76) skill | training (40.9, 1.0E-4) | information technology | 2000 |
| 3 | 39 | 0.722 | (10.07) retrieval | deepmatrix (21.51, 1.0E-4) | geovr | 1994 |

The largest cluster (#0) has 59 members and a silhouette value of 0.503. It is labeled as *virtual environment* by LLR, *visual illusion* by TFIDF, and *computer-enhanced therapy* by MI. The most active citer to the cluster is 0.08 Idesawa,, M (1999) newly found visual illusions and 3-d display.

The second largest cluster (#1) has 47 members and a silhouette value of 0.659. It is labeled as *research* by LLR, *balance* by TFIDF, and *counsellor* by MI. The most active citer to the cluster is 0.06 Berger,, JW (1999) [image-guided macular laser therapy: design considerations and progress towards implementation](http://dx.doi.org/10.1117/12.350586).

The third largest cluster (#2) has 44 members and a silhouette value of 0.744. It is labeled as *training* by LLR, *skill* by TFIDF, and *information technology* by MI. The most active citer to the cluster is 0.16Sharma,, M (2013) [basic laparoscopic skills training using fresh frozen cadaver: a randomized controlled trial](http://dx.doi.org/10.1016/j.amjsurg.2012.10.037).

The 4th largest cluster (#3) has 39 members and a silhouette value of 0.722. It is labeled as *deepmatrix* by LLR, *retrieval* by TFIDF, and *geovr* by MI. The most active citer to the cluster is 0.1 Orford,, S (1999) geography: information visualization in the social sciences - a state-of-the-art review.

**CITATION COUNTS**

The top ranked item by citation counts is virtual reality (1991) in Cluster #1, with citation counts of **8674**. The second one is simulation (1992) in Cluster #2, with citation counts of **1258**. The third is performance (1998) in Cluster #2, with citation counts of **1086**. The 4th is environment (1993) in Cluster #0, with citation counts of **1048**. The 5th is system (1991) in Cluster #0, with citation counts of **805**. The 6th is rehabilitation (1998) in Cluster #4, with citation counts of **731**. The 7th is surgery (1993) in Cluster #2, with citation counts of **693**. The 8th is education (1998) in Cluster #2, with citation counts of**626**. The 9th is model (1995) in Cluster #0, with citation counts of **621**. The 10th is virtual environment (1995) in Cluster #0, with citation counts of **558**.

| **citation counts** | **references** | **cluster #** |
| --- | --- | --- |
| 8674 | virtual reality, 1991, SO, V, P | 1 |
| 1258 | simulation, 1992, SO, V, P | 2 |
| 1086 | performance, 1998, SO, V, P | 2 |
| 1048 | environment, 1993, SO, V, P | 0 |
| 805 | system, 1991, SO, V, P | 0 |
| 731 | rehabilitation, 1998, SO, V, P | 4 |
| 693 | surgery, 1993, SO, V, P | 2 |
| 626 | education, 1998, SO, V, P | 2 |
| 621 | model, 1995, SO, V, P | 0 |
| 558 | virtual environment, 1995, SO, V, P | 0 |

**BURSTS**

| **bursts** | **references** | **cluster #** |
| --- | --- | --- |

**CENTRALITY**

The top ranked item by centrality is virtual reality (1991) in Cluster #1, with centrality of **0.65**. The second one is computer (1991) in Cluster #6, with centrality of **0.14**. The third is system (1991) in Cluster #0, with centrality of **0.12**. The 4th is surgery (1993) in Cluster #2, with centrality of **0.10**. The 5th is visualization (1993) in Cluster #5, with centrality of **0.10**. The 6th is multimedia (1992) in Cluster #3, with centrality of **0.10**. The 7th is design (1993) in Cluster #0, with centrality of **0.09**. The 8th is information (1996) in Cluster #0, with centrality of **0.09**. The 9th is simulation (1992) in Cluster #2, with centrality of **0.08**. The 10th is environment (1993) in Cluster #0, with centrality of **0.08**.

| **centrality** | **references** | **cluster #** |
| --- | --- | --- |
| 0.65 | virtual reality, 1991, SO, V, P | 1 |
| 0.14 | computer, 1991, SO, V, P | 6 |
| 0.12 | system, 1991, SO, V, P | 0 |
| 0.10 | surgery, 1993, SO, V, P | 2 |
| 0.10 | visualization, 1993, SO, V, P | 5 |
| 0.10 | multimedia, 1992, SO, V, P | 3 |
| 0.09 | design, 1993, SO, V, P | 0 |
| 0.09 | information, 1996, SO, V, P | 0 |
| 0.08 | simulation, 1992, SO, V, P | 2 |
| 0.08 | environment, 1993, SO, V, P | 0 |

**SIGMA**

The top ranked item by sigma is virtual reality (1991) in Cluster #1, with sigma of **1.00**. The second one is computer (1991) in Cluster #6, with sigma of **1.00**. The third is system (1991) in Cluster #0, with sigma of **1.00**. The 4th is surgery (1993) in Cluster #2, with sigma of **1.00**. The 5th is visualization (1993) in Cluster #5, with sigma of **1.00**. The 6th is multimedia (1992) in Cluster #3, with sigma of **1.00**. The 7th is design (1993) in Cluster #0, with sigma of **1.00**. The 8th is information (1996) in Cluster #0, with sigma of **1.00**. The 9th is simulation (1992) in Cluster #2, with sigma of **1.00**. The 10th is environment (1993) in Cluster #0, with sigma of **1.00**.

| **sigma** | **references** | **cluster #** |
| --- | --- | --- |
| 1.00 | virtual reality, 1991, SO, V, P | 1 |
| 1.00 | computer, 1991, SO, V, P | 6 |
| 1.00 | system, 1991, SO, V, P | 0 |
| 1.00 | surgery, 1993, SO, V, P | 2 |
| 1.00 | visualization, 1993, SO, V, P | 5 |
| 1.00 | multimedia, 1992, SO, V, P | 3 |
| 1.00 | design, 1993, SO, V, P | 0 |
| 1.00 | information, 1996, SO, V, P | 0 |
| 1.00 | simulation, 1992, SO, V, P | 2 |
| 1.00 | environment, 1993, SO, V, P | 0 |
