# Supplementary material for: The Past, Present, and Future of Virtual and Augmented Reality Research: A Network and Cluster Analysis of the Literature
Source: Front Psychol. 2018 Nov 6;9:2086. doi: 10.3389/fpsyg.2018.02086 (PMC6232426; doi:10.3389/fpsyg.2018.02086)
Supplement: Supplementary file 1 [file Data_Sheet_1.ZIP › Top 24 Countries with Strongest Citation Bursts.docx]

**Top 24 Countries with Strongest Citation Bursts**

| **Countries** | **Year** | **Strength** | **Begin** | **End** | **1990 - 2016** |
| --- | --- | --- | --- | --- | --- |
| USA | 1990 | 85.029 | **1990** | 1997 | ▃▃▃▃▃▃▃▃▂▂▂▂▂▂▂▂▂▂▂▂▂▂▂▂▂▂▂ |
| SCOTLAND | 1990 | 12.6189 | **1993** | 2001 | ▂▂▂▃▃▃▃▃▃▃▃▃▂▂▂▂▂▂▂▂▂▂▂▂▂▂▂ |
| HONG KONG | 1990 | 14.214 | **1996** | 1999 | ▂▂▂▂▂▂▃▃▃▃▂▂▂▂▂▂▂▂▂▂▂▂▂▂▂▂▂ |
| FINLAND | 1990 | 8.0553 | **1997** | 2001 | ▂▂▂▂▂▂▂▃▃▃▃▃▂▂▂▂▂▂▂▂▂▂▂▂▂▂▂ |
| BELGIUM | 1990 | 3.6845 | **1998** | 1999 | ▂▂▂▂▂▂▂▂▃▃▂▂▂▂▂▂▂▂▂▂▂▂▂▂▂▂▂ |
| GERMANY | 1990 | 22.3695 | **1998** | 2000 | ▂▂▂▂▂▂▂▂▃▃▃▂▂▂▂▂▂▂▂▂▂▂▂▂▂▂▂ |
| JAPAN | 1990 | 31.7614 | **1998** | 2000 | ▂▂▂▂▂▂▂▂▃▃▃▂▂▂▂▂▂▂▂▂▂▂▂▂▂▂▂ |
| RUSSIA | 1990 | 5.0944 | **1998** | 1999 | ▂▂▂▂▂▂▂▂▃▃▂▂▂▂▂▂▂▂▂▂▂▂▂▂▂▂▂ |
| SINGAPORE | 1990 | 4.8067 | **2000** | 2004 | ▂▂▂▂▂▂▂▂▂▂▃▃▃▃▃▂▂▂▂▂▂▂▂▂▂▂▂ |
| SWEDEN | 1990 | 4.9861 | **2001** | 2004 | ▂▂▂▂▂▂▂▂▂▂▂▃▃▃▃▂▂▂▂▂▂▂▂▂▂▂▂ |
| CROATIA | 1990 | 4.3289 | **2002** | 2004 | ▂▂▂▂▂▂▂▂▂▂▂▂▃▃▃▂▂▂▂▂▂▂▂▂▂▂▂ |
| MALAYSIA | 1990 | 4.986 | **2008** | 2009 | ▂▂▂▂▂▂▂▂▂▂▂▂▂▂▂▂▂▂▃▃▂▂▂▂▂▂▂ |
| ROMANIA | 1990 | 17.1618 | **2008** | 2014 | ▂▂▂▂▂▂▂▂▂▂▂▂▂▂▂▂▂▂▃▃▃▃▃▃▃▂▂ |
| PORTUGAL | 1990 | 7.273 | **2010** | 2014 | ▂▂▂▂▂▂▂▂▂▂▂▂▂▂▂▂▂▂▂▂▃▃▃▃▃▂▂ |
| TUNISIA | 1990 | 3.9191 | **2010** | 2013 | ▂▂▂▂▂▂▂▂▂▂▂▂▂▂▂▂▂▂▂▂▃▃▃▃▂▂▂ |
| IRELAND | 1990 | 3.9756 | **2011** | 2012 | ▂▂▂▂▂▂▂▂▂▂▂▂▂▂▂▂▂▂▂▂▂▃▃▂▂▂▂ |
| SLOVAKIA | 1990 | 12.5456 | **2011** | 2014 | ▂▂▂▂▂▂▂▂▂▂▂▂▂▂▂▂▂▂▂▂▂▃▃▃▃▂▂ |
| IRAN | 1990 | 3.9689 | **2011** | 2012 | ▂▂▂▂▂▂▂▂▂▂▂▂▂▂▂▂▂▂▂▂▂▃▃▂▂▂▂ |
| SRI LANKA | 1990 | 3.8387 | **2012** | 2013 | ▂▂▂▂▂▂▂▂▂▂▂▂▂▂▂▂▂▂▂▂▂▂▃▃▂▂▂ |
| SAUDI ARABIA | 1990 | 7.6559 | **2012** | 2016 | ▂▂▂▂▂▂▂▂▂▂▂▂▂▂▂▂▂▂▂▂▂▂▃▃▃▃▃ |
| INDIA | 1990 | 7.9201 | **2012** | 2016 | ▂▂▂▂▂▂▂▂▂▂▂▂▂▂▂▂▂▂▂▂▂▂▃▃▃▃▃ |
| SPAIN | 1990 | 9.3851 | **2012** | 2013 | ▂▂▂▂▂▂▂▂▂▂▂▂▂▂▂▂▂▂▂▂▂▂▃▃▂▂▂ |
| BRAZIL | 1990 | 10.8048 | **2013** | 2014 | ▂▂▂▂▂▂▂▂▂▂▂▂▂▂▂▂▂▂▂▂▂▂▂▃▃▂▂ |
| DENMARK | 1990 | 15.4394 | **2014** | 2016 | ▂▂▂▂▂▂▂▂▂▂▂▂▂▂▂▂▂▂▂▂▂▂▂▂▃▃▃ |
