# Supplementary material for: The Past, Present, and Future of Virtual and Augmented Reality Research: A Network and Cluster Analysis of the Literature
Source: Front Psychol. 2018 Nov 6;9:2086. doi: 10.3389/fpsyg.2018.02086 (PMC6232426; doi:10.3389/fpsyg.2018.02086)
Supplement: Supplementary file 1 [file Data_Sheet_1.ZIP › Top 65 Subject Categories with Strongest Citation Bursts.docx]

**Top 65 Subject Categories with Strongest Citation Bursts**

| **Subject Categories** | **Year** | **Strength** | **Begin** | **End** | **1990 - 2016** |
| --- | --- | --- | --- | --- | --- |
| LITERATURE | 1990 | 9.6983 | **1990** | 1997 | ▃▃▃▃▃▃▃▃▂▂▂▂▂▂▂▂▂▂▂▂▂▂▂▂▂▂▂ |
| ARTS & HUMANITIES - OTHER TOPICS | 1990 | 9.5842 | **1990** | 1996 | ▃▃▃▃▃▃▃▂▂▂▂▂▂▂▂▂▂▂▂▂▂▂▂▂▂▂▂ |
| HUMANITIES | 1990 | 9.5842 | **1990** | 1996 | ▃▃▃▃▃▃▃▂▂▂▂▂▂▂▂▂▂▂▂▂▂▂▂▂▂▂▂ |
| LITERARY REVIEWS | 1990 | 8.4301 | **1990** | 2001 | ▃▃▃▃▃▃▃▃▃▃▃▃▂▂▂▂▂▂▂▂▂▂▂▂▂▂▂ |
| INFORMATION SCIENCE & LIBRARY SCIENCE | 1990 | 55.5258 | **1991** | 2000 | ▂▃▃▃▃▃▃▃▃▃▃▂▂▂▂▂▂▂▂▂▂▂▂▂▂▂▂ |
| ERGONOMICS | 1990 | 32.2312 | **1991** | 1997 | ▂▃▃▃▃▃▃▃▂▂▂▂▂▂▂▂▂▂▂▂▂▂▂▂▂▂▂ |
| BUSINESS & ECONOMICS | 1990 | 5.8594 | **1991** | 1996 | ▂▃▃▃▃▃▃▂▂▂▂▂▂▂▂▂▂▂▂▂▂▂▂▂▂▂▂ |
| GOVERNMENT & LAW | 1990 | 5.1499 | **1992** | 2004 | ▂▂▃▃▃▃▃▃▃▃▃▃▃▃▃▂▂▂▂▂▂▂▂▂▂▂▂ |
| PLANNING & DEVELOPMENT | 1990 | 3.5809 | **1993** | 1997 | ▂▂▂▃▃▃▃▃▂▂▂▂▂▂▂▂▂▂▂▂▂▂▂▂▂▂▂ |
| OPTICS | 1990 | 110.6344 | **1994** | 2002 | ▂▂▂▂▃▃▃▃▃▃▃▃▃▂▂▂▂▂▂▂▂▂▂▂▂▂▂ |
| AUTOMATION & CONTROL SYSTEMS | 1990 | 33.5687 | **1994** | 1999 | ▂▂▂▂▃▃▃▃▃▃▂▂▂▂▂▂▂▂▂▂▂▂▂▂▂▂▂ |
| ANATOMY & MORPHOLOGY | 1990 | 3.7407 | **1994** | 2000 | ▂▂▂▂▃▃▃▃▃▃▃▂▂▂▂▂▂▂▂▂▂▂▂▂▂▂▂ |
| MEDICAL LABORATORY TECHNOLOGY | 1990 | 10.6104 | **1994** | 1999 | ▂▂▂▂▃▃▃▃▃▃▂▂▂▂▂▂▂▂▂▂▂▂▂▂▂▂▂ |
| RADIOLOGY | 1990 | 37.1721 | **1995** | 2004 | ▂▂▂▂▂▃▃▃▃▃▃▃▃▃▃▂▂▂▂▂▂▂▂▂▂▂▂ |
| BIOLOGY | 1990 | 6.6494 | **1995** | 1997 | ▂▂▂▂▂▃▃▃▂▂▂▂▂▂▂▂▂▂▂▂▂▂▂▂▂▂▂ |
| LIFE SCIENCES & BIOMEDICINE - OTHER TOPICS | 1990 | 6.6494 | **1995** | 1997 | ▂▂▂▂▂▃▃▃▂▂▂▂▂▂▂▂▂▂▂▂▂▂▂▂▂▂▂ |
| GEOSCIENCES | 1990 | 4.3922 | **1997** | 1999 | ▂▂▂▂▂▂▂▃▃▃▂▂▂▂▂▂▂▂▂▂▂▂▂▂▂▂▂ |
| GEOLOGY | 1990 | 3.9208 | **1997** | 1999 | ▂▂▂▂▂▂▂▃▃▃▂▂▂▂▂▂▂▂▂▂▂▂▂▂▂▂▂ |
| CARDIAC & CARDIOVASCULAR SYSTEMS | 1990 | 10.978 | **1998** | 2001 | ▂▂▂▂▂▂▂▂▃▃▃▃▂▂▂▂▂▂▂▂▂▂▂▂▂▂▂ |
| MEDICAL INFORMATICS | 1990 | 13.069 | **1998** | 1999 | ▂▂▂▂▂▂▂▂▃▃▂▂▂▂▂▂▂▂▂▂▂▂▂▂▂▂▂ |
| TRANSPORTATION | 1990 | 4.7619 | **1998** | 1999 | ▂▂▂▂▂▂▂▂▃▃▂▂▂▂▂▂▂▂▂▂▂▂▂▂▂▂▂ |
| OCEANOGRAPHY | 1990 | 3.7155 | **1999** | 2005 | ▂▂▂▂▂▂▂▂▂▃▃▃▃▃▃▃▂▂▂▂▂▂▂▂▂▂▂ |
| TRANSPORTATION SCIENCE & TECHNOLOGY | 1990 | 3.5187 | **1999** | 2003 | ▂▂▂▂▂▂▂▂▂▃▃▃▃▃▂▂▂▂▂▂▂▂▂▂▂▂▂ |
| ART | 1990 | 4.2234 | **1999** | 2002 | ▂▂▂▂▂▂▂▂▂▃▃▃▃▂▂▂▂▂▂▂▂▂▂▂▂▂▂ |
| COMMUNICATION | 1990 | 75.7524 | **2000** | 2006 | ▂▂▂▂▂▂▂▂▂▂▃▃▃▃▃▃▃▂▂▂▂▂▂▂▂▂▂ |
| ROBOTICS | 1990 | 21.4819 | **2001** | 2006 | ▂▂▂▂▂▂▂▂▂▂▂▃▃▃▃▃▃▂▂▂▂▂▂▂▂▂▂ |
| URBAN STUDIES | 1990 | 7.744 | **2001** | 2007 | ▂▂▂▂▂▂▂▂▂▂▂▃▃▃▃▃▃▃▂▂▂▂▂▂▂▂▂ |
| ASTRONOMY & ASTROPHYSICS | 1990 | 4.0128 | **2001** | 2004 | ▂▂▂▂▂▂▂▂▂▂▂▃▃▃▃▂▂▂▂▂▂▂▂▂▂▂▂ |
| ECOLOGY | 1990 | 4.4885 | **2001** | 2007 | ▂▂▂▂▂▂▂▂▂▂▂▃▃▃▃▃▃▃▂▂▂▂▂▂▂▂▂ |
| REMOTE SENSING | 1990 | 5.0549 | **2002** | 2003 | ▂▂▂▂▂▂▂▂▂▂▂▂▃▃▂▂▂▂▂▂▂▂▂▂▂▂▂ |
| ENERGY & FUELS | 1990 | 3.6635 | **2002** | 2004 | ▂▂▂▂▂▂▂▂▂▂▂▂▃▃▃▂▂▂▂▂▂▂▂▂▂▂▂ |
| INSTRUMENTS & INSTRUMENTATION | 1990 | 16.2857 | **2002** | 2005 | ▂▂▂▂▂▂▂▂▂▂▂▂▃▃▃▃▂▂▂▂▂▂▂▂▂▂▂ |
| PHYSICS | 1990 | 3.6957 | **2002** | 2004 | ▂▂▂▂▂▂▂▂▂▂▂▂▃▃▃▂▂▂▂▂▂▂▂▂▂▂▂ |
| RESEARCH & EXPERIMENTAL MEDICINE | 1990 | 39.0248 | **2003** | 2005 | ▂▂▂▂▂▂▂▂▂▂▂▂▂▃▃▃▂▂▂▂▂▂▂▂▂▂▂ |
| MEDICINE | 1990 | 28.6093 | **2003** | 2005 | ▂▂▂▂▂▂▂▂▂▂▂▂▂▃▃▃▂▂▂▂▂▂▂▂▂▂▂ |
| TELECOMMUNICATIONS | 1990 | 13.3204 | **2004** | 2008 | ▂▂▂▂▂▂▂▂▂▂▂▂▂▂▃▃▃▃▃▂▂▂▂▂▂▂▂ |
| OPERATIONS RESEARCH & MANAGEMENT SCIENCE | 1990 | 4.4233 | **2004** | 2005 | ▂▂▂▂▂▂▂▂▂▂▂▂▂▂▃▃▂▂▂▂▂▂▂▂▂▂▂ |
| MATHEMATICS | 1990 | 3.9468 | **2004** | 2005 | ▂▂▂▂▂▂▂▂▂▂▂▂▂▂▃▃▂▂▂▂▂▂▂▂▂▂▂ |
| NANOSCIENCE & NANOTECHNOLOGY | 1990 | 6.9068 | **2006** | 2012 | ▂▂▂▂▂▂▂▂▂▂▂▂▂▂▂▂▃▃▃▃▃▃▃▂▂▂▂ |
| LINGUISTICS | 1990 | 3.6663 | **2006** | 2008 | ▂▂▂▂▂▂▂▂▂▂▂▂▂▂▂▂▃▃▃▂▂▂▂▂▂▂▂ |
| AGRICULTURE | 1990 | 3.4203 | **2007** | 2008 | ▂▂▂▂▂▂▂▂▂▂▂▂▂▂▂▂▂▃▃▂▂▂▂▂▂▂▂ |
| IMAGING SCIENCE & PHOTOGRAPHIC TECHNOLOGY | 1990 | 67.4172 | **2007** | 2008 | ▂▂▂▂▂▂▂▂▂▂▂▂▂▂▂▂▂▃▃▂▂▂▂▂▂▂▂ |
| SOCIAL ISSUES | 1990 | 6.4368 | **2007** | 2008 | ▂▂▂▂▂▂▂▂▂▂▂▂▂▂▂▂▂▃▃▂▂▂▂▂▂▂▂ |
| BUSINESS | 1990 | 7.3086 | **2008** | 2009 | ▂▂▂▂▂▂▂▂▂▂▂▂▂▂▂▂▂▂▃▃▂▂▂▂▂▂▂ |
| HEALTH CARE SCIENCES & SERVICES | 1990 | 19.4604 | **2009** | 2010 | ▂▂▂▂▂▂▂▂▂▂▂▂▂▂▂▂▂▂▂▃▃▂▂▂▂▂▂ |
| ARCHAEOLOGY | 1990 | 5.4638 | **2009** | 2011 | ▂▂▂▂▂▂▂▂▂▂▂▂▂▂▂▂▂▂▂▃▃▃▂▂▂▂▂ |
| CHEMISTRY | 1990 | 5.0323 | **2009** | 2011 | ▂▂▂▂▂▂▂▂▂▂▂▂▂▂▂▂▂▂▂▃▃▃▂▂▂▂▂ |
| OTORHINOLARYNGOLOGY | 1990 | 5.1911 | **2010** | 2012 | ▂▂▂▂▂▂▂▂▂▂▂▂▂▂▂▂▂▂▂▂▃▃▃▂▂▂▂ |
| ARCHITECTURE | 1990 | 3.6407 | **2010** | 2013 | ▂▂▂▂▂▂▂▂▂▂▂▂▂▂▂▂▂▂▂▂▃▃▃▃▂▂▂ |
| OBSTETRICS & GYNECOLOGY | 1990 | 7.1201 | **2011** | 2014 | ▂▂▂▂▂▂▂▂▂▂▂▂▂▂▂▂▂▂▂▂▂▃▃▃▃▂▂ |
| GENERAL & INTERNAL MEDICINE | 1990 | 4.6706 | **2011** | 2012 | ▂▂▂▂▂▂▂▂▂▂▂▂▂▂▂▂▂▂▂▂▂▃▃▂▂▂▂ |
| PEDIATRICS | 1990 | 7.7528 | **2011** | 2014 | ▂▂▂▂▂▂▂▂▂▂▂▂▂▂▂▂▂▂▂▂▂▃▃▃▃▂▂ |
| MATERIALS SCIENCE | 1990 | 48.7727 | **2011** | 2014 | ▂▂▂▂▂▂▂▂▂▂▂▂▂▂▂▂▂▂▂▂▂▃▃▃▃▂▂ |
| GERIATRICS & GERONTOLOGY | 1990 | 20.0883 | **2012** | 2016 | ▂▂▂▂▂▂▂▂▂▂▂▂▂▂▂▂▂▂▂▂▂▂▃▃▃▃▃ |
| SCIENCE & TECHNOLOGY - OTHER TOPICS | 1990 | 18.2456 | **2012** | 2016 | ▂▂▂▂▂▂▂▂▂▂▂▂▂▂▂▂▂▂▂▂▂▂▃▃▃▃▃ |
| NURSING | 1990 | 10.5211 | **2013** | 2016 | ▂▂▂▂▂▂▂▂▂▂▂▂▂▂▂▂▂▂▂▂▂▂▂▃▃▃▃ |
| SOCIAL SCIENCES - OTHER TOPICS | 1990 | 10.3434 | **2013** | 2016 | ▂▂▂▂▂▂▂▂▂▂▂▂▂▂▂▂▂▂▂▂▂▂▂▃▃▃▃ |
| NEUROSCIENCES & NEUROLOGY | 1990 | 91.2726 | **2013** | 2016 | ▂▂▂▂▂▂▂▂▂▂▂▂▂▂▂▂▂▂▂▂▂▂▂▃▃▃▃ |
| SOCIAL SCIENCES | 1990 | 12.6457 | **2013** | 2016 | ▂▂▂▂▂▂▂▂▂▂▂▂▂▂▂▂▂▂▂▂▂▂▂▃▃▃▃ |
| PSYCHIATRY | 1990 | 12.9246 | **2013** | 2016 | ▂▂▂▂▂▂▂▂▂▂▂▂▂▂▂▂▂▂▂▂▂▂▂▃▃▃▃ |
| MULTIDISCIPLINARY SCIENCES | 1990 | 19.7575 | **2013** | 2016 | ▂▂▂▂▂▂▂▂▂▂▂▂▂▂▂▂▂▂▂▂▂▂▂▃▃▃▃ |
| REHABILITATION | 1990 | 33.3353 | **2014** | 2016 | ▂▂▂▂▂▂▂▂▂▂▂▂▂▂▂▂▂▂▂▂▂▂▂▂▃▃▃ |
| ORTHOPEDICS | 1990 | 15.3496 | **2014** | 2016 | ▂▂▂▂▂▂▂▂▂▂▂▂▂▂▂▂▂▂▂▂▂▂▂▂▃▃▃ |
| BEHAVIORAL SCIENCES | 1990 | 12.7806 | **2014** | 2016 | ▂▂▂▂▂▂▂▂▂▂▂▂▂▂▂▂▂▂▂▂▂▂▂▂▃▃▃ |
| NEUROSCIENCES | 1990 | 77.707 | **2014** | 2016 | ▂▂▂▂▂▂▂▂▂▂▂▂▂▂▂▂▂▂▂▂▂▂▂▂▃▃▃ |
