# Supplementary material for: The Past, Present, and Future of Virtual and Augmented Reality Research: A Network and Cluster Analysis of the Literature
Source: Front Psychol. 2018 Nov 6;9:2086. doi: 10.3389/fpsyg.2018.02086 (PMC6232426; doi:10.3389/fpsyg.2018.02086)
Supplement: Supplementary file 1 [file Data_Sheet_1.ZIP › Top 124 Keywords with Strongest Citation Bursts.docx]

**Top 124 Keywords with Strongest Citation Bursts**

| **Keywords** | **Year** | **Strength** | **Begin** | **End** | **1990 - 2016** |
| --- | --- | --- | --- | --- | --- |
| telepresence | 1990 | 16.4912 | **1990** | 2001 | ▃▃▃▃▃▃▃▃▃▃▃▃▂▂▂▂▂▂▂▂▂▂▂▂▂▂▂ |
| computer | 1990 | 10.7067 | **1990** | 2004 | ▃▃▃▃▃▃▃▃▃▃▃▃▃▃▃▂▂▂▂▂▂▂▂▂▂▂▂ |
| human factor | 1990 | 3.968 | **1990** | 1996 | ▃▃▃▃▃▃▃▂▂▂▂▂▂▂▂▂▂▂▂▂▂▂▂▂▂▂▂ |
| cad | 1990 | 10.8249 | **1990** | 2004 | ▃▃▃▃▃▃▃▃▃▃▃▃▃▃▃▂▂▂▂▂▂▂▂▂▂▂▂ |
| stereoscopic display | 1990 | 7.4959 | **1992** | 2001 | ▂▂▃▃▃▃▃▃▃▃▃▃▂▂▂▂▂▂▂▂▂▂▂▂▂▂▂ |
| robotics | 1990 | 12.0921 | **1992** | 2003 | ▂▂▃▃▃▃▃▃▃▃▃▃▃▃▂▂▂▂▂▂▂▂▂▂▂▂▂ |
| multimedia | 1990 | 19.3298 | **1992** | 2002 | ▂▂▃▃▃▃▃▃▃▃▃▃▃▂▂▂▂▂▂▂▂▂▂▂▂▂▂ |
| cyberspace | 1990 | 7.0191 | **1992** | 1998 | ▂▂▃▃▃▃▃▃▃▂▂▂▂▂▂▂▂▂▂▂▂▂▂▂▂▂▂ |
| force display | 1990 | 3.6156 | **1992** | 2005 | ▂▂▃▃▃▃▃▃▃▃▃▃▃▃▃▃▂▂▂▂▂▂▂▂▂▂▂ |
| teleoperation | 1990 | 16.8969 | **1992** | 2002 | ▂▂▃▃▃▃▃▃▃▃▃▃▃▂▂▂▂▂▂▂▂▂▂▂▂▂▂ |
| scientific visualization | 1990 | 11.3753 | **1993** | 2003 | ▂▂▂▃▃▃▃▃▃▃▃▃▃▃▂▂▂▂▂▂▂▂▂▂▂▂▂ |
| head mounted display | 1990 | 8.7485 | **1993** | 2000 | ▂▂▂▃▃▃▃▃▃▃▃▂▂▂▂▂▂▂▂▂▂▂▂▂▂▂▂ |
| stereopsis | 1990 | 4.5859 | **1993** | 2002 | ▂▂▂▃▃▃▃▃▃▃▃▃▃▂▂▂▂▂▂▂▂▂▂▂▂▂▂ |
| cscw | 1990 | 9.1192 | **1993** | 2006 | ▂▂▂▃▃▃▃▃▃▃▃▃▃▃▃▃▃▂▂▂▂▂▂▂▂▂▂ |
| network | 1990 | 4.6593 | **1993** | 1996 | ▂▂▂▃▃▃▃▂▂▂▂▂▂▂▂▂▂▂▂▂▂▂▂▂▂▂▂ |
| human interface | 1990 | 5.8875 | **1994** | 2006 | ▂▂▂▂▃▃▃▃▃▃▃▃▃▃▃▃▃▂▂▂▂▂▂▂▂▂▂ |
| interactive | 1990 | 4.3042 | **1994** | 1998 | ▂▂▂▂▃▃▃▃▃▂▂▂▂▂▂▂▂▂▂▂▂▂▂▂▂▂▂ |
| neural network | 1990 | 3.5125 | **1994** | 2003 | ▂▂▂▂▃▃▃▃▃▃▃▃▃▃▂▂▂▂▂▂▂▂▂▂▂▂▂ |
| computer animation | 1990 | 6.2928 | **1994** | 2007 | ▂▂▂▂▃▃▃▃▃▃▃▃▃▃▃▃▃▃▂▂▂▂▂▂▂▂▂ |
| image processing | 1990 | 7.8923 | **1994** | 2000 | ▂▂▂▂▃▃▃▃▃▃▃▂▂▂▂▂▂▂▂▂▂▂▂▂▂▂▂ |
| geometric modeling | 1990 | 5.0423 | **1994** | 1998 | ▂▂▂▂▃▃▃▃▃▂▂▂▂▂▂▂▂▂▂▂▂▂▂▂▂▂▂ |
| animation | 1990 | 4.9171 | **1994** | 2001 | ▂▂▂▂▃▃▃▃▃▃▃▃▂▂▂▂▂▂▂▂▂▂▂▂▂▂▂ |
| hyper hospital | 1990 | 3.4059 | **1994** | 1995 | ▂▂▂▂▃▃▂▂▂▂▂▂▂▂▂▂▂▂▂▂▂▂▂▂▂▂▂ |
| user interface | 1990 | 5.9177 | **1994** | 2005 | ▂▂▂▂▃▃▃▃▃▃▃▃▃▃▃▃▂▂▂▂▂▂▂▂▂▂▂ |
| graphics | 1990 | 6.9762 | **1994** | 1998 | ▂▂▂▂▃▃▃▃▃▂▂▂▂▂▂▂▂▂▂▂▂▂▂▂▂▂▂ |
| image | 1990 | 15.5919 | **1994** | 2002 | ▂▂▂▂▃▃▃▃▃▃▃▃▃▂▂▂▂▂▂▂▂▂▂▂▂▂▂ |
| computer graphics | 1990 | 19.5424 | **1994** | 2003 | ▂▂▂▂▃▃▃▃▃▃▃▃▃▃▂▂▂▂▂▂▂▂▂▂▂▂▂ |
| telerobotics | 1990 | 12.6317 | **1994** | 2003 | ▂▂▂▂▃▃▃▃▃▃▃▃▃▃▂▂▂▂▂▂▂▂▂▂▂▂▂ |
| video | 1990 | 3.4737 | **1994** | 1997 | ▂▂▂▂▃▃▃▃▂▂▂▂▂▂▂▂▂▂▂▂▂▂▂▂▂▂▂ |
| atomic force microscope | 1990 | 3.3766 | **1994** | 2006 | ▂▂▂▂▃▃▃▃▃▃▃▃▃▃▃▃▃▂▂▂▂▂▂▂▂▂▂ |
| real time | 1990 | 8.1357 | **1994** | 2005 | ▂▂▂▂▃▃▃▃▃▃▃▃▃▃▃▃▂▂▂▂▂▂▂▂▂▂▂ |
| reconstruction | 1990 | 8.4631 | **1994** | 2002 | ▂▂▂▂▃▃▃▃▃▃▃▃▃▂▂▂▂▂▂▂▂▂▂▂▂▂▂ |
| artificial intelligence | 1990 | 5.0768 | **1995** | 2004 | ▂▂▂▂▂▃▃▃▃▃▃▃▃▃▃▂▂▂▂▂▂▂▂▂▂▂▂ |
| telemanipulation | 1990 | 4.2279 | **1995** | 1999 | ▂▂▂▂▂▃▃▃▃▃▂▂▂▂▂▂▂▂▂▂▂▂▂▂▂▂▂ |
| virtual reality | 1990 | 7.9795 | **1995** | 2002 | ▂▂▂▂▂▃▃▃▃▃▃▃▃▂▂▂▂▂▂▂▂▂▂▂▂▂▂ |
| computer vision | 1990 | 6.6649 | **1995** | 2000 | ▂▂▂▂▂▃▃▃▃▃▃▂▂▂▂▂▂▂▂▂▂▂▂▂▂▂▂ |
| helmet mounted display | 1990 | 3.7048 | **1995** | 1999 | ▂▂▂▂▂▃▃▃▃▃▂▂▂▂▂▂▂▂▂▂▂▂▂▂▂▂▂ |
| human machine interface | 1990 | 3.9093 | **1995** | 2006 | ▂▂▂▂▂▃▃▃▃▃▃▃▃▃▃▃▃▂▂▂▂▂▂▂▂▂▂ |
| distributed interactive simulation | 1990 | 4.8028 | **1995** | 2000 | ▂▂▂▂▂▃▃▃▃▃▃▂▂▂▂▂▂▂▂▂▂▂▂▂▂▂▂ |
| camera | 1990 | 3.3862 | **1995** | 1998 | ▂▂▂▂▂▃▃▃▃▂▂▂▂▂▂▂▂▂▂▂▂▂▂▂▂▂▂ |
| virtual environment | 1990 | 10.7175 | **1995** | 1999 | ▂▂▂▂▂▃▃▃▃▃▂▂▂▂▂▂▂▂▂▂▂▂▂▂▂▂▂ |
| gesture recognition | 1990 | 5.1266 | **1995** | 2002 | ▂▂▂▂▂▃▃▃▃▃▃▃▃▂▂▂▂▂▂▂▂▂▂▂▂▂▂ |
| computed tomography | 1990 | 6.4765 | **1995** | 2004 | ▂▂▂▂▂▃▃▃▃▃▃▃▃▃▃▂▂▂▂▂▂▂▂▂▂▂▂ |
| hmd | 1990 | 5.7868 | **1995** | 2005 | ▂▂▂▂▂▃▃▃▃▃▃▃▃▃▃▃▂▂▂▂▂▂▂▂▂▂▂ |
| medical imaging | 1990 | 3.7795 | **1995** | 2007 | ▂▂▂▂▂▃▃▃▃▃▃▃▃▃▃▃▃▃▂▂▂▂▂▂▂▂▂ |
| java | 1990 | 16.1351 | **1996** | 2003 | ▂▂▂▂▂▂▃▃▃▃▃▃▃▃▂▂▂▂▂▂▂▂▂▂▂▂▂ |
| world wide web | 1990 | 17.0918 | **1996** | 2001 | ▂▂▂▂▂▂▃▃▃▃▃▃▂▂▂▂▂▂▂▂▂▂▂▂▂▂▂ |
| library | 1990 | 5.7526 | **1996** | 2001 | ▂▂▂▂▂▂▃▃▃▃▃▃▂▂▂▂▂▂▂▂▂▂▂▂▂▂▂ |
| simulator sickness | 1990 | 3.6816 | **1996** | 1999 | ▂▂▂▂▂▂▃▃▃▃▂▂▂▂▂▂▂▂▂▂▂▂▂▂▂▂▂ |
| display | 1990 | 13.1599 | **1996** | 2000 | ▂▂▂▂▂▂▃▃▃▃▃▂▂▂▂▂▂▂▂▂▂▂▂▂▂▂▂ |
| endoscopy | 1990 | 9.5002 | **1996** | 2004 | ▂▂▂▂▂▂▃▃▃▃▃▃▃▃▃▂▂▂▂▂▂▂▂▂▂▂▂ |
| vrml | 1990 | 48.7397 | **1996** | 2004 | ▂▂▂▂▂▂▃▃▃▃▃▃▃▃▃▂▂▂▂▂▂▂▂▂▂▂▂ |
| www | 1990 | 9.9616 | **1996** | 2004 | ▂▂▂▂▂▂▃▃▃▃▃▃▃▃▃▂▂▂▂▂▂▂▂▂▂▂▂ |
| database | 1990 | 10.7316 | **1996** | 2003 | ▂▂▂▂▂▂▃▃▃▃▃▃▃▃▂▂▂▂▂▂▂▂▂▂▂▂▂ |
| accommodation | 1990 | 3.737 | **1996** | 2005 | ▂▂▂▂▂▂▃▃▃▃▃▃▃▃▃▃▂▂▂▂▂▂▂▂▂▂▂ |
| modeling | 1990 | 6.6013 | **1997** | 2005 | ▂▂▂▂▂▂▂▃▃▃▃▃▃▃▃▃▂▂▂▂▂▂▂▂▂▂▂ |
| surface | 1990 | 5.17 | **1997** | 2007 | ▂▂▂▂▂▂▂▃▃▃▃▃▃▃▃▃▃▃▂▂▂▂▂▂▂▂▂ |
| object | 1990 | 4.8986 | **1997** | 2004 | ▂▂▂▂▂▂▂▃▃▃▃▃▃▃▃▂▂▂▂▂▂▂▂▂▂▂▂ |
| map | 1990 | 4.3084 | **1997** | 2002 | ▂▂▂▂▂▂▂▃▃▃▃▃▃▂▂▂▂▂▂▂▂▂▂▂▂▂▂ |
| medicine | 1990 | 9.427 | **1997** | 2002 | ▂▂▂▂▂▂▂▃▃▃▃▃▃▂▂▂▂▂▂▂▂▂▂▂▂▂▂ |
| visualisation | 1990 | 9.2336 | **1997** | 2005 | ▂▂▂▂▂▂▂▃▃▃▃▃▃▃▃▃▂▂▂▂▂▂▂▂▂▂▂ |
| visualization | 1990 | 19.3587 | **1997** | 2001 | ▂▂▂▂▂▂▂▃▃▃▃▃▂▂▂▂▂▂▂▂▂▂▂▂▂▂▂ |
| assembly planning | 1990 | 3.7553 | **1997** | 2004 | ▂▂▂▂▂▂▂▃▃▃▃▃▃▃▃▂▂▂▂▂▂▂▂▂▂▂▂ |
| force feedback | 1990 | 6.6754 | **1997** | 2006 | ▂▂▂▂▂▂▂▃▃▃▃▃▃▃▃▃▃▂▂▂▂▂▂▂▂▂▂ |
| image based rendering | 1990 | 11.1233 | **1997** | 2005 | ▂▂▂▂▂▂▂▃▃▃▃▃▃▃▃▃▂▂▂▂▂▂▂▂▂▂▂ |
| virtual endoscopy | 1990 | 10.0731 | **1997** | 2002 | ▂▂▂▂▂▂▂▃▃▃▃▃▃▂▂▂▂▂▂▂▂▂▂▂▂▂▂ |
| segmentation | 1990 | 4.0756 | **1998** | 2001 | ▂▂▂▂▂▂▂▂▃▃▃▃▂▂▂▂▂▂▂▂▂▂▂▂▂▂▂ |
| internet | 1990 | 27.459 | **1998** | 2005 | ▂▂▂▂▂▂▂▂▃▃▃▃▃▃▃▃▂▂▂▂▂▂▂▂▂▂▂ |
| telemedicine | 1990 | 7.1133 | **1998** | 2001 | ▂▂▂▂▂▂▂▂▃▃▃▃▂▂▂▂▂▂▂▂▂▂▂▂▂▂▂ |
| collision detection | 1990 | 5.7228 | **1999** | 2005 | ▂▂▂▂▂▂▂▂▂▃▃▃▃▃▃▃▂▂▂▂▂▂▂▂▂▂▂ |
| virtual prototyping | 1990 | 5.3278 | **1999** | 2009 | ▂▂▂▂▂▂▂▂▂▃▃▃▃▃▃▃▃▃▃▃▂▂▂▂▂▂▂ |
| interface | 1990 | 3.9216 | **1999** | 2002 | ▂▂▂▂▂▂▂▂▂▃▃▃▃▂▂▂▂▂▂▂▂▂▂▂▂▂▂ |
| haptic interface | 1990 | 8.1128 | **1999** | 2007 | ▂▂▂▂▂▂▂▂▂▃▃▃▃▃▃▃▃▃▂▂▂▂▂▂▂▂▂ |
| mist vr | 1990 | 17.3674 | **2000** | 2007 | ▂▂▂▂▂▂▂▂▂▂▃▃▃▃▃▃▃▃▂▂▂▂▂▂▂▂▂ |
| web | 1990 | 5.3716 | **2000** | 2003 | ▂▂▂▂▂▂▂▂▂▂▃▃▃▃▂▂▂▂▂▂▂▂▂▂▂▂▂ |
| endoscopic surgery | 1990 | 6.932 | **2000** | 2005 | ▂▂▂▂▂▂▂▂▂▂▃▃▃▃▃▃▂▂▂▂▂▂▂▂▂▂▂ |
| motion | 1990 | 4.3653 | **2000** | 2002 | ▂▂▂▂▂▂▂▂▂▂▃▃▃▂▂▂▂▂▂▂▂▂▂▂▂▂▂ |
| fear | 1990 | 11.6602 | **2001** | 2003 | ▂▂▂▂▂▂▂▂▂▂▂▃▃▃▂▂▂▂▂▂▂▂▂▂▂▂▂ |
| cave | 1990 | 4.9215 | **2001** | 2006 | ▂▂▂▂▂▂▂▂▂▂▂▃▃▃▃▃▃▂▂▂▂▂▂▂▂▂▂ |
| acrophobia | 1990 | 8.0809 | **2001** | 2005 | ▂▂▂▂▂▂▂▂▂▂▂▃▃▃▃▃▂▂▂▂▂▂▂▂▂▂▂ |
| surgery simulation | 1990 | 6.3829 | **2001** | 2007 | ▂▂▂▂▂▂▂▂▂▂▂▃▃▃▃▃▃▃▂▂▂▂▂▂▂▂▂ |
| assembly | 1990 | 7.0337 | **2002** | 2005 | ▂▂▂▂▂▂▂▂▂▂▂▂▃▃▃▃▂▂▂▂▂▂▂▂▂▂▂ |
| future | 1990 | 3.4795 | **2002** | 2005 | ▂▂▂▂▂▂▂▂▂▂▂▂▃▃▃▃▂▂▂▂▂▂▂▂▂▂▂ |
| virtual reality (vr) | 1990 | 5.9787 | **2002** | 2007 | ▂▂▂▂▂▂▂▂▂▂▂▂▃▃▃▃▃▃▂▂▂▂▂▂▂▂▂ |
| follow up | 1990 | 4.491 | **2003** | 2006 | ▂▂▂▂▂▂▂▂▂▂▂▂▂▃▃▃▃▂▂▂▂▂▂▂▂▂▂ |
| psychomotor skill | 1990 | 11.7081 | **2004** | 2008 | ▂▂▂▂▂▂▂▂▂▂▂▂▂▂▃▃▃▃▃▂▂▂▂▂▂▂▂ |
| surgical training | 1990 | 5.8421 | **2004** | 2006 | ▂▂▂▂▂▂▂▂▂▂▂▂▂▂▃▃▃▂▂▂▂▂▂▂▂▂▂ |
| haptics | 1990 | 7.1016 | **2004** | 2009 | ▂▂▂▂▂▂▂▂▂▂▂▂▂▂▃▃▃▃▃▃▂▂▂▂▂▂▂ |
| adaptation | 1990 | 4.4946 | **2005** | 2006 | ▂▂▂▂▂▂▂▂▂▂▂▂▂▂▂▃▃▂▂▂▂▂▂▂▂▂▂ |
| laparoscopic skill | 1990 | 7.6384 | **2005** | 2008 | ▂▂▂▂▂▂▂▂▂▂▂▂▂▂▂▃▃▃▃▂▂▂▂▂▂▂▂ |
| acquisition | 1990 | 6.9764 | **2006** | 2008 | ▂▂▂▂▂▂▂▂▂▂▂▂▂▂▂▂▃▃▃▂▂▂▂▂▂▂▂ |
| field | 1990 | 5.3157 | **2007** | 2008 | ▂▂▂▂▂▂▂▂▂▂▂▂▂▂▂▂▂▃▃▂▂▂▂▂▂▂▂ |
| mixed reality | 1990 | 5.231 | **2009** | 2010 | ▂▂▂▂▂▂▂▂▂▂▂▂▂▂▂▂▂▂▂▃▃▂▂▂▂▂▂ |
| pain | 1990 | 4.322 | **2009** | 2010 | ▂▂▂▂▂▂▂▂▂▂▂▂▂▂▂▂▂▂▂▃▃▂▂▂▂▂▂ |
| posttraumatic stress disorder | 1990 | 5.2496 | **2010** | 2012 | ▂▂▂▂▂▂▂▂▂▂▂▂▂▂▂▂▂▂▂▂▃▃▃▂▂▂▂ |
| curriculum | 1990 | 4.6356 | **2011** | 2012 | ▂▂▂▂▂▂▂▂▂▂▂▂▂▂▂▂▂▂▂▂▂▃▃▂▂▂▂ |
| virtual reality simulation | 1990 | 5.8972 | **2011** | 2012 | ▂▂▂▂▂▂▂▂▂▂▂▂▂▂▂▂▂▂▂▂▂▃▃▂▂▂▂ |
| construct validity | 1990 | 4.3141 | **2011** | 2014 | ▂▂▂▂▂▂▂▂▂▂▂▂▂▂▂▂▂▂▂▂▂▃▃▃▃▂▂ |
| eye tracking | 1990 | 3.7087 | **2011** | 2016 | ▂▂▂▂▂▂▂▂▂▂▂▂▂▂▂▂▂▂▂▂▂▃▃▃▃▃▃ |
| exercise | 1990 | 10.5367 | **2012** | 2016 | ▂▂▂▂▂▂▂▂▂▂▂▂▂▂▂▂▂▂▂▂▂▂▃▃▃▃▃ |
| video game | 1990 | 15.8515 | **2012** | 2016 | ▂▂▂▂▂▂▂▂▂▂▂▂▂▂▂▂▂▂▂▂▂▂▃▃▃▃▃ |
| executive function | 1990 | 5.9438 | **2012** | 2016 | ▂▂▂▂▂▂▂▂▂▂▂▂▂▂▂▂▂▂▂▂▂▂▃▃▃▃▃ |
| metaanalysis | 1990 | 15.1084 | **2012** | 2016 | ▂▂▂▂▂▂▂▂▂▂▂▂▂▂▂▂▂▂▂▂▂▂▃▃▃▃▃ |
| mild cognitive impairment | 1990 | 10.6212 | **2012** | 2016 | ▂▂▂▂▂▂▂▂▂▂▂▂▂▂▂▂▂▂▂▂▂▂▃▃▃▃▃ |
| reliability | 1990 | 7.646 | **2013** | 2014 | ▂▂▂▂▂▂▂▂▂▂▂▂▂▂▂▂▂▂▂▂▂▂▂▃▃▂▂ |
| behavior | 1990 | 7.3769 | **2013** | 2016 | ▂▂▂▂▂▂▂▂▂▂▂▂▂▂▂▂▂▂▂▂▂▂▂▃▃▃▃ |
| randomized controlled trial | 1990 | 45.7441 | **2013** | 2016 | ▂▂▂▂▂▂▂▂▂▂▂▂▂▂▂▂▂▂▂▂▂▂▂▃▃▃▃ |
| disorder | 1990 | 5.3415 | **2013** | 2016 | ▂▂▂▂▂▂▂▂▂▂▂▂▂▂▂▂▂▂▂▂▂▂▂▃▃▃▃ |
| decision making | 1990 | 3.7958 | **2013** | 2014 | ▂▂▂▂▂▂▂▂▂▂▂▂▂▂▂▂▂▂▂▂▂▂▂▃▃▂▂ |
| walking | 1990 | 7.4654 | **2013** | 2016 | ▂▂▂▂▂▂▂▂▂▂▂▂▂▂▂▂▂▂▂▂▂▂▂▃▃▃▃ |
| cerebral palsy | 1990 | 9.7121 | **2013** | 2014 | ▂▂▂▂▂▂▂▂▂▂▂▂▂▂▂▂▂▂▂▂▂▂▂▃▃▂▂ |
| balance | 1990 | 23.7436 | **2014** | 2016 | ▂▂▂▂▂▂▂▂▂▂▂▂▂▂▂▂▂▂▂▂▂▂▂▂▃▃▃ |
| validation | 1990 | 7.8825 | **2014** | 2016 | ▂▂▂▂▂▂▂▂▂▂▂▂▂▂▂▂▂▂▂▂▂▂▂▂▃▃▃ |
| alzheimers disease | 1990 | 8.9867 | **2014** | 2016 | ▂▂▂▂▂▂▂▂▂▂▂▂▂▂▂▂▂▂▂▂▂▂▂▂▃▃▃ |
| people | 1990 | 12.27 | **2014** | 2016 | ▂▂▂▂▂▂▂▂▂▂▂▂▂▂▂▂▂▂▂▂▂▂▂▂▃▃▃ |
| schizophrenia | 1990 | 10.3957 | **2014** | 2016 | ▂▂▂▂▂▂▂▂▂▂▂▂▂▂▂▂▂▂▂▂▂▂▂▂▃▃▃ |
| trial | 1990 | 4.2278 | **2014** | 2016 | ▂▂▂▂▂▂▂▂▂▂▂▂▂▂▂▂▂▂▂▂▂▂▂▂▃▃▃ |
| anxiety | 1990 | 5.6166 | **2014** | 2016 | ▂▂▂▂▂▂▂▂▂▂▂▂▂▂▂▂▂▂▂▂▂▂▂▂▃▃▃ |
| intervention | 1990 | 12.2486 | **2014** | 2016 | ▂▂▂▂▂▂▂▂▂▂▂▂▂▂▂▂▂▂▂▂▂▂▂▂▃▃▃ |
| upper limb | 1990 | 3.9288 | **2014** | 2016 | ▂▂▂▂▂▂▂▂▂▂▂▂▂▂▂▂▂▂▂▂▂▂▂▂▃▃▃ |
| stroke rehabilitation | 1990 | 6.724 | **2014** | 2016 | ▂▂▂▂▂▂▂▂▂▂▂▂▂▂▂▂▂▂▂▂▂▂▂▂▃▃▃ |
| stroke | 1990 | 3.7338 | **2014** | 2016 | ▂▂▂▂▂▂▂▂▂▂▂▂▂▂▂▂▂▂▂▂▂▂▂▂▃▃▃ |
| physical activity | 1990 | 12.4185 | **2014** | 2016 | ▂▂▂▂▂▂▂▂▂▂▂▂▂▂▂▂▂▂▂▂▂▂▂▂▃▃▃ |
| older adult | 1990 | 26.3984 | **2014** | 2016 | ▂▂▂▂▂▂▂▂▂▂▂▂▂▂▂▂▂▂▂▂▂▂▂▂▃▃▃ |
