# Supplementary material for: The Past, Present, and Future of Virtual and Augmented Reality Research: A Network and Cluster Analysis of the Literature
Source: Front Psychol. 2018 Nov 6;9:2086. doi: 10.3389/fpsyg.2018.02086 (PMC6232426; doi:10.3389/fpsyg.2018.02086)
Supplement: Supplementary file 1 [file Data_Sheet_1.ZIP › Top 155 Institutions with Strongest Citation Bursts.docx]

**Top 155 Institutions with Strongest Citation Bursts**

| **Institutions** | **Year** | **Strength** | **Begin** | **End** | **1990 - 2016** |
| --- | --- | --- | --- | --- | --- |
| UNIV N CAROLINA | 1990 | 3.3339 | **1990** | 1996 | ▃▃▃▃▃▃▃▂▂▂▂▂▂▂▂▂▂▂▂▂▂▂▂▂▂▂▂ |
| NASA | 1990 | 9.2231 | **1990** | 2003 | ▃▃▃▃▃▃▃▃▃▃▃▃▃▃▂▂▂▂▂▂▂▂▂▂▂▂▂ |
| UNIV WASHINGTON | 1990 | 4.5887 | **1993** | 1997 | ▂▂▂▃▃▃▃▃▂▂▂▂▂▂▂▂▂▂▂▂▂▂▂▂▂▂▂ |
| GMD | 1990 | 3.743 | **1994** | 2001 | ▂▂▂▂▃▃▃▃▃▃▃▃▂▂▂▂▂▂▂▂▂▂▂▂▂▂▂ |
| UNIV CALIF BERKELEY | 1990 | 3.9513 | **1994** | 1997 | ▂▂▂▂▃▃▃▃▂▂▂▂▂▂▂▂▂▂▂▂▂▂▂▂▂▂▂ |
| UNIV NOTTINGHAM | 1990 | 4.6104 | **1994** | 1997 | ▂▂▂▂▃▃▃▃▂▂▂▂▂▂▂▂▂▂▂▂▂▂▂▂▂▂▂ |
| UNIV ILLINOIS | 1990 | 7.2486 | **1994** | 1997 | ▂▂▂▂▃▃▃▃▂▂▂▂▂▂▂▂▂▂▂▂▂▂▂▂▂▂▂ |
| ARGONNE NATL LAB | 1990 | 3.3516 | **1994** | 1996 | ▂▂▂▂▃▃▃▂▂▂▂▂▂▂▂▂▂▂▂▂▂▂▂▂▂▂▂ |
| SWISS FED INST TECHNOL | 1990 | 4.0224 | **1994** | 1996 | ▂▂▂▂▃▃▃▂▂▂▂▂▂▂▂▂▂▂▂▂▂▂▂▂▂▂▂ |
| GEORGIA INST TECHNOL | 1990 | 5.3207 | **1995** | 1997 | ▂▂▂▂▂▃▃▃▂▂▂▂▂▂▂▂▂▂▂▂▂▂▂▂▂▂▂ |
| TOKAI UNIV | 1990 | 3.3834 | **1995** | 1996 | ▂▂▂▂▂▃▃▂▂▂▂▂▂▂▂▂▂▂▂▂▂▂▂▂▂▂▂ |
| USN | 1990 | 8.2309 | **1995** | 1999 | ▂▂▂▂▂▃▃▃▃▃▂▂▂▂▂▂▂▂▂▂▂▂▂▂▂▂▂ |
| UNIV HOUSTON | 1990 | 3.9895 | **1995** | 1997 | ▂▂▂▂▂▃▃▃▂▂▂▂▂▂▂▂▂▂▂▂▂▂▂▂▂▂▂ |
| CALTECH | 1990 | 4.0747 | **1996** | 2001 | ▂▂▂▂▂▂▃▃▃▃▃▃▂▂▂▂▂▂▂▂▂▂▂▂▂▂▂ |
| Hong Kong Univ Sci & Technol | 1990 | 10.6576 | **1997** | 2002 | ▂▂▂▂▂▂▂▃▃▃▃▃▃▂▂▂▂▂▂▂▂▂▂▂▂▂▂ |
| Nottingham Trent Univ | 1990 | 4.1988 | **1997** | 2002 | ▂▂▂▂▂▂▂▃▃▃▃▃▃▂▂▂▂▂▂▂▂▂▂▂▂▂▂ |
| Kyoto Univ | 1990 | 6.4353 | **1997** | 2007 | ▂▂▂▂▂▂▂▃▃▃▃▃▃▃▃▃▃▃▂▂▂▂▂▂▂▂▂ |
| Univ Surrey | 1990 | 3.4293 | **1997** | 1999 | ▂▂▂▂▂▂▂▃▃▃▂▂▂▂▂▂▂▂▂▂▂▂▂▂▂▂▂ |
| Univ Oulu | 1990 | 3.8319 | **1997** | 2002 | ▂▂▂▂▂▂▂▃▃▃▃▃▃▂▂▂▂▂▂▂▂▂▂▂▂▂▂ |
| Chinese Univ Hong Kong | 1990 | 8.1379 | **1997** | 2007 | ▂▂▂▂▂▂▂▃▃▃▃▃▃▃▃▃▃▃▂▂▂▂▂▂▂▂▂ |
| Mayo Clin & Mayo Fdn | 1990 | 8.3683 | **1997** | 2002 | ▂▂▂▂▂▂▂▃▃▃▃▃▃▂▂▂▂▂▂▂▂▂▂▂▂▂▂ |
| Nara Inst Sci & Technol | 1990 | 4.7138 | **1997** | 2002 | ▂▂▂▂▂▂▂▃▃▃▃▃▃▂▂▂▂▂▂▂▂▂▂▂▂▂▂ |
| Univ Salford | 1990 | 4.1165 | **1997** | 2003 | ▂▂▂▂▂▂▂▃▃▃▃▃▃▃▂▂▂▂▂▂▂▂▂▂▂▂▂ |
| ETH Zurich | 1990 | 3.906 | **1997** | 2001 | ▂▂▂▂▂▂▂▃▃▃▃▃▂▂▂▂▂▂▂▂▂▂▂▂▂▂▂ |
| Tamkang Univ | 1990 | 5.4402 | **1997** | 2004 | ▂▂▂▂▂▂▂▃▃▃▃▃▃▃▃▂▂▂▂▂▂▂▂▂▂▂▂ |
| Natl Tech Univ Athens | 1990 | 6.6413 | **1997** | 2001 | ▂▂▂▂▂▂▂▃▃▃▃▃▂▂▂▂▂▂▂▂▂▂▂▂▂▂▂ |
| Tsing Hua Univ | 1990 | 9.5663 | **1997** | 2005 | ▂▂▂▂▂▂▂▃▃▃▃▃▃▃▃▃▂▂▂▂▂▂▂▂▂▂▂ |
| Univ Strathclyde | 1990 | 5.686 | **1998** | 2003 | ▂▂▂▂▂▂▂▂▃▃▃▃▃▃▂▂▂▂▂▂▂▂▂▂▂▂▂ |
| Univ Washington | 1990 | 10.307 | **1998** | 2004 | ▂▂▂▂▂▂▂▂▃▃▃▃▃▃▃▂▂▂▂▂▂▂▂▂▂▂▂ |
| Fraunhofer Inst Comp Graph | 1990 | 7.8804 | **1998** | 2003 | ▂▂▂▂▂▂▂▂▃▃▃▃▃▃▂▂▂▂▂▂▂▂▂▂▂▂▂ |
| Chalmers Univ Technol | 1990 | 5.1557 | **1998** | 2004 | ▂▂▂▂▂▂▂▂▃▃▃▃▃▃▃▂▂▂▂▂▂▂▂▂▂▂▂ |
| Univ Erlangen Nurnberg | 1990 | 4.2661 | **1998** | 2001 | ▂▂▂▂▂▂▂▂▃▃▃▃▂▂▂▂▂▂▂▂▂▂▂▂▂▂▂ |
| Univ Illinois | 1990 | 14.5601 | **1998** | 2001 | ▂▂▂▂▂▂▂▂▃▃▃▃▂▂▂▂▂▂▂▂▂▂▂▂▂▂▂ |
| Jikei Univ | 1990 | 3.3669 | **1998** | 2005 | ▂▂▂▂▂▂▂▂▃▃▃▃▃▃▃▃▂▂▂▂▂▂▂▂▂▂▂ |
| Natl Univ Singapore | 1990 | 4.1842 | **1998** | 2002 | ▂▂▂▂▂▂▂▂▃▃▃▃▃▂▂▂▂▂▂▂▂▂▂▂▂▂▂ |
| George Mason Univ | 1990 | 3.5264 | **1998** | 2002 | ▂▂▂▂▂▂▂▂▃▃▃▃▃▂▂▂▂▂▂▂▂▂▂▂▂▂▂ |
| St Petersburg State Tech Univ | 1990 | 4.8041 | **1998** | 2002 | ▂▂▂▂▂▂▂▂▃▃▃▃▃▂▂▂▂▂▂▂▂▂▂▂▂▂▂ |
| Univ Dortmund | 1990 | 11.2324 | **1998** | 2004 | ▂▂▂▂▂▂▂▂▃▃▃▃▃▃▃▂▂▂▂▂▂▂▂▂▂▂▂ |
| Univ Saarland | 1990 | 3.8935 | **1998** | 2000 | ▂▂▂▂▂▂▂▂▃▃▃▂▂▂▂▂▂▂▂▂▂▂▂▂▂▂▂ |
| Nagoya Univ | 1990 | 3.8023 | **1998** | 2002 | ▂▂▂▂▂▂▂▂▃▃▃▃▃▂▂▂▂▂▂▂▂▂▂▂▂▂▂ |
| Ruhr Univ Bochum | 1990 | 4.8384 | **1998** | 2004 | ▂▂▂▂▂▂▂▂▃▃▃▃▃▃▃▂▂▂▂▂▂▂▂▂▂▂▂ |
| Univ Manchester | 1990 | 3.3494 | **1998** | 2007 | ▂▂▂▂▂▂▂▂▃▃▃▃▃▃▃▃▃▃▂▂▂▂▂▂▂▂▂ |
| GSF | 1990 | 4.048 | **1998** | 2004 | ▂▂▂▂▂▂▂▂▃▃▃▃▃▃▃▂▂▂▂▂▂▂▂▂▂▂▂ |
| City Univ Hong Kong | 1990 | 7.6067 | **1998** | 2001 | ▂▂▂▂▂▂▂▂▃▃▃▃▂▂▂▂▂▂▂▂▂▂▂▂▂▂▂ |
| Coll France | 1990 | 3.6926 | **1998** | 2004 | ▂▂▂▂▂▂▂▂▃▃▃▃▃▃▃▂▂▂▂▂▂▂▂▂▂▂▂ |
| Georgia Inst Technol | 1990 | 4.861 | **1999** | 2002 | ▂▂▂▂▂▂▂▂▂▃▃▃▃▂▂▂▂▂▂▂▂▂▂▂▂▂▂ |
| Univ Hamburg | 1990 | 4.1967 | **1999** | 2000 | ▂▂▂▂▂▂▂▂▂▃▃▂▂▂▂▂▂▂▂▂▂▂▂▂▂▂▂ |
| Swiss Fed Inst Technol | 1990 | 7.5451 | **1999** | 2005 | ▂▂▂▂▂▂▂▂▂▃▃▃▃▃▃▃▂▂▂▂▂▂▂▂▂▂▂ |
| Univ Hong Kong | 1990 | 5.9687 | **1999** | 2004 | ▂▂▂▂▂▂▂▂▂▃▃▃▃▃▃▂▂▂▂▂▂▂▂▂▂▂▂ |
| Beijing Univ Aeronaut & Astronaut | 1990 | 7.4826 | **1999** | 2003 | ▂▂▂▂▂▂▂▂▂▃▃▃▃▃▂▂▂▂▂▂▂▂▂▂▂▂▂ |
| Univ Texas | 1990 | 12.2154 | **1999** | 2007 | ▂▂▂▂▂▂▂▂▂▃▃▃▃▃▃▃▃▃▂▂▂▂▂▂▂▂▂ |
| Univ Iowa | 1990 | 6.8703 | **1999** | 2001 | ▂▂▂▂▂▂▂▂▂▃▃▃▂▂▂▂▂▂▂▂▂▂▂▂▂▂▂ |
| Stanford Univ | 1990 | 9.8975 | **2000** | 2005 | ▂▂▂▂▂▂▂▂▂▂▃▃▃▃▃▃▂▂▂▂▂▂▂▂▂▂▂ |
| Multimedia Univ | 1990 | 3.351 | **2000** | 2003 | ▂▂▂▂▂▂▂▂▂▂▃▃▃▃▂▂▂▂▂▂▂▂▂▂▂▂▂ |
| No Jiaotong Univ | 1990 | 4.7096 | **2000** | 2002 | ▂▂▂▂▂▂▂▂▂▂▃▃▃▂▂▂▂▂▂▂▂▂▂▂▂▂▂ |
| Natl Inst Fus Sci | 1990 | 3.481 | **2000** | 2007 | ▂▂▂▂▂▂▂▂▂▂▃▃▃▃▃▃▃▃▂▂▂▂▂▂▂▂▂ |
| Brown Univ | 1990 | 4.0016 | **2000** | 2003 | ▂▂▂▂▂▂▂▂▂▂▃▃▃▃▂▂▂▂▂▂▂▂▂▂▂▂▂ |
| Natl Res Council Canada | 1990 | 5.7817 | **2001** | 2007 | ▂▂▂▂▂▂▂▂▂▂▂▃▃▃▃▃▃▃▂▂▂▂▂▂▂▂▂ |
| Univ Dundee | 1990 | 3.729 | **2001** | 2003 | ▂▂▂▂▂▂▂▂▂▂▂▃▃▃▂▂▂▂▂▂▂▂▂▂▂▂▂ |
| Tokyo Inst Technol | 1990 | 4.7933 | **2001** | 2004 | ▂▂▂▂▂▂▂▂▂▂▂▃▃▃▃▂▂▂▂▂▂▂▂▂▂▂▂ |
| Griffith Univ | 1990 | 3.8548 | **2001** | 2003 | ▂▂▂▂▂▂▂▂▂▂▂▃▃▃▂▂▂▂▂▂▂▂▂▂▂▂▂ |
| Hanyang Univ | 1990 | 8.3224 | **2001** | 2008 | ▂▂▂▂▂▂▂▂▂▂▂▃▃▃▃▃▃▃▃▂▂▂▂▂▂▂▂ |
| Old Dominion Univ | 1990 | 3.4171 | **2001** | 2004 | ▂▂▂▂▂▂▂▂▂▂▂▃▃▃▃▂▂▂▂▂▂▂▂▂▂▂▂ |
| St Marys Hosp | 1990 | 9.1432 | **2001** | 2008 | ▂▂▂▂▂▂▂▂▂▂▂▃▃▃▃▃▃▃▃▂▂▂▂▂▂▂▂ |
| ETRI | 1990 | 3.695 | **2002** | 2006 | ▂▂▂▂▂▂▂▂▂▂▂▂▃▃▃▃▃▂▂▂▂▂▂▂▂▂▂ |
| Kyushu Inst Technol | 1990 | 7.2479 | **2002** | 2006 | ▂▂▂▂▂▂▂▂▂▂▂▂▃▃▃▃▃▂▂▂▂▂▂▂▂▂▂ |
| Nanyang Technol Univ | 1990 | 3.3886 | **2002** | 2006 | ▂▂▂▂▂▂▂▂▂▂▂▂▃▃▃▃▃▂▂▂▂▂▂▂▂▂▂ |
| Univ Zagreb | 1990 | 4.2771 | **2002** | 2004 | ▂▂▂▂▂▂▂▂▂▂▂▂▃▃▃▂▂▂▂▂▂▂▂▂▂▂▂ |
| Korea Inst Sci & Technol | 1990 | 3.9521 | **2002** | 2006 | ▂▂▂▂▂▂▂▂▂▂▂▂▃▃▃▃▃▂▂▂▂▂▂▂▂▂▂ |
| Univ Nottingham | 1990 | 4.2793 | **2002** | 2006 | ▂▂▂▂▂▂▂▂▂▂▂▂▃▃▃▃▃▂▂▂▂▂▂▂▂▂▂ |
| Catharina Hosp Eindhoven | 1990 | 5.2254 | **2002** | 2006 | ▂▂▂▂▂▂▂▂▂▂▂▂▃▃▃▃▃▂▂▂▂▂▂▂▂▂▂ |
| Tianjin Univ | 1990 | 4.3207 | **2003** | 2004 | ▂▂▂▂▂▂▂▂▂▂▂▂▂▃▃▂▂▂▂▂▂▂▂▂▂▂▂ |
| Zhejiang Univ | 1990 | 8.209 | **2003** | 2006 | ▂▂▂▂▂▂▂▂▂▂▂▂▂▃▃▃▃▂▂▂▂▂▂▂▂▂▂ |
| Sungkyunkwan Univ | 1990 | 5.3375 | **2003** | 2006 | ▂▂▂▂▂▂▂▂▂▂▂▂▂▃▃▃▃▂▂▂▂▂▂▂▂▂▂ |
| Univ Teesside | 1990 | 5.3298 | **2003** | 2005 | ▂▂▂▂▂▂▂▂▂▂▂▂▂▃▃▃▂▂▂▂▂▂▂▂▂▂▂ |
| Emory Univ | 1990 | 9.0722 | **2004** | 2006 | ▂▂▂▂▂▂▂▂▂▂▂▂▂▂▃▃▃▂▂▂▂▂▂▂▂▂▂ |
| Univ Malaga | 1990 | 4.0127 | **2004** | 2009 | ▂▂▂▂▂▂▂▂▂▂▂▂▂▂▃▃▃▃▃▃▂▂▂▂▂▂▂ |
| Rehabil Inst Chicago | 1990 | 3.8526 | **2004** | 2007 | ▂▂▂▂▂▂▂▂▂▂▂▂▂▂▃▃▃▃▂▂▂▂▂▂▂▂▂ |
| Univ New Mexico | 1990 | 4.2951 | **2004** | 2008 | ▂▂▂▂▂▂▂▂▂▂▂▂▂▂▃▃▃▃▃▂▂▂▂▂▂▂▂ |
| Univ So Calif | 1990 | 3.3613 | **2004** | 2005 | ▂▂▂▂▂▂▂▂▂▂▂▂▂▂▃▃▂▂▂▂▂▂▂▂▂▂▂ |
| Univ Ottawa | 1990 | 7.6891 | **2004** | 2008 | ▂▂▂▂▂▂▂▂▂▂▂▂▂▂▃▃▃▃▃▂▂▂▂▂▂▂▂ |
| Univ Georgia | 1990 | 7.2379 | **2004** | 2005 | ▂▂▂▂▂▂▂▂▂▂▂▂▂▂▃▃▂▂▂▂▂▂▂▂▂▂▂ |
| Penn State Univ | 1990 | 3.4906 | **2005** | 2007 | ▂▂▂▂▂▂▂▂▂▂▂▂▂▂▂▃▃▃▂▂▂▂▂▂▂▂▂ |
| Hebrew Univ Jerusalem | 1990 | 3.5805 | **2005** | 2006 | ▂▂▂▂▂▂▂▂▂▂▂▂▂▂▂▃▃▂▂▂▂▂▂▂▂▂▂ |
| Osaka Univ | 1990 | 4.0305 | **2005** | 2006 | ▂▂▂▂▂▂▂▂▂▂▂▂▂▂▂▃▃▂▂▂▂▂▂▂▂▂▂ |
| Monash Univ | 1990 | 5.9496 | **2005** | 2007 | ▂▂▂▂▂▂▂▂▂▂▂▂▂▂▂▃▃▃▂▂▂▂▂▂▂▂▂ |
| Wuhan Univ | 1990 | 9.5431 | **2005** | 2009 | ▂▂▂▂▂▂▂▂▂▂▂▂▂▂▂▃▃▃▃▃▂▂▂▂▂▂▂ |
| Univ Med & Dent New Jersey | 1990 | 4.3099 | **2006** | 2009 | ▂▂▂▂▂▂▂▂▂▂▂▂▂▂▂▂▃▃▃▃▂▂▂▂▂▂▂ |
| Univ Haifa | 1990 | 4.686 | **2006** | 2009 | ▂▂▂▂▂▂▂▂▂▂▂▂▂▂▂▂▃▃▃▃▂▂▂▂▂▂▂ |
| Univ Patras | 1990 | 4.7991 | **2006** | 2007 | ▂▂▂▂▂▂▂▂▂▂▂▂▂▂▂▂▃▃▂▂▂▂▂▂▂▂▂ |
| China Jiliang Univ | 1990 | 3.6424 | **2006** | 2009 | ▂▂▂▂▂▂▂▂▂▂▂▂▂▂▂▂▃▃▃▃▂▂▂▂▂▂▂ |
| Harbin Engn Univ | 1990 | 5.8346 | **2006** | 2010 | ▂▂▂▂▂▂▂▂▂▂▂▂▂▂▂▂▃▃▃▃▃▂▂▂▂▂▂ |
| Univ Politecn Madrid | 1990 | 3.691 | **2006** | 2010 | ▂▂▂▂▂▂▂▂▂▂▂▂▂▂▂▂▃▃▃▃▃▂▂▂▂▂▂ |
| Univ Munster | 1990 | 3.4117 | **2006** | 2011 | ▂▂▂▂▂▂▂▂▂▂▂▂▂▂▂▂▃▃▃▃▃▃▂▂▂▂▂ |
| Univ Ulster | 1990 | 3.7663 | **2006** | 2007 | ▂▂▂▂▂▂▂▂▂▂▂▂▂▂▂▂▃▃▂▂▂▂▂▂▂▂▂ |
| Vanderbilt Univ | 1990 | 4.1789 | **2007** | 2011 | ▂▂▂▂▂▂▂▂▂▂▂▂▂▂▂▂▂▃▃▃▃▃▂▂▂▂▂ |
| Catharina Hosp | 1990 | 8.784 | **2007** | 2010 | ▂▂▂▂▂▂▂▂▂▂▂▂▂▂▂▂▂▃▃▃▃▂▂▂▂▂▂ |
| Johannes Gutenberg Univ Mainz | 1990 | 4.5881 | **2007** | 2014 | ▂▂▂▂▂▂▂▂▂▂▂▂▂▂▂▂▂▃▃▃▃▃▃▃▃▂▂ |
| Univ Western Ontario | 1990 | 4.0376 | **2008** | 2010 | ▂▂▂▂▂▂▂▂▂▂▂▂▂▂▂▂▂▂▃▃▃▂▂▂▂▂▂ |
| Beihang Univ | 1990 | 4.9026 | **2008** | 2009 | ▂▂▂▂▂▂▂▂▂▂▂▂▂▂▂▂▂▂▃▃▂▂▂▂▂▂▂ |
| Erasmus MC | 1990 | 3.55 | **2008** | 2014 | ▂▂▂▂▂▂▂▂▂▂▂▂▂▂▂▂▂▂▃▃▃▃▃▃▃▂▂ |
| Northeastern Univ | 1990 | 7.5287 | **2008** | 2011 | ▂▂▂▂▂▂▂▂▂▂▂▂▂▂▂▂▂▂▃▃▃▃▂▂▂▂▂ |
| Tech Univ Cluj Napoca | 1990 | 6.4532 | **2008** | 2010 | ▂▂▂▂▂▂▂▂▂▂▂▂▂▂▂▂▂▂▃▃▃▂▂▂▂▂▂ |
| Harbin Inst Technol | 1990 | 4.598 | **2008** | 2010 | ▂▂▂▂▂▂▂▂▂▂▂▂▂▂▂▂▂▂▃▃▃▂▂▂▂▂▂ |
| Delft Univ Technol | 1990 | 4.845 | **2008** | 2010 | ▂▂▂▂▂▂▂▂▂▂▂▂▂▂▂▂▂▂▃▃▃▂▂▂▂▂▂ |
| Natl Taiwan Univ | 1990 | 3.4299 | **2008** | 2009 | ▂▂▂▂▂▂▂▂▂▂▂▂▂▂▂▂▂▂▃▃▂▂▂▂▂▂▂ |
| Macquarie Univ | 1990 | 3.3476 | **2008** | 2011 | ▂▂▂▂▂▂▂▂▂▂▂▂▂▂▂▂▂▂▃▃▃▃▂▂▂▂▂ |
| Univ Tecn Lisbon | 1990 | 4.4681 | **2009** | 2010 | ▂▂▂▂▂▂▂▂▂▂▂▂▂▂▂▂▂▂▂▃▃▂▂▂▂▂▂ |
| Graz Univ Technol | 1990 | 4.7343 | **2009** | 2012 | ▂▂▂▂▂▂▂▂▂▂▂▂▂▂▂▂▂▂▂▃▃▃▃▂▂▂▂ |
| Virtual Real Med Ctr | 1990 | 3.8717 | **2009** | 2010 | ▂▂▂▂▂▂▂▂▂▂▂▂▂▂▂▂▂▂▂▃▃▂▂▂▂▂▂ |
| Univ Alabama | 1990 | 3.5019 | **2009** | 2011 | ▂▂▂▂▂▂▂▂▂▂▂▂▂▂▂▂▂▂▂▃▃▃▂▂▂▂▂ |
| Rensselaer Polytech Inst | 1990 | 3.8725 | **2009** | 2016 | ▂▂▂▂▂▂▂▂▂▂▂▂▂▂▂▂▂▂▂▃▃▃▃▃▃▃▃ |
| Univ Babes Bolyai | 1990 | 3.7202 | **2009** | 2016 | ▂▂▂▂▂▂▂▂▂▂▂▂▂▂▂▂▂▂▂▃▃▃▃▃▃▃▃ |
| Univ Barcelona | 1990 | 18.7074 | **2010** | 2016 | ▂▂▂▂▂▂▂▂▂▂▂▂▂▂▂▂▂▂▂▂▃▃▃▃▃▃▃ |
| Radboud Univ Nijmegen | 1990 | 3.4984 | **2010** | 2014 | ▂▂▂▂▂▂▂▂▂▂▂▂▂▂▂▂▂▂▂▂▃▃▃▃▃▂▂ |
| Univ Tecn Lisboa | 1990 | 5.3606 | **2010** | 2012 | ▂▂▂▂▂▂▂▂▂▂▂▂▂▂▂▂▂▂▂▂▃▃▃▂▂▂▂ |
| Tech Univ Kosice | 1990 | 6.147 | **2010** | 2014 | ▂▂▂▂▂▂▂▂▂▂▂▂▂▂▂▂▂▂▂▂▃▃▃▃▃▂▂ |
| VTT Tech Res Ctr Finland | 1990 | 4.5007 | **2010** | 2011 | ▂▂▂▂▂▂▂▂▂▂▂▂▂▂▂▂▂▂▂▂▃▃▂▂▂▂▂ |
| Univ Cincinnati | 1990 | 3.3944 | **2010** | 2012 | ▂▂▂▂▂▂▂▂▂▂▂▂▂▂▂▂▂▂▂▂▃▃▃▂▂▂▂ |
| Purdue Univ Calumet | 1990 | 6.6426 | **2010** | 2014 | ▂▂▂▂▂▂▂▂▂▂▂▂▂▂▂▂▂▂▂▂▃▃▃▃▃▂▂ |
| Transilvania Univ Brasov | 1990 | 6.2007 | **2010** | 2014 | ▂▂▂▂▂▂▂▂▂▂▂▂▂▂▂▂▂▂▂▂▃▃▃▃▃▂▂ |
| Kings Coll London | 1990 | 7.34 | **2010** | 2016 | ▂▂▂▂▂▂▂▂▂▂▂▂▂▂▂▂▂▂▂▂▃▃▃▃▃▃▃ |
| Tsinghua Univ | 1990 | 4.5552 | **2010** | 2014 | ▂▂▂▂▂▂▂▂▂▂▂▂▂▂▂▂▂▂▂▂▃▃▃▃▃▂▂ |
| Univ Exeter | 1990 | 4.2993 | **2011** | 2014 | ▂▂▂▂▂▂▂▂▂▂▂▂▂▂▂▂▂▂▂▂▂▃▃▃▃▂▂ |
| Northwestern Univ | 1990 | 5.6576 | **2011** | 2016 | ▂▂▂▂▂▂▂▂▂▂▂▂▂▂▂▂▂▂▂▂▂▃▃▃▃▃▃ |
| Flinders Univ S Australia | 1990 | 4.8999 | **2011** | 2016 | ▂▂▂▂▂▂▂▂▂▂▂▂▂▂▂▂▂▂▂▂▂▃▃▃▃▃▃ |
| Univ Almeria | 1990 | 3.4317 | **2011** | 2016 | ▂▂▂▂▂▂▂▂▂▂▂▂▂▂▂▂▂▂▂▂▂▃▃▃▃▃▃ |
| Univ Porto | 1990 | 6.3841 | **2011** | 2014 | ▂▂▂▂▂▂▂▂▂▂▂▂▂▂▂▂▂▂▂▂▂▃▃▃▃▂▂ |
| Scuola Super Sant Anna | 1990 | 4.5915 | **2011** | 2016 | ▂▂▂▂▂▂▂▂▂▂▂▂▂▂▂▂▂▂▂▂▂▃▃▃▃▃▃ |
| Islamic Azad Univ | 1990 | 3.6222 | **2011** | 2012 | ▂▂▂▂▂▂▂▂▂▂▂▂▂▂▂▂▂▂▂▂▂▃▃▂▂▂▂ |
| Univ Milan | 1990 | 3.792 | **2012** | 2014 | ▂▂▂▂▂▂▂▂▂▂▂▂▂▂▂▂▂▂▂▂▂▂▃▃▃▂▂ |
| Sahmyook Univ | 1990 | 6.5133 | **2012** | 2016 | ▂▂▂▂▂▂▂▂▂▂▂▂▂▂▂▂▂▂▂▂▂▂▃▃▃▃▃ |
| Univ Toronto | 1990 | 4.4001 | **2012** | 2014 | ▂▂▂▂▂▂▂▂▂▂▂▂▂▂▂▂▂▂▂▂▂▂▃▃▃▂▂ |
| Heidelberg Univ | 1990 | 6.5133 | **2012** | 2016 | ▂▂▂▂▂▂▂▂▂▂▂▂▂▂▂▂▂▂▂▂▂▂▃▃▃▃▃ |
| Ecole Polytech Fed Lausanne | 1990 | 4.6775 | **2012** | 2013 | ▂▂▂▂▂▂▂▂▂▂▂▂▂▂▂▂▂▂▂▂▂▂▃▃▂▂▂ |
| Univ Wurzburg | 1990 | 11.0738 | **2012** | 2016 | ▂▂▂▂▂▂▂▂▂▂▂▂▂▂▂▂▂▂▂▂▂▂▃▃▃▃▃ |
| Natl Univ Ireland Univ Coll Cork | 1990 | 5.6071 | **2012** | 2014 | ▂▂▂▂▂▂▂▂▂▂▂▂▂▂▂▂▂▂▂▂▂▂▃▃▃▂▂ |
| Mayo Clin | 1990 | 5.3446 | **2013** | 2014 | ▂▂▂▂▂▂▂▂▂▂▂▂▂▂▂▂▂▂▂▂▂▂▂▃▃▂▂ |
| Univ Sao Paulo | 1990 | 6.6166 | **2013** | 2014 | ▂▂▂▂▂▂▂▂▂▂▂▂▂▂▂▂▂▂▂▂▂▂▂▃▃▂▂ |
| Univ Roma La Sapienza | 1990 | 5.6712 | **2013** | 2016 | ▂▂▂▂▂▂▂▂▂▂▂▂▂▂▂▂▂▂▂▂▂▂▂▃▃▃▃ |
| Harvard Univ | 1990 | 5.8889 | **2013** | 2016 | ▂▂▂▂▂▂▂▂▂▂▂▂▂▂▂▂▂▂▂▂▂▂▂▃▃▃▃ |
| Univ Bern | 1990 | 3.6487 | **2013** | 2016 | ▂▂▂▂▂▂▂▂▂▂▂▂▂▂▂▂▂▂▂▂▂▂▂▃▃▃▃ |
| Natl Cent Univ | 1990 | 3.4701 | **2013** | 2014 | ▂▂▂▂▂▂▂▂▂▂▂▂▂▂▂▂▂▂▂▂▂▂▂▃▃▂▂ |
| Univ Oxford | 1990 | 3.5687 | **2013** | 2016 | ▂▂▂▂▂▂▂▂▂▂▂▂▂▂▂▂▂▂▂▂▂▂▂▃▃▃▃ |
| Univ Pisa | 1990 | 5.4917 | **2013** | 2014 | ▂▂▂▂▂▂▂▂▂▂▂▂▂▂▂▂▂▂▂▂▂▂▂▃▃▂▂ |
| Aix Marseille Univ | 1990 | 7.9124 | **2013** | 2016 | ▂▂▂▂▂▂▂▂▂▂▂▂▂▂▂▂▂▂▂▂▂▂▂▃▃▃▃ |
| Vrije Univ Amsterdam | 1990 | 6.0662 | **2013** | 2016 | ▂▂▂▂▂▂▂▂▂▂▂▂▂▂▂▂▂▂▂▂▂▂▂▃▃▃▃ |
| Inria | 1990 | 3.5574 | **2013** | 2016 | ▂▂▂▂▂▂▂▂▂▂▂▂▂▂▂▂▂▂▂▂▂▂▂▃▃▃▃ |
| Univ Queensland | 1990 | 4.5026 | **2014** | 2016 | ▂▂▂▂▂▂▂▂▂▂▂▂▂▂▂▂▂▂▂▂▂▂▂▂▃▃▃ |
| Univ London Imperial Coll Sci Technol & Med | 1990 | 4.4224 | **2014** | 2016 | ▂▂▂▂▂▂▂▂▂▂▂▂▂▂▂▂▂▂▂▂▂▂▂▂▃▃▃ |
| Univ Alabama Birmingham | 1990 | 4.3027 | **2014** | 2016 | ▂▂▂▂▂▂▂▂▂▂▂▂▂▂▂▂▂▂▂▂▂▂▂▂▃▃▃ |
| Univ Lisbon | 1990 | 4.7623 | **2014** | 2016 | ▂▂▂▂▂▂▂▂▂▂▂▂▂▂▂▂▂▂▂▂▂▂▂▂▃▃▃ |
| Univ Copenhagen | 1990 | 6.5131 | **2014** | 2016 | ▂▂▂▂▂▂▂▂▂▂▂▂▂▂▂▂▂▂▂▂▂▂▂▂▃▃▃ |
| Aalborg Univ | 1990 | 7.9562 | **2014** | 2016 | ▂▂▂▂▂▂▂▂▂▂▂▂▂▂▂▂▂▂▂▂▂▂▂▂▃▃▃ |
| Univ Regensburg | 1990 | 6.7233 | **2014** | 2016 | ▂▂▂▂▂▂▂▂▂▂▂▂▂▂▂▂▂▂▂▂▂▂▂▂▃▃▃ |
