# Supplementary material for: The Past, Present, and Future of Virtual and Augmented Reality Research: A Network and Cluster Analysis of the Literature
Source: Front Psychol. 2018 Nov 6;9:2086. doi: 10.3389/fpsyg.2018.02086 (PMC6232426; doi:10.3389/fpsyg.2018.02086)
Supplement: Supplementary file 1 [file Data_Sheet_1.ZIP › Top 394 References (AR) with Strongest Citation Bursts.docx]

**Top 394 References with Strongest Citation Bursts**

| **References** | **Year** | **Strength** | **Begin** | **End** | **1990 - 2017** |
| --- | --- | --- | --- | --- | --- |
| MEYER K, 1992, PRESENCE, V1, P173 | 1992 | 7.6235 | **1990** | 2000 | ▂▂▃▃▃▃▃▃▃▃▃▃▃▂▂▂▂▂▂▂▂▂▂▂▂▂▂▂▂▂ |
| WEISER M, 1991, SCI AM, V265, P94, [DOI](http://dx.doi.org/10.1038%2FSCIENTIFICAMERICAN0991-94) | 1991 | 6.7308 | **1990** | 1999 | ▂▃▃▃▃▃▃▃▃▃▃▂▂▂▂▂▂▂▂▂▂▂▂▂▂▂▂▂▂ |
| DEMENTHON DF, 1995, INT J COMPUT VISION, V15, P123, [DOI](http://dx.doi.org/10.1007%2FBF01450852) | 1995 | 5.7341 | **1994** | 2003 | ▂▂▂▂▂▃▃▃▃▃▃▃▃▃▃▂▂▂▂▂▂▂▂▂▂▂▂▂▂ |
| AZUMA R, 1994, COMPUTER GRAPHICS, V, P194 | 1994 | 4.6391 | **1994** | 2002 | ▂▂▂▂▃▃▃▃▃▃▃▃▃▂▂▂▂▂▂▂▂▂▂▂▂▂▂▂ |
| DEERING M, 1992, COMP GRAPH, V26, P195 | 1992 | 6.4825 | **1994** | 2000 | ▂▂▂▂▃▃▃▃▃▃▃▂▂▂▂▂▂▂▂▂▂▂▂▂▂▂▂▂ |
| WENG JY, 1992, IEEE T PATTERN ANAL, V14, P965, [DOI](http://dx.doi.org/10.1109%2F34.159901) | 1992 | 8.2536 | **1994** | 2000 | ▂▂▂▂▃▃▃▃▃▃▃▂▂▂▂▂▂▂▂▂▂▂▂▂▂▂▂▂ |
| JANIN AL, 1993, P IEEE VIRT REAL ANN, V, P246, [DOI](http://dx.doi.org/10.1109%2FVRAIS.1993.380772) | 1993 | 9.4073 | **1994** | 2001 | ▂▂▂▂▃▃▃▃▃▃▃▃▂▂▂▂▂▂▂▂▂▂▂▂▂▂▂▂ |
| GOTTSCHALK S, 1993, COMPUTER GRAPHICS PROCEEDINGS, V, P65 | 1993 | 5.5287 | **1994** | 2001 | ▂▂▂▂▃▃▃▃▃▃▃▃▂▂▂▂▂▂▂▂▂▂▂▂▂▂▂▂ |
| MAYBANK SJ, 1992, INT J COMPUT VISION, V8, P123, [DOI](http://dx.doi.org/10.1007%2FBF00127171) | 1992 | 3.5336 | **1994** | 2000 | ▂▂▂▂▃▃▃▃▃▃▃▂▂▂▂▂▂▂▂▂▂▂▂▂▂▂▂▂ |
| FEINER S, 1993, COMMUNICATIONS OF THE ACM, V36, P52 | 1993 | 11.6267 | **1994** | 2001 | ▂▂▂▂▃▃▃▃▃▃▃▃▂▂▂▂▂▂▂▂▂▂▂▂▂▂▂▂ |
| FOURNIER A, 1994, JOURNEE INRIA ANAL S, V, P1 | 1994 | 4.1231 | **1994** | 2000 | ▂▂▂▂▃▃▃▃▃▃▃▂▂▂▂▂▂▂▂▂▂▂▂▂▂▂▂▂ |
| FEINER S, 1993, COMMUN ACM, V36, P53, [DOI](http://dx.doi.org/10.1145%2F159544.159587) | 1993 | 29.4645 | **1994** | 2001 | ▂▂▂▂▃▃▃▃▃▃▃▃▂▂▂▂▂▂▂▂▂▂▂▂▂▂▂▂ |
| FEINER STEVEN, 1993, P 6 ANN ACM S US INT, V, P145 | 1993 | 3.8686 | **1994** | 2001 | ▂▂▂▂▃▃▃▃▃▃▃▃▂▂▂▂▂▂▂▂▂▂▂▂▂▂▂▂ |
| BAJURA M, 1995, PROCEEDINGS. VIRTUAL REALITY ANNUAL INTERNATIONAL SYMPOSIUM 95 (CAT. NO.95CH35761), V, P189, [DOI](http://dx.doi.org/10.1109%2FVRAIS.1995.512495) | 1995 | 6.2128 | **1994** | 2003 | ▂▂▂▂▂▃▃▃▃▃▃▃▃▃▃▂▂▂▂▂▂▂▂▂▂▂▂▂▂ |
| WELLNER P, 1993, COMMUN ACM, V36, P87, [DOI](http://dx.doi.org/10.1145%2F159544.159630) | 1993 | 11.6267 | **1994** | 2001 | ▂▂▂▂▃▃▃▃▃▃▃▃▂▂▂▂▂▂▂▂▂▂▂▂▂▂▂▂ |
| BAJURA M, 1992, COMP GRAPH, V26, P203 | 1992 | 19.165 | **1996** | 2000 | ▂▂▂▂▂▂▃▃▃▃▃▂▂▂▂▂▂▂▂▂▂▂▂▂▂▂▂▂ |
| TUCERYAN M, 1995, IEEE T VIS COMPUT GR, V1, P255, [DOI](http://dx.doi.org/10.1109%2F2945.466720) | 1995 | 15.5858 | **1996** | 2003 | ▂▂▂▂▂▂▃▃▃▃▃▃▃▃▂▂▂▂▂▂▂▂▂▂▂▂▂▂ |
| BAJURA M, 1992, P SIGGRAPH 92, V, P203 | 1992 | 4.7765 | **1996** | 2000 | ▂▂▂▂▂▂▃▃▃▃▃▂▂▂▂▂▂▂▂▂▂▂▂▂▂▂▂▂ |
| HOLLOWAY RL, 1995, THESIS U N CAROLINA, V, P | 1995 | 6.8785 | **1997** | 2003 | ▂▂▂▂▂▂▂▃▃▃▃▃▃▃▂▂▂▂▂▂▂▂▂▂▂▂▂▂ |
| BECKER D E, 1995, PROCEEDINGS. INTERNATIONAL CONFERENCE ON IMAGE PROCESSING (CAT. NO.95CB35819), V, P426, [DOI](http://dx.doi.org/10.1109%2FICIP.1995.529737) | 1995 | 3.3956 | **1997** | 2001 | ▂▂▂▂▂▂▂▃▃▃▃▃▂▂▂▂▂▂▂▂▂▂▂▂▂▂▂▂ |
| KUTULAKOS KN, 1996, P IEEE VIRT REAL ANN, V, P25, [DOI](http://dx.doi.org/10.1109%2FVRAIS.1996.490507) | 1996 | 6.3239 | **1997** | 2004 | ▂▂▂▂▂▂▂▃▃▃▃▃▃▃▃▂▂▂▂▂▂▂▂▂▂▂▂▂ |
| GHAZISAEDY M, 1995, PROCEEDINGS. VIRTUAL REALITY ANNUAL INTERNATIONAL SYMPOSIUM 95 (CAT. NO.95CH35761), V, P179, [DOI](http://dx.doi.org/10.1109%2FVRAIS.1995.512494) | 1995 | 4.4191 | **1997** | 2003 | ▂▂▂▂▂▂▂▃▃▃▃▃▃▃▂▂▂▂▂▂▂▂▂▂▂▂▂▂ |
| HARALICK RM, 1994, INT J COMPUT VISION, V13, P331 | 1994 | 5.8183 | **1997** | 2002 | ▂▂▂▂▂▂▂▃▃▃▃▃▃▂▂▂▂▂▂▂▂▂▂▂▂▂▂▂ |
| WATSON B A, 1995, PROCEEDINGS. VIRTUAL REALITY ANNUAL INTERNATIONAL SYMPOSIUM 95 (CAT. NO.95CH35761), V, P172, [DOI](http://dx.doi.org/10.1109%2FVRAIS.1995.512493) | 1995 | 3.9276 | **1997** | 2003 | ▂▂▂▂▂▂▂▃▃▃▃▃▃▃▂▂▂▂▂▂▂▂▂▂▂▂▂▂ |
| BAJURA M, 1995, IEEE COMPUT GRAPH, V15, P52, [DOI](http://dx.doi.org/10.1109%2F38.403828) | 1995 | 16.7474 | **1997** | 2003 | ▂▂▂▂▂▂▂▃▃▃▃▃▃▃▂▂▂▂▂▂▂▂▂▂▂▂▂▂ |
| WARD M, 1992, P 1992 S INT 3D GRAP, V, P43 | 1992 | 4.2165 | **1997** | 2000 | ▂▂▂▂▂▂▂▃▃▃▃▂▂▂▂▂▂▂▂▂▂▂▂▂▂▂▂▂ |
| AZARBAYEJANI A, 1995, IEEE T PATTERN ANAL, V17, P562, [DOI](http://dx.doi.org/10.1109%2F34.387503) | 1995 | 3.9276 | **1997** | 2003 | ▂▂▂▂▂▂▂▃▃▃▃▃▃▃▂▂▂▂▂▂▂▂▂▂▂▂▂▂ |
| BOWSKILL J, 1995, COMPUTER GRAPHICS, V29, P61 | 1995 | 3.3956 | **1997** | 2001 | ▂▂▂▂▂▂▂▃▃▃▃▃▂▂▂▂▂▂▂▂▂▂▂▂▂▂▂▂ |
| REKIMOTO J, 1995, P UIST 95, V, P29 | 1995 | 8.8482 | **1997** | 2003 | ▂▂▂▂▂▂▂▃▃▃▃▃▃▃▂▂▂▂▂▂▂▂▂▂▂▂▂▂ |
| CAUDELL T, 1992, P HAW INT C SYST SCI, V, P | 1992 | 9.6488 | **1997** | 2000 | ▂▂▂▂▂▂▂▃▃▃▃▂▂▂▂▂▂▂▂▂▂▂▂▂▂▂▂▂ |
| GRIMSON WEL, 1995, P COMPUTER VISION VI, V, P3 | 1995 | 5.8183 | **1997** | 2002 | ▂▂▂▂▂▂▂▃▃▃▃▃▃▂▂▂▂▂▂▂▂▂▂▂▂▂▂▂ |
| MILGRAM P, 1994, IEICE T INF SYST, VE77D, P1321 | 1994 | 8.4684 | **1997** | 2002 | ▂▂▂▂▂▂▂▃▃▃▃▃▃▂▂▂▂▂▂▂▂▂▂▂▂▂▂▂ |
| UENOHARA M, 1995, P COMP VIS VIRT REAL, V, P13 | 1995 | 12.7938 | **1997** | 2003 | ▂▂▂▂▂▂▂▃▃▃▃▃▃▃▂▂▂▂▂▂▂▂▂▂▂▂▂▂ |
| STATE A, 1996, ANN C SERIES, V, P439 | 1996 | 4.5148 | **1997** | 2004 | ▂▂▂▂▂▂▂▃▃▃▃▃▃▃▃▂▂▂▂▂▂▂▂▂▂▂▂▂ |
| MELLOR JP, 1995, P COMP VIS VIRT REAL, V, P471 | 1995 | 5.4024 | **1997** | 2003 | ▂▂▂▂▂▂▂▃▃▃▃▃▃▃▂▂▂▂▂▂▂▂▂▂▂▂▂▂ |
| GRIMSON WEL, 1996, IEEE T MED IMAGING, V15, P129, [DOI](http://dx.doi.org/10.1109%2F42.491415) | 1996 | 9.9476 | **1997** | 2004 | ▂▂▂▂▂▂▂▃▃▃▃▃▃▃▃▂▂▂▂▂▂▂▂▂▂▂▂▂ |
| MILGRAM P, 1993, P INT C INT ROB SYST, V, P1467 | 1993 | 3.6137 | **1997** | 2000 | ▂▂▂▂▂▂▂▃▃▃▃▂▂▂▂▂▂▂▂▂▂▂▂▂▂▂▂▂ |
| AZUMA R, 1993, COMMUN ACM, V36, P50, [DOI](http://dx.doi.org/10.1145%2F159544.159581) | 1993 | 6.2292 | **1997** | 2001 | ▂▂▂▂▂▂▂▃▃▃▃▃▂▂▂▂▂▂▂▂▂▂▂▂▂▂▂▂ |
| FITZMAURICE GW, 1993, COMMUN ACM, V36, P39, [DOI](http://dx.doi.org/10.1145%2F159544.159566) | 1993 | 5.6622 | **1997** | 2001 | ▂▂▂▂▂▂▂▃▃▃▃▃▂▂▂▂▂▂▂▂▂▂▂▂▂▂▂▂ |
| ROLLAND JP, 1995, PRESENCE-TELEOP VIRT, V4, P24, [DOI](http://dx.doi.org/10.1162%2FPRES.1995.4.1.24) | 1995 | 6.8779 | **1997** | 2002 | ▂▂▂▂▂▂▂▃▃▃▃▃▃▂▂▂▂▂▂▂▂▂▂▂▂▂▂▂ |
| FAUGERAS O, 1993, 3 DIMENSIONAL COMPUT, V, P | 1993 | 13.0448 | **1997** | 2001 | ▂▂▂▂▂▂▂▃▃▃▃▃▂▂▂▂▂▂▂▂▂▂▂▂▂▂▂▂ |
| AZUMA RONALD T, 1994, P SIGGRAPH 94, V, P197 | 1994 | 12.7156 | **1997** | 2002 | ▂▂▂▂▂▂▂▃▃▃▃▃▃▂▂▂▂▂▂▂▂▂▂▂▂▂▂▂ |
| MILGRAM P, 1994, P SOC PHOTO-OPT INS, V2351, P282 | 1994 | 8.4684 | **1997** | 2002 | ▂▂▂▂▂▂▂▃▃▃▃▃▃▂▂▂▂▂▂▂▂▂▂▂▂▂▂▂ |
| WLOKA M W, 1995, PROCEEDINGS 1995 SYMPOSIUM ON INTERACTIVE 3D GRAPHICS, V, P5, [DOI](http://dx.doi.org/10.1145%2F199404.199405) | 1995 | 6.1022 | **1997** | 2001 | ▂▂▂▂▂▂▂▃▃▃▃▃▂▂▂▂▂▂▂▂▂▂▂▂▂▂▂▂ |
| ISEKI H, 1996, P INT C VIRT SYST MU, V, P97 | 1996 | 3.3956 | **1997** | 2001 | ▂▂▂▂▂▂▂▃▃▃▃▃▂▂▂▂▂▂▂▂▂▂▂▂▂▂▂▂ |
| REKIMOTO J, 1997, PRESENCE-TELEOP VIRT, V6, P399, [DOI](http://dx.doi.org/10.1162%2FPRES.1997.6.4.399) | 1997 | 7.278 | **1998** | 2005 | ▂▂▂▂▂▂▂▂▃▃▃▃▃▃▃▃▂▂▂▂▂▂▂▂▂▂▂▂ |
| KLINKER GJ, 1997, PRESENCE-TELEOP VIRT, V6, P433, [DOI](http://dx.doi.org/10.1162%2FPRES.1997.6.4.433) | 1997 | 8.1363 | **1998** | 2005 | ▂▂▂▂▂▂▂▂▃▃▃▃▃▃▃▃▂▂▂▂▂▂▂▂▂▂▂▂ |
| AZUMA RT, 1997, PRESENCE-TELEOP VIRT, V6, P355, [DOI](http://dx.doi.org/10.1162%2FPRES.1997.6.4.355) | 1997 | 101.6424 | **1998** | 2005 | ▂▂▂▂▂▂▂▂▃▃▃▃▃▃▃▃▂▂▂▂▂▂▂▂▂▂▂▂ |
| STATE A, 1994, PROCEEDINGS. VISUALIZATION 94 (CAT. NO.94CH35707), V, P364, [DOI](http://dx.doi.org/10.1109%2FVISUAL.1994.346295) | 1994 | 4.3402 | **1998** | 2002 | ▂▂▂▂▂▂▂▂▃▃▃▃▃▂▂▂▂▂▂▂▂▂▂▂▂▂▂▂ |
| STATE A, 1996, P SIGGRAPH 96, V, P439 | 1996 | 15.8675 | **1998** | 2004 | ▂▂▂▂▂▂▂▂▃▃▃▃▃▃▃▂▂▂▂▂▂▂▂▂▂▂▂▂ |
| SHARMA R, 1997, PRESENCE-TELEOP VIRT, V6, P292, [DOI](http://dx.doi.org/10.1162%2FPRES.1997.6.3.292) | 1997 | 6.9835 | **1998** | 2004 | ▂▂▂▂▂▂▂▂▃▃▃▃▃▃▃▂▂▂▂▂▂▂▂▂▂▂▂▂ |
| STATE A, 1996, P SIGGRAPH 96, V,, P | 1996 | 28.3395 | **1998** | 2004 | ▂▂▂▂▂▂▂▂▃▃▃▃▃▃▃▂▂▂▂▂▂▂▂▂▂▂▂▂ |
| OHTA Y, 1999, MIXED REALITY MERGIN, V, P | 1999 | 6.3705 | **1999** | 2002 | ▂▂▂▂▂▂▂▂▂▃▃▃▃▂▂▂▂▂▂▂▂▂▂▂▂▂▂▂ |
| WAGNER A, 1997, J ORAL MAXIL SURG, V55, P456, [DOI](http://dx.doi.org/10.1016%2FS0278-2391%2897%2990689-3) | 1997 | 3.9507 | **1999** | 2005 | ▂▂▂▂▂▂▂▂▂▃▃▃▃▃▃▃▂▂▂▂▂▂▂▂▂▂▂▂ |
| OHSHIMA T, 1998, P IEEE VIRT REAL ANN, V, P268, [DOI](http://dx.doi.org/10.1109%2FVRAIS.1998.658505) | 1998 | 10.3984 | **1999** | 2002 | ▂▂▂▂▂▂▂▂▂▃▃▃▃▂▂▂▂▂▂▂▂▂▂▂▂▂▂▂ |
| REKIMOTO J, 1998, P 2 INT S WEAR COMP, V, P68 | 1998 | 4.291 | **1999** | 2002 | ▂▂▂▂▂▂▂▂▂▃▃▃▃▂▂▂▂▂▂▂▂▂▂▂▂▂▂▂ |
| WELCH G, 1997, P SIGGRAPH 97, V,, P | 1997 | 5.811 | **1999** | 2001 | ▂▂▂▂▂▂▂▂▂▃▃▃▂▂▂▂▂▂▂▂▂▂▂▂▂▂▂▂ |
| HOFF WA, 1996, P SOC PHOTO-OPT INS, V2904, P538, [DOI](http://dx.doi.org/10.1117%2F12.256311) | 1996 | 8.1087 | **1999** | 2004 | ▂▂▂▂▂▂▂▂▂▃▃▃▃▃▃▂▂▂▂▂▂▂▂▂▂▂▂▂ |
| WEBSTER A, 1996, P ASCE 3 C COMP CIV, V, P913 | 1996 | 8.5868 | **1999** | 2004 | ▂▂▂▂▂▂▂▂▂▃▃▃▃▃▃▂▂▂▂▂▂▂▂▂▂▂▂▂ |
| HOLLOWAY RL, 1997, PRESENCE-TELEOP VIRT, V6, P413, [DOI](http://dx.doi.org/10.1162%2FPRES.1997.6.4.413) | 1997 | 8.1087 | **1999** | 2004 | ▂▂▂▂▂▂▂▂▂▃▃▃▃▃▃▂▂▂▂▂▂▂▂▂▂▂▂▂ |
| ULLMER B, 1997, PROCEEDINGS OF THE ACM SYMPOSIUM ON USER INTERFACE SOFTWARE AND TECHNOLOGY. 10TH ANNUAL SYMPOSIUM. UIST 97, V, P223, [DOI](http://dx.doi.org/10.1145%2F263407.263551) | 1997 | 6.1495 | **1999** | 2005 | ▂▂▂▂▂▂▂▂▂▃▃▃▃▃▃▃▂▂▂▂▂▂▂▂▂▂▂▂ |
| EDWARDS P J, 1995, J IMAGE GUID SURG, V1, P172, [DOI](http://dx.doi.org/10.1002%2F%28SICI%291522-712X%281995%291%3A3%3C172%3A%3AAID-IGS7%3E3.0.CO%3B2-7) | 1995 | 7.2298 | **1999** | 2003 | ▂▂▂▂▂▂▂▂▂▃▃▃▃▃▂▂▂▂▂▂▂▂▂▂▂▂▂▂ |
| NEUMANN U, 1998, P IEEE VIRT REAL ANN, V, P148, [DOI](http://dx.doi.org/10.1109%2FVRAIS.1998.658482) | 1998 | 5.2428 | **1999** | 2004 | ▂▂▂▂▂▂▂▂▂▃▃▃▃▃▃▂▂▂▂▂▂▂▂▂▂▂▂▂ |
| AZUMA R, 1999, P IEEE VIRT REAL ANN, V, P252, [DOI](http://dx.doi.org/10.1109%2FVR.1999.756959) | 1999 | 10.5461 | **1999** | 2007 | ▂▂▂▂▂▂▂▂▂▃▃▃▃▃▃▃▃▃▂▂▂▂▂▂▂▂▂▂ |
| OKUMA T, 1998, P 14 IAPR INT C PATT, V2, P1226 | 1998 | 5.2428 | **1999** | 2004 | ▂▂▂▂▂▂▂▂▂▃▃▃▃▃▃▂▂▂▂▂▂▂▂▂▂▂▂▂ |
| FEINER STEVEN, 1997, P 1 INT S WEAR COMP, V, P74 | 1997 | 16.3003 | **1999** | 2004 | ▂▂▂▂▂▂▂▂▂▃▃▃▃▃▃▂▂▂▂▂▂▂▂▂▂▂▂▂ |
| HARTLEY RI, 1997, IEEE T PATTERN ANAL, V19, P580, [DOI](http://dx.doi.org/10.1109%2F34.601246) | 1997 | 4.3903 | **1999** | 2005 | ▂▂▂▂▂▂▂▂▂▃▃▃▃▃▃▃▂▂▂▂▂▂▂▂▂▂▂▂ |
| FITZPATRICK JM, 1998, IEEE T MED IMAGING, V17, P694, [DOI](http://dx.doi.org/10.1109%2F42.736021) | 1998 | 5.9993 | **1999** | 2006 | ▂▂▂▂▂▂▂▂▂▃▃▃▃▃▃▃▃▂▂▂▂▂▂▂▂▂▂▂ |
| NEUMANN U, 1996, P ACM VIRT REAL SOFT, V, P109 | 1996 | 13.6132 | **1999** | 2003 | ▂▂▂▂▂▂▂▂▂▃▃▃▃▃▂▂▂▂▂▂▂▂▂▂▂▂▂▂ |
| TANG SL, 1998, IEEE ENG MED BIOL, V17, P49, [DOI](http://dx.doi.org/10.1109%2F51.677169) | 1998 | 6.8009 | **1999** | 2006 | ▂▂▂▂▂▂▂▂▂▃▃▃▃▃▃▃▃▂▂▂▂▂▂▂▂▂▂▂ |
| NEUMANN U, 1998, P IEEE VIRT REAL ANN, V, P4, [DOI](http://dx.doi.org/10.1109%2FVRAIS.1998.658416) | 1998 | 5.2697 | **1999** | 2005 | ▂▂▂▂▂▂▂▂▂▃▃▃▃▃▃▃▂▂▂▂▂▂▂▂▂▂▂▂ |
| SZALAVARI Z, 1998, VIRTUAL REALITY, V3, P37, [DOI](http://dx.doi.org/10.1007%2FBF01409796) | 1998 | 4.5487 | **1999** | 2004 | ▂▂▂▂▂▂▂▂▂▃▃▃▃▃▃▂▂▂▂▂▂▂▂▂▂▂▂▂ |
| PFEIFER G, 1998, P 25 ANN C COMP GRAP, V, P371 | 1998 | 6.1976 | **1999** | 2004 | ▂▂▂▂▂▂▂▂▂▃▃▃▃▃▃▂▂▂▂▂▂▂▂▂▂▂▂▂ |
| STARNER T, 1997, PRESENCE-TELEOP VIRT, V6, P386, [DOI](http://dx.doi.org/10.1162%2FPRES.1997.6.4.386) | 1997 | 10.5553 | **1999** | 2005 | ▂▂▂▂▂▂▂▂▂▃▃▃▃▃▃▃▂▂▂▂▂▂▂▂▂▂▂▂ |
| KANADE T, 1996, PROC CVPR IEEE, V, P196, [DOI](http://dx.doi.org/10.1109%2FCVPR.1996.517074) | 1996 | 3.8762 | **1999** | 2002 | ▂▂▂▂▂▂▂▂▂▃▃▃▃▂▂▂▂▂▂▂▂▂▂▂▂▂▂▂ |
| KUTULAKOS KN, 1998, IEEE T VIS COMPUT GR, V4, P1, [DOI](http://dx.doi.org/10.1109%2F2945.675647) | 1998 | 17.6623 | **1999** | 2006 | ▂▂▂▂▂▂▂▂▂▃▃▃▃▃▃▃▃▂▂▂▂▂▂▂▂▂▂▂ |
| BERGER JW, 1997, LECT NOTES COMPUT SC, V1205, P399 | 1997 | 3.546 | **1999** | 2001 | ▂▂▂▂▂▂▂▂▂▃▃▃▂▂▂▂▂▂▂▂▂▂▂▂▂▂▂▂ |
| YOU S, 1999, P IEEE VIRT REAL ANN, V, P260, [DOI](http://dx.doi.org/10.1109%2FVR.1999.756960) | 1999 | 12.3722 | **1999** | 2007 | ▂▂▂▂▂▂▂▂▂▃▃▃▃▃▃▃▃▃▂▂▂▂▂▂▂▂▂▂ |
| KOLLER D, 1997, P ACM S VIRT REAL SO, V, P87 | 1997 | 7.0298 | **1999** | 2005 | ▂▂▂▂▂▂▂▂▂▃▃▃▃▃▃▃▂▂▂▂▂▂▂▂▂▂▂▂ |
| NEUMANN U, 1999, IEEE T MULTIMEDIA, V1, P53, [DOI](http://dx.doi.org/10.1109%2F6046.748171) | 1999 | 9.8181 | **2000** | 2007 | ▂▂▂▂▂▂▂▂▂▂▃▃▃▃▃▃▃▃▂▂▂▂▂▂▂▂▂▂ |
| KANBARA M, 2000, PROCEEDINGS IEEE VIRTUAL REALITY 2000 (CAT. NO.00CB37048), V, P255, [DOI](http://dx.doi.org/10.1109%2FVR.2000.840506) | 2000 | 12.0175 | **2000** | 2005 | ▂▂▂▂▂▂▂▂▂▂▃▃▃▃▃▃▂▂▂▂▂▂▂▂▂▂▂▂ |
| HOLLERER T, 1999, P 3 INT S WEAR COMP, V, P79 | 1999 | 8.8916 | **2000** | 2007 | ▂▂▂▂▂▂▂▂▂▂▃▃▃▃▃▃▃▃▂▂▂▂▂▂▂▂▂▂ |
| YOKOKOHJI Y, 2000, PROCEEDINGS IEEE VIRTUAL REALITY 2000 (CAT. NO.00CB37048), V, P247, [DOI](http://dx.doi.org/10.1109%2FVR.2000.840505) | 2000 | 7.0609 | **2000** | 2004 | ▂▂▂▂▂▂▂▂▂▂▃▃▃▃▃▂▂▂▂▂▂▂▂▂▂▂▂▂ |
| ZHANG Z, 1999, P INT C COMP VIS, V, P666 | 1999 | 7.2713 | **2000** | 2007 | ▂▂▂▂▂▂▂▂▂▂▃▃▃▃▃▃▃▃▂▂▂▂▂▂▂▂▂▂ |
| BAILLOT Y, 2000, PRESENCE-TELEOP VIRT, V9, P223, [DOI](http://dx.doi.org/10.1162%2F105474600566763) | 2000 | 4.6577 | **2000** | 2002 | ▂▂▂▂▂▂▂▂▂▂▃▃▃▂▂▂▂▂▂▂▂▂▂▂▂▂▂▂ |
| SATO I, 1999, IEEE T VIS COMPUT GR, V5, P1, [DOI](http://dx.doi.org/10.1109%2F2945.764865) | 1999 | 3.6315 | **2000** | 2007 | ▂▂▂▂▂▂▂▂▂▂▃▃▃▃▃▃▃▃▂▂▂▂▂▂▂▂▂▂ |
| BLACKWELL M, 1998, CLIN ORTHOP RELAT R, V, P111 | 1998 | 5.2869 | **2000** | 2006 | ▂▂▂▂▂▂▂▂▂▂▃▃▃▃▃▃▃▂▂▂▂▂▂▂▂▂▂▂ |
| ISHII H, 1997, P SIGCHI C HUM FACT, V1997, P234 | 1997 | 14.004 | **2000** | 2005 | ▂▂▂▂▂▂▂▂▂▂▃▃▃▃▃▃▂▂▂▂▂▂▂▂▂▂▂▂ |
| RASKAR R, 1998, P SIGGRAPH 98, V,, P | 1998 | 6.2396 | **2000** | 2005 | ▂▂▂▂▂▂▂▂▂▂▃▃▃▃▃▃▂▂▂▂▂▂▂▂▂▂▂▂ |
| ROLLAND JP, 2000, PRESENCE-TELEOP VIRT, V9, P287, [DOI](http://dx.doi.org/10.1162%2F105474600566808) | 2000 | 7.8317 | **2000** | 2008 | ▂▂▂▂▂▂▂▂▂▂▃▃▃▃▃▃▃▃▃▂▂▂▂▂▂▂▂▂ |
| FITZGIBBON A W, 1998, P EUR C COMP VIS, V, P311 | 1998 | 4.7978 | **2000** | 2005 | ▂▂▂▂▂▂▂▂▂▂▃▃▃▃▃▃▂▂▂▂▂▂▂▂▂▂▂▂ |
| FUCHS H, 1998, LECT NOTES COMPUT SC, V1496, P934 | 1998 | 7.9363 | **2000** | 2006 | ▂▂▂▂▂▂▂▂▂▂▃▃▃▃▃▃▃▂▂▂▂▂▂▂▂▂▂▂ |
| PIEKARSKI W, 1999, PROCEEDINGS 2ND IEEE AND ACM INTERNATIONAL WORKSHOP ON AUGMENTED REALITY (IWAR99), V, P45, [DOI](http://dx.doi.org/10.1109%2FIWAR.1999.803805) | 1999 | 3.6198 | **2000** | 2004 | ▂▂▂▂▂▂▂▂▂▂▃▃▃▃▃▂▂▂▂▂▂▂▂▂▂▂▂▂ |
| HOLLERER T, 1999, COMPUT GRAPH-UK, V23, P779, [DOI](http://dx.doi.org/10.1016%2FS0097-8493%2899%2900103-X) | 1999 | 20.2796 | **2000** | 2007 | ▂▂▂▂▂▂▂▂▂▂▃▃▃▃▃▃▃▃▂▂▂▂▂▂▂▂▂▂ |
| BILLINGHURST M, 1999, P INT S MIX REAL ISM, V, P261 | 1999 | 10.9193 | **2000** | 2007 | ▂▂▂▂▂▂▂▂▂▂▃▃▃▃▃▃▃▃▂▂▂▂▂▂▂▂▂▂ |
| KATO H, 1999, PROCEEDINGS 2ND IEEE AND ACM INTERNATIONAL WORKSHOP ON AUGMENTED REALITY (IWAR99), V, P85, [DOI](http://dx.doi.org/10.1109%2FIWAR.1999.803809) | 1999 | 32.1596 | **2000** | 2007 | ▂▂▂▂▂▂▂▂▂▂▃▃▃▃▃▃▃▃▂▂▂▂▂▂▂▂▂▂ |
| BUTZ A, 1999, PROCEEDINGS 2ND IEEE AND ACM INTERNATIONAL WORKSHOP ON AUGMENTED REALITY (IWAR99), V, P35, [DOI](http://dx.doi.org/10.1109%2FIWAR.1999.803804) | 1999 | 4.0356 | **2000** | 2007 | ▂▂▂▂▂▂▂▂▂▂▃▃▃▃▃▃▃▃▂▂▂▂▂▂▂▂▂▂ |
| SCHMALSTIEG D, 1999, PROCEEDINGS 1999 SYMPOSIUM ON INTERACTIVE 3D GRAPHICS, V, P147, [DOI](http://dx.doi.org/10.1145%2F300523.300542) | 1999 | 3.5664 | **2000** | 2002 | ▂▂▂▂▂▂▂▂▂▂▃▃▃▂▂▂▂▂▂▂▂▂▂▂▂▂▂▂ |
| BEHRINGER R, 1999, P IEEE VIRT REAL ANN, V, P244, [DOI](http://dx.doi.org/10.1109%2FVR.1999.756958) | 1999 | 6.5765 | **2000** | 2007 | ▂▂▂▂▂▂▂▂▂▂▃▃▃▃▃▃▃▃▂▂▂▂▂▂▂▂▂▂ |
| WRIGHT D, 1995, RADIOLOGIC TECHNOLOG, V66, P167 | 1995 | 4.1613 | **2000** | 2002 | ▂▂▂▂▂▂▂▂▂▂▃▃▃▂▂▂▂▂▂▂▂▂▂▂▂▂▂▂ |
| TAKAGI A, 2000, IEEE AND ACM INTERNATIONAL SYMPOSIUM ON AUGMENTED REALITY, V, P68, [DOI](http://dx.doi.org/10.1109%2FISAR.2000.880925) | 2000 | 5.8784 | **2001** | 2005 | ▂▂▂▂▂▂▂▂▂▂▂▃▃▃▃▃▂▂▂▂▂▂▂▂▂▂▂▂ |
| JULIER S, 2000, IEEE AND ACM INTERNATIONAL SYMPOSIUM ON AUGMENTED REALITY, V, P3, [DOI](http://dx.doi.org/10.1109%2FISAR.2000.880917) | 2000 | 9.1618 | **2001** | 2008 | ▂▂▂▂▂▂▂▂▂▂▂▃▃▃▃▃▃▃▃▂▂▂▂▂▂▂▂▂ |
| QUAN L, 1999, IEEE T PATTERN ANAL, V21, P774, [DOI](http://dx.doi.org/10.1109%2F34.784291) | 1999 | 4.7215 | **2001** | 2007 | ▂▂▂▂▂▂▂▂▂▂▂▃▃▃▃▃▃▃▂▂▂▂▂▂▂▂▂▂ |
| TUCERYAN M, 2000, IEEE AND ACM INTERNATIONAL SYMPOSIUM ON AUGMENTED REALITY, V, P149, [DOI](http://dx.doi.org/10.1109%2FISAR.2000.880938) | 2000 | 6.7602 | **2001** | 2004 | ▂▂▂▂▂▂▂▂▂▂▂▃▃▃▃▂▂▂▂▂▂▂▂▂▂▂▂▂ |
| KATO H, 2000, IEEE AND ACM INTERNATIONAL SYMPOSIUM ON AUGMENTED REALITY, V, P111, [DOI](http://dx.doi.org/10.1109%2FISAR.2000.880934) | 2000 | 20.6918 | **2001** | 2008 | ▂▂▂▂▂▂▂▂▂▂▂▃▃▃▃▃▃▃▃▂▂▂▂▂▂▂▂▂ |
| AZUMA R, 1999, COMPUT GRAPH-UK, V23, P787, [DOI](http://dx.doi.org/10.1016%2FS0097-8493%2899%2900104-1) | 1999 | 7.7329 | **2001** | 2007 | ▂▂▂▂▂▂▂▂▂▂▂▃▃▃▃▃▃▃▂▂▂▂▂▂▂▂▂▂ |
| SAUER F, 2000, IEEE AND ACM INTERNATIONAL SYMPOSIUM ON AUGMENTED REALITY, V, P47, [DOI](http://dx.doi.org/10.1109%2FISAR.2000.880922) | 2000 | 8.4112 | **2001** | 2006 | ▂▂▂▂▂▂▂▂▂▂▂▃▃▃▃▃▃▂▂▂▂▂▂▂▂▂▂▂ |
| BIRKFELLNER W, 2001, CLIN ORAL IMPLAN RES, V12, P69, [DOI](http://dx.doi.org/10.1034%2FJ.1600-0501.2001.012001069.X) | 2001 | 3.7991 | **2001** | 2004 | ▂▂▂▂▂▂▂▂▂▂▂▃▃▃▃▂▂▂▂▂▂▂▂▂▂▂▂▂ |
| INAMI M, 2000, PROCEEDINGS IEEE VIRTUAL REALITY 2000 (CAT. NO.00CB37048), V, P233, [DOI](http://dx.doi.org/10.1109%2FVR.2000.840503) | 2000 | 6.4839 | **2001** | 2008 | ▂▂▂▂▂▂▂▂▂▂▂▃▃▃▃▃▃▃▃▂▂▂▂▂▂▂▂▂ |
| FUCHS H, 1998, P 1 INT C MED IM COM, V, P934 | 1998 | 7.9304 | **2001** | 2006 | ▂▂▂▂▂▂▂▂▂▂▂▃▃▃▃▃▃▂▂▂▂▂▂▂▂▂▂▂ |
| DRASCIC D, 1996, P SOC PHOTO-OPT INS, V2653, P123, [DOI](http://dx.doi.org/10.1117%2F12.237425) | 1996 | 6.3665 | **2001** | 2004 | ▂▂▂▂▂▂▂▂▂▂▂▃▃▃▃▂▂▂▂▂▂▂▂▂▂▂▂▂ |
| BERGER JW, 1999, OPHTHALMOLOGY, V106, P1935, [DOI](http://dx.doi.org/10.1016%2FS0161-6420%2899%2990404-9) | 1999 | 3.5365 | **2001** | 2005 | ▂▂▂▂▂▂▂▂▂▂▂▃▃▃▃▃▂▂▂▂▂▂▂▂▂▂▂▂ |
| KATO H, 1999, 2 IEEE ACM INT WORKS, V, P85 | 1999 | 6.0115 | **2001** | 2007 | ▂▂▂▂▂▂▂▂▂▂▂▃▃▃▃▃▃▃▂▂▂▂▂▂▂▂▂▂ |
| SIMON G, 2000, IEEE AND ACM INTERNATIONAL SYMPOSIUM ON AUGMENTED REALITY, V, P120, [DOI](http://dx.doi.org/10.1109%2FISAR.2000.880935) | 2000 | 11.4608 | **2001** | 2008 | ▂▂▂▂▂▂▂▂▂▂▂▃▃▃▃▃▃▃▃▂▂▂▂▂▂▂▂▂ |
| SEO Y, 2000, IEEE T VIS COMPUT GR, V6, P346, [DOI](http://dx.doi.org/10.1109%2F2945.895879) | 2000 | 7.2485 | **2001** | 2008 | ▂▂▂▂▂▂▂▂▂▂▂▃▃▃▃▃▃▃▃▂▂▂▂▂▂▂▂▂ |
| KANBARA M, 2000, IEEE AND ACM INTERNATIONAL SYMPOSIUM ON AUGMENTED REALITY, V, P97, [DOI](http://dx.doi.org/10.1109%2FISAR.2000.880931) | 2000 | 6.4839 | **2001** | 2008 | ▂▂▂▂▂▂▂▂▂▂▂▃▃▃▃▃▃▃▃▂▂▂▂▂▂▂▂▂ |
| CHAI L, 1999, P IEEE INT WORKSH AU, V, P23 | 1999 | 4.36 | **2001** | 2005 | ▂▂▂▂▂▂▂▂▂▂▂▃▃▃▃▃▂▂▂▂▂▂▂▂▂▂▂▂ |
| REKIMOTO J, 1999, P CHI 99, V,, P | 1999 | 5.5814 | **2001** | 2007 | ▂▂▂▂▂▂▂▂▂▂▂▃▃▃▃▃▃▃▂▂▂▂▂▂▂▂▂▂ |
| SCHMALSTIEG D, 2000, IEEE AND ACM INTERNATIONAL SYMPOSIUM ON AUGMENTED REALITY, V, P20, [DOI](http://dx.doi.org/10.1109%2FISAR.2000.880919) | 2000 | 5.8252 | **2001** | 2003 | ▂▂▂▂▂▂▂▂▂▂▂▃▃▃▂▂▂▂▂▂▂▂▂▂▂▂▂▂ |
| BELL B, 2001, P ACM S US INT SOFTW, V, P101 | 2001 | 5.7249 | **2001** | 2009 | ▂▂▂▂▂▂▂▂▂▂▂▃▃▃▃▃▃▃▃▃▂▂▂▂▂▂▂▂ |
| BIRKFELLNER W, 2000, IEEE T MED IMAGING, V19, P616, [DOI](http://dx.doi.org/10.1109%2F42.870668) | 2000 | 5.054 | **2001** | 2005 | ▂▂▂▂▂▂▂▂▂▂▂▃▃▃▃▃▂▂▂▂▂▂▂▂▂▂▂▂ |
| TAMURA H, 2001, IEEE COMPUT GRAPH, V21, P64, [DOI](http://dx.doi.org/10.1109%2F38.963462) | 2001 | 5.9698 | **2002** | 2009 | ▂▂▂▂▂▂▂▂▂▂▂▂▃▃▃▃▃▃▃▃▂▂▂▂▂▂▂▂ |
| LU CP, 2000, IEEE T PATTERN ANAL, V22, P610, [DOI](http://dx.doi.org/10.1109%2F34.862199) | 2000 | 11.8589 | **2002** | 2008 | ▂▂▂▂▂▂▂▂▂▂▂▂▃▃▃▃▃▃▃▂▂▂▂▂▂▂▂▂ |
| BLACKWELL M, 2000, MED IMAGE ANAL, V4, P67, [DOI](http://dx.doi.org/10.1016%2FS1361-8415%2800%2900007-4) | 2000 | 6.3434 | **2002** | 2006 | ▂▂▂▂▂▂▂▂▂▂▂▂▃▃▃▃▃▂▂▂▂▂▂▂▂▂▂▂ |
| BILLINGHURST M, 2001, COMPUT GRAPH-UK, V25, P745, [DOI](http://dx.doi.org/10.1016%2FS0097-8493%2801%2900117-0) | 2001 | 11.5882 | **2002** | 2009 | ▂▂▂▂▂▂▂▂▂▂▂▂▃▃▃▃▃▃▃▃▂▂▂▂▂▂▂▂ |
| BIMBER O, 2001, IEEE COMPUT GRAPH, V21, P48, [DOI](http://dx.doi.org/10.1109%2F38.963460) | 2001 | 4.5924 | **2002** | 2008 | ▂▂▂▂▂▂▂▂▂▂▂▂▃▃▃▃▃▃▃▂▂▂▂▂▂▂▂▂ |
| SATOH K, 2001, IEEE AND ACM INTERNATIONAL SYMPOSIUM ON AUGMENTED REALITY, V, P67, [DOI](http://dx.doi.org/10.1109%2FISAR.2001.970516) | 2001 | 7.9327 | **2002** | 2007 | ▂▂▂▂▂▂▂▂▂▂▂▂▃▃▃▃▃▃▂▂▂▂▂▂▂▂▂▂ |
| FERRARI V, 2001, IEEE AND ACM INTERNATIONAL SYMPOSIUM ON AUGMENTED REALITY, V, P87, [DOI](http://dx.doi.org/10.1109%2FISAR.2001.970518) | 2001 | 5.2223 | **2002** | 2009 | ▂▂▂▂▂▂▂▂▂▂▂▂▃▃▃▃▃▃▃▃▂▂▂▂▂▂▂▂ |
| HARTLEY R, 2000, MULTIPLE VIEW GEOMET, V, P | 2000 | 37.7054 | **2002** | 2008 | ▂▂▂▂▂▂▂▂▂▂▂▂▃▃▃▃▃▃▃▂▂▂▂▂▂▂▂▂ |
| VERRI A, 1998, INTRO TECHNIQUES 3D, V, P | 1998 | 4.5214 | **2002** | 2006 | ▂▂▂▂▂▂▂▂▂▂▂▂▃▃▃▃▃▂▂▂▂▂▂▂▂▂▂▂ |
| BILLINGHURST M, 2001, IEEE COMPUT GRAPH, V, P2 | 2001 | 4.5924 | **2002** | 2008 | ▂▂▂▂▂▂▂▂▂▂▂▂▃▃▃▃▃▃▃▂▂▂▂▂▂▂▂▂ |
| HOLLERER T, 2001, COMPUT GRAPH-UK, V25, P799, [DOI](http://dx.doi.org/10.1016%2FS0097-8493%2801%2900122-4) | 2001 | 4.1017 | **2002** | 2009 | ▂▂▂▂▂▂▂▂▂▂▂▂▃▃▃▃▃▃▃▃▂▂▂▂▂▂▂▂ |
| EDWARDS PJ, 2000, IEEE T MED IMAGING, V19, P1082, [DOI](http://dx.doi.org/10.1109%2F42.896784) | 2000 | 20.4265 | **2002** | 2008 | ▂▂▂▂▂▂▂▂▂▂▂▂▃▃▃▃▃▃▃▂▂▂▂▂▂▂▂▂ |
| ELLIS SR, 1998, HUM FACTORS, V40, P415, [DOI](http://dx.doi.org/10.1518%2F001872098779591278) | 1998 | 3.5158 | **2002** | 2006 | ▂▂▂▂▂▂▂▂▂▂▂▂▃▃▃▃▃▂▂▂▂▂▂▂▂▂▂▂ |
| AZUMA R, 2001, IEEE COMPUT GRAPH, V21, P34, [DOI](http://dx.doi.org/10.1109%2F38.963459) | 2001 | 84.2331 | **2002** | 2009 | ▂▂▂▂▂▂▂▂▂▂▂▂▃▃▃▃▃▃▃▃▂▂▂▂▂▂▂▂ |
| SAUER F, 2001, IEEE AND ACM INTERNATIONAL SYMPOSIUM ON AUGMENTED REALITY, V, P30, [DOI](http://dx.doi.org/10.1109%2FISAR.2001.970513) | 2001 | 7.0918 | **2002** | 2009 | ▂▂▂▂▂▂▂▂▂▂▂▂▃▃▃▃▃▃▃▃▂▂▂▂▂▂▂▂ |
| YOU SY, 2001, P IEEE VIRT REAL ANN, V, P71, [DOI](http://dx.doi.org/10.1109%2FVR.2001.913772) | 2001 | 12.279 | **2002** | 2008 | ▂▂▂▂▂▂▂▂▂▂▂▂▃▃▃▃▃▃▃▂▂▂▂▂▂▂▂▂ |
| WATZINGER F, 2001, PLAST RECONSTR SURG, V107, P659, [DOI](http://dx.doi.org/10.1097%2F00006534-200103000-00003) | 2001 | 3.7909 | **2002** | 2005 | ▂▂▂▂▂▂▂▂▂▂▂▂▃▃▃▃▂▂▂▂▂▂▂▂▂▂▂▂ |
| HUA H, 2000, APPL OPTICS, V39, P3814, [DOI](http://dx.doi.org/10.1364%2FAO.39.003814) | 2000 | 7.1027 | **2002** | 2008 | ▂▂▂▂▂▂▂▂▂▂▂▂▃▃▃▃▃▃▃▂▂▂▂▂▂▂▂▂ |
| PIEKARSKI W, 2001, FIFTH INTERNATIONAL SYMPOSIUM ON WEARABLE COMPUTERS, V, P31, [DOI](http://dx.doi.org/10.1109%2FISWC.2001.962093) | 2001 | 6.7177 | **2002** | 2009 | ▂▂▂▂▂▂▂▂▂▂▂▂▃▃▃▃▃▃▃▃▂▂▂▂▂▂▂▂ |
| BILLAUD C, 2001, SCI ALIMENT, V21, P3, [DOI](http://dx.doi.org/10.3166%2FSDA.21.3-26) | 2001 | 4.6554 | **2002** | 2007 | ▂▂▂▂▂▂▂▂▂▂▂▂▃▃▃▃▃▃▂▂▂▂▂▂▂▂▂▂ |
| CHAI L, 2002, PRESENCE-TELEOP VIRT, V11, P474, [DOI](http://dx.doi.org/10.1162%2F105474602320935829) | 2002 | 6.7032 | **2003** | 2010 | ▂▂▂▂▂▂▂▂▂▂▂▂▂▃▃▃▃▃▃▃▃▂▂▂▂▂▂▂ |
| GENC Y, 2002, INTERNATIONAL SYMPOSIUM ON MIXED AND AUGMENTED REALITY, V, P295, [DOI](http://dx.doi.org/10.1109%2FISMAR.2002.1115122) | 2002 | 10.6863 | **2003** | 2009 | ▂▂▂▂▂▂▂▂▂▂▂▂▂▃▃▃▃▃▃▃▂▂▂▂▂▂▂▂ |
| SCHARSTEIN D, 2002, INT J COMPUT VISION, V47, P7, [DOI](http://dx.doi.org/10.1023%2FA%3A1014573219977) | 2002 | 4.4654 | **2003** | 2010 | ▂▂▂▂▂▂▂▂▂▂▂▂▂▃▃▃▃▃▃▃▃▂▂▂▂▂▂▂ |
| FEINER SK, 2002, SCI AM, V286, P48, [DOI](http://dx.doi.org/10.1038%2FSCIENTIFICAMERICAN0402-48) | 2002 | 5.5839 | **2003** | 2010 | ▂▂▂▂▂▂▂▂▂▂▂▂▂▃▃▃▃▃▃▃▃▂▂▂▂▂▂▂ |
| APPEL M, 2002, MACH VISION APPL, V13, P111, [DOI](http://dx.doi.org/10.1007%2FS001380100066) | 2002 | 4.7039 | **2003** | 2006 | ▂▂▂▂▂▂▂▂▂▂▂▂▂▃▃▃▃▂▂▂▂▂▂▂▂▂▂▂ |
| SITTI M, 1998, 1998 IEEE/RSJ INTERNATIONAL CONFERENCE ON INTELLIGENT ROBOTS AND SYSTEMS - PROCEEDINGS, VOLS 1-3, P1739, [DOI](http://dx.doi.org/10.1109%2FIROS.1998.724849) | 1998 | 6.9512 | **2003** | 2005 | ▂▂▂▂▂▂▂▂▂▂▂▂▂▃▃▃▂▂▂▂▂▂▂▂▂▂▂▂ |
| BILLINGHURST M, 2002, COMMUN ACM, V45, P64 | 2002 | 10.4402 | **2003** | 2010 | ▂▂▂▂▂▂▂▂▂▂▂▂▂▃▃▃▃▃▃▃▃▂▂▂▂▂▂▂ |
| NEWMAN J, 2001, IEEE AND ACM INTERNATIONAL SYMPOSIUM ON AUGMENTED REALITY, V, P77, [DOI](http://dx.doi.org/10.1109%2FISAR.2001.970517) | 2001 | 5.9145 | **2003** | 2008 | ▂▂▂▂▂▂▂▂▂▂▂▂▂▃▃▃▃▃▃▂▂▂▂▂▂▂▂▂ |
| GUTHOLD M, 2000, IEEE-ASME T MECH, V5, P189, [DOI](http://dx.doi.org/10.1109%2F3516.847092) | 2000 | 7.4174 | **2003** | 2005 | ▂▂▂▂▂▂▂▂▂▂▂▂▂▃▃▃▂▂▂▂▂▂▂▂▂▂▂▂ |
| LI G Y, 2003, P IEEE INT C NAN SAN, V, P | 2003 | 4.0523 | **2003** | 2005 | ▂▂▂▂▂▂▂▂▂▂▂▂▂▃▃▃▂▂▂▂▂▂▂▂▂▂▂▂ |
| BIRKFELLNER W, 2002, IEEE T MED IMAGING, V21, P991, [DOI](http://dx.doi.org/10.1109%2FTMI.2002.803099) | 2002 | 13.4365 | **2003** | 2010 | ▂▂▂▂▂▂▂▂▂▂▂▂▂▃▃▃▃▃▃▃▃▂▂▂▂▂▂▂ |
| RIBO M, 2002, IEEE COMPUT GRAPH, V22, P54, [DOI](http://dx.doi.org/10.1109%2FMCG.2002.1046629) | 2002 | 8.4124 | **2003** | 2008 | ▂▂▂▂▂▂▂▂▂▂▂▂▂▃▃▃▃▃▃▂▂▂▂▂▂▂▂▂ |
| POUPYREV I, 2002, COMPUTER, V35, P44, [DOI](http://dx.doi.org/10.1109%2F2.989929) | 2002 | 8.1739 | **2003** | 2008 | ▂▂▂▂▂▂▂▂▂▂▂▂▂▃▃▃▃▃▃▂▂▂▂▂▂▂▂▂ |
| SHAHIDI R, 2002, IEEE T MED IMAGING, V21, P1524, [DOI](http://dx.doi.org/10.1109%2FTMI.2002.806597) | 2002 | 6.33 | **2003** | 2010 | ▂▂▂▂▂▂▂▂▂▂▂▂▂▃▃▃▃▃▃▃▃▂▂▂▂▂▂▂ |
| FURMANSKI C, 2002, INTERNATIONAL SYMPOSIUM ON MIXED AND AUGMENTED REALITY, V, P215, [DOI](http://dx.doi.org/10.1109%2FISMAR.2002.1115091) | 2002 | 4.5126 | **2003** | 2009 | ▂▂▂▂▂▂▂▂▂▂▂▂▂▃▃▃▃▃▃▃▂▂▂▂▂▂▂▂ |
| VOGT S, 2002, INTERNATIONAL SYMPOSIUM ON MIXED AND AUGMENTED REALITY, V, P127, [DOI](http://dx.doi.org/10.1109%2FISMAR.2002.1115082) | 2002 | 5.7455 | **2003** | 2009 | ▂▂▂▂▂▂▂▂▂▂▂▂▂▃▃▃▃▃▃▃▂▂▂▂▂▂▂▂ |
| KAWAMATA T, 2002, NEUROSURGERY, V50, P1393, [DOI](http://dx.doi.org/10.1097%2F00006123-200206000-00038) | 2002 | 6.33 | **2003** | 2010 | ▂▂▂▂▂▂▂▂▂▂▂▂▂▃▃▃▃▃▃▃▃▂▂▂▂▂▂▂ |
| ZHANG ZY, 2000, IEEE T PATTERN ANAL, V22, P1330, [DOI](http://dx.doi.org/10.1109%2F34.888718) | 2000 | 18.7031 | **2003** | 2008 | ▂▂▂▂▂▂▂▂▂▂▂▂▂▃▃▃▃▃▃▂▂▂▂▂▂▂▂▂ |
| MANN S, 2002, IEEE COMPUT, V30, P25 | 2002 | 3.4729 | **2003** | 2005 | ▂▂▂▂▂▂▂▂▂▂▂▂▂▃▃▃▂▂▂▂▂▂▂▂▂▂▂▂ |
| HANSEN LT, 1998, NANOTECHNOLOGY, V9, P337, [DOI](http://dx.doi.org/10.1088%2F0957-4484%2F9%2F4%2F006) | 1998 | 6.9512 | **2003** | 2005 | ▂▂▂▂▂▂▂▂▂▂▂▂▂▃▃▃▂▂▂▂▂▂▂▂▂▂▂▂ |
| REQUICHA AAG, 1998, IEEE INT CONF ROBOT, V, P3368, [DOI](http://dx.doi.org/10.1109%2FROBOT.1998.680958) | 1998 | 5.7912 | **2003** | 2005 | ▂▂▂▂▂▂▂▂▂▂▂▂▂▃▃▃▂▂▂▂▂▂▂▂▂▂▂▂ |
| AZUMA RT, 1997, TELEOPERATORS VIRTUA, V6, P355 | 1997 | 4.9341 | **2004** | 2005 | ▂▂▂▂▂▂▂▂▂▂▂▂▂▂▃▃▂▂▂▂▂▂▂▂▂▂▂▂ |
| REGENBRECHT H T, 2002, VIRTUAL REALITY, V6, P151, [DOI](http://dx.doi.org/10.1007%2FS100550200016) | 2002 | 6.275 | **2004** | 2009 | ▂▂▂▂▂▂▂▂▂▂▂▂▂▂▃▃▃▃▃▃▂▂▂▂▂▂▂▂ |
| PRINCE SJD, 2002, IEEE COMPUT GRAPH, V22, P39, [DOI](http://dx.doi.org/10.1109%2FMCG.2002.1046627) | 2002 | 6.2054 | **2004** | 2008 | ▂▂▂▂▂▂▂▂▂▂▂▂▂▂▃▃▃▃▃▂▂▂▂▂▂▂▂▂ |
| STETTEN GD, 2001, J ULTRAS MED, V20, P235 | 2001 | 7.4862 | **2004** | 2008 | ▂▂▂▂▂▂▂▂▂▂▂▂▂▂▃▃▃▃▃▂▂▂▂▂▂▂▂▂ |
| SIMON G, 2002, INTERNATIONAL SYMPOSIUM ON MIXED AND AUGMENTED REALITY, V, P285, [DOI](http://dx.doi.org/10.1109%2FISMAR.2002.1115118) | 2002 | 8.3347 | **2004** | 2010 | ▂▂▂▂▂▂▂▂▂▂▂▂▂▂▃▃▃▃▃▃▃▂▂▂▂▂▂▂ |
| LEPETIT V, 2003, SECOND IEEE AND ACM INTERNATIONAL SYMPOSIUM ON MIXED AND AUGMENTED REALITY, V, P93, [DOI](http://dx.doi.org/10.1109%2FISMAR.2003.1240692) | 2003 | 5.3728 | **2004** | 2011 | ▂▂▂▂▂▂▂▂▂▂▂▂▂▂▃▃▃▃▃▃▃▃▂▂▂▂▂▂ |
| JIANG BL, 2004, P IEEE VIRT REAL ANN, V, P3, [DOI](http://dx.doi.org/10.1109%2FVR.2004.1310049) | 2004 | 7.3357 | **2004** | 2009 | ▂▂▂▂▂▂▂▂▂▂▂▂▂▂▃▃▃▃▃▃▂▂▂▂▂▂▂▂ |
| PIEKARSKI W, 2002, COMMUN ACM, V45, P36 | 2002 | 6.5622 | **2004** | 2010 | ▂▂▂▂▂▂▂▂▂▂▂▂▂▂▃▃▃▃▃▃▃▂▂▂▂▂▂▂ |
| NAIMARK L, 2002, INTERNATIONAL SYMPOSIUM ON MIXED AND AUGMENTED REALITY, V, P27, [DOI](http://dx.doi.org/10.1109%2FISMAR.2002.1115065) | 2002 | 8.0307 | **2004** | 2009 | ▂▂▂▂▂▂▂▂▂▂▂▂▂▂▃▃▃▃▃▃▂▂▂▂▂▂▂▂ |
| AZUMA R, 2003, SECOND IEEE AND ACM INTERNATIONAL SYMPOSIUM ON MIXED AND AUGMENTED REALITY, V, P66, [DOI](http://dx.doi.org/10.1109%2FISMAR.2003.1240689) | 2003 | 7.3843 | **2004** | 2010 | ▂▂▂▂▂▂▂▂▂▂▂▂▂▂▃▃▃▃▃▃▃▂▂▂▂▂▂▂ |
| COMPORT AI, 2003, SECOND IEEE AND ACM INTERNATIONAL SYMPOSIUM ON MIXED AND AUGMENTED REALITY, V, P36, [DOI](http://dx.doi.org/10.1109%2FISMAR.2003.1240686) | 2003 | 7.9232 | **2004** | 2010 | ▂▂▂▂▂▂▂▂▂▂▂▂▂▂▃▃▃▃▃▃▃▂▂▂▂▂▂▂ |
| KLEIN G, 2003, PROCEEDINGS SECOND IEEE AND ACM INTERNATIONAL SYMPOSIUM ON MIXED AND AUGMENTED REALITY, V, P113 | 2003 | 5.046 | **2004** | 2010 | ▂▂▂▂▂▂▂▂▂▂▂▂▂▂▃▃▃▃▃▃▃▂▂▂▂▂▂▂ |
| LIVINGSTON MA, 2003, SECOND IEEE AND ACM INTERNATIONAL SYMPOSIUM ON MIXED AND AUGMENTED REALITY, V, P56, [DOI](http://dx.doi.org/10.1109%2FISMAR.2003.1240688) | 2003 | 5.5805 | **2004** | 2009 | ▂▂▂▂▂▂▂▂▂▂▂▂▂▂▃▃▃▃▃▃▂▂▂▂▂▂▂▂ |
| ISHII H, 2002, INTERNATIONAL SYMPOSIUM ON MIXED AND AUGMENTED REALITY, V, P203, [DOI](http://dx.doi.org/10.1109%2FISMAR.2002.1115090) | 2002 | 4.9191 | **2004** | 2010 | ▂▂▂▂▂▂▂▂▂▂▂▂▂▂▃▃▃▃▃▃▃▂▂▂▂▂▂▂ |
| THOMAS B, 2002, PERS UBIQUIT COMPUT, V6, P75, [DOI](http://dx.doi.org/10.1007%2FS007790200007) | 2002 | 5.4568 | **2004** | 2010 | ▂▂▂▂▂▂▂▂▂▂▂▂▂▂▃▃▃▃▃▃▃▂▂▂▂▂▂▂ |
| WAGNER D, 2003, P 7 IEEE INT S WEAR, V, P127 | 2003 | 7.887 | **2004** | 2011 | ▂▂▂▂▂▂▂▂▂▂▂▂▂▂▃▃▃▃▃▃▃▃▂▂▂▂▂▂ |
| SIMON G, 2002, IEEE COMPUT GRAPH, V22, P46, [DOI](http://dx.doi.org/10.1109%2FMCG.2002.1046628) | 2002 | 7.1732 | **2004** | 2009 | ▂▂▂▂▂▂▂▂▂▂▂▂▂▂▃▃▃▃▃▃▂▂▂▂▂▂▂▂ |
| ROSENTHAL M, 2002, MED IMAGE ANAL, V6, P313, [DOI](http://dx.doi.org/10.1016%2FS1361-8415%2802%2900088-9) | 2002 | 13.9762 | **2004** | 2010 | ▂▂▂▂▂▂▂▂▂▂▂▂▂▂▃▃▃▃▃▃▃▂▂▂▂▂▂▂ |
| HARTLEY R, 2001, MULTIPLE VIEW GEOMET, V, P | 2001 | 3.583 | **2004** | 2009 | ▂▂▂▂▂▂▂▂▂▂▂▂▂▂▃▃▃▃▃▃▂▂▂▂▂▂▂▂ |
| TENMOKU R, 2003, P IEEE INT S WEAR CO, V, P110 | 2003 | 5.0175 | **2004** | 2008 | ▂▂▂▂▂▂▂▂▂▂▂▂▂▂▃▃▃▃▃▂▂▂▂▂▂▂▂▂ |
| CHEOK AD, 2002, PERS UBIQUIT COMPUT, V6, P430, [DOI](http://dx.doi.org/10.1007%2FS007790200047) | 2002 | 3.688 | **2004** | 2010 | ▂▂▂▂▂▂▂▂▂▂▂▂▂▂▃▃▃▃▃▃▃▂▂▂▂▂▂▂ |
| WELCH G, 2002, IEEE COMPUT GRAPH, V22, P24, [DOI](http://dx.doi.org/10.1109%2FMCG.2002.1046626) | 2002 | 9.9815 | **2004** | 2010 | ▂▂▂▂▂▂▂▂▂▂▂▂▂▂▃▃▃▃▃▃▃▂▂▂▂▂▂▂ |
| TANG A, 2003, P SIGCHI C HUM FACT, V, , [DOI](http://dx.doi.org/10.1145%2F642611.642626%5D) | 2003 | 8.8628 | **2005** | 2011 | ▂▂▂▂▂▂▂▂▂▂▂▂▂▂▂▃▃▃▃▃▃▃▂▂▂▂▂▂ |
| BILLINGHURST M, 2001, IEEE COMPUT GRAPH, V21, P6, [DOI](http://dx.doi.org/10.1109%2F38.920621) | 2001 | 12.4511 | **2005** | 2009 | ▂▂▂▂▂▂▂▂▂▂▂▂▂▂▂▃▃▃▃▃▂▂▂▂▂▂▂▂ |
| DRUMMOND T, 2002, IEEE T PATTERN ANAL, V24, P932, [DOI](http://dx.doi.org/10.1109%2FTPAMI.2002.1017620) | 2002 | 7.1961 | **2005** | 2010 | ▂▂▂▂▂▂▂▂▂▂▂▂▂▂▂▃▃▃▃▃▃▂▂▂▂▂▂▂ |
| HENRYSSON A, 2004, P 3 INT C MOB UB MUL, V, P41 | 2004 | 3.8356 | **2005** | 2012 | ▂▂▂▂▂▂▂▂▂▂▂▂▂▂▂▃▃▃▃▃▃▃▃▂▂▂▂▂ |
| VLAHAKIS V, 2002, IEEE COMPUT GRAPH, V22, P52, [DOI](http://dx.doi.org/10.1109%2FMCG.2002.1028726) | 2002 | 7.4029 | **2005** | 2010 | ▂▂▂▂▂▂▂▂▂▂▂▂▂▂▂▃▃▃▃▃▃▂▂▂▂▂▂▂ |
| LEE GA, 2004, P 3 IEEE ACM INT S M, V, P172 | 2004 | 5.1701 | **2005** | 2011 | ▂▂▂▂▂▂▂▂▂▂▂▂▂▂▂▃▃▃▃▃▃▃▂▂▂▂▂▂ |
| HEDLEY NR, 2002, PRESENCE-TELEOP VIRT, V11, P119, [DOI](http://dx.doi.org/10.1162%2F105474602317396002) | 2002 | 4.0442 | **2005** | 2010 | ▂▂▂▂▂▂▂▂▂▂▂▂▂▂▂▃▃▃▃▃▃▂▂▂▂▂▂▂ |
| VACCHETTI L, 2004, P 3 IEEE ACM INT S M, V, P48 | 2004 | 9.6617 | **2005** | 2011 | ▂▂▂▂▂▂▂▂▂▂▂▂▂▂▂▃▃▃▃▃▃▃▂▂▂▂▂▂ |
| SCHMALSTIEG D, 2002, PRESENCE-TELEOP VIRT, V11, P33, [DOI](http://dx.doi.org/10.1162%2F105474602317343640) | 2002 | 12.125 | **2005** | 2010 | ▂▂▂▂▂▂▂▂▂▂▂▂▂▂▂▃▃▃▃▃▃▂▂▂▂▂▂▂ |
| BOWMAN D A, 2004, 3D USER INTERFACES T, V, P | 2004 | 8.3821 | **2005** | 2012 | ▂▂▂▂▂▂▂▂▂▂▂▂▂▂▂▃▃▃▃▃▃▃▃▂▂▂▂▂ |
| KAISER E, 2003, P 5 INT C MULT INT I, V, P12 | 2003 | 4.7718 | **2005** | 2011 | ▂▂▂▂▂▂▂▂▂▂▂▂▂▂▂▃▃▃▃▃▃▃▂▂▂▂▂▂ |
| CLIFFORD MARK A, 2002, COMPUT AIDED SURG, V7, P291, [DOI](http://dx.doi.org/10.1002%2FIGS.10049) | 2002 | 3.8996 | **2005** | 2009 | ▂▂▂▂▂▂▂▂▂▂▂▂▂▂▂▃▃▃▃▃▂▂▂▂▂▂▂▂ |
| NISTER D, 2004, IEEE T PATTERN ANAL, V26, P756, [DOI](http://dx.doi.org/10.1109%2FTPAMI.2004.17) | 2004 | 6.7644 | **2005** | 2011 | ▂▂▂▂▂▂▂▂▂▂▂▂▂▂▂▃▃▃▃▃▃▃▂▂▂▂▂▂ |
| VACCHETTI L, 2004, IEEE T PATTERN ANAL, V26, P1385, [DOI](http://dx.doi.org/10.1109%2FTPAMI.2004.92) | 2004 | 9.1587 | **2005** | 2011 | ▂▂▂▂▂▂▂▂▂▂▂▂▂▂▂▃▃▃▃▃▃▃▂▂▂▂▂▂ |
| BUCHMANN V, 2004, P 2 INT C COMP GRAPH, V, P212 | 2004 | 5.3565 | **2005** | 2010 | ▂▂▂▂▂▂▂▂▂▂▂▂▂▂▂▃▃▃▃▃▃▂▂▂▂▂▂▂ |
| ABAWI D F, 2004, P 3 IEEE ACM INT S M, V, P260 | 2004 | 5.1701 | **2005** | 2011 | ▂▂▂▂▂▂▂▂▂▂▂▂▂▂▂▃▃▃▃▃▃▃▂▂▂▂▂▂ |
| LI GY, 2004, IEEE-ASME T MECH, V9, P358, [DOI](http://dx.doi.org/10.1109%2FTMECH.2004.828651) | 2004 | 4.5341 | **2005** | 2012 | ▂▂▂▂▂▂▂▂▂▂▂▂▂▂▂▃▃▃▃▃▃▃▃▂▂▂▂▂ |
| HARTLEY R, 2003, MULTIPLE VIEW GEOMET, V, P | 2003 | 16.0676 | **2005** | 2011 | ▂▂▂▂▂▂▂▂▂▂▂▂▂▂▂▃▃▃▃▃▃▃▂▂▂▂▂▂ |
| CHEOK AD, 2003, P 2 WORKSH NETW SYST, V, P106 | 2003 | 3.9153 | **2006** | 2011 | ▂▂▂▂▂▂▂▂▂▂▂▂▂▂▂▂▃▃▃▃▃▃▂▂▂▂▂▂ |
| FEUERSTEIN M, 2005, LECT NOTES COMPUT SC, V3750, P287 | 2005 | 4.7254 | **2006** | 2009 | ▂▂▂▂▂▂▂▂▂▂▂▂▂▂▂▂▃▃▃▃▂▂▂▂▂▂▂▂ |
| HENRYSSON A, 2005, INTERNATIONAL SYMPOSIUM ON MIXED AND AUGMENTED REALITY, V, P80 | 2005 | 8.1162 | **2006** | 2011 | ▂▂▂▂▂▂▂▂▂▂▂▂▂▂▂▂▃▃▃▃▃▃▂▂▂▂▂▂ |
| WACKER FK, 2006, RADIOLOGY, V238, P497, [DOI](http://dx.doi.org/10.1148%2FRADIOL.2382041441) | 2006 | 7.421 | **2006** | 2013 | ▂▂▂▂▂▂▂▂▂▂▂▂▂▂▂▂▃▃▃▃▃▃▃▃▂▂▂▂ |
| FICHTINGER G, 2005, IEEE T BIO-MED ENG, V52, P1415, [DOI](http://dx.doi.org/10.1109%2FTBME.2005.851493) | 2005 | 9.0398 | **2006** | 2013 | ▂▂▂▂▂▂▂▂▂▂▂▂▂▂▂▂▃▃▃▃▃▃▃▃▂▂▂▂ |
| FALK V, 2005, ANN THORAC SURG, V79, P2040, [DOI](http://dx.doi.org/10.1016%2FJ%2FATHORASCUR.2004.11.060) | 2005 | 5.4319 | **2006** | 2010 | ▂▂▂▂▂▂▂▂▂▂▂▂▂▂▂▂▃▃▃▃▃▂▂▂▂▂▂▂ |
| NAVAB N, 2004, IEEE COMPUT GRAPH, V24, P16, [DOI](http://dx.doi.org/10.1109%2FMCG.2004.1297006) | 2004 | 5.2223 | **2006** | 2011 | ▂▂▂▂▂▂▂▂▂▂▂▂▂▂▂▂▃▃▃▃▃▃▂▂▂▂▂▂ |
| HALLAWAY D, 2004, APPL ARTIF INTELL, V18, P477, [DOI](http://dx.doi.org/10.1080%2F08839510490462768) | 2004 | 3.4798 | **2006** | 2011 | ▂▂▂▂▂▂▂▂▂▂▂▂▂▂▂▂▃▃▃▃▃▃▂▂▂▂▂▂ |
| YUAN ML, 2005, IEEE T VIS COMPUT GR, V11, P254, [DOI](http://dx.doi.org/10.1109%2FTVCG.2005.48) | 2005 | 5.3571 | **2006** | 2010 | ▂▂▂▂▂▂▂▂▂▂▂▂▂▂▂▂▃▃▃▃▃▂▂▂▂▂▂▂ |
| PUPILLI M, 2005, P BRIT MACH VIS C, V, P519 | 2005 | 4.9639 | **2006** | 2011 | ▂▂▂▂▂▂▂▂▂▂▂▂▂▂▂▂▃▃▃▃▃▃▂▂▂▂▂▂ |
| WUEST H, 2005, INTERNATIONAL SYMPOSIUM ON MIXED AND AUGMENTED REALITY, V, P62 | 2005 | 5.7134 | **2006** | 2010 | ▂▂▂▂▂▂▂▂▂▂▂▂▂▂▂▂▃▃▃▃▃▂▂▂▂▂▂▂ |
| BURDEA GC, 2003, VIRTUAL REALITY TECH, V, P | 2003 | 5.2192 | **2007** | 2011 | ▂▂▂▂▂▂▂▂▂▂▂▂▂▂▂▂▂▃▃▃▃▃▂▂▂▂▂▂ |
| PAN ZG, 2006, COMPUT GRAPH-UK, V30, P20, [DOI](http://dx.doi.org/10.1016%2FJ.CAG.2005.10.004) | 2006 | 5.5344 | **2007** | 2014 | ▂▂▂▂▂▂▂▂▂▂▂▂▂▂▂▂▂▃▃▃▃▃▃▃▃▂▂▂ |
| JUAN MC, 2005, IEEE COMPUT GRAPH, V25, P31, [DOI](http://dx.doi.org/10.1109%2FMCG.2005.143) | 2005 | 7.2688 | **2007** | 2013 | ▂▂▂▂▂▂▂▂▂▂▂▂▂▂▂▂▂▃▃▃▃▃▃▃▂▂▂▂ |
| KAUFMANN H, 2003, COMPUT GRAPH-UK, V27, P339, [DOI](http://dx.doi.org/10.1016%2FS0097-8493%2803%2900028-1) | 2003 | 11.6642 | **2007** | 2011 | ▂▂▂▂▂▂▂▂▂▂▂▂▂▂▂▂▂▃▃▃▃▃▂▂▂▂▂▂ |
| COMPORT AI, 2006, IEEE T VIS COMPUT GR, V12, P615, [DOI](http://dx.doi.org/10.1109%2FTVCG.2006.78) | 2006 | 11.4615 | **2007** | 2014 | ▂▂▂▂▂▂▂▂▂▂▂▂▂▂▂▂▂▃▃▃▃▃▃▃▃▂▂▂ |
| DOUCET A, 2001, SEQUENTIAL MONTE CAR, V, P | 2001 | 3.9495 | **2007** | 2009 | ▂▂▂▂▂▂▂▂▂▂▂▂▂▂▂▂▂▃▃▃▂▂▂▂▂▂▂▂ |
| YAMAGUCHI TETSUZO, 2004, COMPUT AIDED SURG, V9, P203, [DOI](http://dx.doi.org/10.1080%2F10929080500163505) | 2004 | 4.6811 | **2007** | 2012 | ▂▂▂▂▂▂▂▂▂▂▂▂▂▂▂▂▂▃▃▃▃▃▃▂▂▂▂▂ |
| ONG S K, 2004, VIRTUAL AUGMENTED RE, V, P | 2004 | 5.96 | **2007** | 2012 | ▂▂▂▂▂▂▂▂▂▂▂▂▂▂▂▂▂▃▃▃▃▃▃▂▂▂▂▂ |
| STOYANOV D, 2005, LECT NOTES COMPUT SC, V3750, P139 | 2005 | 4.7007 | **2007** | 2013 | ▂▂▂▂▂▂▂▂▂▂▂▂▂▂▂▂▂▃▃▃▃▃▃▃▂▂▂▂ |
| FOXLIN E, 2003, P IEEE VIRT REAL ANN, V, P199, [DOI](http://dx.doi.org/10.1109%2FVR.2003.1191139) | 2003 | 4.3059 | **2007** | 2011 | ▂▂▂▂▂▂▂▂▂▂▂▂▂▂▂▂▂▃▃▃▃▃▂▂▂▂▂▂ |
| PANG Y, 2006, ASSEMBLY AUTOM, V26, P34, [DOI](http://dx.doi.org/10.1108%2F01445150610645648) | 2006 | 4.7612 | **2007** | 2010 | ▂▂▂▂▂▂▂▂▂▂▂▂▂▂▂▂▂▃▃▃▃▂▂▂▂▂▂▂ |
| CHEOK AD, 2004, PERS UBIQUIT COMPUT, V8, P71, [DOI](http://dx.doi.org/10.1007%2FS00779-004-0267-X) | 2004 | 5.2656 | **2007** | 2012 | ▂▂▂▂▂▂▂▂▂▂▂▂▂▂▂▂▂▃▃▃▃▃▃▂▂▂▂▂ |
| MARESCAUX J, 2004, JAMA-J AM MED ASSOC, V292, P2214, [DOI](http://dx.doi.org/10.1001%2FJAMA.292.18.2214-C) | 2004 | 16.2819 | **2007** | 2012 | ▂▂▂▂▂▂▂▂▂▂▂▂▂▂▂▂▂▃▃▃▃▃▃▂▂▂▂▂ |
| SHUHAIBER JH, 2004, ARCH SURG-CHICAGO, V139, P170, [DOI](http://dx.doi.org/10.1001%2FARCHSURG.139.2.170) | 2004 | 8.8395 | **2007** | 2012 | ▂▂▂▂▂▂▂▂▂▂▂▂▂▂▂▂▂▃▃▃▃▃▃▂▂▂▂▂ |
| REITMAYR G, 2006, ISMAR, V,, P | 2006 | 5.2424 | **2007** | 2014 | ▂▂▂▂▂▂▂▂▂▂▂▂▂▂▂▂▂▃▃▃▃▃▃▃▃▂▂▂ |
| HOLLERER T H, 2004, TELEGEOINFORMATICS L, V, P | 2004 | 4.6811 | **2007** | 2012 | ▂▂▂▂▂▂▂▂▂▂▂▂▂▂▂▂▂▃▃▃▃▃▃▂▂▂▂▂ |
| YUAN ML, 2006, IEEE T VIS COMPUT GR, V12, P569, [DOI](http://dx.doi.org/10.1109%2FTVCG.2006.79) | 2006 | 9.1399 | **2007** | 2011 | ▂▂▂▂▂▂▂▂▂▂▂▂▂▂▂▂▂▃▃▃▃▃▂▂▂▂▂▂ |
| HARTLEY RI, 2004, MULTIPLE VIEW GEOMET, V, P | 2004 | 19.8827 | **2007** | 2012 | ▂▂▂▂▂▂▂▂▂▂▂▂▂▂▂▂▂▃▃▃▃▃▃▂▂▂▂▂ |
| BARAKONYI I, 2004, PROC GRAPH INTERF, V, P89 | 2004 | 4.8393 | **2007** | 2012 | ▂▂▂▂▂▂▂▂▂▂▂▂▂▂▂▂▂▃▃▃▃▃▃▂▂▂▂▂ |
| LEVEN J, 2005, LECT NOTES COMPUT SC, V3749, P811 | 2005 | 5.0629 | **2007** | 2013 | ▂▂▂▂▂▂▂▂▂▂▂▂▂▂▂▂▂▃▃▃▃▃▃▃▂▂▂▂ |
| REITMAYR G, 2006, P 5 IEEE ACM INT S M, V,, P | 2006 | 9.7459 | **2007** | 2012 | ▂▂▂▂▂▂▂▂▂▂▂▂▂▂▂▂▂▃▃▃▃▃▃▂▂▂▂▂ |
| LEPETIT V, 2006, IEEE T PATTERN ANAL, V28, P1465, [DOI](http://dx.doi.org/10.1109%2FTPAMI.2006.188) | 2006 | 12.5109 | **2008** | 2013 | ▂▂▂▂▂▂▂▂▂▂▂▂▂▂▂▂▂▂▃▃▃▃▃▃▂▂▂▂ |
| FJELD M, 2007, CONFERENCE ON HUMAN FACTORS IN COMPUTING SYSTEMS, VOLS 1 AND 2, P805 | 2007 | 4.2638 | **2008** | 2014 | ▂▂▂▂▂▂▂▂▂▂▂▂▂▂▂▂▂▂▃▃▃▃▃▃▃▂▂▂ |
| ROSTEN E, 2006, P EUR C COMP VIS, V,, P | 2006 | 7.8235 | **2008** | 2011 | ▂▂▂▂▂▂▂▂▂▂▂▂▂▂▂▂▂▂▃▃▃▃▂▂▂▂▂▂ |
| WIERZBICKI M, 2004, MED IMAGE ANAL, V8, P387, [DOI](http://dx.doi.org/10.1016%2FJ.MEDIA.2004.06.014) | 2004 | 4.5043 | **2008** | 2010 | ▂▂▂▂▂▂▂▂▂▂▂▂▂▂▂▂▂▂▃▃▃▂▂▂▂▂▂▂ |
| FEUERSTEIN M, 2008, IEEE T MED IMAGING, V27, P355, [DOI](http://dx.doi.org/10.1109%2FTMI.2007.907327) | 2008 | 7.1489 | **2008** | 2014 | ▂▂▂▂▂▂▂▂▂▂▂▂▂▂▂▂▂▂▃▃▃▃▃▃▃▂▂▂ |
| OHLENBURG JAN, 2004, J VIRTUAL REALITY BR, V1, P1 | 2004 | 3.5778 | **2008** | 2011 | ▂▂▂▂▂▂▂▂▂▂▂▂▂▂▂▂▂▂▃▃▃▃▂▂▂▂▂▂ |
| FIGL M, 2005, IEEE T MED IMAGING, V24, P1492, [DOI](http://dx.doi.org/10.1109%2FTMI.2005.856746) | 2005 | 4.931 | **2008** | 2011 | ▂▂▂▂▂▂▂▂▂▂▂▂▂▂▂▂▂▂▃▃▃▃▂▂▂▂▂▂ |
| LOWE DG, 2004, INT J COMPUT VISION, V60, P91, [DOI](http://dx.doi.org/10.1023%2FB%3AVISI.0000029664.99615.94) | 2004 | 64.0712 | **2008** | 2012 | ▂▂▂▂▂▂▂▂▂▂▂▂▂▂▂▂▂▂▃▃▃▃▃▂▂▂▂▂ |
| BIMBER O, 2005, SPATIAL AUGMENTED RE, V, P | 2005 | 27.9489 | **2008** | 2013 | ▂▂▂▂▂▂▂▂▂▂▂▂▂▂▂▂▂▂▃▃▃▃▃▃▂▂▂▂ |
| REGENBRECHT H, 2005, IEEE COMPUT GRAPH, V25, P48, [DOI](http://dx.doi.org/10.1109%2FMCG.2005.124) | 2005 | 7.2823 | **2008** | 2013 | ▂▂▂▂▂▂▂▂▂▂▂▂▂▂▂▂▂▂▃▃▃▃▃▃▂▂▂▂ |
| MOHRING M, 2004, P 3 IEEE ACM INT S M, V, P252 | 2004 | 4.856 | **2008** | 2012 | ▂▂▂▂▂▂▂▂▂▂▂▂▂▂▂▂▂▂▃▃▃▃▃▂▂▂▂▂ |
| KLEIN G, 2007, IEEE ACM INT S MIX A, V,, P | 2007 | 4.8467 | **2008** | 2014 | ▂▂▂▂▂▂▂▂▂▂▂▂▂▂▂▂▂▂▃▃▃▃▃▃▃▂▂▂ |
| CAKMAKCI O, 2006, J DISP TECHNOL, V2, P199, [DOI](http://dx.doi.org/10.1109%2FJDT.2006.879846) | 2006 | 7.553 | **2008** | 2014 | ▂▂▂▂▂▂▂▂▂▂▂▂▂▂▂▂▂▂▃▃▃▃▃▃▃▂▂▂ |
| WANG Y, 2003, GRAPH MODELS, V65, P185, [DOI](http://dx.doi.org/10.1016%2FS1524-0703%2803%2900043-2) | 2003 | 3.9407 | **2008** | 2010 | ▂▂▂▂▂▂▂▂▂▂▂▂▂▂▂▂▂▂▃▃▃▂▂▂▂▂▂▂ |
| SCHWEIGHOFER G, 2006, IEEE T PATTERN ANAL, V28, P2024, [DOI](http://dx.doi.org/10.1109%2FTPAMI.2006.252) | 2006 | 7.87 | **2008** | 2012 | ▂▂▂▂▂▂▂▂▂▂▂▂▂▂▂▂▂▂▃▃▃▃▃▂▂▂▂▂ |
| LEPETIT VINCENT, 2005, FOUNDATIONS AND TRENDS IN COMPUTER GRAPHICS AND VISION, V1, P1, [DOI](http://dx.doi.org/10.1561%2F0600000001) | 2005 | 9.2824 | **2008** | 2013 | ▂▂▂▂▂▂▂▂▂▂▂▂▂▂▂▂▂▂▃▃▃▃▃▃▂▂▂▂ |
| PAPAGIANNAKIS G, 2005, COMPUT ANIMAT VIRT W, V16, P11, [DOI](http://dx.doi.org/10.1002%2FCAV.53) | 2005 | 4.3825 | **2008** | 2013 | ▂▂▂▂▂▂▂▂▂▂▂▂▂▂▂▂▂▂▃▃▃▃▃▃▂▂▂▂ |
| BAKER S, 2004, INT J COMPUT VISION, V56, P221, [DOI](http://dx.doi.org/10.1023%2FB%3AVISI.0000011205.11775.FD) | 2004 | 8.5649 | **2008** | 2012 | ▂▂▂▂▂▂▂▂▂▂▂▂▂▂▂▂▂▂▃▃▃▃▃▂▂▂▂▂ |
| GORDON G, 2002, INTERNATIONAL SYMPOSIUM ON MIXED AND AUGMENTED REALITY, V, P14, [DOI](http://dx.doi.org/10.1109%2FISMAR.2002.1115063) | 2002 | 4.5043 | **2008** | 2010 | ▂▂▂▂▂▂▂▂▂▂▂▂▂▂▂▂▂▂▃▃▃▂▂▂▂▂▂▂ |
| MIKOLAJCZYK K, 2005, INT J COMPUT VISION, V65, P43, [DOI](http://dx.doi.org/10.1007%2FS11263-005-3848-X) | 2005 | 6.7095 | **2008** | 2012 | ▂▂▂▂▂▂▂▂▂▂▂▂▂▂▂▂▂▂▃▃▃▃▃▂▂▂▂▂ |
| KLEIN G, 2007, P 6 IEEE ACM INT S M, V, P | 2007 | 5.7617 | **2009** | 2014 | ▂▂▂▂▂▂▂▂▂▂▂▂▂▂▂▂▂▂▂▃▃▃▃▃▃▂▂▂ |
| BOTELLA CM, 2005, CYBERPSYCHOL BEHAV, V8, P162, [DOI](http://dx.doi.org/10.1089%2FCPB.2005.8.162) | 2005 | 6.0906 | **2009** | 2012 | ▂▂▂▂▂▂▂▂▂▂▂▂▂▂▂▂▂▂▂▃▃▃▃▂▂▂▂▂ |
| SNAVELY N, 2006, ACM T GRAPHIC, V25, P835, [DOI](http://dx.doi.org/10.1145%2F1141911.1141964) | 2006 | 7.5886 | **2009** | 2014 | ▂▂▂▂▂▂▂▂▂▂▂▂▂▂▂▂▂▂▂▃▃▃▃▃▃▂▂▂ |
| LEROTIC M, 2007, LECT NOTES COMPUT SC, V4792, P102 | 2007 | 3.8661 | **2009** | 2010 | ▂▂▂▂▂▂▂▂▂▂▂▂▂▂▂▂▂▂▂▃▃▂▂▂▂▂▂▂ |
| BOTDEN SMBI, 2007, WORLD J SURG, V31, P764, [DOI](http://dx.doi.org/10.1007%2FS00268-006-0724-Y) | 2007 | 5.0614 | **2009** | 2015 | ▂▂▂▂▂▂▂▂▂▂▂▂▂▂▂▂▂▂▂▃▃▃▃▃▃▃▂▂ |
| BAUMHAUER M, 2008, J ENDOUROL, V22, P751, [DOI](http://dx.doi.org/10.1089%2FEND.2007.9827) | 2008 | 5.2619 | **2009** | 2017 | ▂▂▂▂▂▂▂▂▂▂▂▂▂▂▂▂▂▂▂▃▃▃▃▃▃▃▃▃ |
| GEE JP, 2003, WHAT VIDEO GAMES HAVE TO TEACH US ABOUT LEARNING AND LITERACY, V, P1 | 2003 | 3.6462 | **2009** | 2011 | ▂▂▂▂▂▂▂▂▂▂▂▂▂▂▂▂▂▂▂▃▃▃▂▂▂▂▂▂ |
| SANDOR C, 2005, PERS UBIQUIT COMPUT, V9, P169, [DOI](http://dx.doi.org/10.1007%2FS00779-004-0328-1) | 2005 | 4.0176 | **2009** | 2013 | ▂▂▂▂▂▂▂▂▂▂▂▂▂▂▂▂▂▂▂▃▃▃▃▃▂▂▂▂ |
| SIELHORST T, 2008, J DISP TECHNOL, V4, P451, [DOI](http://dx.doi.org/10.1109%2FJDT.2008.2001575) | 2008 | 8.2035 | **2009** | 2017 | ▂▂▂▂▂▂▂▂▂▂▂▂▂▂▂▂▂▂▂▃▃▃▃▃▃▃▃▃ |
| OZUYSAL M, 2007, P IEEE C COMP VIS PA, V, P1 | 2007 | 4.9241 | **2009** | 2011 | ▂▂▂▂▂▂▂▂▂▂▂▂▂▂▂▂▂▂▂▃▃▃▂▂▂▂▂▂ |
| KLEIN G, 2007, P INT S MIX AUGM REA, V6, P225 | 2007 | 12.2943 | **2009** | 2015 | ▂▂▂▂▂▂▂▂▂▂▂▂▂▂▂▂▂▂▂▃▃▃▃▃▃▃▂▂ |
| HORNBAEK K, 2006, INT J HUM-COMPUT ST, V64, P79, [DOI](http://dx.doi.org/10.1016%2FJ.IJHCS.2005.06.002) | 2006 | 4.411 | **2009** | 2012 | ▂▂▂▂▂▂▂▂▂▂▂▂▂▂▂▂▂▂▂▃▃▃▃▂▂▂▂▂ |
| WANG XY, 2006, AUTOMAT CONSTR, V15, P314, [DOI](http://dx.doi.org/10.1016%2FJ.AUTCON.2005.06.002) | 2006 | 6.0794 | **2009** | 2014 | ▂▂▂▂▂▂▂▂▂▂▂▂▂▂▂▂▂▂▂▃▃▃▃▃▃▂▂▂ |
| HALLER M, 2007, EMERGING TECHNOLOGIES OF AUGMENTED REALITY: INTERFACES AND DESIGN, V, P1 | 2007 | 6.3336 | **2009** | 2015 | ▂▂▂▂▂▂▂▂▂▂▂▂▂▂▂▂▂▂▂▃▃▃▃▃▃▃▂▂ |
| KLEIN G, 2007, P INT S MIX AUGM REA, V, P | 2007 | 5.7596 | **2009** | 2013 | ▂▂▂▂▂▂▂▂▂▂▂▂▂▂▂▂▂▂▂▃▃▃▃▃▂▂▂▂ |
| KAMAT VR, 2007, J COMPUT CIVIL ENG, V21, P303, [DOI](http://dx.doi.org/10.1061%2F%28ASCE%290887-3801%282007%2921%3A5%28303%29) | 2007 | 5.7564 | **2009** | 2015 | ▂▂▂▂▂▂▂▂▂▂▂▂▂▂▂▂▂▂▂▃▃▃▃▃▃▃▂▂ |
| FIALA M, 2005, PROC CVPR IEEE, V, P590 | 2005 | 13.1812 | **2009** | 2013 | ▂▂▂▂▂▂▂▂▂▂▂▂▂▂▂▂▂▂▂▃▃▃▃▃▂▂▂▂ |
| WAGNER D, 2007, P 12 COMP VIS WINT W, V, P | 2007 | 4.4742 | **2009** | 2011 | ▂▂▂▂▂▂▂▂▂▂▂▂▂▂▂▂▂▂▂▃▃▃▂▂▂▂▂▂ |
| DAVISON AJ, 2007, IEEE T PATTERN ANAL, V29, P1052, [DOI](http://dx.doi.org/10.1109%2FTPAMI.2007.1049) | 2007 | 16.7738 | **2009** | 2015 | ▂▂▂▂▂▂▂▂▂▂▂▂▂▂▂▂▂▂▂▃▃▃▃▃▃▃▂▂ |
| WAGNER D, 2008, P 7 IEEE ACM INT S M, V, P125 | 2008 | 8.3211 | **2009** | 2012 | ▂▂▂▂▂▂▂▂▂▂▂▂▂▂▂▂▂▂▂▃▃▃▃▂▂▂▂▂ |
| UKIMURA O, 2008, J ENDOUROL, V22, P803, [DOI](http://dx.doi.org/10.1089%2FEND.2007.9823) | 2008 | 8.086 | **2009** | 2014 | ▂▂▂▂▂▂▂▂▂▂▂▂▂▂▂▂▂▂▂▃▃▃▃▃▃▂▂▂ |
| MIKOLAJCZYK K, 2005, IEEE T PATTERN ANAL, V27, P1615, [DOI](http://dx.doi.org/10.1109%2FTPAMI.2005.188) | 2005 | 15.8362 | **2009** | 2013 | ▂▂▂▂▂▂▂▂▂▂▂▂▂▂▂▂▂▂▂▃▃▃▃▃▂▂▂▂ |
| MIYASHITA T, 2008, P 7 IEEE ACM INT S M, V, P103 | 2008 | 3.717 | **2009** | 2012 | ▂▂▂▂▂▂▂▂▂▂▂▂▂▂▂▂▂▂▂▃▃▃▃▂▂▂▂▂ |
| ROSTEN E, 2006, EUR C COMP VIS, V1, P430 | 2006 | 4.3014 | **2010** | 2012 | ▂▂▂▂▂▂▂▂▂▂▂▂▂▂▂▂▂▂▂▂▃▃▃▂▂▂▂▂ |
| ONG SK, 2008, INT J PROD RES, V46, P2707, [DOI](http://dx.doi.org/10.1080%2F00207540601064773) | 2008 | 9.4781 | **2010** | 2017 | ▂▂▂▂▂▂▂▂▂▂▂▂▂▂▂▂▂▂▂▂▃▃▃▃▃▃▃▃ |
| CAWOOD S, 2008, AUGMENTED REALITY PR, V, P | 2008 | 3.888 | **2010** | 2012 | ▂▂▂▂▂▂▂▂▂▂▂▂▂▂▂▂▂▂▂▂▃▃▃▂▂▂▂▂ |
| SCHALL G, 2009, INT SYM MIX AUGMENT, V, P153, [DOI](http://dx.doi.org/10.1109%2FISMAR.2009.5336489) | 2009 | 3.4549 | **2010** | 2017 | ▂▂▂▂▂▂▂▂▂▂▂▂▂▂▂▂▂▂▂▂▃▃▃▃▃▃▃▃ |
| PARK H, 2009, COMPUT IND, V60, P114, [DOI](http://dx.doi.org/10.1016%2FJ.COMPIND.2008.09.001) | 2009 | 4.3902 | **2010** | 2013 | ▂▂▂▂▂▂▂▂▂▂▂▂▂▂▂▂▂▂▂▂▃▃▃▃▂▂▂▂ |
| SQUIRE K, 2007, J SCI EDUC TECHNOL, V16, P5 | 2007 | 8.6232 | **2010** | 2015 | ▂▂▂▂▂▂▂▂▂▂▂▂▂▂▂▂▂▂▂▂▃▃▃▃▃▃▂▂ |
| UKIMURA O, 2009, UROL CLIN N AM, V36, P115, [DOI](http://dx.doi.org/10.1016%2FJ.UCL.2009.02.012) | 2009 | 5.9752 | **2010** | 2012 | ▂▂▂▂▂▂▂▂▂▂▂▂▂▂▂▂▂▂▂▂▃▃▃▂▂▂▂▂ |
| WAGNER D, 2007, P 12 COMP VIS WINT W, V, P139 | 2007 | 9.6193 | **2010** | 2014 | ▂▂▂▂▂▂▂▂▂▂▂▂▂▂▂▂▂▂▂▂▃▃▃▃▃▂▂▂ |
| CHONG JWS, 2009, ROBOT CIM-INT MANUF, V25, P689, [DOI](http://dx.doi.org/10.1016%2FJ.RCIM.2008.05.002) | 2009 | 5.3043 | **2010** | 2017 | ▂▂▂▂▂▂▂▂▂▂▂▂▂▂▂▂▂▂▂▂▃▃▃▃▃▃▃▃ |
| HARDERS M, 2009, IEEE T VIS COMPUT GR, V15, P138, [DOI](http://dx.doi.org/10.1109%2FTVCG.2008.63) | 2009 | 5.1197 | **2010** | 2014 | ▂▂▂▂▂▂▂▂▂▂▂▂▂▂▂▂▂▂▂▂▃▃▃▃▃▂▂▂ |
| BEHZADAN AH, 2008, ADV ENG INFORM, V22, P90, [DOI](http://dx.doi.org/10.1016%2FJ.AEI.2007.08.005) | 2008 | 3.899 | **2010** | 2015 | ▂▂▂▂▂▂▂▂▂▂▂▂▂▂▂▂▂▂▂▂▃▃▃▃▃▃▂▂ |
| SU LM, 2009, UROLOGY, V73, P896, [DOI](http://dx.doi.org/10.1016%2FJ.UROLOGY.2008.11.040) | 2009 | 14.0312 | **2010** | 2014 | ▂▂▂▂▂▂▂▂▂▂▂▂▂▂▂▂▂▂▂▂▃▃▃▃▃▂▂▂ |
| WAGNER D, 2008, INT SYM MIX AUGMENT, V, P125, [DOI](http://dx.doi.org/10.1109%2FISMAR.2008.4637338) | 2008 | 7.6209 | **2010** | 2017 | ▂▂▂▂▂▂▂▂▂▂▂▂▂▂▂▂▂▂▂▂▃▃▃▃▃▃▃▃ |
| CASH DM, 2007, J GASTROINTEST SURG, V11, P844, [DOI](http://dx.doi.org/10.1007%2FS11605-007-0090-6) | 2007 | 3.6243 | **2010** | 2015 | ▂▂▂▂▂▂▂▂▂▂▂▂▂▂▂▂▂▂▂▂▃▃▃▃▃▃▂▂ |
| GABBARD JL, 2008, IEEE T VIS COMPUT GR, V14, P513, [DOI](http://dx.doi.org/10.1109%2FTVCG.2008.24) | 2008 | 5.9548 | **2010** | 2015 | ▂▂▂▂▂▂▂▂▂▂▂▂▂▂▂▂▂▂▂▂▃▃▃▃▃▃▂▂ |
| KERAWALLA L, 2006, VIRTUAL REALITY, V10, P163 | 2006 | 12.9505 | **2010** | 2014 | ▂▂▂▂▂▂▂▂▂▂▂▂▂▂▂▂▂▂▂▂▃▃▃▃▃▂▂▂ |
| KLEIN G, 2009, INT SYM MIX AUGMENT, V, P83, [DOI](http://dx.doi.org/10.1109%2FISMAR.2009.5336495) | 2009 | 10.5282 | **2011** | 2014 | ▂▂▂▂▂▂▂▂▂▂▂▂▂▂▂▂▂▂▂▂▂▃▃▃▃▂▂▂ |
| HENDERSON S, 2010, IEEE T VIS COMPUT GR, V16, P4, [DOI](http://dx.doi.org/10.1109%2FTVCG.2009.91) | 2010 | 5.557 | **2011** | 2015 | ▂▂▂▂▂▂▂▂▂▂▂▂▂▂▂▂▂▂▂▂▂▃▃▃▃▃▂▂ |
| ZHOU F, 2008, INT SYM MIX AUGMENT, V, P193, [DOI](http://dx.doi.org/10.1109%2FISMAR.2008.4637362) | 2008 | 27.8772 | **2011** | 2017 | ▂▂▂▂▂▂▂▂▂▂▂▂▂▂▂▂▂▂▂▂▂▃▃▃▃▃▃▃ |
| CHINTAMANI K, 2010, IEEE T SYST MAN CY A, V40, P29, [DOI](http://dx.doi.org/10.1109%2FTSMCA.2009.2030166) | 2010 | 4.6354 | **2011** | 2013 | ▂▂▂▂▂▂▂▂▂▂▂▂▂▂▂▂▂▂▂▂▂▃▃▃▂▂▂▂ |
| PAPAGIANNAKIS G, 2008, COMPUT ANIMAT VIRT W, V19, P3, [DOI](http://dx.doi.org/10.1002%2FCAV.221) | 2008 | 11.7835 | **2011** | 2017 | ▂▂▂▂▂▂▂▂▂▂▂▂▂▂▂▂▂▂▂▂▂▃▃▃▃▃▃▃ |
| ROSTEN E, 2006, LECT NOTES COMPUT SC, V3951, P430 | 2006 | 10.7948 | **2011** | 2014 | ▂▂▂▂▂▂▂▂▂▂▂▂▂▂▂▂▂▂▂▂▂▃▃▃▃▂▂▂ |
| SCHALL G, 2009, PERS UBIQUIT COMPUT, V13, P281, [DOI](http://dx.doi.org/10.1007%2FS00779-008-0204-5) | 2009 | 7.4923 | **2011** | 2017 | ▂▂▂▂▂▂▂▂▂▂▂▂▂▂▂▂▂▂▂▂▂▃▃▃▃▃▃▃ |
| GOLPARVAR-FARD M, 2009, J INF TECHNOL CONSTR, V14, P129 | 2009 | 5.7484 | **2011** | 2015 | ▂▂▂▂▂▂▂▂▂▂▂▂▂▂▂▂▂▂▂▂▂▃▃▃▃▃▂▂ |
| BAY H, 2008, COMPUT VIS IMAGE UND, V110, P346, [DOI](http://dx.doi.org/10.1016%2FJ.CVIU.2007.09.014) | 2008 | 27.7168 | **2011** | 2015 | ▂▂▂▂▂▂▂▂▂▂▂▂▂▂▂▂▂▂▂▂▂▃▃▃▃▃▂▂ |
| BLESER G, 2009, COMPUT GRAPH-UK, V33, P59, [DOI](http://dx.doi.org/10.1016%2FJ.CAG.2008.11.004) | 2009 | 3.8918 | **2011** | 2015 | ▂▂▂▂▂▂▂▂▂▂▂▂▂▂▂▂▂▂▂▂▂▃▃▃▃▃▂▂ |
| HENDERSON SJ, 2009, INT SYM MIX AUGMENT, V, P135, [DOI](http://dx.doi.org/10.1109%2FISMAR.2009.5336486) | 2009 | 9.3398 | **2011** | 2015 | ▂▂▂▂▂▂▂▂▂▂▂▂▂▂▂▂▂▂▂▂▂▃▃▃▃▃▂▂ |
| BOTELLA C, 2010, BEHAV THER, V41, P401, [DOI](http://dx.doi.org/10.1016%2FJ.BETH.2009.07.002) | 2010 | 4.8373 | **2011** | 2017 | ▂▂▂▂▂▂▂▂▂▂▂▂▂▂▂▂▂▂▂▂▂▃▃▃▃▃▃▃ |
| ROSTEN E, 2010, IEEE T PATTERN ANAL, V32, P105, [DOI](http://dx.doi.org/10.1109%2FTPAMI.2008.275) | 2010 | 7.6072 | **2011** | 2015 | ▂▂▂▂▂▂▂▂▂▂▂▂▂▂▂▂▂▂▂▂▂▃▃▃▃▃▂▂ |
| ARTH C, 2009, INT SYM MIX AUGMENT, V, P73, [DOI](http://dx.doi.org/10.1109%2FISMAR.2009.5336494) | 2009 | 4.9101 | **2011** | 2015 | ▂▂▂▂▂▂▂▂▂▂▂▂▂▂▂▂▂▂▂▂▂▃▃▃▃▃▂▂ |
| KLEIN GEORGE, 2007, P1, V, P, [DOI](http://dx.doi.org/10.1007%2F978-0-387-36523-7_1) | 2007 | 7.7964 | **2011** | 2014 | ▂▂▂▂▂▂▂▂▂▂▂▂▂▂▂▂▂▂▂▂▂▃▃▃▃▂▂▂ |
| WAGNER D, 2010, IEEE T VIS COMPUT GR, V16, P355, [DOI](http://dx.doi.org/10.1109%2FTVCG.2009.99) | 2010 | 14.6337 | **2011** | 2017 | ▂▂▂▂▂▂▂▂▂▂▂▂▂▂▂▂▂▂▂▂▂▃▃▃▃▃▃▃ |
| BAY H, 2006, LECT NOTES COMPUT SC, V3951, P404 | 2006 | 16.786 | **2011** | 2014 | ▂▂▂▂▂▂▂▂▂▂▂▂▂▂▂▂▂▂▂▂▂▃▃▃▃▂▂▂ |
| DEDE C, 2009, SCIENCE, V323, P66, [DOI](http://dx.doi.org/10.1126%2FSCIENCE.1167311) | 2009 | 9.2651 | **2011** | 2017 | ▂▂▂▂▂▂▂▂▂▂▂▂▂▂▂▂▂▂▂▂▂▃▃▃▃▃▃▃ |
| WANG XY, 2008, AUTOMAT CONSTR, V17, P399, [DOI](http://dx.doi.org/10.1016%2FJ.AUTCON.2007.07.002) | 2008 | 5.9444 | **2012** | 2015 | ▂▂▂▂▂▂▂▂▂▂▂▂▂▂▂▂▂▂▂▂▂▂▃▃▃▃▂▂ |
| EL SAYED NAM, 2011, COMPUT EDUC, V56, P1045, [DOI](http://dx.doi.org/10.1016%2FJ.COMPEDU.2010.10.019) | 2011 | 5.3604 | **2012** | 2014 | ▂▂▂▂▂▂▂▂▂▂▂▂▂▂▂▂▂▂▂▂▂▂▃▃▃▂▂▂ |
| SIMPFENDORFER T, 2011, J ENDOUROL, V25, P1841, [DOI](http://dx.doi.org/10.1089%2FEND.2010.0724) | 2011 | 7.2245 | **2012** | 2017 | ▂▂▂▂▂▂▂▂▂▂▂▂▂▂▂▂▂▂▂▂▂▂▃▃▃▃▃▃ |
| CASTLE R, 2008, TWELFTH IEEE INTERNATIONAL SYMPOSIUM ON WEARABLE COMPUTERS, V, P15, [DOI](http://dx.doi.org/10.1109%2FISWC.2008.4911577) | 2008 | 5.0771 | **2012** | 2017 | ▂▂▂▂▂▂▂▂▂▂▂▂▂▂▂▂▂▂▂▂▂▂▃▃▃▃▃▃ |
| CHENG DW, 2009, APPL OPTICS, V48, P2655, [DOI](http://dx.doi.org/10.1364%2FAO.48.002655) | 2009 | 6.5169 | **2012** | 2017 | ▂▂▂▂▂▂▂▂▂▂▂▂▂▂▂▂▂▂▂▂▂▂▃▃▃▃▃▃ |
| TEBER D, 2009, EUR UROL, V56, P332, [DOI](http://dx.doi.org/10.1016%2FJ.EURURO.2009.05.017) | 2009 | 12.2137 | **2012** | 2015 | ▂▂▂▂▂▂▂▂▂▂▂▂▂▂▂▂▂▂▂▂▂▂▃▃▃▃▂▂ |
| CARMIGNIANI J, 2011, HANDBOOK OF AUGMENTED REALITY, V, P3, [DOI](http://dx.doi.org/10.1007%2F978-1-4614-0064-6_1) | 2011 | 11.4642 | **2012** | 2017 | ▂▂▂▂▂▂▂▂▂▂▂▂▂▂▂▂▂▂▂▂▂▂▃▃▃▃▃▃ |
| REINHART G, 2008, CIRP ANN-MANUF TECHN, V57, P37, [DOI](http://dx.doi.org/10.1016%2FJ.CIRP.2008.03.120) | 2008 | 3.6163 | **2012** | 2013 | ▂▂▂▂▂▂▂▂▂▂▂▂▂▂▂▂▂▂▂▂▂▂▃▃▂▂▂▂ |
| NAVAB N, 2010, IEEE T MED IMAGING, V29, P1412, [DOI](http://dx.doi.org/10.1109%2FTMI.2009.2021947) | 2010 | 6.1548 | **2012** | 2017 | ▂▂▂▂▂▂▂▂▂▂▂▂▂▂▂▂▂▂▂▂▂▂▃▃▃▃▃▃ |
| AITTALA M, 2010, VISUAL COMPUT, V26, P669, [DOI](http://dx.doi.org/10.1007%2FS00371-010-0501-7) | 2010 | 6.4873 | **2012** | 2015 | ▂▂▂▂▂▂▂▂▂▂▂▂▂▂▂▂▂▂▂▂▂▂▃▃▃▃▂▂ |
| KRUIJFF E, 2010, P 9 IEEE INT S MIX A, V, P3 | 2010 | 5.7926 | **2012** | 2015 | ▂▂▂▂▂▂▂▂▂▂▂▂▂▂▂▂▂▂▂▂▂▂▃▃▃▃▂▂ |
| SQUIRE K, 2007, J LEARN SCI, V16, P371, [DOI](http://dx.doi.org/10.1080%2F10508400701413435) | 2007 | 9.1816 | **2012** | 2015 | ▂▂▂▂▂▂▂▂▂▂▂▂▂▂▂▂▂▂▂▂▂▂▃▃▃▃▂▂ |
| VOLONTE F, 2011, J HEPATO-BIL-PAN SCI, V18, P506, [DOI](http://dx.doi.org/10.1007%2FS00534-011-0385-6) | 2011 | 7.2477 | **2012** | 2015 | ▂▂▂▂▂▂▂▂▂▂▂▂▂▂▂▂▂▂▂▂▂▂▃▃▃▃▂▂ |
| MARTIN-GUTIERREZ J, 2010, COMPUT GRAPH-UK, V34, P77, [DOI](http://dx.doi.org/10.1016%2FJ.CAG.2009.11.003) | 2010 | 14.0842 | **2012** | 2017 | ▂▂▂▂▂▂▂▂▂▂▂▂▂▂▂▂▂▂▂▂▂▂▃▃▃▃▃▃ |
| NEWCOMBE RA, 2011, INT SYM MIX AUGMENT, V, P127, [DOI](http://dx.doi.org/10.1109%2FISMAR.2011.6092378) | 2011 | 29.7244 | **2012** | 2017 | ▂▂▂▂▂▂▂▂▂▂▂▂▂▂▂▂▂▂▂▂▂▂▃▃▃▃▃▃ |
| CALONDER M, 2010, LECT NOTES COMPUT SC, V6314, P778, [DOI](http://dx.doi.org/10.1007%2F978-3-642-15561-1_56) | 2010 | 7.9432 | **2012** | 2015 | ▂▂▂▂▂▂▂▂▂▂▂▂▂▂▂▂▂▂▂▂▂▂▃▃▃▃▂▂ |
| BOTDEN SMBI, 2009, SURG ENDOSC, V23, P1693, [DOI](http://dx.doi.org/10.1007%2FS00464-008-0144-1) | 2009 | 5.8851 | **2012** | 2017 | ▂▂▂▂▂▂▂▂▂▂▂▂▂▂▂▂▂▂▂▂▂▂▃▃▃▃▃▃ |
| SHIN DH, 2008, AUTOMAT CONSTR, V17, P882, [DOI](http://dx.doi.org/10.1016%2FJ.AUTCON.2008.02.012) | 2008 | 9.8274 | **2012** | 2015 | ▂▂▂▂▂▂▂▂▂▂▂▂▂▂▂▂▂▂▂▂▂▂▃▃▃▃▂▂ |
| BICHLMEIER C, 2009, IEEE T MED IMAGING, V28, P1498, [DOI](http://dx.doi.org/10.1109%2FTMI.2009.2018622) | 2009 | 5.4671 | **2012** | 2017 | ▂▂▂▂▂▂▂▂▂▂▂▂▂▂▂▂▂▂▂▂▂▂▃▃▃▃▃▃ |
| SHEKHAR R, 2010, SURG ENDOSC, V24, P1976, [DOI](http://dx.doi.org/10.1007%2FS00464-010-0890-8) | 2010 | 7.6436 | **2013** | 2017 | ▂▂▂▂▂▂▂▂▂▂▂▂▂▂▂▂▂▂▂▂▂▂▂▃▃▃▃▃ |
| ANDUJAR JM, 2011, IEEE T EDUC, V54, P492, [DOI](http://dx.doi.org/10.1109%2FTE.2010.2085047) | 2011 | 8.4382 | **2013** | 2017 | ▂▂▂▂▂▂▂▂▂▂▂▂▂▂▂▂▂▂▂▂▂▂▂▃▃▃▃▃ |
| KIM Y, 2008, INT J, V2, P4 | 2008 | 4.8477 | **2013** | 2017 | ▂▂▂▂▂▂▂▂▂▂▂▂▂▂▂▂▂▂▂▂▂▂▂▃▃▃▃▃ |
| BARNA J, 2012, MANUFACTURING TECHNO, V12, P2 | 2012 | 3.915 | **2013** | 2014 | ▂▂▂▂▂▂▂▂▂▂▂▂▂▂▂▂▂▂▂▂▂▂▂▃▃▂▂▂ |
| LEPETIT V, 2009, INT J COMPUT VISION, V81, P155, [DOI](http://dx.doi.org/10.1007%2FS11263-008-0152-6) | 2009 | 9.3451 | **2013** | 2017 | ▂▂▂▂▂▂▂▂▂▂▂▂▂▂▂▂▂▂▂▂▂▂▂▃▃▃▃▃ |
| NICOLAU S, 2011, SURG ONCOL, V20, P189, [DOI](http://dx.doi.org/10.1016%2FJ.SURONC.2011.07.002) | 2011 | 26.5063 | **2013** | 2017 | ▂▂▂▂▂▂▂▂▂▂▂▂▂▂▂▂▂▂▂▂▂▂▂▃▃▃▃▃ |
| CHEN CM, 2012, COMPUT EDUC, V59, P638, [DOI](http://dx.doi.org/10.1016%2FJ.COMPEDU.2012.03.001) | 2012 | 13.0276 | **2013** | 2017 | ▂▂▂▂▂▂▂▂▂▂▂▂▂▂▂▂▂▂▂▂▂▂▂▃▃▃▃▃ |
| KLOPFER E, 2008, ETR&D-EDUC TECH RES, V56, P203, [DOI](http://dx.doi.org/10.1007%2FS11423-007-9037-6) | 2008 | 17.7289 | **2013** | 2017 | ▂▂▂▂▂▂▂▂▂▂▂▂▂▂▂▂▂▂▂▂▂▂▂▃▃▃▃▃ |
| MORRISON A, 2009, CHI2009: PROCEEDINGS OF THE 27TH ANNUAL CHI CONFERENCE ON HUMAN FACTORS IN COMPUTING SYSTEMS, VOLS 1-4, P1889 | 2009 | 5.3552 | **2013** | 2017 | ▂▂▂▂▂▂▂▂▂▂▂▂▂▂▂▂▂▂▂▂▂▂▂▃▃▃▃▃ |
| KLOPFER E, 2008, AUGMENTED LEARNING R, V, P | 2008 | 8.4615 | **2013** | 2017 | ▂▂▂▂▂▂▂▂▂▂▂▂▂▂▂▂▂▂▂▂▂▂▂▃▃▃▃▃ |
| FRITZ J, 2012, AM J ROENTGENOL, V198, , [DOI](http://dx.doi.org/10.2214%2FAJR.11.6918) | 2012 | 4.0618 | **2013** | 2017 | ▂▂▂▂▂▂▂▂▂▂▂▂▂▂▂▂▂▂▂▂▂▂▂▃▃▃▃▃ |
| JOHNSON L, 2011, 2011 HORIZON REPORT, V, P | 2011 | 9.3353 | **2013** | 2017 | ▂▂▂▂▂▂▂▂▂▂▂▂▂▂▂▂▂▂▂▂▂▂▂▃▃▃▃▃ |
| LIAO HE, 2010, IEEE T BIO-MED ENG, V57, P1476, [DOI](http://dx.doi.org/10.1109%2FTBME.2010.2040278) | 2010 | 7.9425 | **2013** | 2017 | ▂▂▂▂▂▂▂▂▂▂▂▂▂▂▂▂▂▂▂▂▂▂▂▃▃▃▃▃ |
| ARVANITIS T N, 2007, PERS UBIQUIT COMPUT, V13, P243 | 2007 | 4.901 | **2013** | 2014 | ▂▂▂▂▂▂▂▂▂▂▂▂▂▂▂▂▂▂▂▂▂▂▂▃▃▂▂▂ |
| BRADSKI G, 2008, LEARNING OPENCV COMP, V, P | 2008 | 8.0901 | **2013** | 2017 | ▂▂▂▂▂▂▂▂▂▂▂▂▂▂▂▂▂▂▂▂▂▂▂▃▃▃▃▃ |
| HENDERSON S, 2011, IEEE T VIS COMPUT GR, V17, P1355, [DOI](http://dx.doi.org/10.1109%2FTVCG.2010.245) | 2011 | 14.4401 | **2013** | 2017 | ▂▂▂▂▂▂▂▂▂▂▂▂▂▂▂▂▂▂▂▂▂▂▂▃▃▃▃▃ |
| ALAHI A, 2012, PROC CVPR IEEE, V, P510, [DOI](http://dx.doi.org/10.1109%2FCVPR.2012.6247715) | 2012 | 9.2324 | **2013** | 2017 | ▂▂▂▂▂▂▂▂▂▂▂▂▂▂▂▂▂▂▂▂▂▂▂▃▃▃▃▃ |
| DUNLEAVY M, 2009, J SCI EDUC TECHNOL, V18, P7, [DOI](http://dx.doi.org/10.1007%2FS10956-008-9119-1) | 2009 | 33.223 | **2013** | 2017 | ▂▂▂▂▂▂▂▂▂▂▂▂▂▂▂▂▂▂▂▂▂▂▂▃▃▃▃▃ |
| JOHNSON L, 2012, NMC HORIZON REPORT 2, V, P | 2012 | 4.7563 | **2013** | 2017 | ▂▂▂▂▂▂▂▂▂▂▂▂▂▂▂▂▂▂▂▂▂▂▂▃▃▃▃▃ |
| CHEN YC, 2011, J PROF ISS ENG ED PR, V137, P267, [DOI](http://dx.doi.org/10.1061%2F%28ASCE%29EI.1943-5541.0000078) | 2011 | 7.8577 | **2013** | 2017 | ▂▂▂▂▂▂▂▂▂▂▂▂▂▂▂▂▂▂▂▂▂▂▂▃▃▃▃▃ |
| LEE T, 2007, ELEVENTH IEEE INTERNATIONAL SYMPOSIUM ON WEARABLE COMPUTERS, V, P83 | 2007 | 9.6828 | **2013** | 2015 | ▂▂▂▂▂▂▂▂▂▂▂▂▂▂▂▂▂▂▂▂▂▂▂▃▃▃▂▂ |
| MARTIN S, 2011, COMPUT EDUC, V57, P1893, [DOI](http://dx.doi.org/10.1016%2FJ.COMPEDU.2011.04.003) | 2011 | 13.8306 | **2013** | 2017 | ▂▂▂▂▂▂▂▂▂▂▂▂▂▂▂▂▂▂▂▂▂▂▂▃▃▃▃▃ |
| OLSSON T, 2013, PERS UBIQUIT COMPUT, V17, P287, [DOI](http://dx.doi.org/10.1007%2FS00779-011-0494-X) | 2013 | 9.0747 | **2014** | 2017 | ▂▂▂▂▂▂▂▂▂▂▂▂▂▂▂▂▂▂▂▂▂▂▂▂▃▃▃▃ |
| FONSECA D, 2014, COMPUT HUM BEHAV, V31, P434, [DOI](http://dx.doi.org/10.1016%2FJ.CHB.2013.03.006) | 2014 | 8.3792 | **2014** | 2017 | ▂▂▂▂▂▂▂▂▂▂▂▂▂▂▂▂▂▂▂▂▂▂▂▂▃▃▃▃ |
| KESIM M, 2012, PROCD SOC BEHV, V47, P297, [DOI](http://dx.doi.org/10.1016%2FJ.SBSPRO.2012.06.654) | 2012 | 8.7118 | **2014** | 2017 | ▂▂▂▂▂▂▂▂▂▂▂▂▂▂▂▂▂▂▂▂▂▂▂▂▃▃▃▃ |
| CRAIG A B, 2013, UNDERSTANDING AUGMEN, V, P | 2013 | 9.7703 | **2014** | 2017 | ▂▂▂▂▂▂▂▂▂▂▂▂▂▂▂▂▂▂▂▂▂▂▂▂▃▃▃▃ |
| BUJAK KR, 2013, COMPUT EDUC, V68, P536, [DOI](http://dx.doi.org/10.1016%2FJ.COMPEDU.2013.02.017) | 2013 | 13.0732 | **2014** | 2017 | ▂▂▂▂▂▂▂▂▂▂▂▂▂▂▂▂▂▂▂▂▂▂▂▂▃▃▃▃ |
| IBANEZ MB, 2014, COMPUT EDUC, V71, P1, [DOI](http://dx.doi.org/10.1016%2FJ.COMPEDU.2013.09.004) | 2014 | 18.1453 | **2014** | 2017 | ▂▂▂▂▂▂▂▂▂▂▂▂▂▂▂▂▂▂▂▂▂▂▂▂▃▃▃▃ |
| SILTANEN SANNI, 2012, THEORY APPL MARKER B, V, P | 2012 | 8.1553 | **2014** | 2015 | ▂▂▂▂▂▂▂▂▂▂▂▂▂▂▂▂▂▂▂▂▂▂▂▂▃▃▂▂ |
| GARRIDO-JURADO S, 2014, PATTERN RECOGN, V47, P2280, [DOI](http://dx.doi.org/10.1016%2FJ.PATCOG.2014.01.005) | 2014 | 9.4073 | **2014** | 2017 | ▂▂▂▂▂▂▂▂▂▂▂▂▂▂▂▂▂▂▂▂▂▂▂▂▃▃▃▃ |
| CUENDET S, 2013, COMPUT EDUC, V68, P557, [DOI](http://dx.doi.org/10.1016%2FJ.COMPEDU.2013.02.015) | 2013 | 13.9973 | **2014** | 2017 | ▂▂▂▂▂▂▂▂▂▂▂▂▂▂▂▂▂▂▂▂▂▂▂▂▃▃▃▃ |
| CHI HL, 2013, AUTOMAT CONSTR, V33, P116, [DOI](http://dx.doi.org/10.1016%2FJ.AUTCON.2012.12.017) | 2013 | 9.4683 | **2014** | 2017 | ▂▂▂▂▂▂▂▂▂▂▂▂▂▂▂▂▂▂▂▂▂▂▂▂▃▃▃▃ |
| BILLINGHURST M, 2012, COMPUTER, V45, P56, [DOI](http://dx.doi.org/10.1109%2FMC.2012.111) | 2012 | 13.9277 | **2014** | 2017 | ▂▂▂▂▂▂▂▂▂▂▂▂▂▂▂▂▂▂▂▂▂▂▂▂▃▃▃▃ |
| RUBLEE E, 2011, 2011 IEEE INTERNATIONAL CONFERENCE ON COMPUTER VISION (ICCV), V, P2564, [DOI](http://dx.doi.org/10.1109%2FICCV.2011.6126544) | 2011 | 19.4391 | **2014** | 2017 | ▂▂▂▂▂▂▂▂▂▂▂▂▂▂▂▂▂▂▂▂▂▂▂▂▃▃▃▃ |
| WEBEL S, 2013, ROBOT AUTON SYST, V61, P398, [DOI](http://dx.doi.org/10.1016%2FJ.ROBOT.2012.09.013) | 2013 | 10.4967 | **2014** | 2017 | ▂▂▂▂▂▂▂▂▂▂▂▂▂▂▂▂▂▂▂▂▂▂▂▂▃▃▃▃ |
| NEE AYC, 2012, CIRP ANN-MANUF TECHN, V61, P657, [DOI](http://dx.doi.org/10.1016%2FJ.CIRP.2012.05.010) | 2012 | 19.1061 | **2014** | 2017 | ▂▂▂▂▂▂▂▂▂▂▂▂▂▂▂▂▂▂▂▂▂▂▂▂▃▃▃▃ |
| DI SERIO A, 2013, COMPUT EDUC, V68, P586, [DOI](http://dx.doi.org/10.1016%2FJ.COMPEDU.2012.03.002) | 2013 | 25.5017 | **2014** | 2017 | ▂▂▂▂▂▂▂▂▂▂▂▂▂▂▂▂▂▂▂▂▂▂▂▂▃▃▃▃ |
| WU HK, 2013, COMPUT EDUC, V62, P41, [DOI](http://dx.doi.org/10.1016%2FJ.COMPEDU.2012.10.024) | 2013 | 47.2306 | **2014** | 2017 | ▂▂▂▂▂▂▂▂▂▂▂▂▂▂▂▂▂▂▂▂▂▂▂▂▃▃▃▃ |
| IRIZARRY J, 2013, AUTOMAT CONSTR, V33, P11, [DOI](http://dx.doi.org/10.1016%2FJ.AUTCON.2012.09.002) | 2013 | 7.2609 | **2014** | 2017 | ▂▂▂▂▂▂▂▂▂▂▂▂▂▂▂▂▂▂▂▂▂▂▂▂▃▃▃▃ |
| LEE K, 2012, TECHTRENDS, V56, P13 | 2012 | 15.6554 | **2014** | 2017 | ▂▂▂▂▂▂▂▂▂▂▂▂▂▂▂▂▂▂▂▂▂▂▂▂▃▃▃▃ |
| HANSEN C, 2010, INT J COMPUT ASS RAD, V5, P133, [DOI](http://dx.doi.org/10.1007%2FS11548-009-0365-3) | 2010 | 7.3808 | **2014** | 2017 | ▂▂▂▂▂▂▂▂▂▂▂▂▂▂▂▂▂▂▂▂▂▂▂▂▃▃▃▃ |
| VAN KREVELEN D W F, 2010, INT J VIRTUAL REALIT, V9, P1 | 2010 | 50.9895 | **2014** | 2017 | ▂▂▂▂▂▂▂▂▂▂▂▂▂▂▂▂▂▂▂▂▂▂▂▂▃▃▃▃ |
| MAIER-HEIN L, 2013, MED IMAGE ANAL, V17, P974, [DOI](http://dx.doi.org/10.1016%2FJ.MEDIA.2013.04.003) | 2013 | 7.231 | **2014** | 2017 | ▂▂▂▂▂▂▂▂▂▂▂▂▂▂▂▂▂▂▂▂▂▂▂▂▃▃▃▃ |
| YUEN S, 2011, J ED TECHNOLOGY DEV, V4, P119 | 2011 | 20.471 | **2014** | 2017 | ▂▂▂▂▂▂▂▂▂▂▂▂▂▂▂▂▂▂▂▂▂▂▂▂▃▃▃▃ |
| LIN TJ, 2013, COMPUT EDUC, V68, P314, [DOI](http://dx.doi.org/10.1016%2FJ.COMPEDU.2013.05.011) | 2013 | 10.8908 | **2014** | 2017 | ▂▂▂▂▂▂▂▂▂▂▂▂▂▂▂▂▂▂▂▂▂▂▂▂▃▃▃▃ |
| CARMIGNIANI J, 2011, MULTIMED TOOLS APPL, V51, P341, [DOI](http://dx.doi.org/10.1007%2FS11042-010-0660-6) | 2011 | 26.0552 | **2014** | 2017 | ▂▂▂▂▂▂▂▂▂▂▂▂▂▂▂▂▂▂▂▂▂▂▂▂▃▃▃▃ |
| CHANG KE, 2014, COMPUT EDUC, V71, P185, [DOI](http://dx.doi.org/10.1016%2FJ.COMPEDU.2013.09.022) | 2014 | 13.4057 | **2014** | 2017 | ▂▂▂▂▂▂▂▂▂▂▂▂▂▂▂▂▂▂▂▂▂▂▂▂▃▃▃▃ |
| LEUTENEGGER S, 2011, 2011 IEEE INTERNATIONAL CONFERENCE ON COMPUTER VISION (ICCV), V, P2548, [DOI](http://dx.doi.org/10.1109%2FICCV.2011.6126542) | 2011 | 9.1964 | **2014** | 2017 | ▂▂▂▂▂▂▂▂▂▂▂▂▂▂▂▂▂▂▂▂▂▂▂▂▃▃▃▃ |
| NAKAMOTO M, 2012, CURR OPIN UROL, V22, P121, [DOI](http://dx.doi.org/10.1097%2FMOU.0B013E3283501774) | 2012 | 4.42 | **2014** | 2017 | ▂▂▂▂▂▂▂▂▂▂▂▂▂▂▂▂▂▂▂▂▂▂▂▂▃▃▃▃ |
| KAMARAINEN AM, 2013, COMPUT EDUC, V68, P545, [DOI](http://dx.doi.org/10.1016%2FJ.COMPEDU.2013.02.018) | 2013 | 12.7405 | **2014** | 2017 | ▂▂▂▂▂▂▂▂▂▂▂▂▂▂▂▂▂▂▂▂▂▂▂▂▃▃▃▃ |
| NAVAB N, 2012, COMPUTER, V45, P48, [DOI](http://dx.doi.org/10.1109%2FMC.2012.75) | 2012 | 8.1303 | **2015** | 2017 | ▂▂▂▂▂▂▂▂▂▂▂▂▂▂▂▂▂▂▂▂▂▂▂▂▂▃▃▃ |
| CHIANG THC, 2014, EDUC TECHNOL SOC, V17, P352 | 2014 | 9.5094 | **2015** | 2017 | ▂▂▂▂▂▂▂▂▂▂▂▂▂▂▂▂▂▂▂▂▂▂▂▂▂▃▃▃ |
| KANG X, 2014, SURG ENDOSC, V28, P2227, [DOI](http://dx.doi.org/10.1007%2FS00464-014-3433-X) | 2014 | 7.9478 | **2015** | 2017 | ▂▂▂▂▂▂▂▂▂▂▂▂▂▂▂▂▂▂▂▂▂▂▂▂▂▃▃▃ |
| WEI XD, 2015, COMPUT EDUC, V81, P221, [DOI](http://dx.doi.org/10.1016%2FJ.COMPEDU.2014.10.017) | 2015 | 8.2096 | **2015** | 2017 | ▂▂▂▂▂▂▂▂▂▂▂▂▂▂▂▂▂▂▂▂▂▂▂▂▂▃▃▃ |
| BRESSLER DM, 2013, J COMPUT ASSIST LEAR, V29, P505, [DOI](http://dx.doi.org/10.1111%2FJCAL.12008) | 2013 | 8.5525 | **2015** | 2017 | ▂▂▂▂▂▂▂▂▂▂▂▂▂▂▂▂▂▂▂▂▂▂▂▂▂▃▃▃ |
| HUA H, 2014, OPT EXPRESS, V22, P13484, [DOI](http://dx.doi.org/10.1364%2FOE.22.013484) | 2014 | 10.5486 | **2015** | 2017 | ▂▂▂▂▂▂▂▂▂▂▂▂▂▂▂▂▂▂▂▂▂▂▂▂▂▃▃▃ |
| KERSTEN-OERTEL M, 2013, COMPUT MED IMAG GRAP, V37, P98, [DOI](http://dx.doi.org/10.1016%2FJ.COMPMEDIMAG.2013.01.009) | 2013 | 7.4241 | **2015** | 2017 | ▂▂▂▂▂▂▂▂▂▂▂▂▂▂▂▂▂▂▂▂▂▂▂▂▂▃▃▃ |
| HURST W, 2013, MULTIMED TOOLS APPL, V62, P233, [DOI](http://dx.doi.org/10.1007%2FS11042-011-0983-Y) | 2013 | 6.387 | **2015** | 2017 | ▂▂▂▂▂▂▂▂▂▂▂▂▂▂▂▂▂▂▂▂▂▂▂▂▂▃▃▃ |
| WOJCIECHOWSKI R, 2013, COMPUT EDUC, V68, P570, [DOI](http://dx.doi.org/10.1016%2FJ.COMPEDU.2013.02.014) | 2013 | 14.5423 | **2015** | 2017 | ▂▂▂▂▂▂▂▂▂▂▂▂▂▂▂▂▂▂▂▂▂▂▂▂▂▃▃▃ |
| BOWER M, 2014, EDUC MEDIA INT, V51, P1, [DOI](http://dx.doi.org/10.1080%2F09523987.2014.889400) | 2014 | 12.4573 | **2015** | 2017 | ▂▂▂▂▂▂▂▂▂▂▂▂▂▂▂▂▂▂▂▂▂▂▂▂▂▃▃▃ |
| FITZGERALD E, 2013, INT J MOB BLENDED LE, V5, P43, [DOI](http://dx.doi.org/10.4018%2FIJMBL.2013100103) | 2013 | 6.9107 | **2015** | 2017 | ▂▂▂▂▂▂▂▂▂▂▂▂▂▂▂▂▂▂▂▂▂▂▂▂▂▃▃▃ |
| SOMMERAUER P, 2014, COMPUT EDUC, V79, P59, [DOI](http://dx.doi.org/10.1016%2FJ.COMPEDU.2014.07.013) | 2014 | 12.5462 | **2015** | 2017 | ▂▂▂▂▂▂▂▂▂▂▂▂▂▂▂▂▂▂▂▂▂▂▂▂▂▃▃▃ |
| CHENG KH, 2013, J SCI EDUC TECHNOL, V22, P449, [DOI](http://dx.doi.org/10.1007%2FS10956-012-9405-9) | 2013 | 19.3347 | **2015** | 2017 | ▂▂▂▂▂▂▂▂▂▂▂▂▂▂▂▂▂▂▂▂▂▂▂▂▂▃▃▃ |
| YOON SA, 2012, INT J COMP-SUPP COLL, V7, P519, [DOI](http://dx.doi.org/10.1007%2FS11412-012-9156-X) | 2012 | 7.5147 | **2015** | 2017 | ▂▂▂▂▂▂▂▂▂▂▂▂▂▂▂▂▂▂▂▂▂▂▂▂▂▃▃▃ |
