# Supplementary material for: The Past, Present, and Future of Virtual and Augmented Reality Research: A Network and Cluster Analysis of the Literature
Source: Front Psychol. 2018 Nov 6;9:2086. doi: 10.3389/fpsyg.2018.02086 (PMC6232426; doi:10.3389/fpsyg.2018.02086)
Supplement: Supplementary file 1 [file Data_Sheet_1.ZIP › Top 486 References (VR) with Strongest Citation Bursts.docx]

**Top 486 References with Strongest Citation Bursts**

| **References** | **Year** | **Strength** | **Begin** | **End** | **1990 - 2016** |
| --- | --- | --- | --- | --- | --- |
| GIBSON W, 1984, NEUROMANCER, V, P | 1984 | 3.4233 | **1990** | 1992 | ▃▃▃▂▂▂▂▂▂▂▂▂▂▂▂▂▂▂▂▂▂▂▂▂▂▂▂ |
| ELLIS STEPHEN R, 1991, PICTORIAL COMMUNICAT, V, P | 1991 | 5.8545 | **1991** | 1999 | ▂▃▃▃▃▃▃▃▃▃▂▂▂▂▂▂▂▂▂▂▂▂▂▂▂▂▂ |
| HELSEL S K, 1991, VIRTUAL REALITY THEO, V, P | 1991 | 7.0812 | **1991** | 1996 | ▂▃▃▃▃▃▃▂▂▂▂▂▂▂▂▂▂▂▂▂▂▂▂▂▂▂▂ |
| LAUREL BRENDA, 1991, COMPUTERS THEATER, V, P | 1991 | 5.8545 | **1991** | 1999 | ▂▃▃▃▃▃▃▃▃▃▂▂▂▂▂▂▂▂▂▂▂▂▂▂▂▂▂ |
| RHEINGOLD H, 1991, VIRTUAL REALITY, V, P | 1991 | 25.8316 | **1991** | 1999 | ▂▃▃▃▃▃▃▃▃▃▂▂▂▂▂▂▂▂▂▂▂▂▂▂▂▂▂ |
| BENEDIKT MICHAEL L, 1991, CYBERSPACE 1 STEPS, V, P | 1991 | 7.5816 | **1991** | 1997 | ▂▃▃▃▃▃▃▃▂▂▂▂▂▂▂▂▂▂▂▂▂▂▂▂▂▂▂ |
| KRUEGER MYRON, 1991, ARTIFICIAL REALITY, V, P | 1991 | 9.3718 | **1991** | 1999 | ▂▃▃▃▃▃▃▃▃▃▂▂▂▂▂▂▂▂▂▂▂▂▂▂▂▂▂ |
| WENZEL EM, 1992, PRESENCE-TELEOP VIRT, V1, P80 | 1992 | 7.0676 | **1992** | 1999 | ▂▂▃▃▃▃▃▃▃▃▂▂▂▂▂▂▂▂▂▂▂▂▂▂▂▂▂ |
| ELLIS S R, 1991, COMPUTING SYSTEMS IN ENGINEERING, V2, P, [DOI](http://dx.doi.org/10.1016%2F0956-0521%2891%2990001-L) | 1991 | 8.2469 | **1992** | 1999 | ▂▂▃▃▃▃▃▃▃▃▂▂▂▂▂▂▂▂▂▂▂▂▂▂▂▂▂ |
| BIOCCA F, 1992, J COMMUN, V42, P5, [DOI](http://dx.doi.org/10.1111%2FJ.1460-2466.1992.TB00810.X) | 1992 | 8.9488 | **1992** | 2000 | ▂▂▃▃▃▃▃▃▃▃▃▂▂▂▂▂▂▂▂▂▂▂▂▂▂▂▂ |
| SHERIDAN T B, 1992, PRESENCE-TELEOP VIRT, V1, P120 | 1992 | 9.5089 | **1992** | 2000 | ▂▂▃▃▃▃▃▃▃▃▃▂▂▂▂▂▂▂▂▂▂▂▂▂▂▂▂ |
| SHAPIRO MA, 1992, J COMMUN, V42, P94, [DOI](http://dx.doi.org/10.1111%2FJ.1460-2466.1992.TB00813.X) | 1992 | 5.0308 | **1992** | 2000 | ▂▂▃▃▃▃▃▃▃▃▃▂▂▂▂▂▂▂▂▂▂▂▂▂▂▂▂ |
| LANIER J, 1992, J COMMUN, V42, P150, [DOI](http://dx.doi.org/10.1111%2FJ.1460-2466.1992.TB00816.X) | 1992 | 3.3531 | **1992** | 2000 | ▂▂▃▃▃▃▃▃▃▃▃▂▂▂▂▂▂▂▂▂▂▂▂▂▂▂▂ |
| STEUER J, 1992, J COMMUN, V42, P73, [DOI](http://dx.doi.org/10.1111%2FJ.1460-2466.1992.TB00812.X) | 1992 | 13.432 | **1992** | 2000 | ▂▂▃▃▃▃▃▃▃▃▃▂▂▂▂▂▂▂▂▂▂▂▂▂▂▂▂ |
| APPINO PA, 1992, PRESENCE-TELEOP VIRT, V1, P1 | 1992 | 3.9122 | **1992** | 2000 | ▂▂▃▃▃▃▃▃▃▃▃▂▂▂▂▂▂▂▂▂▂▂▂▂▂▂▂ |
| FOLEY JD, 1990, COMPUTER GRAPHICS PR, V, P | 1990 | 12.3 | **1992** | 1998 | ▂▂▃▃▃▃▃▃▃▂▂▂▂▂▂▂▂▂▂▂▂▂▂▂▂▂▂ |
| BIOCCA F, 1992, J COMMUN, V42, P23, [DOI](http://dx.doi.org/10.1111%2FJ.1460-2466.1992.TB00811.X) | 1992 | 3.5321 | **1992** | 1999 | ▂▂▃▃▃▃▃▃▃▃▂▂▂▂▂▂▂▂▂▂▂▂▂▂▂▂▂ |
| STONE A, 1991, CYBERSPACE 1 STEPS, V, P81 | 1991 | 3.5321 | **1992** | 1999 | ▂▂▃▃▃▃▃▃▃▃▂▂▂▂▂▂▂▂▂▂▂▂▂▂▂▂▂ |
| LOOMIS J M, 1992, PRESENCE-TELEOP VIRT, V1, P113 | 1992 | 5.2994 | **1992** | 1999 | ▂▂▃▃▃▃▃▃▃▃▂▂▂▂▂▂▂▂▂▂▂▂▂▂▂▂▂ |
| ROBINETT W, 1992, PRESENCE, V1, P45 | 1992 | 6.145 | **1992** | 1998 | ▂▂▃▃▃▃▃▃▃▂▂▂▂▂▂▂▂▂▂▂▂▂▂▂▂▂▂ |
| ENVIRONMENTS VIRTUAL, 1992, PRESENCE-TELEOP VIRT, V1, P127 | 1992 | 10.6293 | **1992** | 2000 | ▂▂▃▃▃▃▃▃▃▃▃▂▂▂▂▂▂▂▂▂▂▂▂▂▂▂▂ |
| REGIAN JW, 1992, J COMMUN, V42, P136, [DOI](http://dx.doi.org/10.1111%2FJ.1460-2466.1992.TB00815.X) | 1992 | 12.8713 | **1992** | 2000 | ▂▂▃▃▃▃▃▃▃▃▃▂▂▂▂▂▂▂▂▂▂▂▂▂▂▂▂ |
| BURDEA G, 1992, PRESENCE-TELEOP VIRT, V1, P18 | 1992 | 6.773 | **1993** | 2000 | ▂▂▂▃▃▃▃▃▃▃▃▂▂▂▂▂▂▂▂▂▂▂▂▂▂▂▂ |
| BRYSON S, 1992, IEEE COMPUT GRAPH, V12, P25, [DOI](http://dx.doi.org/10.1109%2F38.144824) | 1992 | 3.9493 | **1993** | 2000 | ▂▂▂▃▃▃▃▃▃▃▃▂▂▂▂▂▂▂▂▂▂▂▂▂▂▂▂ |
| DEERING M, 1992, COMP GRAPH, V26, P195 | 1992 | 7.9031 | **1993** | 2000 | ▂▂▂▃▃▃▃▃▃▃▃▂▂▂▂▂▂▂▂▂▂▂▂▂▂▂▂ |
| BAJURA M, 1992, COMP GRAPH, V26, P203 | 1992 | 12.9932 | **1993** | 2000 | ▂▂▂▃▃▃▃▃▃▃▃▂▂▂▂▂▂▂▂▂▂▂▂▂▂▂▂ |
| BROOKS JR FP, 1990, ACM COMPUTER GRAPHIC, V24, P177, [DOI](http://dx.doi.org/10.1145%2F97880.97899) | 1990 | 7.4388 | **1993** | 1998 | ▂▂▂▃▃▃▃▃▃▂▂▂▂▂▂▂▂▂▂▂▂▂▂▂▂▂▂ |
| KALAWSKY R S, 1993, SCI VIRTUAL REALITY, V, P | 1993 | 21.7537 | **1993** | 2001 | ▂▂▂▃▃▃▃▃▃▃▃▃▂▂▂▂▂▂▂▂▂▂▂▂▂▂▂ |
| MEYER K, 1992, PRESENCE, V, P173 | 1992 | 6.2081 | **1993** | 2000 | ▂▂▂▃▃▃▃▃▃▃▃▂▂▂▂▂▂▂▂▂▂▂▂▂▂▂▂ |
| WEISER M, 1991, SCI AM, V265, P94 | 1991 | 3.7176 | **1993** | 1998 | ▂▂▂▃▃▃▃▃▃▂▂▂▂▂▂▂▂▂▂▂▂▂▂▂▂▂▂ |
| HEIM M, 1993, METAPHYSICS VIRTUAL, V, P | 1993 | 7.9419 | **1993** | 2001 | ▂▂▂▃▃▃▃▃▃▃▃▃▂▂▂▂▂▂▂▂▂▂▂▂▂▂▂ |
| EARNSHAW R A, 1993, VIRTUAL REALITY SYST, V, P | 1993 | 7.4118 | **1993** | 2001 | ▂▂▂▃▃▃▃▃▃▃▃▃▂▂▂▂▂▂▂▂▂▂▂▂▂▂▂ |
| HOLLOWAY R, 1992, COMPUTER GRAPHICS IN, V, P109 | 1992 | 5.3471 | **1993** | 1999 | ▂▂▂▃▃▃▃▃▃▃▂▂▂▂▂▂▂▂▂▂▂▂▂▂▂▂▂ |
| SHAW C, 1993, ACM T INFORM SYST, V11, P287, [DOI](http://dx.doi.org/10.1145%2F159161.173948) | 1993 | 13.2472 | **1993** | 2001 | ▂▂▂▃▃▃▃▃▃▃▃▃▂▂▂▂▂▂▂▂▂▂▂▂▂▂▂ |
| AUKSTAKALNIS S, 1992, SILICON MIRAGE ART S, V, P | 1992 | 11.8614 | **1993** | 2000 | ▂▂▂▃▃▃▃▃▃▃▃▂▂▂▂▂▂▂▂▂▂▂▂▂▂▂▂ |
| KENNEDY RS, 1993, INT J AVIAT PSYCHOL, V3, P203 | 1993 | 11.8102 | **1994** | 2001 | ▂▂▂▂▃▃▃▃▃▃▃▃▂▂▂▂▂▂▂▂▂▂▂▂▂▂▂ |
| SATAVA RM, 1993, SURG ENDOSC-ULTRAS, V7, P203, [DOI](http://dx.doi.org/10.1007%2FBF00594110) | 1993 | 26.3624 | **1994** | 2001 | ▂▂▂▂▃▃▃▃▃▃▃▃▂▂▂▂▂▂▂▂▂▂▂▂▂▂▂ |
| IWATA H, 1990, COMPUTER GRAPHICS, V24, P | 1990 | 6.2686 | **1994** | 1998 | ▂▂▂▂▃▃▃▃▃▂▂▂▂▂▂▂▂▂▂▂▂▂▂▂▂▂▂ |
| WOOLLEY BENJAMIN, 1992, VIRTUAL WORLDS JOURN, V, P | 1992 | 3.6062 | **1994** | 1999 | ▂▂▂▂▃▃▃▃▃▃▂▂▂▂▂▂▂▂▂▂▂▂▂▂▂▂▂ |
| GLASSNER A, 1989, INTRO RAY TRACING, V, P | 1989 | 3.9802 | **1994** | 1996 | ▂▂▂▂▃▃▃▂▂▂▂▂▂▂▂▂▂▂▂▂▂▂▂▂▂▂▂ |
| CARLSSON C, 1993, COMPUT GRAPH, V17, P663, [DOI](http://dx.doi.org/10.1016%2F0097-8493%2893%2990115-P) | 1993 | 11.2725 | **1994** | 2001 | ▂▂▂▂▃▃▃▃▃▃▃▃▂▂▂▂▂▂▂▂▂▂▂▂▂▂▂ |
| REGAN EC, 1994, AVIAT SPACE ENVIR MD, V65, P527 | 1994 | 14.1489 | **1994** | 2002 | ▂▂▂▂▃▃▃▃▃▃▃▃▃▂▂▂▂▂▂▂▂▂▂▂▂▂▂ |
| SATAVA RM, 1992, MINIM INVASIV THER, V1, P357 | 1992 | 9.7191 | **1994** | 2000 | ▂▂▂▂▃▃▃▃▃▃▃▂▂▂▂▂▂▂▂▂▂▂▂▂▂▂▂ |
| GREEN PE, 1991, AM SURGEON, V57, P192 | 1991 | 5.6412 | **1994** | 1998 | ▂▂▂▂▃▃▃▃▃▂▂▂▂▂▂▂▂▂▂▂▂▂▂▂▂▂▂ |
| CRUZ-NEIRA C, 1993, COMPUTER GRAPHICS PROCEEDINGS, V, P | 1993 | 5.8998 | **1994** | 2001 | ▂▂▂▂▃▃▃▃▃▃▃▃▂▂▂▂▂▂▂▂▂▂▂▂▂▂▂ |
| MONWILLIAMS M, 1993, OPHTHAL PHYSL OPT, V13, P387, [DOI](http://dx.doi.org/10.1111%2FJ.1475-1313.1993.TB00496.X) | 1993 | 12.5828 | **1994** | 2000 | ▂▂▂▂▃▃▃▃▃▃▃▂▂▂▂▂▂▂▂▂▂▂▂▂▂▂▂ |
| KOZAK JJ, 1993, ERGONOMICS, V36, P777, [DOI](http://dx.doi.org/10.1080%2F00140139308967941) | 1993 | 10.891 | **1995** | 2001 | ▂▂▂▂▂▃▃▃▃▃▃▃▂▂▂▂▂▂▂▂▂▂▂▂▂▂▂ |
| ROTHBAUM BO, 1995, BEHAV THER, V26, P547, [DOI](http://dx.doi.org/10.1016%2FS0005-7894%2805%2980100-5) | 1995 | 10.9751 | **1995** | 2003 | ▂▂▂▂▂▃▃▃▃▃▃▃▃▃▂▂▂▂▂▂▂▂▂▂▂▂▂ |
| BENEDIKT M, 1991, CYBERSPACE 1 STEPS, V, P | 1991 | 3.653 | **1995** | 1999 | ▂▂▂▂▂▃▃▃▃▃▂▂▂▂▂▂▂▂▂▂▂▂▂▂▂▂▂ |
| HETTINGER LJ, 1992, PRESENCE-TELEOP VIRT, V1, P306 | 1992 | 4.8715 | **1995** | 1999 | ▂▂▂▂▂▃▃▃▃▃▂▂▂▂▂▂▂▂▂▂▂▂▂▂▂▂▂ |
| CRUZ-NEIRA C, 1993, P 20 ANN C COMP GRAP, V,, P | 1993 | 16.8961 | **1995** | 2001 | ▂▂▂▂▂▃▃▃▃▃▃▃▂▂▂▂▂▂▂▂▂▂▂▂▂▂▂ |
| BIOCCA F, 1995, COMMUNICATION AGE VI, V, P | 1995 | 4.2897 | **1995** | 2003 | ▂▂▂▂▂▃▃▃▃▃▃▃▃▃▂▂▂▂▂▂▂▂▂▂▂▂▂ |
| LATTA JN, 1994, IEEE COMPUT GRAPH, V14, P23, [DOI](http://dx.doi.org/10.1109%2F38.250915) | 1994 | 4.8966 | **1995** | 2001 | ▂▂▂▂▂▃▃▃▃▃▃▃▂▂▂▂▂▂▂▂▂▂▂▂▂▂▂ |
| RUSHTON S, 1994, DISPLAYS, V15, P255, [DOI](http://dx.doi.org/10.1016%2F0141-9382%2894%2990073-6) | 1994 | 6.9514 | **1995** | 2000 | ▂▂▂▂▂▃▃▃▃▃▃▂▂▂▂▂▂▂▂▂▂▂▂▂▂▂▂ |
| BURDEA G, 1994, VIRTUAL REALITY TECH, V, P | 1994 | 23.6387 | **1995** | 2002 | ▂▂▂▂▂▃▃▃▃▃▃▃▃▂▂▂▂▂▂▂▂▂▂▂▂▂▂ |
| TERZOPOULOS D, 1993, IEEE T PATTERN ANAL, V15, P569, [DOI](http://dx.doi.org/10.1109%2F34.216726) | 1993 | 5.4411 | **1995** | 2001 | ▂▂▂▂▂▃▃▃▃▃▃▃▂▂▂▂▂▂▂▂▂▂▂▂▂▂▂ |
| STURMAN DJ, 1994, IEEE COMPUT GRAPH, V14, P30, [DOI](http://dx.doi.org/10.1109%2F38.250916) | 1994 | 7.62 | **1995** | 2001 | ▂▂▂▂▂▃▃▃▃▃▃▃▂▂▂▂▂▂▂▂▂▂▂▂▂▂▂ |
| BADLER NI, 1993, SIMULATING HUMANS CO, V, P | 1993 | 5.4411 | **1995** | 2001 | ▂▂▂▂▂▃▃▃▃▃▃▃▂▂▂▂▂▂▂▂▂▂▂▂▂▂▂ |
| ELLIS SR, 1994, IEEE COMPUT GRAPH, V14, P17, [DOI](http://dx.doi.org/10.1109%2F38.250914) | 1994 | 9.8003 | **1995** | 2001 | ▂▂▂▂▂▃▃▃▃▃▃▃▂▂▂▂▂▂▂▂▂▂▂▂▂▂▂ |
| BRICKEN M, 1993, VIRTUAL REALITY APPL, V, P199 | 1993 | 3.653 | **1995** | 1999 | ▂▂▂▂▂▃▃▃▃▃▂▂▂▂▂▂▂▂▂▂▂▂▂▂▂▂▂ |
| SATAVA R M, 1993, ENDOSC SURG ALLIED TECHNOL, V1, P151 | 1993 | 3.8078 | **1995** | 2001 | ▂▂▂▂▂▃▃▃▃▃▃▃▂▂▂▂▂▂▂▂▂▂▂▂▂▂▂ |
| ARTHUR KW, 1993, ACM T INFORM SYST, V11, P239, [DOI](http://dx.doi.org/10.1145%2F159161.155359) | 1993 | 4.8966 | **1995** | 2001 | ▂▂▂▂▂▃▃▃▃▃▃▃▂▂▂▂▂▂▂▂▂▂▂▂▂▂▂ |
| SHERIDAN T B, 1992, TELEROBOTICS AUTOMAT, V, P | 1992 | 4.8715 | **1995** | 1999 | ▂▂▂▂▂▃▃▃▃▃▂▂▂▂▂▂▂▂▂▂▂▂▂▂▂▂▂ |
| KENNEDY RS, 1992, PRESENCE, V1, P295 | 1992 | 7.5313 | **1995** | 2000 | ▂▂▂▂▂▃▃▃▃▃▃▂▂▂▂▂▂▂▂▂▂▂▂▂▂▂▂ |
| LORENSEN WE, 1995, ST HEAL T, V18, P221 | 1995 | 4.6328 | **1995** | 2000 | ▂▂▂▂▂▃▃▃▃▃▃▂▂▂▂▂▂▂▂▂▂▂▂▂▂▂▂ |
| HELD RM, 1992, PRESENCE-TELEOP VIRT, V1, P109 | 1992 | 4.6328 | **1995** | 2000 | ▂▂▂▂▂▃▃▃▃▃▃▂▂▂▂▂▂▂▂▂▂▂▂▂▂▂▂ |
| KIJIMA R, 1994, PRESENCE-TELEOP VIRT, V3, P45 | 1994 | 3.8923 | **1995** | 1997 | ▂▂▂▂▂▃▃▃▂▂▂▂▂▂▂▂▂▂▂▂▂▂▂▂▂▂▂ |
| KALTENBORN KF, 1993, METHOD INFORM MED, V32, P407 | 1993 | 11.982 | **1995** | 2001 | ▂▂▂▂▂▃▃▃▃▃▃▃▂▂▂▂▂▂▂▂▂▂▂▂▂▂▂ |
| LOFTIN RB, 1995, IEEE COMPUT GRAPH, V15, P31, [DOI](http://dx.doi.org/10.1109%2F38.403825) | 1995 | 6.8136 | **1996** | 2003 | ▂▂▂▂▂▂▃▃▃▃▃▃▃▃▂▂▂▂▂▂▂▂▂▂▂▂▂ |
| COVER SA, 1993, IEEE COMPUT GRAPH, V13, P68, [DOI](http://dx.doi.org/10.1109%2F38.252559) | 1993 | 8.3128 | **1996** | 2001 | ▂▂▂▂▂▂▃▃▃▃▃▃▂▂▂▂▂▂▂▂▂▂▂▂▂▂▂ |
| AYACHE N, 1995, IMAGE VISION COMPUT, V13, P295, [DOI](http://dx.doi.org/10.1016%2F0262-8856%2895%2999717-F) | 1995 | 3.4287 | **1996** | 2000 | ▂▂▂▂▂▂▃▃▃▃▃▂▂▂▂▂▂▂▂▂▂▂▂▂▂▂▂ |
| ADAM JA, 1993, IEEE SPECTRUM, V30, P22, [DOI](http://dx.doi.org/10.1109%2F6.237580) | 1993 | 5.5396 | **1996** | 2001 | ▂▂▂▂▂▂▃▃▃▃▃▃▂▂▂▂▂▂▂▂▂▂▂▂▂▂▂ |
| ROTHBAUM BO, 1995, AM J PSYCHIAT, V152, P626 | 1995 | 38.9385 | **1996** | 2003 | ▂▂▂▂▂▂▃▃▃▃▃▃▃▃▂▂▂▂▂▂▂▂▂▂▂▂▂ |
| LIANG JD, 1994, COMPUT GRAPH, V18, P499, [DOI](http://dx.doi.org/10.1016%2F0097-8493%2894%2990062-0) | 1994 | 7.8367 | **1996** | 2002 | ▂▂▂▂▂▂▃▃▃▃▃▃▃▂▂▂▂▂▂▂▂▂▂▂▂▂▂ |
| BRICKMANN J, 1996, TRENDS BIOTECHNOL, V14, P167, [DOI](http://dx.doi.org/10.1016%2F0167-7799%2896%2910022-6) | 1996 | 3.5331 | **1996** | 2000 | ▂▂▂▂▂▂▃▃▃▃▃▂▂▂▂▂▂▂▂▂▂▂▂▂▂▂▂ |
| CHEN S E, 1995, COMPUTER GRAPHICS PROCEEDINGS. SIGGRAPH 95, V, P | 1995 | 5.3523 | **1996** | 2003 | ▂▂▂▂▂▂▃▃▃▃▃▃▃▃▂▂▂▂▂▂▂▂▂▂▂▂▂ |
| BELL G, 1995, VIRTUAL REALITY MODE, V, P | 1995 | 4.3311 | **1996** | 1999 | ▂▂▂▂▂▂▃▃▃▃▂▂▂▂▂▂▂▂▂▂▂▂▂▂▂▂▂ |
| ALTOBELLI DE, 1993, PLAST RECONSTR SURG, V92, P576, [DOI](http://dx.doi.org/10.1097%2F00006534-199309001-00003) | 1993 | 5.5396 | **1996** | 2001 | ▂▂▂▂▂▂▃▃▃▃▃▃▂▂▂▂▂▂▂▂▂▂▂▂▂▂▂ |
| KENNEDY RS, 1996, INT J HUM-COMPUT INT, V8, P25 | 1996 | 8.5263 | **1996** | 2004 | ▂▂▂▂▂▂▃▃▃▃▃▃▃▃▃▂▂▂▂▂▂▂▂▂▂▂▂ |
| WANN J, 1996, INT J HUM-COMPUT ST, V44, P829, [DOI](http://dx.doi.org/10.1006%2FIJHC.1996.0035) | 1996 | 10.4532 | **1996** | 2002 | ▂▂▂▂▂▂▃▃▃▃▃▃▃▂▂▂▂▂▂▂▂▂▂▂▂▂▂ |
| DURLACH N I, 1995, VIRTUAL REALITY SCI, V, P | 1995 | 23.9153 | **1996** | 2003 | ▂▂▂▂▂▂▃▃▃▃▃▃▃▃▂▂▂▂▂▂▂▂▂▂▂▂▂ |
| HODGES LF, 1995, COMPUTER, V28, P27, [DOI](http://dx.doi.org/10.1109%2F2.391038) | 1995 | 12.178 | **1996** | 2003 | ▂▂▂▂▂▂▃▃▃▃▃▃▃▃▂▂▂▂▂▂▂▂▂▂▂▂▂ |
| HOHNE KH, 1992, IEEE COMPUT GRAPH, V12, P72, [DOI](http://dx.doi.org/10.1109%2F38.144829) | 1992 | 4.3311 | **1996** | 1999 | ▂▂▂▂▂▂▃▃▃▃▂▂▂▂▂▂▂▂▂▂▂▂▂▂▂▂▂ |
| POSTON T, 1996, COMMUN ACM, V39, P37, [DOI](http://dx.doi.org/10.1145%2F229459.229464) | 1996 | 5.1967 | **1996** | 2000 | ▂▂▂▂▂▂▃▃▃▃▃▂▂▂▂▂▂▂▂▂▂▂▂▂▂▂▂ |
| SLATER M, 1994, PRESENCE-TELEOP VIRT, V3, P130 | 1994 | 8.8831 | **1996** | 2002 | ▂▂▂▂▂▂▃▃▃▃▃▃▃▂▂▂▂▂▂▂▂▂▂▂▂▂▂ |
| SACHS E, 1991, IEEE COMPUT GRAPH, V11, P18, [DOI](http://dx.doi.org/10.1109%2F38.103389) | 1991 | 4.9503 | **1996** | 1999 | ▂▂▂▂▂▂▃▃▃▃▂▂▂▂▂▂▂▂▂▂▂▂▂▂▂▂▂ |
| CALVIN J, 1993, P IEEE VIRT REAL ANN, V, P450, [DOI](http://dx.doi.org/10.1109%2FVRAIS.1993.380745) | 1993 | 6.6486 | **1996** | 2001 | ▂▂▂▂▂▂▃▃▃▃▃▃▂▂▂▂▂▂▂▂▂▂▂▂▂▂▂ |
| LAMPTON D R, 1994, PRESENCE, V3, P145 | 1994 | 4.6998 | **1996** | 2002 | ▂▂▂▂▂▂▃▃▃▃▃▃▃▂▂▂▂▂▂▂▂▂▂▂▂▂▂ |
| OTA D, 1995, COMPUT BIOL MED, V25, P127, [DOI](http://dx.doi.org/10.1016%2F0010-4825%2894%2900009-F) | 1995 | 15.108 | **1996** | 2003 | ▂▂▂▂▂▂▃▃▃▃▃▃▃▃▂▂▂▂▂▂▂▂▂▂▂▂▂ |
| CABRAL B, 1994, P 1994 S VOL VIS, V, P91, [DOI](http://dx.doi.org/10.1145%2F197938.197972) | 1994 | 5.2224 | **1996** | 2002 | ▂▂▂▂▂▂▃▃▃▃▃▃▃▂▂▂▂▂▂▂▂▂▂▂▂▂▂ |
| REGAN C, 1995, VIRTUAL REALITY, V1, P17, [DOI](http://dx.doi.org/10.1007%2FBF02009710) | 1995 | 6.094 | **1996** | 2001 | ▂▂▂▂▂▂▃▃▃▃▃▃▂▂▂▂▂▂▂▂▂▂▂▂▂▂▂ |
| WICKHAM JEA, 1994, BRIT MED J, V308, P193 | 1994 | 5.2224 | **1996** | 2002 | ▂▂▂▂▂▂▃▃▃▃▃▃▃▂▂▂▂▂▂▂▂▂▂▂▂▂▂ |
| SATAVA RM, 1995, J MED SYST, V19, P275, [DOI](http://dx.doi.org/10.1007%2FBF02257178) | 1995 | 10.2824 | **1996** | 2002 | ▂▂▂▂▂▂▃▃▃▃▃▃▃▂▂▂▂▂▂▂▂▂▂▂▂▂▂ |
| ROEHL B, 1997, LATE NIGHT VRML 2 0, V, P | 1997 | 8.0693 | **1997** | 2004 | ▂▂▂▂▂▂▂▃▃▃▃▃▃▃▃▂▂▂▂▂▂▂▂▂▂▂▂ |
| HENDRIX C, 1996, PRESENCE-TELEOP VIRT, V5, P274 | 1996 | 4.277 | **1997** | 2002 | ▂▂▂▂▂▂▂▃▃▃▃▃▃▂▂▂▂▂▂▂▂▂▂▂▂▂▂ |
| MARSH A, 1996, INT C PAR DISTR PROC, V, P | 1996 | 4.4184 | **1997** | 1999 | ▂▂▂▂▂▂▂▃▃▃▂▂▂▂▂▂▂▂▂▂▂▂▂▂▂▂▂ |
| GLANTZ K, 1997, PRESENCE-TELEOP VIRT, V6, P87 | 1997 | 7.7937 | **1997** | 2003 | ▂▂▂▂▂▂▂▃▃▃▃▃▃▃▂▂▂▂▂▂▂▂▂▂▂▂▂ |
| SATAVA R M, 1994, ARTIF INTELL MED, V6, P281, [DOI](http://dx.doi.org/10.1016%2F0933-3657%2894%2990033-7) | 1994 | 3.3975 | **1997** | 2001 | ▂▂▂▂▂▂▂▃▃▃▃▃▂▂▂▂▂▂▂▂▂▂▂▂▂▂▂ |
| MORELAND J L, 1997, VRML 2 0 SOURCEBOOK, V, P | 1997 | 15.9321 | **1997** | 2004 | ▂▂▂▂▂▂▂▃▃▃▃▃▃▃▃▂▂▂▂▂▂▂▂▂▂▂▂ |
| AMERICAN PSYCHIATRIC ASSOCIATION, 1994, DIAGN STAT MAN MENT, V, P | 1994 | 16.0674 | **1997** | 2002 | ▂▂▂▂▂▂▂▃▃▃▃▃▃▂▂▂▂▂▂▂▂▂▂▂▂▂▂ |
| BOWSKILL J, 1995, COMPUTER GRAPHICS, V29, P | 1995 | 4.4184 | **1997** | 1999 | ▂▂▂▂▂▂▂▃▃▃▂▂▂▂▂▂▂▂▂▂▂▂▂▂▂▂▂ |
| KRUEGER W, 1994, IEEE COMPUT GRAPH, V14, P12, [DOI](http://dx.doi.org/10.1109%2F38.279036) | 1994 | 6.4176 | **1997** | 2002 | ▂▂▂▂▂▂▂▃▃▃▃▃▃▂▂▂▂▂▂▂▂▂▂▂▂▂▂ |
| VINING DJ, 1996, CHEST, V109, P549, [DOI](http://dx.doi.org/10.1378%2FCHEST.109.2.549) | 1996 | 12.3113 | **1997** | 2002 | ▂▂▂▂▂▂▂▃▃▃▃▃▃▂▂▂▂▂▂▂▂▂▂▂▂▂▂ |
| ROTHBAUM BO, 1996, BEHAV RES THER, V34, P477, [DOI](http://dx.doi.org/10.1016%2F0005-7967%2896%2900007-1) | 1996 | 17.0905 | **1997** | 2004 | ▂▂▂▂▂▂▂▃▃▃▃▃▃▃▃▂▂▂▂▂▂▂▂▂▂▂▂ |
| BURDEA GC, 1996, FORCE TOUCH FEEDBACK, V, P | 1996 | 32.4198 | **1997** | 2004 | ▂▂▂▂▂▂▂▃▃▃▃▃▃▃▃▂▂▂▂▂▂▂▂▂▂▂▂ |
| SATAVA RM, 1995, COMPUT BIOL MED, V25, P229, [DOI](http://dx.doi.org/10.1016%2F0010-4825%2894%2900006-C) | 1995 | 6.4176 | **1997** | 2002 | ▂▂▂▂▂▂▂▃▃▃▃▃▃▂▂▂▂▂▂▂▂▂▂▂▂▂▂ |
| CARLSSON C, 1993, P IEEE VIRT REAL ANN, V, P394, [DOI](http://dx.doi.org/10.1109%2FVRAIS.1993.380753) | 1993 | 6.2313 | **1997** | 2001 | ▂▂▂▂▂▂▂▃▃▃▃▃▂▂▂▂▂▂▂▂▂▂▂▂▂▂▂ |
| VINING DJ, 1996, RADIOLOGY, V200, P30 | 1996 | 8.3733 | **1997** | 2001 | ▂▂▂▂▂▂▂▃▃▃▃▃▂▂▂▂▂▂▂▂▂▂▂▂▂▂▂ |
| GREENHALGH C, 1995, ACM T COMPUTER HUMAN, V2, P239, [DOI](http://dx.doi.org/10.1145%2F210079.210088) | 1995 | 6.4886 | **1997** | 2003 | ▂▂▂▂▂▂▂▃▃▃▃▃▃▃▂▂▂▂▂▂▂▂▂▂▂▂▂ |
| BURT DER, 1995, BRIT J ANAESTH, V75, P472 | 1995 | 5.989 | **1997** | 2003 | ▂▂▂▂▂▂▂▃▃▃▃▃▃▃▂▂▂▂▂▂▂▂▂▂▂▂▂ |
| SATAVA RM, 1993, SURG ENDOSC-ULTRAS, V7, P429, [DOI](http://dx.doi.org/10.1007%2FBF00311737) | 1993 | 3.9641 | **1997** | 2001 | ▂▂▂▂▂▂▂▃▃▃▃▃▂▂▂▂▂▂▂▂▂▂▂▂▂▂▂ |
| LEVOY M, 1996, COMPUTER GRAPHICS PROCEEDINGS. SIGGRAPH 96, V, P, [DOI](http://dx.doi.org/10.1145%2F237170.237199) | 1996 | 7.4881 | **1997** | 2003 | ▂▂▂▂▂▂▂▃▃▃▃▃▃▃▂▂▂▂▂▂▂▂▂▂▂▂▂ |
| DAVIS CP, 1996, RADIOLOGY, V199, P37 | 1996 | 3.9641 | **1997** | 2001 | ▂▂▂▂▂▂▂▃▃▃▃▃▂▂▂▂▂▂▂▂▂▂▂▂▂▂▂ |
| COLEMAN J, 1994, BRIT J SURG, V81, P1709, [DOI](http://dx.doi.org/10.1002%2FBJS.1800811204) | 1994 | 13.9205 | **1997** | 2002 | ▂▂▂▂▂▂▂▃▃▃▃▃▃▂▂▂▂▂▂▂▂▂▂▂▂▂▂ |
| FEINER S, 1993, COMMUN ACM, V36, P53, [DOI](http://dx.doi.org/10.1145%2F159544.159587) | 1993 | 5.6643 | **1997** | 2001 | ▂▂▂▂▂▂▂▃▃▃▃▃▂▂▂▂▂▂▂▂▂▂▂▂▂▂▂ |
| SCHROEDER R, 1996, POSSIBLE WORLDS SOCI, V, P | 1996 | 4.8113 | **1997** | 2000 | ▂▂▂▂▂▂▂▃▃▃▃▂▂▂▂▂▂▂▂▂▂▂▂▂▂▂▂ |
| PEREDNIA DA, 1995, JAMA-J AM MED ASSOC, V273, P483, [DOI](http://dx.doi.org/10.1001%2FJAMA.273.6.483) | 1995 | 6.2313 | **1997** | 2001 | ▂▂▂▂▂▂▂▃▃▃▃▃▂▂▂▂▂▂▂▂▂▂▂▂▂▂▂ |
| BRO- NIELSEN M, 1996, COMPUT GRAPH FORUM, V15, P57 | 1996 | 4.6534 | **1997** | 2002 | ▂▂▂▂▂▂▂▃▃▃▃▃▃▂▂▂▂▂▂▂▂▂▂▂▂▂▂ |
| RUBIN GD, 1996, RADIOLOGY, V199, P321 | 1996 | 11.0807 | **1997** | 2002 | ▂▂▂▂▂▂▂▃▃▃▃▃▃▂▂▂▂▂▂▂▂▂▂▂▂▂▂ |
| WANN JP, 1995, VISION RES, V35, P2731, [DOI](http://dx.doi.org/10.1016%2F0042-6989%2895%2900018-U) | 1995 | 6.9883 | **1997** | 2003 | ▂▂▂▂▂▂▂▃▃▃▃▃▃▃▂▂▂▂▂▂▂▂▂▂▂▂▂ |
| HAGSAND O, 1996, IEEE MULTIMEDIA, V3, P30, [DOI](http://dx.doi.org/10.1109%2F93.486702) | 1996 | 6.4176 | **1997** | 2002 | ▂▂▂▂▂▂▂▃▃▃▃▃▃▂▂▂▂▂▂▂▂▂▂▂▂▂▂ |
| HARA AK, 1996, RADIOLOGY, V200, P49 | 1996 | 5.8823 | **1997** | 2002 | ▂▂▂▂▂▂▂▃▃▃▃▃▃▂▂▂▂▂▂▂▂▂▂▂▂▂▂ |
| GLEASON PL, 1994, STEREOT FUNCT NEUROS, V63, P139, [DOI](http://dx.doi.org/10.1159%2F000100305) | 1994 | 5.6643 | **1997** | 2001 | ▂▂▂▂▂▂▂▃▃▃▃▃▂▂▂▂▂▂▂▂▂▂▂▂▂▂▂ |
| MAGUIRE EA, 1996, P ROY SOC B-BIOL SCI, V263, P1745, [DOI](http://dx.doi.org/10.1098%2FRSPB.1996.0255) | 1996 | 6.4547 | **1997** | 2004 | ▂▂▂▂▂▂▂▃▃▃▃▃▃▃▃▂▂▂▂▂▂▂▂▂▂▂▂ |
| MCMILLAN L, 1995, COMPUTER GRAPHICS PROCEEDINGS. SIGGRAPH 95, V, P | 1995 | 3.9914 | **1997** | 2003 | ▂▂▂▂▂▂▂▃▃▃▃▃▃▃▂▂▂▂▂▂▂▂▂▂▂▂▂ |
| MAGUIRE EA, 1996, NEUROPSYCHOLOGIA, V34, P993, [DOI](http://dx.doi.org/10.1016%2F0028-3932%2896%2900022-X) | 1996 | 5.989 | **1997** | 2003 | ▂▂▂▂▂▂▂▃▃▃▃▃▃▃▂▂▂▂▂▂▂▂▂▂▂▂▂ |
| KIM WS, 1996, PRESENCE-TELEOP VIRT, V5, P173 | 1996 | 3.7869 | **1997** | 1999 | ▂▂▂▂▂▂▂▃▃▃▂▂▂▂▂▂▂▂▂▂▂▂▂▂▂▂▂ |
| AGUIRRE GK, 1996, CEREB CORTEX, V6, P823, [DOI](http://dx.doi.org/10.1093%2FCERCOR%2F6.6.823) | 1996 | 10.6118 | **1997** | 2004 | ▂▂▂▂▂▂▂▃▃▃▃▃▃▃▃▂▂▂▂▂▂▂▂▂▂▂▂ |
| BOWERSOX JC, 1996, J VASC SURG, V23, P281, [DOI](http://dx.doi.org/10.1016%2FS0741-5214%2896%2970272-0) | 1996 | 4.6357 | **1998** | 2003 | ▂▂▂▂▂▂▂▂▃▃▃▃▃▃▂▂▂▂▂▂▂▂▂▂▂▂▂ |
| HOFFMAN H, 1997, ACAD MED, V72, P1076, [DOI](http://dx.doi.org/10.1097%2F00001888-199712000-00018) | 1997 | 9.9273 | **1998** | 2005 | ▂▂▂▂▂▂▂▂▃▃▃▃▃▃▃▃▂▂▂▂▂▂▂▂▂▂▂ |
| MARESCAUX J, 1998, ANN SURG, V228, P627, [DOI](http://dx.doi.org/10.1097%2F00000658-199811000-00001) | 1998 | 14.2505 | **1998** | 2003 | ▂▂▂▂▂▂▂▂▃▃▃▃▃▃▂▂▂▂▂▂▂▂▂▂▂▂▂ |
| WILSON PN, 1997, DISABIL REHABIL, V19, P213 | 1997 | 15.555 | **1998** | 2004 | ▂▂▂▂▂▂▂▂▃▃▃▃▃▃▃▂▂▂▂▂▂▂▂▂▂▂▂ |
| CZERNUSZENKO M, 1997, COMPUTER GRAPHICS, V31, P | 1997 | 12.5231 | **1998** | 2005 | ▂▂▂▂▂▂▂▂▃▃▃▃▃▃▃▃▂▂▂▂▂▂▂▂▂▂▂ |
| MAGUIRE EA, 1998, J COGNITIVE NEUROSCI, V10, P61, [DOI](http://dx.doi.org/10.1162%2F089892998563789) | 1998 | 8.3683 | **1998** | 2005 | ▂▂▂▂▂▂▂▂▃▃▃▃▃▃▃▃▂▂▂▂▂▂▂▂▂▂▂ |
| HAUBNER M, 1997, IEEE TRANS INF TECHNOL BIOMED, V1, P61, [DOI](http://dx.doi.org/10.1109%2F4233.594047) | 1997 | 8.1139 | **1998** | 2004 | ▂▂▂▂▂▂▂▂▃▃▃▃▃▃▃▂▂▂▂▂▂▂▂▂▂▂▂ |
| SHERIDAN TB, 1996, PRESENCE-TELEOP VIRT, V5, P241 | 1996 | 7.5718 | **1998** | 2002 | ▂▂▂▂▂▂▂▂▃▃▃▃▃▂▂▂▂▂▂▂▂▂▂▂▂▂▂ |
| INFORMATION TECHNOLOGY, 1997, 147721 ISOIEC, V, P | 1997 | 8.8538 | **1998** | 2004 | ▂▂▂▂▂▂▂▂▃▃▃▃▃▃▃▂▂▂▂▂▂▂▂▂▂▂▂ |
| ANDREAE MH, 1996, BRIT MED J, V312, P4 | 1996 | 3.605 | **1998** | 2003 | ▂▂▂▂▂▂▂▂▃▃▃▃▃▃▂▂▂▂▂▂▂▂▂▂▂▂▂ |
| KRUGER W, 1995, COMPUTER, V28, P42, [DOI](http://dx.doi.org/10.1109%2F2.391040) | 1995 | 8.8175 | **1998** | 2002 | ▂▂▂▂▂▂▂▂▃▃▃▃▃▂▂▂▂▂▂▂▂▂▂▂▂▂▂ |
| AGUIRRE GK, 1997, J NEUROSCI, V17, P2512 | 1997 | 7.636 | **1998** | 2004 | ▂▂▂▂▂▂▂▂▃▃▃▃▃▃▃▂▂▂▂▂▂▂▂▂▂▂▂ |
| HUBBARD PM, 1995, IEEE T VIS COMPUT GR, V1, P218, [DOI](http://dx.doi.org/10.1109%2F2945.466717) | 1995 | 5.6668 | **1998** | 2003 | ▂▂▂▂▂▂▂▂▃▃▃▃▃▃▂▂▂▂▂▂▂▂▂▂▂▂▂ |
| KUHNAPFEL UG, 1997, MINIM INVASIV THER, V6, P122, [DOI](http://dx.doi.org/10.3109%2F13645709709152715) | 1997 | 6.6804 | **1998** | 2004 | ▂▂▂▂▂▂▂▂▃▃▃▃▃▃▃▂▂▂▂▂▂▂▂▂▂▂▂ |
| CHU CCP, 1997, COMPUT AIDED DESIGN, V29, P709, [DOI](http://dx.doi.org/10.1016%2FS0010-4485%2897%2900021-3) | 1997 | 6.9021 | **1998** | 2005 | ▂▂▂▂▂▂▂▂▃▃▃▃▃▃▃▃▂▂▂▂▂▂▂▂▂▂▂ |
| PALMER IJ, 1995, COMPUT GRAPH FORUM, V14, P105, [DOI](http://dx.doi.org/10.1111%2F1467-8659.1420105) | 1995 | 4.6357 | **1998** | 2003 | ▂▂▂▂▂▂▂▂▃▃▃▃▃▃▂▂▂▂▂▂▂▂▂▂▂▂▂ |
| MAGUIRE EA, 1998, SCIENCE, V280, P921, [DOI](http://dx.doi.org/10.1126%2FSCIENCE.280.5365.921) | 1998 | 15.2756 | **1998** | 2006 | ▂▂▂▂▂▂▂▂▃▃▃▃▃▃▃▃▃▂▂▂▂▂▂▂▂▂▂ |
| AZUMA RT, 1997, PRESENCE-TELEOP VIRT, V6, P355 | 1997 | 12.0903 | **1998** | 2005 | ▂▂▂▂▂▂▂▂▃▃▃▃▃▃▃▃▂▂▂▂▂▂▂▂▂▂▂ |
| SUMMERS RM, 1996, RADIOLOGY, V200, P857 | 1996 | 4.2928 | **1998** | 2004 | ▂▂▂▂▂▂▂▂▃▃▃▃▃▃▃▂▂▂▂▂▂▂▂▂▂▂▂ |
| GREENLEAF W, 1996, IEEE ENG MED BIOL, V15, P23, [DOI](http://dx.doi.org/10.1109%2F51.486714) | 1996 | 4.6596 | **1998** | 2001 | ▂▂▂▂▂▂▂▂▃▃▃▃▂▂▂▂▂▂▂▂▂▂▂▂▂▂▂ |
| ROSE FD, 1996, CURR OPIN NEUROL, V9, P461, [DOI](http://dx.doi.org/10.1097%2F00019052-199612000-00012) | 1996 | 9.5481 | **1998** | 2004 | ▂▂▂▂▂▂▂▂▃▃▃▃▃▃▃▂▂▂▂▂▂▂▂▂▂▂▂ |
| BIOCCA F, 1995, COMMUNICATION AGE VI, V, P57 | 1995 | 6.0596 | **1998** | 2002 | ▂▂▂▂▂▂▂▂▃▃▃▃▃▂▂▂▂▂▂▂▂▂▂▂▂▂▂ |
| KALAWSKY RS, 1994, SCI VIRTUAL REALITY, V, P | 1994 | 3.8548 | **1998** | 2002 | ▂▂▂▂▂▂▂▂▃▃▃▃▃▂▂▂▂▂▂▂▂▂▂▂▂▂▂ |
| CAREY R, 1997, ANNOTATED VRML 2 0 R, V, P | 1997 | 9.9273 | **1998** | 2005 | ▂▂▂▂▂▂▂▂▃▃▃▃▃▃▃▃▂▂▂▂▂▂▂▂▂▂▂ |
| COHEN J D, 1995, PROCEEDINGS 1995 SYMPOSIUM ON INTERACTIVE 3D GRAPHICS, V, P, [DOI](http://dx.doi.org/10.1145%2F199404.199437) | 1995 | 8.2459 | **1998** | 2003 | ▂▂▂▂▂▂▂▂▃▃▃▃▃▃▂▂▂▂▂▂▂▂▂▂▂▂▂ |
| BASDOGAN C, 1998, P MED MEETS VIRT REA, V, P385 | 1998 | 4.4059 | **1998** | 2002 | ▂▂▂▂▂▂▂▂▃▃▃▃▃▂▂▂▂▂▂▂▂▂▂▂▂▂▂ |
| STANNEY KM, 1998, PRESENCE-TELEOP VIRT, V7, P327, [DOI](http://dx.doi.org/10.1162%2F105474698565767) | 1998 | 7.4586 | **1999** | 2006 | ▂▂▂▂▂▂▂▂▂▃▃▃▃▃▃▃▃▂▂▂▂▂▂▂▂▂▂ |
| NICHOLS S, 1997, PRESENCE-TELEOP VIRT, V6, P667 | 1997 | 3.5276 | **1999** | 2001 | ▂▂▂▂▂▂▂▂▂▃▃▃▂▂▂▂▂▂▂▂▂▂▂▂▂▂▂ |
| JOHNSON WL, 1998, PRESENCE-TELEOP VIRT, V7, P523, [DOI](http://dx.doi.org/10.1162%2F105474698565929) | 1998 | 6.4299 | **1999** | 2003 | ▂▂▂▂▂▂▂▂▂▃▃▃▃▃▂▂▂▂▂▂▂▂▂▂▂▂▂ |
| AGRAWALA M, 1997, ANN C SERIES, V, P327, [DOI](http://dx.doi.org/10.1145%2F258734.258875) | 1997 | 4.2211 | **1999** | 2001 | ▂▂▂▂▂▂▂▂▂▃▃▃▂▂▂▂▂▂▂▂▂▂▂▂▂▂▂ |
| BIOCCA F, 1997, J COMPUTER MEDIATED, V3, P | 1997 | 5.8747 | **1999** | 2005 | ▂▂▂▂▂▂▂▂▂▃▃▃▃▃▃▃▂▂▂▂▂▂▂▂▂▂▂ |
| REZNICK RK, 1993, AM J SURG, V165, P358, [DOI](http://dx.doi.org/10.1016%2FS0002-9610%2805%2980843-8) | 1993 | 7.2392 | **1999** | 2001 | ▂▂▂▂▂▂▂▂▂▃▃▃▂▂▂▂▂▂▂▂▂▂▂▂▂▂▂ |
| PIEGL L, 1997, NURBS BOOK, V, P | 1997 | 6.4708 | **1999** | 2004 | ▂▂▂▂▂▂▂▂▂▃▃▃▃▃▃▂▂▂▂▂▂▂▂▂▂▂▂ |
| CRUZ-NEIRA C, 1992, COMMUN ACM, V35, P65 | 1992 | 5.1051 | **1999** | 2000 | ▂▂▂▂▂▂▂▂▂▃▃▂▂▂▂▂▂▂▂▂▂▂▂▂▂▂▂ |
| [ANONYMOUS], 1998, CYBERPSYCHOL BEHAV, V, P, [DOI](http://dx.doi.org/10.1089%2FCPB.1998.1.79) | 1998 | 4.7081 | **1999** | 2006 | ▂▂▂▂▂▂▂▂▂▃▃▃▃▃▃▃▃▂▂▂▂▂▂▂▂▂▂ |
| RIVA G, 1997, VIRTUAL REALITY NEUR, V, P | 1997 | 7.7244 | **1999** | 2003 | ▂▂▂▂▂▂▂▂▂▃▃▃▃▃▂▂▂▂▂▂▂▂▂▂▂▂▂ |
| JAYARAM S, 1997, COMPUT AIDED DESIGN, V29, P575, [DOI](http://dx.doi.org/10.1016%2FS0010-4485%2896%2900094-2) | 1997 | 10.402 | **1999** | 2005 | ▂▂▂▂▂▂▂▂▂▃▃▃▃▃▃▃▂▂▂▂▂▂▂▂▂▂▂ |
| TAFFINDER NJ, 1998, BRIT J SURG, V85, P75 | 1998 | 5.8747 | **1999** | 2005 | ▂▂▂▂▂▂▂▂▂▃▃▃▃▃▃▃▂▂▂▂▂▂▂▂▂▂▂ |
| CHRISTIANSEN C, 1998, ARCH PHYS MED REHAB, V79, P888, [DOI](http://dx.doi.org/10.1016%2FS0003-9993%2898%2990083-1) | 1998 | 12.6393 | **1999** | 2005 | ▂▂▂▂▂▂▂▂▂▃▃▃▃▃▃▃▂▂▂▂▂▂▂▂▂▂▂ |
| DANI TH, 1997, COMPUT AIDED DESIGN, V29, P555, [DOI](http://dx.doi.org/10.1016%2FS0010-4485%2896%2900091-7) | 1997 | 8.8016 | **1999** | 2005 | ▂▂▂▂▂▂▂▂▂▃▃▃▃▃▃▃▂▂▂▂▂▂▂▂▂▂▂ |
| MOLINARI E, 1998, VIRTUAL ENV CLIN PSY, V, P | 1998 | 11.067 | **1999** | 2005 | ▂▂▂▂▂▂▂▂▂▃▃▃▃▃▃▃▂▂▂▂▂▂▂▂▂▂▂ |
| LOMBARD M, 1997, J COMPUTER MEDIATED, V3, P | 1997 | 8.9632 | **1999** | 2004 | ▂▂▂▂▂▂▂▂▂▃▃▃▃▃▃▂▂▂▂▂▂▂▂▂▂▂▂ |
| GLANTZ K, 1996, PSYCHOTHERAPY, V33, P464, [DOI](http://dx.doi.org/10.1037%2F0033-3204.33.3.464) | 1996 | 7.5027 | **1999** | 2003 | ▂▂▂▂▂▂▂▂▂▃▃▃▃▃▂▂▂▂▂▂▂▂▂▂▂▂▂ |
| NORTH MM, 1997, AM J PSYCHIAT, V154, P130 | 1997 | 8.5902 | **1999** | 2005 | ▂▂▂▂▂▂▂▂▂▃▃▃▃▃▃▃▂▂▂▂▂▂▂▂▂▂▂ |
| PREECE J, 1994, HUMAN COMPUTER INTER, V, P | 1994 | 5.1426 | **1999** | 2002 | ▂▂▂▂▂▂▂▂▂▃▃▃▃▂▂▂▂▂▂▂▂▂▂▂▂▂▂ |
| FREUND E, 1994, P IEEE RSJ GI INT RO, V, P | 1994 | 6.2864 | **1999** | 2002 | ▂▂▂▂▂▂▂▂▂▃▃▃▃▂▂▂▂▂▂▂▂▂▂▂▂▂▂ |
| GUPTA R, 1997, PRESENCE-TELEOP VIRT, V6, P318 | 1997 | 5.4223 | **1999** | 2005 | ▂▂▂▂▂▂▂▂▂▃▃▃▃▃▃▃▂▂▂▂▂▂▂▂▂▂▂ |
| MIRTICH B V, 1996, THESIS U CALIFORNIA, V, P | 1996 | 4.8245 | **1999** | 2001 | ▂▂▂▂▂▂▂▂▂▃▃▃▂▂▂▂▂▂▂▂▂▂▂▂▂▂▂ |
| WILSON MS, 1997, ANN ROY COLL SURG, V79, P403 | 1997 | 14.4536 | **1999** | 2004 | ▂▂▂▂▂▂▂▂▂▃▃▃▃▃▃▂▂▂▂▂▂▂▂▂▂▂▂ |
| FREUND E, 1999, IEEE T ROBOTIC AUTOM, V15, P411, [DOI](http://dx.doi.org/10.1109%2F70.768175) | 1999 | 9.3403 | **2000** | 2005 | ▂▂▂▂▂▂▂▂▂▂▃▃▃▃▃▃▂▂▂▂▂▂▂▂▂▂▂ |
| REGENBRECHT HT, 1998, INT J HUM-COMPUT INT, V10, P233, [DOI](http://dx.doi.org/10.1207%2FS15327590IJHC1003_2) | 1998 | 8.7837 | **2000** | 2006 | ▂▂▂▂▂▂▂▂▂▂▃▃▃▃▃▃▃▂▂▂▂▂▂▂▂▂▂ |
| GREALY MA, 1999, ARCH PHYS MED REHAB, V80, P661, [DOI](http://dx.doi.org/10.1016%2FS0003-9993%2899%2990169-7) | 1999 | 9.3403 | **2000** | 2005 | ▂▂▂▂▂▂▂▂▂▂▃▃▃▃▃▃▂▂▂▂▂▂▂▂▂▂▂ |
| RIZZO A A, 1997, VIRTUAL REALITY NEUR, V, P123 | 1997 | 8.2921 | **2000** | 2003 | ▂▂▂▂▂▂▂▂▂▂▃▃▃▃▂▂▂▂▂▂▂▂▂▂▂▂▂ |
| BRIDGES M, 1999, AM J SURG, V177, P28, [DOI](http://dx.doi.org/10.1016%2FS0002-9610%2898%2900289-X) | 1999 | 19.3091 | **2000** | 2007 | ▂▂▂▂▂▂▂▂▂▂▃▃▃▃▃▃▃▃▂▂▂▂▂▂▂▂▂ |
| KRUMMEL TM, 1998, ANN SURG, V228, P635, [DOI](http://dx.doi.org/10.1097%2F00000658-199811000-00002) | 1998 | 17.1767 | **2000** | 2006 | ▂▂▂▂▂▂▂▂▂▂▃▃▃▃▃▃▃▂▂▂▂▂▂▂▂▂▂ |
| CARLIN AS, 1997, BEHAV RES THER, V35, P153, [DOI](http://dx.doi.org/10.1016%2FS0005-7967%2896%2900085-X) | 1997 | 26.9378 | **2000** | 2005 | ▂▂▂▂▂▂▂▂▂▂▃▃▃▃▃▃▂▂▂▂▂▂▂▂▂▂▂ |
| BLACKWELL M, 1998, CLIN ORTHOP RELAT R, V, P111 | 1998 | 3.8187 | **2000** | 2005 | ▂▂▂▂▂▂▂▂▂▂▃▃▃▃▃▃▂▂▂▂▂▂▂▂▂▂▂ |
| TAFFINDER N, 1998, ST HEAL T, V50, P124 | 1998 | 30.9569 | **2000** | 2006 | ▂▂▂▂▂▂▂▂▂▂▃▃▃▃▃▃▃▂▂▂▂▂▂▂▂▂▂ |
| ISSENBERG SB, 1999, JAMA-J AM MED ASSOC, V282, P861, [DOI](http://dx.doi.org/10.1001%2FJAMA.282.9.861) | 1999 | 14.6294 | **2000** | 2007 | ▂▂▂▂▂▂▂▂▂▂▃▃▃▃▃▃▃▃▂▂▂▂▂▂▂▂▂ |
| OTOOLE RV, 1999, J AM COLL SURGEONS, V189, P114, [DOI](http://dx.doi.org/10.1016%2FS1072-7515%2899%2900076-9) | 1999 | 20.5578 | **2000** | 2006 | ▂▂▂▂▂▂▂▂▂▂▃▃▃▃▃▃▃▂▂▂▂▂▂▂▂▂▂ |
| ROSE FD, 1999, DISABIL REHABIL, V21, P548 | 1999 | 8.3833 | **2000** | 2005 | ▂▂▂▂▂▂▂▂▂▂▃▃▃▃▃▃▂▂▂▂▂▂▂▂▂▂▂ |
| DEROSSIS AM, 1998, AM J SURG, V175, P482, [DOI](http://dx.doi.org/10.1016%2FS0002-9610%2898%2900080-4) | 1998 | 18.0174 | **2000** | 2006 | ▂▂▂▂▂▂▂▂▂▂▃▃▃▃▃▃▃▂▂▂▂▂▂▂▂▂▂ |
| TAFFINDER NJ, 1998, LANCET, V352, P1191, [DOI](http://dx.doi.org/10.1016%2FS0140-6736%2898%2900034-8) | 1998 | 9.6218 | **2000** | 2006 | ▂▂▂▂▂▂▂▂▂▂▃▃▃▃▃▃▃▂▂▂▂▂▂▂▂▂▂ |
| MARTIN JA, 1997, BRIT J SURG, V84, P273, [DOI](http://dx.doi.org/10.1002%2FBJS.1800840237) | 1997 | 16.7434 | **2000** | 2005 | ▂▂▂▂▂▂▂▂▂▂▃▃▃▃▃▃▂▂▂▂▂▂▂▂▂▂▂ |
| GALLAGHER AG, 1998, ENDOSCOPY, V30, P617, [DOI](http://dx.doi.org/10.1055%2FS-2007-1001366) | 1998 | 12.5572 | **2000** | 2006 | ▂▂▂▂▂▂▂▂▂▂▃▃▃▃▃▃▃▂▂▂▂▂▂▂▂▂▂ |
| DEROSSIS AM, 1998, SURG ENDOSC-ULTRAS, V12, P1117, [DOI](http://dx.doi.org/10.1007%2FS004649900796) | 1998 | 12.956 | **2000** | 2005 | ▂▂▂▂▂▂▂▂▂▂▃▃▃▃▃▃▂▂▂▂▂▂▂▂▂▂▂ |
| BROOKS FP, 1999, IEEE COMPUT GRAPH, V19, P16, [DOI](http://dx.doi.org/10.1109%2F38.799723) | 1999 | 9.4902 | **2000** | 2006 | ▂▂▂▂▂▂▂▂▂▂▃▃▃▃▃▃▃▂▂▂▂▂▂▂▂▂▂ |
| RIZZO AA, 1998, VIRTUAL ENV CLIN PSY, V, P21 | 1998 | 5.4341 | **2000** | 2006 | ▂▂▂▂▂▂▂▂▂▂▃▃▃▃▃▃▃▂▂▂▂▂▂▂▂▂▂ |
| MACMILLAN AIM, 1999, AM J SURG, V177, P274, [DOI](http://dx.doi.org/10.1016%2FS0002-9610%2899%2900016-1) | 1999 | 7.8381 | **2000** | 2007 | ▂▂▂▂▂▂▂▂▂▂▃▃▃▃▃▃▃▃▂▂▂▂▂▂▂▂▂ |
| BROOKS BM, 1999, NEUROPSYCHOL REHABIL, V9, P63, [DOI](http://dx.doi.org/10.1080%2F713755589) | 1999 | 8.9273 | **2000** | 2006 | ▂▂▂▂▂▂▂▂▂▂▃▃▃▃▃▃▃▂▂▂▂▂▂▂▂▂▂ |
| VINCELLI F, 1999, CYBERPSYCHOL BEHAV, V2, P241, [DOI](http://dx.doi.org/10.1089%2FCPB.1999.2.241) | 1999 | 8.8618 | **2000** | 2005 | ▂▂▂▂▂▂▂▂▂▂▃▃▃▃▃▃▂▂▂▂▂▂▂▂▂▂▂ |
| ROSSER JC, 1997, ARCH SURG-CHICAGO, V132, P200 | 1997 | 15.089 | **2000** | 2005 | ▂▂▂▂▂▂▂▂▂▂▃▃▃▃▃▃▂▂▂▂▂▂▂▂▂▂▂ |
| BURDEA G, 1999, IEEE T BIO-MED ENG, V46, P1253, [DOI](http://dx.doi.org/10.1109%2F10.790503) | 1999 | 8.1951 | **2000** | 2007 | ▂▂▂▂▂▂▂▂▂▂▃▃▃▃▃▃▃▃▂▂▂▂▂▂▂▂▂ |
| GALLAGHER AG, 1999, ENDOSCOPY, V31, P310 | 1999 | 22.8779 | **2000** | 2007 | ▂▂▂▂▂▂▂▂▂▂▃▃▃▃▃▃▃▃▂▂▂▂▂▂▂▂▂ |
| SINGHAL S, 1999, NETWORKED VIRTUAL EN, V, P | 1999 | 14.6294 | **2000** | 2007 | ▂▂▂▂▂▂▂▂▂▂▃▃▃▃▃▃▃▃▂▂▂▂▂▂▂▂▂ |
| BOTELLA C, 1998, BEHAV RES THER, V36, P239, [DOI](http://dx.doi.org/10.1016%2FS0005-7967%2897%2910006-7) | 1998 | 19.2791 | **2000** | 2006 | ▂▂▂▂▂▂▂▂▂▂▃▃▃▃▃▃▃▂▂▂▂▂▂▂▂▂▂ |
| HOFFMAN HG, 2000, PAIN, V85, P305, [DOI](http://dx.doi.org/10.1016%2FS0304-3959%2899%2900275-4) | 2000 | 16.0411 | **2001** | 2008 | ▂▂▂▂▂▂▂▂▂▂▂▃▃▃▃▃▃▃▃▂▂▂▂▂▂▂▂ |
| GORMAN PJ, 1999, ARCH SURG-CHICAGO, V134, P1203, [DOI](http://dx.doi.org/10.1001%2FARCHSURG.134.11.1203) | 1999 | 10.5099 | **2001** | 2007 | ▂▂▂▂▂▂▂▂▂▂▂▃▃▃▃▃▃▃▂▂▂▂▂▂▂▂▂ |
| KOCKRO RA, 2000, NEUROSURGERY, V46, P118 | 2000 | 9.1724 | **2001** | 2008 | ▂▂▂▂▂▂▂▂▂▂▂▃▃▃▃▃▃▃▃▂▂▂▂▂▂▂▂ |
| COTIN S, 2000, VISUAL COMPUT, V16, P437, [DOI](http://dx.doi.org/10.1007%2FPL00007215) | 2000 | 11.4719 | **2001** | 2008 | ▂▂▂▂▂▂▂▂▂▂▂▃▃▃▃▃▃▃▃▂▂▂▂▂▂▂▂ |
| BOHBOT VD, 1998, NEUROPSYCHOLOGIA, V36, P1217, [DOI](http://dx.doi.org/10.1016%2FS0028-3932%2897%2900161-9) | 1998 | 5.5765 | **2001** | 2006 | ▂▂▂▂▂▂▂▂▂▂▂▃▃▃▃▃▃▂▂▂▂▂▂▂▂▂▂ |
| ROTHBAUM BO, 1999, J TRAUMA STRESS, V12, P263, [DOI](http://dx.doi.org/10.1023%2FA%3A1024772308758) | 1999 | 13.5293 | **2001** | 2007 | ▂▂▂▂▂▂▂▂▂▂▂▃▃▃▃▃▃▃▂▂▂▂▂▂▂▂▂ |
| OST LG, 1997, BEHAV RES THER, V35, P987, [DOI](http://dx.doi.org/10.1016%2FS0005-7967%2897%2900077-6) | 1997 | 6.0881 | **2001** | 2005 | ▂▂▂▂▂▂▂▂▂▂▂▃▃▃▃▃▂▂▂▂▂▂▂▂▂▂▂ |
| COTIN S, 1999, IEEE T VIS COMPUT GR, V5, P62, [DOI](http://dx.doi.org/10.1109%2F2945.764872) | 1999 | 17.4087 | **2001** | 2007 | ▂▂▂▂▂▂▂▂▂▂▂▃▃▃▃▃▃▃▂▂▂▂▂▂▂▂▂ |
| RIZZO AA, 2000, CYBERPSYCHOL BEHAV, V3, P483, [DOI](http://dx.doi.org/10.1089%2F10949310050078940) | 2000 | 8.8442 | **2001** | 2008 | ▂▂▂▂▂▂▂▂▂▂▂▃▃▃▃▃▃▃▃▂▂▂▂▂▂▂▂ |
| HOFFMAN HG, 2000, CLIN J PAIN, V16, P244, [DOI](http://dx.doi.org/10.1097%2F00002508-200009000-00010) | 2000 | 16.4055 | **2001** | 2007 | ▂▂▂▂▂▂▂▂▂▂▂▃▃▃▃▃▃▃▂▂▂▂▂▂▂▂▂ |
| ROTHBAUM BO, 2000, J CONSULT CLIN PSYCH, V68, P1020, [DOI](http://dx.doi.org/10.1037%2F0022-006X.68.6.1020) | 2000 | 21.6492 | **2001** | 2008 | ▂▂▂▂▂▂▂▂▂▂▂▃▃▃▃▃▃▃▃▂▂▂▂▂▂▂▂ |
| KUHNAPFEL U, 2000, COMPUT GRAPH-UK, V24, P671, [DOI](http://dx.doi.org/10.1016%2FS0097-8493%2800%2900070-4) | 2000 | 16.4077 | **2001** | 2008 | ▂▂▂▂▂▂▂▂▂▂▂▃▃▃▃▃▃▃▃▂▂▂▂▂▂▂▂ |
| HONJO T, 2001, LANDSCAPE URBAN PLAN, V55, P175, [DOI](http://dx.doi.org/10.1016%2FS0169-2046%2801%2900152-9) | 2001 | 4.6301 | **2001** | 2007 | ▂▂▂▂▂▂▂▂▂▂▂▃▃▃▃▃▃▃▂▂▂▂▂▂▂▂▂ |
| WIEDERHOLD B K, 1998, CYBERPSYCHOL BEHAV, V1, P97, [DOI](http://dx.doi.org/10.1089%2FCPB.1998.1.97) | 1998 | 6.2705 | **2001** | 2006 | ▂▂▂▂▂▂▂▂▂▂▂▃▃▃▃▃▃▂▂▂▂▂▂▂▂▂▂ |
| ADAMS RJ, 1999, IEEE T ROBOTIC AUTOM, V15, P465, [DOI](http://dx.doi.org/10.1109%2F70.768179) | 1999 | 5.7891 | **2001** | 2007 | ▂▂▂▂▂▂▂▂▂▂▂▃▃▃▃▃▃▃▂▂▂▂▂▂▂▂▂ |
| CHAUDHRY A, 1999, ANN ROY COLL SURG, V81, P281 | 1999 | 20.128 | **2001** | 2007 | ▂▂▂▂▂▂▂▂▂▂▂▃▃▃▃▃▃▃▂▂▂▂▂▂▂▂▂ |
| PRYSTOWSKY JB, 1999, AM J SURG, V177, P171, [DOI](http://dx.doi.org/10.1016%2FS0002-9610%2898%2900328-6) | 1999 | 11.5734 | **2001** | 2006 | ▂▂▂▂▂▂▂▂▂▂▂▃▃▃▃▃▃▂▂▂▂▂▂▂▂▂▂ |
| REZNICK R, 1997, AM J SURG, V173, P226, [DOI](http://dx.doi.org/10.1016%2FS0002-9610%2897%2989597-9) | 1997 | 14.5476 | **2001** | 2005 | ▂▂▂▂▂▂▂▂▂▂▂▃▃▃▃▃▂▂▂▂▂▂▂▂▂▂▂ |
| GRON G, 2000, NAT NEUROSCI, V3, P404 | 2000 | 8.5295 | **2001** | 2005 | ▂▂▂▂▂▂▂▂▂▂▂▃▃▃▃▃▂▂▂▂▂▂▂▂▂▂▂ |
| DARZI A, 1999, BRIT MED J, V318, P887 | 1999 | 13.3718 | **2001** | 2006 | ▂▂▂▂▂▂▂▂▂▂▂▃▃▃▃▃▃▂▂▂▂▂▂▂▂▂▂ |
| WITMER BG, 1998, PRESENCE-TELEOP VIRT, V7, P225, [DOI](http://dx.doi.org/10.1162%2F105474698565686) | 1998 | 30.7227 | **2001** | 2006 | ▂▂▂▂▂▂▂▂▂▂▂▃▃▃▃▃▃▂▂▂▂▂▂▂▂▂▂ |
| HOLDEN M, 1999, NEUROLOGY REPORT, V23, P57 | 1999 | 11.5919 | **2001** | 2007 | ▂▂▂▂▂▂▂▂▂▂▂▃▃▃▃▃▃▃▂▂▂▂▂▂▂▂▂ |
| JORDAN JA, 2000, AM J SURG, V180, P208, [DOI](http://dx.doi.org/10.1016%2FS0002-9610%2800%2900469-4) | 2000 | 9.8292 | **2001** | 2008 | ▂▂▂▂▂▂▂▂▂▂▂▃▃▃▃▃▃▃▃▂▂▂▂▂▂▂▂ |
| SCHULTHEIS MT, 2001, REHABIL PSYCHOL, V46, P296, [DOI](http://dx.doi.org/10.1037%2F0090-5550.46.3.296) | 2001 | 13.7948 | **2002** | 2009 | ▂▂▂▂▂▂▂▂▂▂▂▂▃▃▃▃▃▃▃▃▂▂▂▂▂▂▂ |
| ANASTAKIS DJ, 1999, AM J SURG, V177, P167, [DOI](http://dx.doi.org/10.1016%2FS0002-9610%2898%2900327-4) | 1999 | 15.0612 | **2002** | 2007 | ▂▂▂▂▂▂▂▂▂▂▂▂▃▃▃▃▃▃▂▂▂▂▂▂▂▂▂ |
| SZEKELY G, 2000, PRESENCE-TELEOP VIRT, V9, P310, [DOI](http://dx.doi.org/10.1162%2F105474600566817) | 2000 | 5.798 | **2002** | 2008 | ▂▂▂▂▂▂▂▂▂▂▂▂▃▃▃▃▃▃▃▂▂▂▂▂▂▂▂ |
| MCCLOY R, 2001, BRIT MED J, V323, P912, [DOI](http://dx.doi.org/10.1136%2FBMJ.323.7318.912) | 2001 | 15.0193 | **2002** | 2008 | ▂▂▂▂▂▂▂▂▂▂▂▂▃▃▃▃▃▃▃▂▂▂▂▂▂▂▂ |
| GORMAN PJ, 2000, AM J SURG, V180, P353, [DOI](http://dx.doi.org/10.1016%2FS0002-9610%2800%2900514-6) | 2000 | 5.894 | **2002** | 2007 | ▂▂▂▂▂▂▂▂▂▂▂▂▃▃▃▃▃▃▂▂▂▂▂▂▂▂▂ |
| FURUSHO J, 1999, INT J MOD PHYS B, V13, P2151, [DOI](http://dx.doi.org/10.1142%2FS0217979299002253) | 1999 | 5.894 | **2002** | 2007 | ▂▂▂▂▂▂▂▂▂▂▂▂▃▃▃▃▃▃▂▂▂▂▂▂▂▂▂ |
| BOTELLA C, 2000, BEHAV THER, V31, P583, [DOI](http://dx.doi.org/10.1016%2FS0005-7894%2800%2980032-5) | 2000 | 8.4506 | **2002** | 2007 | ▂▂▂▂▂▂▂▂▂▂▂▂▃▃▃▃▃▃▂▂▂▂▂▂▂▂▂ |
| GRANTCHAROV TP, 2001, SURG ENDOSC-ULTRAS, V15, P242, [DOI](http://dx.doi.org/10.1007%2FS004640090008) | 2001 | 14.0958 | **2002** | 2009 | ▂▂▂▂▂▂▂▂▂▂▂▂▃▃▃▃▃▃▃▃▂▂▂▂▂▂▂ |
| FRIED GM, 1999, SURG ENDOSC-ULTRAS, V13, P1077, [DOI](http://dx.doi.org/10.1007%2FS004649901176) | 1999 | 11.3788 | **2002** | 2007 | ▂▂▂▂▂▂▂▂▂▂▂▂▃▃▃▃▃▃▂▂▂▂▂▂▂▂▂ |
| EMMELKAMP PMG, 2001, CYBERPSYCHOL BEHAV, V4, P335, [DOI](http://dx.doi.org/10.1089%2F109493101300210222) | 2001 | 11.0881 | **2002** | 2009 | ▂▂▂▂▂▂▂▂▂▂▂▂▃▃▃▃▃▃▃▃▂▂▂▂▂▂▂ |
| JACK D, 2001, IEEE T NEUR SYS REH, V9, P308, [DOI](http://dx.doi.org/10.1109%2F7333.948460) | 2001 | 20.1255 | **2002** | 2009 | ▂▂▂▂▂▂▂▂▂▂▂▂▃▃▃▃▃▃▃▃▂▂▂▂▂▂▂ |
| MARESCAUX J, 2001, NATURE, V413, P379, [DOI](http://dx.doi.org/10.1038%2F35096636) | 2001 | 6.1304 | **2002** | 2006 | ▂▂▂▂▂▂▂▂▂▂▂▂▃▃▃▃▃▂▂▂▂▂▂▂▂▂▂ |
| CHEN F, 2000, OPT ENG, V39, P10, [DOI](http://dx.doi.org/10.1117%2F1.602438) | 2000 | 4.5996 | **2002** | 2004 | ▂▂▂▂▂▂▂▂▂▂▂▂▃▃▃▂▂▂▂▂▂▂▂▂▂▂▂ |
| BALLARO A, 1999, J UROLOGY, V162, P1633, [DOI](http://dx.doi.org/10.1016%2FS0022-5347%2805%2968184-0) | 1999 | 4.6299 | **2002** | 2007 | ▂▂▂▂▂▂▂▂▂▂▂▂▃▃▃▃▃▃▂▂▂▂▂▂▂▂▂ |
| HALUCK RS, 2000, ARCH SURG-CHICAGO, V135, P786, [DOI](http://dx.doi.org/10.1001%2FARCHSURG.135.7.786) | 2000 | 17.5053 | **2002** | 2008 | ▂▂▂▂▂▂▂▂▂▂▂▂▃▃▃▃▃▃▃▂▂▂▂▂▂▂▂ |
| BOWMAN DA, 2001, PRESENCE-TELEOP VIRT, V10, P96, [DOI](http://dx.doi.org/10.1162%2F105474601750182342) | 2001 | 7.2527 | **2003** | 2009 | ▂▂▂▂▂▂▂▂▂▂▂▂▂▃▃▃▃▃▃▃▂▂▂▂▂▂▂ |
| SCOTT DJ, 2000, J AM COLL SURGEONS, V191, P272, [DOI](http://dx.doi.org/10.1016%2FS1072-7515%2800%2900339-2) | 2000 | 33.3868 | **2003** | 2008 | ▂▂▂▂▂▂▂▂▂▂▂▂▂▃▃▃▃▃▃▂▂▂▂▂▂▂▂ |
| ROTHBAUM BO, 2002, J CONSULT CLIN PSYCH, V70, P428, [DOI](http://dx.doi.org/10.1037%2F%2F0022-006X.70.2.428) | 2002 | 12.7168 | **2003** | 2010 | ▂▂▂▂▂▂▂▂▂▂▂▂▂▃▃▃▃▃▃▃▃▂▂▂▂▂▂ |
| GALLAGHER AG, 2001, WORLD J SURG, V25, P1478, [DOI](http://dx.doi.org/10.1007%2FS00268-001-0133-1) | 2001 | 22.5085 | **2003** | 2009 | ▂▂▂▂▂▂▂▂▂▂▂▂▂▃▃▃▃▃▃▃▂▂▂▂▂▂▂ |
| SCOTT DJ, 2001, AM J SURG, V182, P137, [DOI](http://dx.doi.org/10.1016%2FS0002-9610%2801%2900669-9) | 2001 | 7.1905 | **2003** | 2008 | ▂▂▂▂▂▂▂▂▂▂▂▂▂▃▃▃▃▃▃▂▂▂▂▂▂▂▂ |
| PAISLEY AM, 2001, BRIT J SURG, V88, P1525, [DOI](http://dx.doi.org/10.1046%2FJ.0007-1323.2001.01880.X) | 2001 | 8.6108 | **2003** | 2009 | ▂▂▂▂▂▂▂▂▂▂▂▂▂▃▃▃▃▃▃▃▂▂▂▂▂▂▂ |
| EMMELKAMP PMG, 2002, BEHAV RES THER, V40, P509, [DOI](http://dx.doi.org/10.1016%2FS0005-7967%2801%2900023-7) | 2002 | 18.8307 | **2003** | 2010 | ▂▂▂▂▂▂▂▂▂▂▂▂▂▃▃▃▃▃▃▃▃▂▂▂▂▂▂ |
| DATTA V, 2001, J AM COLL SURGEONS, V193, P479, [DOI](http://dx.doi.org/10.1016%2FS1072-7515%2801%2901041-9) | 2001 | 14.5628 | **2003** | 2009 | ▂▂▂▂▂▂▂▂▂▂▂▂▂▃▃▃▃▃▃▃▂▂▂▂▂▂▂ |
| GUTHOLD M, 2000, IEEE-ASME T MECH, V5, P189, [DOI](http://dx.doi.org/10.1109%2F3516.847092) | 2000 | 3.9393 | **2003** | 2008 | ▂▂▂▂▂▂▂▂▂▂▂▂▂▃▃▃▃▃▃▂▂▂▂▂▂▂▂ |
| BASDOGAN C, 2001, IEEE-ASME T MECH, V6, P269, [DOI](http://dx.doi.org/10.1109%2F3516.951365) | 2001 | 7.9472 | **2003** | 2009 | ▂▂▂▂▂▂▂▂▂▂▂▂▂▃▃▃▃▃▃▃▂▂▂▂▂▂▂ |
| COLT HG, 2001, CHEST, V120, P1333, [DOI](http://dx.doi.org/10.1378%2FCHEST.120.4.1333) | 2001 | 9.908 | **2003** | 2009 | ▂▂▂▂▂▂▂▂▂▂▂▂▂▃▃▃▃▃▃▃▂▂▂▂▂▂▂ |
| WIEDERHOLD BK, 2002, IEEE T INF TECHNOL B, V6, P218, [DOI](http://dx.doi.org/10.1109%2FTITB.2002.802378) | 2002 | 7.5721 | **2003** | 2008 | ▂▂▂▂▂▂▂▂▂▂▂▂▂▃▃▃▃▃▃▂▂▂▂▂▂▂▂ |
| REZNEK M, 2002, ACAD EMERG MED, V9, P78 | 2002 | 5.1912 | **2003** | 2007 | ▂▂▂▂▂▂▂▂▂▂▂▂▂▃▃▃▃▃▂▂▂▂▂▂▂▂▂ |
| SCHUBERT T, 2001, PRESENCE-TELEOP VIRT, V10, P266, [DOI](http://dx.doi.org/10.1162%2F105474601300343603) | 2001 | 8.9427 | **2003** | 2009 | ▂▂▂▂▂▂▂▂▂▂▂▂▂▃▃▃▃▃▃▃▂▂▂▂▂▂▂ |
| JORDAN JA, 2001, SURG ENDOSC-ULTRAS, V15, P1080, [DOI](http://dx.doi.org/10.1007%2FS004640000374) | 2001 | 16.2277 | **2003** | 2009 | ▂▂▂▂▂▂▂▂▂▂▂▂▂▃▃▃▃▃▃▃▂▂▂▂▂▂▂ |
| NEWMAN MG, 1997, J CONSULT CLIN PSYCH, V65, P178, [DOI](http://dx.doi.org/10.1037%2F0022-006X.65.1.178) | 1997 | 4.3532 | **2003** | 2005 | ▂▂▂▂▂▂▂▂▂▂▂▂▂▃▃▃▂▂▂▂▂▂▂▂▂▂▂ |
| ROTHBAUM BO, 2001, J CLIN PSYCHIAT, V62, P617 | 2001 | 19.8658 | **2003** | 2009 | ▂▂▂▂▂▂▂▂▂▂▂▂▂▃▃▃▃▃▃▃▂▂▂▂▂▂▂ |
| BURGAR CG, 2000, J REHABIL RES DEV, V37, P663 | 2000 | 9.4648 | **2003** | 2008 | ▂▂▂▂▂▂▂▂▂▂▂▂▂▃▃▃▃▃▃▂▂▂▂▂▂▂▂ |
| GARCIA-PALACIOS A, 2002, BEHAV RES THER, V40, P983, [DOI](http://dx.doi.org/10.1016%2FS0005-7967%2801%2900068-7) | 2002 | 12.6406 | **2003** | 2010 | ▂▂▂▂▂▂▂▂▂▂▂▂▂▃▃▃▃▃▃▃▃▂▂▂▂▂▂ |
| TORKINGTON J, 2001, SURG ENDOSC, V15, P1076, [DOI](http://dx.doi.org/10.1007%2FS004640000233) | 2001 | 24.4184 | **2003** | 2008 | ▂▂▂▂▂▂▂▂▂▂▂▂▂▃▃▃▃▃▃▂▂▂▂▂▂▂▂ |
| DATTA V, 2002, AM J SURG, V184, P70, [DOI](http://dx.doi.org/10.1016%2FS0002-9610%2802%2900891-7) | 2002 | 8.4514 | **2003** | 2010 | ▂▂▂▂▂▂▂▂▂▂▂▂▂▃▃▃▃▃▃▃▃▂▂▂▂▂▂ |
| DAWSON SL, 2000, CATHETER CARDIO INTE, V51, P522 | 2000 | 7.9803 | **2003** | 2008 | ▂▂▂▂▂▂▂▂▂▂▂▂▂▃▃▃▃▃▃▂▂▂▂▂▂▂▂ |
| HOFFMAN HG, 2001, CLIN J PAIN, V17, P229, [DOI](http://dx.doi.org/10.1097%2F00002508-200109000-00007) | 2001 | 16.531 | **2003** | 2009 | ▂▂▂▂▂▂▂▂▂▂▂▂▂▃▃▃▃▃▃▃▂▂▂▂▂▂▂ |
| HYLTANDER A, 2002, SURG ENDOSC, V16, P1324, [DOI](http://dx.doi.org/10.1007%2FS00464-001-9184-5) | 2002 | 25.0114 | **2004** | 2009 | ▂▂▂▂▂▂▂▂▂▂▂▂▂▂▃▃▃▃▃▃▂▂▂▂▂▂▂ |
| HAMILTON EC, 2002, SURG ENDOSC, V16, P406, [DOI](http://dx.doi.org/10.1007%2FS00464-001-8149-Z) | 2002 | 20.3507 | **2004** | 2010 | ▂▂▂▂▂▂▂▂▂▂▂▂▂▂▃▃▃▃▃▃▃▂▂▂▂▂▂ |
| REINKENSMEYER DJ, 2002, IEEE T NEUR SYS REH, V10, P102, [DOI](http://dx.doi.org/10.1109%2FTNSRE.2002.1031978) | 2002 | 8.7544 | **2004** | 2010 | ▂▂▂▂▂▂▂▂▂▂▂▂▂▂▃▃▃▃▃▃▃▂▂▂▂▂▂ |
| AHLBERG G, 2002, SURG ENDOSC, V16, P126, [DOI](http://dx.doi.org/10.1007%2FS00464-001-9025-6) | 2002 | 17.6919 | **2004** | 2008 | ▂▂▂▂▂▂▂▂▂▂▂▂▂▂▃▃▃▃▃▂▂▂▂▂▂▂▂ |
| TARR MJ, 2002, NAT NEUROSCI, V5, P1089, [DOI](http://dx.doi.org/10.1038%2FNN948) | 2002 | 9.8149 | **2004** | 2010 | ▂▂▂▂▂▂▂▂▂▂▂▂▂▂▃▃▃▃▃▃▃▂▂▂▂▂▂ |
| KOTHARI SN, 2002, J LAPAROENDOSC ADV A, V12, P167, [DOI](http://dx.doi.org/10.1089%2F10926420260188056) | 2002 | 12.5006 | **2004** | 2007 | ▂▂▂▂▂▂▂▂▂▂▂▂▂▂▃▃▃▃▂▂▂▂▂▂▂▂▂ |
| BURDEA G C, 2003, VIRTUAL REALITY TECH, V, P | 2003 | 31.3048 | **2004** | 2010 | ▂▂▂▂▂▂▂▂▂▂▂▂▂▂▃▃▃▃▃▃▃▂▂▂▂▂▂ |
| GRANTCHAROV TP, 2003, AM J SURG, V185, P146, [DOI](http://dx.doi.org/10.1016%2FS0002-9610%2802%2901213-8) | 2003 | 23.6715 | **2004** | 2010 | ▂▂▂▂▂▂▂▂▂▂▂▂▂▂▃▃▃▃▃▃▃▂▂▂▂▂▂ |
| GALLAGHER AG, 2002, SURG ENDOSC, V16, P1746, [DOI](http://dx.doi.org/10.1007%2FS00464-001-8215-6) | 2002 | 24.857 | **2004** | 2009 | ▂▂▂▂▂▂▂▂▂▂▂▂▂▂▃▃▃▃▃▃▂▂▂▂▂▂▂ |
| GOR M, 2003, BJOG-INT J OBSTET GY, V110, P181, [DOI](http://dx.doi.org/10.1016%2FS1470-0328%2802%2902516-8) | 2003 | 6.7916 | **2004** | 2007 | ▂▂▂▂▂▂▂▂▂▂▂▂▂▂▃▃▃▃▂▂▂▂▂▂▂▂▂ |
| SCHIJVEN M, 2003, SURG ENDOSC, V17, P803, [DOI](http://dx.doi.org/10.1007%2FS00464-002-9151-9) | 2003 | 9.7267 | **2004** | 2011 | ▂▂▂▂▂▂▂▂▂▂▂▂▂▂▃▃▃▃▃▃▃▃▂▂▂▂▂ |
| AMERICAN PSYCHIATRIC ASSOCIATION, 2000, DIAGN STAT MAN MENT, V, P | 2000 | 8.7667 | **2004** | 2008 | ▂▂▂▂▂▂▂▂▂▂▂▂▂▂▃▃▃▃▃▂▂▂▂▂▂▂▂ |
| SEYMOUR NE, 2002, ANN SURG, V236, P458, [DOI](http://dx.doi.org/10.1097%2F01.SLA.0000028969.51489.B4) | 2002 | 88.9251 | **2004** | 2010 | ▂▂▂▂▂▂▂▂▂▂▂▂▂▂▃▃▃▃▃▃▃▂▂▂▂▂▂ |
| MERIANS AS, 2002, PHYS THER, V82, P898 | 2002 | 19.7935 | **2004** | 2010 | ▂▂▂▂▂▂▂▂▂▂▂▂▂▂▃▃▃▃▃▃▃▂▂▂▂▂▂ |
| KREBS H I, 1998, IEEE TRANS REHABIL ENG, V6, P75, [DOI](http://dx.doi.org/10.1109%2F86.662623) | 1998 | 7.9715 | **2004** | 2006 | ▂▂▂▂▂▂▂▂▂▂▂▂▂▂▃▃▃▂▂▂▂▂▂▂▂▂▂ |
| STROM P, 2003, SURG ENDOSC, V17, P227, [DOI](http://dx.doi.org/10.1007%2FS00464-002-9078-1) | 2003 | 10.9757 | **2004** | 2006 | ▂▂▂▂▂▂▂▂▂▂▂▂▂▂▃▃▃▂▂▂▂▂▂▂▂▂▂ |
| WATTERSON JD, 2002, J UROLOGY, V168, P1928, [DOI](http://dx.doi.org/10.1097%2F01.JU.0000034357.84449.56) | 2002 | 11.0958 | **2004** | 2010 | ▂▂▂▂▂▂▂▂▂▂▂▂▂▂▃▃▃▃▃▃▃▂▂▂▂▂▂ |
| FERLITSCH A, 2002, ENDOSCOPY, V34, P698, [DOI](http://dx.doi.org/10.1055%2FS-2002-33456) | 2002 | 8.937 | **2004** | 2010 | ▂▂▂▂▂▂▂▂▂▂▂▂▂▂▃▃▃▃▃▃▃▂▂▂▂▂▂ |
| DIFEDE J, 2002, CYBERPSYCHOL BEHAV, V5, P529, [DOI](http://dx.doi.org/10.1089%2F109493102321018169) | 2002 | 11.7524 | **2005** | 2010 | ▂▂▂▂▂▂▂▂▂▂▂▂▂▂▂▃▃▃▃▃▃▂▂▂▂▂▂ |
| SCHIJVEN M, 2003, SURG ENDOSC, V17, P1943, [DOI](http://dx.doi.org/10.1007%2FS00464-003-9052-6) | 2003 | 10.4096 | **2005** | 2008 | ▂▂▂▂▂▂▂▂▂▂▂▂▂▂▂▃▃▃▃▂▂▂▂▂▂▂▂ |
| PEARSON AM, 2002, SURG ENDOSC, V16, P130, [DOI](http://dx.doi.org/10.1007%2FS00464-001-8113-Y) | 2002 | 10.3914 | **2005** | 2008 | ▂▂▂▂▂▂▂▂▂▂▂▂▂▂▂▃▃▃▃▂▂▂▂▂▂▂▂ |
| MOORTHY K, 2003, BRIT MED J, V327, P1032, [DOI](http://dx.doi.org/10.1136%2FBMJ.327.7422.1032) | 2003 | 17.01 | **2005** | 2011 | ▂▂▂▂▂▂▂▂▂▂▂▂▂▂▂▃▃▃▃▃▃▃▂▂▂▂▂ |
| ERNST MO, 2002, NATURE, V415, P429, [DOI](http://dx.doi.org/10.1038%2F415429A) | 2002 | 9.7992 | **2005** | 2010 | ▂▂▂▂▂▂▂▂▂▂▂▂▂▂▂▃▃▃▃▃▃▂▂▂▂▂▂ |
| GALLAGHER AG, 2004, SURG ENDOSC, V18, P660, [DOI](http://dx.doi.org/10.1007%2FS00464-003-8176-Z) | 2004 | 12.4036 | **2005** | 2007 | ▂▂▂▂▂▂▂▂▂▂▂▂▂▂▂▃▃▃▂▂▂▂▂▂▂▂▂ |
| CRAIG A, 2003, UNDERSTANDING VIRTUA, V, P | 2003 | 8.8403 | **2005** | 2010 | ▂▂▂▂▂▂▂▂▂▂▂▂▂▂▂▃▃▃▃▃▃▂▂▂▂▂▂ |
| GALLAGHER AG, 2003, J AM COLL SURGEONS, V197, P479, [DOI](http://dx.doi.org/10.1016%2FS1072-7515%2803%2900535-0) | 2003 | 11.3307 | **2005** | 2008 | ▂▂▂▂▂▂▂▂▂▂▂▂▂▂▂▃▃▃▃▂▂▂▂▂▂▂▂ |
| GRANTCHAROV TP, 2004, BRIT J SURG, V91, P146, [DOI](http://dx.doi.org/10.1002%2FBJS.4407) | 2004 | 51.4032 | **2005** | 2012 | ▂▂▂▂▂▂▂▂▂▂▂▂▂▂▂▃▃▃▃▃▃▃▃▂▂▂▂ |
| GERSHON J, 2004, J AM ACAD CHILD PSY, V43, P1243, [DOI](http://dx.doi.org/10.1097%2F01.CHI.0000135621.23145.05) | 2004 | 6.6595 | **2005** | 2011 | ▂▂▂▂▂▂▂▂▂▂▂▂▂▂▂▃▃▃▃▃▃▃▂▂▂▂▂ |
| KRIJN M, 2004, BEHAV RES THER, V42, P229, [DOI](http://dx.doi.org/10.1016%2FS0005-7967%2803%2900139-6) | 2004 | 8.8656 | **2005** | 2012 | ▂▂▂▂▂▂▂▂▂▂▂▂▂▂▂▃▃▃▃▃▃▃▃▂▂▂▂ |
| MUNZ Y, 2004, SURG ENDOSC, V18, P485, [DOI](http://dx.doi.org/10.1007%2FS00464-003-9043-7) | 2004 | 22.4854 | **2005** | 2012 | ▂▂▂▂▂▂▂▂▂▂▂▂▂▂▂▃▃▃▃▃▃▃▃▂▂▂▂ |
| GALLAGHER AG, 2003, SURG ENDOSC, V17, P1525, [DOI](http://dx.doi.org/10.1007%2FS00464-003-0035-4) | 2003 | 9.9586 | **2005** | 2011 | ▂▂▂▂▂▂▂▂▂▂▂▂▂▂▂▃▃▃▃▃▃▃▂▂▂▂▂ |
| GLANTZ K, 2003, PSYCHOTHERAPY, V40, P55, [DOI](http://dx.doi.org/10.1037%2F0033-3204.40.1%2F2.55) | 2003 | 8.0494 | **2005** | 2011 | ▂▂▂▂▂▂▂▂▂▂▂▂▂▂▂▃▃▃▃▃▃▃▂▂▂▂▂ |
| AGGARWAL R, 2004, BRIT J SURG, V91, P1549, [DOI](http://dx.doi.org/10.1002%2FBJS.4816) | 2004 | 17.8659 | **2006** | 2012 | ▂▂▂▂▂▂▂▂▂▂▂▂▂▂▂▂▃▃▃▃▃▃▃▂▂▂▂ |
| DUFFY AJ, 2005, SURG ENDOSC, V19, P401, [DOI](http://dx.doi.org/10.1007%2FS00464-004-8202-9) | 2005 | 9.8764 | **2006** | 2013 | ▂▂▂▂▂▂▂▂▂▂▂▂▂▂▂▂▃▃▃▃▃▃▃▃▂▂▂ |
| LEHMANN KS, 2005, ANN SURG, V241, P442, [DOI](http://dx.doi.org/10.1097%2F01.SLA.0000154552.89886.91) | 2005 | 8.6514 | **2006** | 2013 | ▂▂▂▂▂▂▂▂▂▂▂▂▂▂▂▂▃▃▃▃▃▃▃▃▂▂▂ |
| HOFFMAN HG, 2004, J CLIN PSYCHOL, V60, P189, [DOI](http://dx.doi.org/10.1002%2FJCLP.10244) | 2004 | 7.4467 | **2006** | 2011 | ▂▂▂▂▂▂▂▂▂▂▂▂▂▂▂▂▃▃▃▃▃▃▂▂▂▂▂ |
| HOFFMAN HG, 2004, NEUROREPORT, V15, P1245, [DOI](http://dx.doi.org/10.1097%2F01.WNR.0000127829.73576.91) | 2004 | 10.1231 | **2006** | 2011 | ▂▂▂▂▂▂▂▂▂▂▂▂▂▂▂▂▃▃▃▃▃▃▂▂▂▂▂ |
| GROBER ED, 2004, ANN SURG, V240, P374, [DOI](http://dx.doi.org/10.1097%2F01.SLA.0000133346.07434.30) | 2004 | 11.6236 | **2006** | 2012 | ▂▂▂▂▂▂▂▂▂▂▂▂▂▂▂▂▃▃▃▃▃▃▃▂▂▂▂ |
| WEISS PL, 2004, J NEUROENG REHABIL, V1, P12 | 2004 | 10.4517 | **2006** | 2012 | ▂▂▂▂▂▂▂▂▂▂▂▂▂▂▂▂▃▃▃▃▃▃▃▂▂▂▂ |
| GRANTCHAROV TP, 2003, SURG ENDOSC, V17, P1082, [DOI](http://dx.doi.org/10.1007%2FS00464-002-9176-0) | 2003 | 7.5535 | **2006** | 2011 | ▂▂▂▂▂▂▂▂▂▂▂▂▂▂▂▂▃▃▃▃▃▃▂▂▂▂▂ |
| SVEISTRUP H, 2004, J NEUROENG REHABIL, V1, P10 | 2004 | 12.1552 | **2006** | 2012 | ▂▂▂▂▂▂▂▂▂▂▂▂▂▂▂▂▃▃▃▃▃▃▃▂▂▂▂ |
| MATSUMOTO ED, 2002, J UROLOGY, V167, P1243, [DOI](http://dx.doi.org/10.1016%2FS0022-5347%2805%2965274-3) | 2002 | 10.9928 | **2006** | 2010 | ▂▂▂▂▂▂▂▂▂▂▂▂▂▂▂▂▃▃▃▃▃▂▂▂▂▂▂ |
| BRUNNER WC, 2004, J SURG RES, V122, P150, [DOI](http://dx.doi.org/10.1016%2FJ.JSS.2004.08.006) | 2004 | 8.4123 | **2006** | 2010 | ▂▂▂▂▂▂▂▂▂▂▂▂▂▂▂▂▃▃▃▃▃▂▂▂▂▂▂ |
| KNEEBONE R, 2003, MED EDUC, V37, P267, [DOI](http://dx.doi.org/10.1046%2FJ.1365-2923.2003.01440.X) | 2003 | 12.0681 | **2006** | 2011 | ▂▂▂▂▂▂▂▂▂▂▂▂▂▂▂▂▃▃▃▃▃▃▂▂▂▂▂ |
| FRIED GM, 2004, ANN SURG, V240, P518, [DOI](http://dx.doi.org/10.1097%2F01.SLA.0000136941.46529.56) | 2004 | 23.3969 | **2006** | 2012 | ▂▂▂▂▂▂▂▂▂▂▂▂▂▂▂▂▃▃▃▃▃▃▃▂▂▂▂ |
| GALLAGHER AG, 2004, JAMA-J AM MED ASSOC, V292, P3024, [DOI](http://dx.doi.org/10.1001%2FJAMA.292.24.3024) | 2004 | 9.0423 | **2006** | 2012 | ▂▂▂▂▂▂▂▂▂▂▂▂▂▂▂▂▃▃▃▃▃▃▃▂▂▂▂ |
| JACOMIDES L, 2004, J UROLOGY, V171, P320, [DOI](http://dx.doi.org/10.1097%2F01.JU.0000101515.70623.4A) | 2004 | 9.543 | **2006** | 2010 | ▂▂▂▂▂▂▂▂▂▂▂▂▂▂▂▂▃▃▃▃▃▂▂▂▂▂▂ |
| HENN JS, 2002, J NEUROSURG, V96, P144, [DOI](http://dx.doi.org/10.3171%2FJNS.2002.96.1.0144) | 2002 | 8.7188 | **2007** | 2010 | ▂▂▂▂▂▂▂▂▂▂▂▂▂▂▂▂▂▃▃▃▃▂▂▂▂▂▂ |
| YOUNGBLOOD PL, 2005, J AM COLL SURGEONS, V200, P546, [DOI](http://dx.doi.org/10.1016%2FJ.JAMCOLLSURG.2004.11.011) | 2005 | 9.8941 | **2007** | 2013 | ▂▂▂▂▂▂▂▂▂▂▂▂▂▂▂▂▂▃▃▃▃▃▃▃▂▂▂ |
| KORNDORFFER JR, 2005, J AM COLL SURGEONS, V201, P23, [DOI](http://dx.doi.org/10.1016%2FJ.JAMCOLLSURG.2005.02.021) | 2005 | 14.3445 | **2007** | 2012 | ▂▂▂▂▂▂▂▂▂▂▂▂▂▂▂▂▂▃▃▃▃▃▃▂▂▂▂ |
| WOODRUM DT, 2006, AM J SURG, V191, P28, [DOI](http://dx.doi.org/10.1016%2FJ.AMJSURG.2005.10.018) | 2006 | 9.2233 | **2007** | 2013 | ▂▂▂▂▂▂▂▂▂▂▂▂▂▂▂▂▂▃▃▃▃▃▃▃▂▂▂ |
| FUNG J, 2006, CYBERPSYCHOL BEHAV, V9, P157, [DOI](http://dx.doi.org/10.1089%2FCPB.2006.9.157) | 2006 | 7.5543 | **2007** | 2011 | ▂▂▂▂▂▂▂▂▂▂▂▂▂▂▂▂▂▃▃▃▃▃▂▂▂▂▂ |
| RIVA G, 2005, CYBERPSYCHOL BEHAV, V8, P220, [DOI](http://dx.doi.org/10.1089%2FCPB.2005.8.220) | 2005 | 14.0634 | **2007** | 2013 | ▂▂▂▂▂▂▂▂▂▂▂▂▂▂▂▂▂▃▃▃▃▃▃▃▂▂▂ |
| CARTER FJ, 2005, SURG ENDOSC, V19, P1523, [DOI](http://dx.doi.org/10.1007%2FS00464-005-0384-2) | 2005 | 15.7404 | **2007** | 2012 | ▂▂▂▂▂▂▂▂▂▂▂▂▂▂▂▂▂▃▃▃▃▃▃▂▂▂▂ |
| KORNDORFFER JR, 2006, AM J SURG, V191, P17, [DOI](http://dx.doi.org/10.1016%2FJ.AMJSURG.2005.05.048) | 2006 | 9.284 | **2007** | 2012 | ▂▂▂▂▂▂▂▂▂▂▂▂▂▂▂▂▂▃▃▃▃▃▃▂▂▂▂ |
| BOWMAN DA, 2004, 3D USER INTERFACES T, V, P | 2004 | 9.7877 | **2007** | 2011 | ▂▂▂▂▂▂▂▂▂▂▂▂▂▂▂▂▂▃▃▃▃▃▂▂▂▂▂ |
| SEYMOUR NE, 2002, ANN SURG, V236, P463 | 2002 | 9.9305 | **2007** | 2010 | ▂▂▂▂▂▂▂▂▂▂▂▂▂▂▂▂▂▃▃▃▃▂▂▂▂▂▂ |
| DAS DEBASHISH A, 2005, BMC PEDIATR, V5, P1, [DOI](http://dx.doi.org/10.1186%2F1471-2431-5-1) | 2005 | 5.9533 | **2007** | 2010 | ▂▂▂▂▂▂▂▂▂▂▂▂▂▂▂▂▂▃▃▃▃▂▂▂▂▂▂ |
| HOLDEN MK, 2005, CYBERPSYCHOL BEHAV, V8, P187, [DOI](http://dx.doi.org/10.1089%2FCPB.2005.8.187) | 2005 | 37.5173 | **2007** | 2013 | ▂▂▂▂▂▂▂▂▂▂▂▂▂▂▂▂▂▃▃▃▃▃▃▃▂▂▂ |
| PETERS JH, 2004, SURGERY, V135, P21, [DOI](http://dx.doi.org/10.1016%2FS0039-6060%2803%2900156-9) | 2004 | 9.7785 | **2007** | 2012 | ▂▂▂▂▂▂▂▂▂▂▂▂▂▂▂▂▂▃▃▃▃▃▃▂▂▂▂ |
| MERIANS AS, 2006, NEUROREHAB NEURAL RE, V20, P252, [DOI](http://dx.doi.org/10.1177%2F1545968306286914) | 2006 | 11.4769 | **2007** | 2011 | ▂▂▂▂▂▂▂▂▂▂▂▂▂▂▂▂▂▃▃▃▃▃▂▂▂▂▂ |
| ERICSSON KA, 2004, ACAD MED, V79, , [DOI](http://dx.doi.org/10.1097%2F00001888-200410001-00022) | 2004 | 9.9796 | **2007** | 2012 | ▂▂▂▂▂▂▂▂▂▂▂▂▂▂▂▂▂▃▃▃▃▃▃▂▂▂▂ |
| THOMPSON WB, 2004, PRESENCE-TELEOP VIRT, V13, P560, [DOI](http://dx.doi.org/10.1162%2F1054746042545292) | 2004 | 6.7861 | **2007** | 2011 | ▂▂▂▂▂▂▂▂▂▂▂▂▂▂▂▂▂▃▃▃▃▃▂▂▂▂▂ |
| AGGARWAL R, 2006, EUR J VASC ENDOVASC, V31, P588, [DOI](http://dx.doi.org/10.1016%2FJ.EJVS.2005.11.009) | 2006 | 7.6964 | **2007** | 2014 | ▂▂▂▂▂▂▂▂▂▂▂▂▂▂▂▂▂▃▃▃▃▃▃▃▃▂▂ |
| GALLAGHER AG, 2005, ANN SURG, V241, P364, [DOI](http://dx.doi.org/10.1097%2F01.SLA.0000151982.85062.80) | 2005 | 27.9285 | **2007** | 2013 | ▂▂▂▂▂▂▂▂▂▂▂▂▂▂▂▂▂▃▃▃▃▃▃▃▂▂▂ |
| CHAER RA, 2006, ANN SURG, V244, P343, [DOI](http://dx.doi.org/10.1097%2F01.SLA.0000234932.88487.75) | 2006 | 9.1252 | **2008** | 2014 | ▂▂▂▂▂▂▂▂▂▂▂▂▂▂▂▂▂▂▃▃▃▃▃▃▃▂▂ |
| ANDREATTA PB, 2006, ANN SURG, V243, P854, [DOI](http://dx.doi.org/10.1097%2F01.SLA.0000219641.79092.E5) | 2006 | 15.5861 | **2008** | 2014 | ▂▂▂▂▂▂▂▂▂▂▂▂▂▂▂▂▂▂▃▃▃▃▃▃▃▂▂ |
| ANDERSON PL, 2005, DEPRESS ANXIETY, V22, P156, [DOI](http://dx.doi.org/10.1002%2FDA.20090) | 2005 | 9.5295 | **2008** | 2013 | ▂▂▂▂▂▂▂▂▂▂▂▂▂▂▂▂▂▂▃▃▃▃▃▃▂▂▂ |
| SANCHEZ-VIVES MV, 2005, NAT REV NEUROSCI, V6, P332, [DOI](http://dx.doi.org/10.1038%2FNRN1651) | 2005 | 27.7624 | **2008** | 2013 | ▂▂▂▂▂▂▂▂▂▂▂▂▂▂▂▂▂▂▃▃▃▃▃▃▂▂▂ |
| MADAN AK, 2005, AM J SURG, V189, P758, [DOI](http://dx.doi.org/10.1016%2FJ.AMJSURG.2005.03.022) | 2005 | 6.5774 | **2008** | 2013 | ▂▂▂▂▂▂▂▂▂▂▂▂▂▂▂▂▂▂▃▃▃▃▃▃▂▂▂ |
| SEDLACK RE, 2004, CLIN GASTROENTEROL H, V2, P348, [DOI](http://dx.doi.org/10.1053%2FS1542-3565%2804%2900067-9) | 2004 | 8.3601 | **2008** | 2012 | ▂▂▂▂▂▂▂▂▂▂▂▂▂▂▂▂▂▂▃▃▃▃▃▂▂▂▂ |
| AGGARWAL R, 2006, AM J SURG, V191, P128, [DOI](http://dx.doi.org/10.1016%2FJ.AMJSURG.2005.10.014) | 2006 | 12.431 | **2008** | 2013 | ▂▂▂▂▂▂▂▂▂▂▂▂▂▂▂▂▂▂▃▃▃▃▃▃▂▂▂ |
| SUTHERLAND LM, 2006, ANN SURG, V243, P291, [DOI](http://dx.doi.org/10.1097%2F01.SLA.0000200839.93965.26) | 2006 | 17.3025 | **2008** | 2014 | ▂▂▂▂▂▂▂▂▂▂▂▂▂▂▂▂▂▂▃▃▃▃▃▃▃▂▂ |
| ROTHBAUM BO, 2006, BEHAV THER, V37, P80, [DOI](http://dx.doi.org/10.1016%2FJ.BETH.2005.04.004) | 2006 | 9.1252 | **2008** | 2014 | ▂▂▂▂▂▂▂▂▂▂▂▂▂▂▂▂▂▂▃▃▃▃▃▃▃▂▂ |
| STEFANIDIS D, 2006, J AM COLL SURGEONS, V202, P599, [DOI](http://dx.doi.org/10.1016%2FJ.JAMCOLLSURG.2005.12.018) | 2006 | 6.9232 | **2008** | 2013 | ▂▂▂▂▂▂▂▂▂▂▂▂▂▂▂▂▂▂▃▃▃▃▃▃▂▂▂ |
| WALSHE DG, 2003, CYBERPSYCHOL BEHAV, V6, P329, [DOI](http://dx.doi.org/10.1089%2F109493103322011641) | 2003 | 9.9323 | **2008** | 2011 | ▂▂▂▂▂▂▂▂▂▂▂▂▂▂▂▂▂▂▃▃▃▃▂▂▂▂▂ |
| CHOU DS, 2006, J ENDOUROL, V20, P266, [DOI](http://dx.doi.org/10.1089%2FEND.2006.20.266) | 2006 | 6.7764 | **2008** | 2011 | ▂▂▂▂▂▂▂▂▂▂▂▂▂▂▂▂▂▂▃▃▃▃▂▂▂▂▂ |
| BLASCOVICH J, 2002, PSYCHOL INQ, V13, P103, [DOI](http://dx.doi.org/10.1207%2FS15327965PLI1302_01) | 2002 | 10.8641 | **2008** | 2010 | ▂▂▂▂▂▂▂▂▂▂▂▂▂▂▂▂▂▂▃▃▃▂▂▂▂▂▂ |
| RIZZO A, 2005, PRESENCE-TELEOP VIRT, V14, P119, [DOI](http://dx.doi.org/10.1162%2F1054746053967094) | 2005 | 17.9039 | **2008** | 2013 | ▂▂▂▂▂▂▂▂▂▂▂▂▂▂▂▂▂▂▃▃▃▃▃▃▂▂▂ |
| MCDOUGALL EM, 2006, J AM COLL SURGEONS, V202, P779, [DOI](http://dx.doi.org/10.1016%2FJ.JAMCOLLSURG.2006.01-004) | 2006 | 12.7787 | **2008** | 2013 | ▂▂▂▂▂▂▂▂▂▂▂▂▂▂▂▂▂▂▃▃▃▃▃▃▂▂▂ |
| MOULTON CAE, 2006, ANN SURG, V244, P400, [DOI](http://dx.doi.org/10.1097%2F01.SLA.0000234808.85789.6A) | 2006 | 11.7334 | **2008** | 2013 | ▂▂▂▂▂▂▂▂▂▂▂▂▂▂▂▂▂▂▃▃▃▃▃▃▂▂▂ |
| REZNICK RK, 2006, NEW ENGL J MED, V355, P2664 | 2006 | 24.6838 | **2008** | 2013 | ▂▂▂▂▂▂▂▂▂▂▂▂▂▂▂▂▂▂▃▃▃▃▃▃▂▂▂ |
| YOU SH, 2005, STROKE, V36, P1166, [DOI](http://dx.doi.org/10.1161%2F01.STR.0000162715.43417.91) | 2005 | 12.0245 | **2008** | 2013 | ▂▂▂▂▂▂▂▂▂▂▂▂▂▂▂▂▂▂▃▃▃▃▃▃▂▂▂ |
| MADAN AK, 2007, SURG ENDOSC, V21, P209, [DOI](http://dx.doi.org/10.1007%2FS00464-006-0149-6) | 2007 | 7.4453 | **2008** | 2013 | ▂▂▂▂▂▂▂▂▂▂▂▂▂▂▂▂▂▂▃▃▃▃▃▃▂▂▂ |
| AGGARWAL R, 2006, ANN SURG, V244, P310, [DOI](http://dx.doi.org/10.1097%2F01.SLA.0000218094.92650.44) | 2006 | 16.2754 | **2008** | 2013 | ▂▂▂▂▂▂▂▂▂▂▂▂▂▂▂▂▂▂▃▃▃▃▃▃▂▂▂ |
| LEE KM, 2004, COMMUN THEOR, V14, P27, [DOI](http://dx.doi.org/10.1093%2FCT%2F14.1.27) | 2004 | 8.6273 | **2009** | 2012 | ▂▂▂▂▂▂▂▂▂▂▂▂▂▂▂▂▂▂▂▃▃▃▃▂▂▂▂ |
| HOFFMAN HG, 2008, CLIN J PAIN, V24, P299, [DOI](http://dx.doi.org/10.1097%2FAJP.0B013E318164D2CC) | 2008 | 8.8419 | **2009** | 2016 | ▂▂▂▂▂▂▂▂▂▂▂▂▂▂▂▂▂▂▂▃▃▃▃▃▃▃▃ |
| BRYANTON C, 2006, CYBERPSYCHOL BEHAV, V9, P123, [DOI](http://dx.doi.org/10.1089%2FCPB.2006.9.123) | 2006 | 12.1692 | **2009** | 2014 | ▂▂▂▂▂▂▂▂▂▂▂▂▂▂▂▂▂▂▂▃▃▃▃▃▃▂▂ |
| RIVA G, 2007, CYBERPSYCHOL BEHAV, V10, P45, [DOI](http://dx.doi.org/10.1089%2FCPB.2006.9993) | 2007 | 8.7854 | **2009** | 2016 | ▂▂▂▂▂▂▂▂▂▂▂▂▂▂▂▂▂▂▂▃▃▃▃▃▃▃▃ |
| ISSENBERG SB, 2005, MED TEACH, V27, P10, [DOI](http://dx.doi.org/10.1080%2F01421590500046924) | 2005 | 18.8332 | **2009** | 2013 | ▂▂▂▂▂▂▂▂▂▂▂▂▂▂▂▂▂▂▂▃▃▃▃▃▂▂▂ |
| YOU SH, 2005, DEV MED CHILD NEUROL, V47, P628, [DOI](http://dx.doi.org/10.1017%2FS0012162205001234) | 2005 | 9.388 | **2009** | 2013 | ▂▂▂▂▂▂▂▂▂▂▂▂▂▂▂▂▂▂▂▃▃▃▃▃▂▂▂ |
| ROSE FD, 2005, CYBERPSYCHOL BEHAV, V8, P241, [DOI](http://dx.doi.org/10.1089%2FCPB.2005.8.241) | 2005 | 13.9486 | **2009** | 2013 | ▂▂▂▂▂▂▂▂▂▂▂▂▂▂▂▂▂▂▂▃▃▃▃▃▂▂▂ |
| BOTDEN SMBI, 2007, WORLD J SURG, V31, P764, [DOI](http://dx.doi.org/10.1007%2FS00268-006-0724-Y) | 2007 | 8.2478 | **2009** | 2014 | ▂▂▂▂▂▂▂▂▂▂▂▂▂▂▂▂▂▂▂▃▃▃▃▃▃▂▂ |
| AGGARWAL R, 2007, ANN SURG, V246, P771, [DOI](http://dx.doi.org/10.1097%2FSLA.0B013E3180F61B09) | 2007 | 22.7963 | **2009** | 2016 | ▂▂▂▂▂▂▂▂▂▂▂▂▂▂▂▂▂▂▂▃▃▃▃▃▃▃▃ |
| VAN DONGEN KW, 2007, SURG ENDOSC, V21, P1413, [DOI](http://dx.doi.org/10.1007%2FS00464-006-9188-2) | 2007 | 11.6176 | **2009** | 2014 | ▂▂▂▂▂▂▂▂▂▂▂▂▂▂▂▂▂▂▂▃▃▃▃▃▃▂▂ |
| GERARDI M, 2008, J TRAUMA STRESS, V21, P209, [DOI](http://dx.doi.org/10.1002%2FJTS.20331) | 2008 | 9.0644 | **2009** | 2016 | ▂▂▂▂▂▂▂▂▂▂▂▂▂▂▂▂▂▂▂▃▃▃▃▃▃▃▃ |
| VASSILIOU MC, 2005, AM J SURG, V190, P107, [DOI](http://dx.doi.org/10.1016%2FJ.AMJSURG.2005.04.004) | 2005 | 13.5773 | **2009** | 2013 | ▂▂▂▂▂▂▂▂▂▂▂▂▂▂▂▂▂▂▂▃▃▃▃▃▂▂▂ |
| DIFEDE J, 2007, J CLIN PSYCHIAT, V68, P1639 | 2007 | 12.5851 | **2009** | 2014 | ▂▂▂▂▂▂▂▂▂▂▂▂▂▂▂▂▂▂▂▃▃▃▃▃▃▂▂ |
| GREGG L, 2007, SOC PSYCH PSYCH EPID, V42, P343, [DOI](http://dx.doi.org/10.1007%2FS00127-007-0173-4) | 2007 | 9.8986 | **2009** | 2016 | ▂▂▂▂▂▂▂▂▂▂▂▂▂▂▂▂▂▂▂▃▃▃▃▃▃▃▃ |
| HENDERSON A, 2007, TOP STROKE REHABIL, V14, P52, [DOI](http://dx.doi.org/10.1310%2FTSR1402-52) | 2007 | 17.6612 | **2009** | 2016 | ▂▂▂▂▂▂▂▂▂▂▂▂▂▂▂▂▂▂▂▃▃▃▃▃▃▃▃ |
| EHRSSON HH, 2007, SCIENCE, V317, P1048, [DOI](http://dx.doi.org/10.1126%2FSCIENCE.1142175) | 2007 | 8.1463 | **2009** | 2016 | ▂▂▂▂▂▂▂▂▂▂▂▂▂▂▂▂▂▂▂▃▃▃▃▃▃▃▃ |
| WOOD DP, 2007, CYBERPSYCHOL BEHAV, V10, P309, [DOI](http://dx.doi.org/10.1089%2FCPB.2006.9951) | 2007 | 6.5385 | **2009** | 2010 | ▂▂▂▂▂▂▂▂▂▂▂▂▂▂▂▂▂▂▂▃▃▂▂▂▂▂▂ |
| JANG SUNG HO, 2005, ARCH PHYS MED REHABIL, V86, P2218, [DOI](http://dx.doi.org/10.1016%2FJ.APMR.2005.04.015) | 2005 | 10.7803 | **2009** | 2013 | ▂▂▂▂▂▂▂▂▂▂▂▂▂▂▂▂▂▂▂▃▃▃▃▃▂▂▂ |
| PARSONS TD, 2008, CYBERPSYCHOL BEHAV, V11, P17, [DOI](http://dx.doi.org/10.1089%2FCPB.2007.9934) | 2008 | 6.3341 | **2009** | 2016 | ▂▂▂▂▂▂▂▂▂▂▂▂▂▂▂▂▂▂▂▃▃▃▃▃▃▃▃ |
| RIZZO AA, 2004, NEUROPSYCHOL REHABIL, V14, P207, [DOI](http://dx.doi.org/10.1080%2F09602010343000183) | 2004 | 7.8262 | **2009** | 2012 | ▂▂▂▂▂▂▂▂▂▂▂▂▂▂▂▂▂▂▂▃▃▃▃▂▂▂▂ |
| GORINI ALESSANDRA, 2008, EXPERT REV NEUROTHER, V8, P215, [DOI](http://dx.doi.org/10.1586%2F14737175.8.2.215) | 2008 | 6.1023 | **2009** | 2011 | ▂▂▂▂▂▂▂▂▂▂▂▂▂▂▂▂▂▂▂▃▃▃▂▂▂▂▂ |
| THORNTON M, 2005, BRAIN INJURY, V19, P989, [DOI](http://dx.doi.org/10.1080%2F02699050500109944) | 2005 | 7.9907 | **2009** | 2013 | ▂▂▂▂▂▂▂▂▂▂▂▂▂▂▂▂▂▂▂▃▃▃▃▃▂▂▂ |
| ROSSER JC, 2007, ARCH SURG-CHICAGO, V142, P181, [DOI](http://dx.doi.org/10.1001%2FARCHSURG.142.2.181) | 2007 | 11.048 | **2009** | 2014 | ▂▂▂▂▂▂▂▂▂▂▂▂▂▂▂▂▂▂▂▃▃▃▃▃▃▂▂ |
| RESSLER KJ, 2004, ARCH GEN PSYCHIAT, V61, P1136, [DOI](http://dx.doi.org/10.1001%2FARCHPSYC.61.11.1136) | 2004 | 9.6493 | **2009** | 2011 | ▂▂▂▂▂▂▂▂▂▂▂▂▂▂▂▂▂▂▂▃▃▃▂▂▂▂▂ |
| POWERS MB, 2008, J ANXIETY DISORD, V22, P561, [DOI](http://dx.doi.org/10.1016%2FJ.JANXDIS.2007.04.006) | 2008 | 27.5704 | **2010** | 2016 | ▂▂▂▂▂▂▂▂▂▂▂▂▂▂▂▂▂▂▂▂▃▃▃▃▃▃▃ |
| SATAVA RM, 2008, WORLD J SURG, V32, P141, [DOI](http://dx.doi.org/10.1007%2FS00268-007-9374-Y) | 2008 | 8.0257 | **2010** | 2013 | ▂▂▂▂▂▂▂▂▂▂▂▂▂▂▂▂▂▂▂▂▃▃▃▃▂▂▂ |
| MIRELMAN A, 2009, STROKE, V40, P169, [DOI](http://dx.doi.org/10.1161%2FSTROKEAHA.108.516328) | 2009 | 11.7784 | **2010** | 2016 | ▂▂▂▂▂▂▂▂▂▂▂▂▂▂▂▂▂▂▂▂▃▃▃▃▃▃▃ |
| PARK J, 2007, AM J SURG, V194, P205, [DOI](http://dx.doi.org/10.1016%2FJ.AMJSURG.2006.11.032) | 2007 | 9.2687 | **2010** | 2016 | ▂▂▂▂▂▂▂▂▂▂▂▂▂▂▂▂▂▂▂▂▃▃▃▃▃▃▃ |
| PARSONS TD, 2008, J BEHAV THER EXP PSY, V39, P250, [DOI](http://dx.doi.org/10.1016%2FJ.JBTEP.2007.07.007) | 2008 | 27.4003 | **2010** | 2016 | ▂▂▂▂▂▂▂▂▂▂▂▂▂▂▂▂▂▂▂▂▃▃▃▃▃▃▃ |
| LARSEN CR, 2009, BRIT MED J, V338, P, [DOI](http://dx.doi.org/10.1136%2FBMJ.B1802) | 2009 | 25.5602 | **2010** | 2016 | ▂▂▂▂▂▂▂▂▂▂▂▂▂▂▂▂▂▂▂▂▃▃▃▃▃▃▃ |
| YAVUZER G, 2008, EUR J PHYS REHAB MED, V44, P237 | 2008 | 14.428 | **2010** | 2016 | ▂▂▂▂▂▂▂▂▂▂▂▂▂▂▂▂▂▂▂▂▃▃▃▃▃▃▃ |
| WIGNALL GR, 2008, J UROLOGY, V179, P1690, [DOI](http://dx.doi.org/10.1016%2FJ.JURO.2008.01.014) | 2008 | 7.4149 | **2010** | 2011 | ▂▂▂▂▂▂▂▂▂▂▂▂▂▂▂▂▂▂▂▂▃▃▂▂▂▂▂ |
| KLINGER E, 2005, CYBERPSYCHOL BEHAV, V8, P76, [DOI](http://dx.doi.org/10.1089%2FCPB.2005.8.76) | 2005 | 10.2861 | **2010** | 2013 | ▂▂▂▂▂▂▂▂▂▂▂▂▂▂▂▂▂▂▂▂▃▃▃▃▂▂▂ |
| ZHANG AM, 2008, SURG ENDOSC, V22, P1440, [DOI](http://dx.doi.org/10.1007%2FS00464-007-9625-X) | 2008 | 9.6794 | **2010** | 2016 | ▂▂▂▂▂▂▂▂▂▂▂▂▂▂▂▂▂▂▂▂▃▃▃▃▃▃▃ |
| SCOTT DJ, 2008, J GASTROINTEST SURG, V12, P213, [DOI](http://dx.doi.org/10.1007%2FS11605-007-0357-Y) | 2008 | 8.8122 | **2010** | 2016 | ▂▂▂▂▂▂▂▂▂▂▂▂▂▂▂▂▂▂▂▂▃▃▃▃▃▃▃ |
| STURM LP, 2008, ANN SURG, V248, P166, [DOI](http://dx.doi.org/10.1097%2FSLA.0B013E318176BF24) | 2008 | 19.4719 | **2010** | 2016 | ▂▂▂▂▂▂▂▂▂▂▂▂▂▂▂▂▂▂▂▂▃▃▃▃▃▃▃ |
| CROSBIE JH, 2007, DISABIL REHABIL, V29, P1139, [DOI](http://dx.doi.org/10.1080%2F09638280600960909) | 2007 | 10.4601 | **2010** | 2016 | ▂▂▂▂▂▂▂▂▂▂▂▂▂▂▂▂▂▂▂▂▃▃▃▃▃▃▃ |
| LUCCA LF, 2009, J REHABIL MED, V41, P1003, [DOI](http://dx.doi.org/10.2340%2F16501977-0405) | 2009 | 9.1167 | **2010** | 2016 | ▂▂▂▂▂▂▂▂▂▂▂▂▂▂▂▂▂▂▂▂▃▃▃▃▃▃▃ |
| FREEMAN D, 2008, SCHIZOPHRENIA BULL, V34, P605, [DOI](http://dx.doi.org/10.1093%2FSCHBUL%2FSBN020) | 2008 | 8.8022 | **2010** | 2016 | ▂▂▂▂▂▂▂▂▂▂▂▂▂▂▂▂▂▂▂▂▃▃▃▃▃▃▃ |
| GURUSAMY K, 2008, BRIT J SURG, V95, P1088, [DOI](http://dx.doi.org/10.1002%2FBJS.6344) | 2008 | 17.8426 | **2010** | 2016 | ▂▂▂▂▂▂▂▂▂▂▂▂▂▂▂▂▂▂▂▂▃▃▃▃▃▃▃ |
| SANDLUND M, 2009, DEV MED CHILD NEUROL, V51, P173, [DOI](http://dx.doi.org/10.1111%2FJ.1469-8749.2008.03184.X) | 2009 | 8.8668 | **2010** | 2014 | ▂▂▂▂▂▂▂▂▂▂▂▂▂▂▂▂▂▂▂▂▃▃▃▃▃▂▂ |
| BOUCHARD S, 2008, PRESENCE-TELEOP VIRT, V17, P376, [DOI](http://dx.doi.org/10.1162%2FPRES.17.4.376) | 2008 | 6.6171 | **2010** | 2016 | ▂▂▂▂▂▂▂▂▂▂▂▂▂▂▂▂▂▂▂▂▃▃▃▃▃▃▃ |
| ADAMOVICH SV, 2009, NEUROREHABILITATION, V25, P29, [DOI](http://dx.doi.org/10.3233%2FNRE-2009-0497) | 2009 | 16.5983 | **2010** | 2016 | ▂▂▂▂▂▂▂▂▂▂▂▂▂▂▂▂▂▂▂▂▃▃▃▃▃▃▃ |
| SEYMOUR NE, 2008, WORLD J SURG, V32, P182, [DOI](http://dx.doi.org/10.1007%2FS00268-007-9307-9) | 2008 | 18.5261 | **2010** | 2016 | ▂▂▂▂▂▂▂▂▂▂▂▂▂▂▂▂▂▂▂▂▃▃▃▃▃▃▃ |
| MCDOUGALL EM, 2007, J ENDOUROL, V21, P244, [DOI](http://dx.doi.org/10.1089%2FEND.2007.9985) | 2007 | 11.3958 | **2010** | 2016 | ▂▂▂▂▂▂▂▂▂▂▂▂▂▂▂▂▂▂▂▂▃▃▃▃▃▃▃ |
| BISSON E, 2007, CYBERPSYCHOL BEHAV, V10, P16, [DOI](http://dx.doi.org/10.1089%2FCPB.2006.9997) | 2007 | 8.1299 | **2011** | 2016 | ▂▂▂▂▂▂▂▂▂▂▂▂▂▂▂▂▂▂▂▂▂▃▃▃▃▃▃ |
| LARSEN CR, 2006, SURG ENDOSC, V20, P1460, [DOI](http://dx.doi.org/10.1007%2FS00464-005-0745-X) | 2006 | 9.9044 | **2011** | 2014 | ▂▂▂▂▂▂▂▂▂▂▂▂▂▂▂▂▂▂▂▂▂▃▃▃▃▂▂ |
| AGGARWAL R, 2009, BRIT J SURG, V96, P1086, [DOI](http://dx.doi.org/10.1002%2FBJS.6679) | 2009 | 23.8899 | **2011** | 2016 | ▂▂▂▂▂▂▂▂▂▂▂▂▂▂▂▂▂▂▂▂▂▃▃▃▃▃▃ |
| SNIDER L, 2010, DEV NEUROREHABIL, V13, P120, [DOI](http://dx.doi.org/10.3109%2F17518420903357753) | 2010 | 11.1911 | **2011** | 2016 | ▂▂▂▂▂▂▂▂▂▂▂▂▂▂▂▂▂▂▂▂▂▃▃▃▃▃▃ |
| AHLBERG G, 2007, AM J SURG, V193, P797, [DOI](http://dx.doi.org/10.1016%2FJ.AMJSURG.2006.06.050) | 2007 | 26.3445 | **2011** | 2016 | ▂▂▂▂▂▂▂▂▂▂▂▂▂▂▂▂▂▂▂▂▂▃▃▃▃▃▃ |
| YANG YR, 2008, GAIT POSTURE, V28, P201, [DOI](http://dx.doi.org/10.1016%2FJ.GAITPOST.2007.11.007) | 2008 | 16.7309 | **2011** | 2016 | ▂▂▂▂▂▂▂▂▂▂▂▂▂▂▂▂▂▂▂▂▂▃▃▃▃▃▃ |
| KRIJN M, 2004, CLIN PSYCHOL REV, V24, P259, [DOI](http://dx.doi.org/10.1016%2FJ.CPR.2004.04.001) | 2004 | 8.7802 | **2011** | 2012 | ▂▂▂▂▂▂▂▂▂▂▂▂▂▂▂▂▂▂▂▂▂▃▃▂▂▂▂ |
| SLATER M, 2009, PHILOS T R SOC B, V364, P3549, [DOI](http://dx.doi.org/10.1098%2FRSTB.2009.0138) | 2009 | 15.377 | **2011** | 2016 | ▂▂▂▂▂▂▂▂▂▂▂▂▂▂▂▂▂▂▂▂▂▃▃▃▃▃▃ |
| DEUTSCH JE, 2008, PHYS THER, V88, P1196, [DOI](http://dx.doi.org/10.2522%2FPTJ.20080062) | 2008 | 23.0244 | **2011** | 2016 | ▂▂▂▂▂▂▂▂▂▂▂▂▂▂▂▂▂▂▂▂▂▃▃▃▃▃▃ |
| DALGARNO B, 2010, BRIT J EDUC TECHNOL, V41, P10, [DOI](http://dx.doi.org/10.1111%2FJ.1467-8535.2009.01038.X) | 2010 | 7.0599 | **2011** | 2016 | ▂▂▂▂▂▂▂▂▂▂▂▂▂▂▂▂▂▂▂▂▂▃▃▃▃▃▃ |
| LENGGENHAGER B, 2007, SCIENCE, V317, P1096, [DOI](http://dx.doi.org/10.1126%2FSCIENCE.1143439) | 2007 | 12.2128 | **2011** | 2016 | ▂▂▂▂▂▂▂▂▂▂▂▂▂▂▂▂▂▂▂▂▂▃▃▃▃▃▃ |
| RAND DEBBIE, 2008, J NEUROL PHYS THER, V32, P155, [DOI](http://dx.doi.org/10.1097%2FNPT.0B013E31818EE779) | 2008 | 10.9623 | **2011** | 2016 | ▂▂▂▂▂▂▂▂▂▂▂▂▂▂▂▂▂▂▂▂▂▃▃▃▃▃▃ |
| SROKA G, 2010, AM J SURG, V199, P115, [DOI](http://dx.doi.org/10.1016%2FJ.AMJSURG.2009.07.035) | 2010 | 18.1628 | **2011** | 2016 | ▂▂▂▂▂▂▂▂▂▂▂▂▂▂▂▂▂▂▂▂▂▃▃▃▃▃▃ |
| CALATAYUD D, 2010, ANN SURG, V251, P1181, [DOI](http://dx.doi.org/10.1097%2FSLA.0B013E3181DEB630) | 2010 | 14.819 | **2011** | 2016 | ▂▂▂▂▂▂▂▂▂▂▂▂▂▂▂▂▂▂▂▂▂▃▃▃▃▃▃ |
| SAPOSNIK G, 2010, INT J STROKE, V5, P47, [DOI](http://dx.doi.org/10.1111%2FJ.1747-4949.2009.00404.X) | 2010 | 10.0723 | **2011** | 2016 | ▂▂▂▂▂▂▂▂▂▂▂▂▂▂▂▂▂▂▂▂▂▃▃▃▃▃▃ |
| GOLOMB MR, 2010, ARCH PHYS MED REHAB, V91, P1, [DOI](http://dx.doi.org/10.1016%2FJ.APMR.2009.08.153) | 2010 | 9.7472 | **2011** | 2016 | ▂▂▂▂▂▂▂▂▂▂▂▂▂▂▂▂▂▂▂▂▂▃▃▃▃▃▃ |
| KENNEY PA, 2009, UROLOGY, V73, P1288, [DOI](http://dx.doi.org/10.1016%2FJ.UROLOGY.2008.12.044) | 2009 | 11.9678 | **2011** | 2016 | ▂▂▂▂▂▂▂▂▂▂▂▂▂▂▂▂▂▂▂▂▂▃▃▃▃▃▃ |
| PIRON L, 2009, J REHABIL MED, V41, P1016, [DOI](http://dx.doi.org/10.2340%2F16501977-0459) | 2009 | 10.7266 | **2011** | 2016 | ▂▂▂▂▂▂▂▂▂▂▂▂▂▂▂▂▂▂▂▂▂▃▃▃▃▃▃ |
| GURUSAMY KS, 2009, COCHRANE DB SYST REV, V, P, [DOI](http://dx.doi.org/10.1002%2F14651858.CD006575.PUB2) | 2009 | 10.0333 | **2011** | 2014 | ▂▂▂▂▂▂▂▂▂▂▂▂▂▂▂▂▂▂▂▂▂▃▃▃▃▂▂ |
| FREEMAN D, 2008, BRIT J PSYCHIAT, V192, P258, [DOI](http://dx.doi.org/10.1192%2FBJP.BP.107.044677) | 2008 | 10.8806 | **2011** | 2016 | ▂▂▂▂▂▂▂▂▂▂▂▂▂▂▂▂▂▂▂▂▂▃▃▃▃▃▃ |
| HOWELLS NR, 2008, J BONE JOINT SURG BR, V90B, P494, [DOI](http://dx.doi.org/10.1302%2F0301-620X.90B4.20414) | 2008 | 10.3484 | **2011** | 2016 | ▂▂▂▂▂▂▂▂▂▂▂▂▂▂▂▂▂▂▂▂▂▃▃▃▃▃▃ |
| CUSHMAN LA, 2008, NEUROLOGY, V71, P888, [DOI](http://dx.doi.org/10.1212%2F01.WNL.0000326262.67613.FE) | 2008 | 8.6086 | **2012** | 2016 | ▂▂▂▂▂▂▂▂▂▂▂▂▂▂▂▂▂▂▂▂▂▂▃▃▃▃▃ |
| SCHWEBEL DC, 2008, ACCIDENT ANAL PREV, V40, P1394, [DOI](http://dx.doi.org/10.1016%2FJ.AAP.2008.03.005) | 2008 | 6.6922 | **2012** | 2016 | ▂▂▂▂▂▂▂▂▂▂▂▂▂▂▂▂▂▂▂▂▂▂▃▃▃▃▃ |
| VAN SICKLE KR, 2008, J AM COLL SURGEONS, V207, P560, [DOI](http://dx.doi.org/10.1016%2FJ.JAMCOLLSURG.2008.05.007) | 2008 | 11.26 | **2012** | 2016 | ▂▂▂▂▂▂▂▂▂▂▂▂▂▂▂▂▂▂▂▂▂▂▃▃▃▃▃ |
| SLATER M, 2010, PLOS ONE, V5, P, [DOI](http://dx.doi.org/10.1371%2FJOURNAL.PONE.0010564) | 2010 | 20.9817 | **2012** | 2016 | ▂▂▂▂▂▂▂▂▂▂▂▂▂▂▂▂▂▂▂▂▂▂▃▃▃▃▃ |
| MEYERBROKER K, 2010, DEPRESS ANXIETY, V27, P933, [DOI](http://dx.doi.org/10.1002%2FDA.20734) | 2010 | 17.0565 | **2012** | 2016 | ▂▂▂▂▂▂▂▂▂▂▂▂▂▂▂▂▂▂▂▂▂▂▃▃▃▃▃ |
| SLATER MEL, 2009, FRONT NEUROSCI, V3, P214, [DOI](http://dx.doi.org/10.3389%2FNEURO.01.029.2009) | 2009 | 9.831 | **2012** | 2016 | ▂▂▂▂▂▂▂▂▂▂▂▂▂▂▂▂▂▂▂▂▂▂▃▃▃▃▃ |
| BANERJEE PP, 2007, J NEUROSURG, V107, P515, [DOI](http://dx.doi.org/10.3171%2FJNS-07%2F09%2F0515) | 2007 | 8.744 | **2012** | 2016 | ▂▂▂▂▂▂▂▂▂▂▂▂▂▂▂▂▂▂▂▂▂▂▃▃▃▃▃ |
| LAVER KE, 2011, COCHRANE DB SYST REV, V9,, P | 2011 | 11.7952 | **2012** | 2016 | ▂▂▂▂▂▂▂▂▂▂▂▂▂▂▂▂▂▂▂▂▂▂▃▃▃▃▃ |
| HAFTING T, 2005, NATURE, V436, P801, [DOI](http://dx.doi.org/10.1038%2FNATURE03721) | 2005 | 5.5635 | **2012** | 2013 | ▂▂▂▂▂▂▂▂▂▂▂▂▂▂▂▂▂▂▂▂▂▂▃▃▂▂▂ |
| FERRER-GARCIA M, 2012, BODY IMAGE, V9, P1, [DOI](http://dx.doi.org/10.1016%2FJ.BODYIM.2011.10.001) | 2012 | 6.9078 | **2012** | 2016 | ▂▂▂▂▂▂▂▂▂▂▂▂▂▂▂▂▂▂▂▂▂▂▃▃▃▃▃ |
| MABREY JD, 2010, CLIN ORTHOP RELAT R, V468, P2586, [DOI](http://dx.doi.org/10.1007%2FS11999-010-1426-1) | 2010 | 8.1288 | **2012** | 2016 | ▂▂▂▂▂▂▂▂▂▂▂▂▂▂▂▂▂▂▂▂▂▂▃▃▃▃▃ |
| LANGHORNE P, 2009, LANCET NEUROL, V8, P741, [DOI](http://dx.doi.org/10.1016%2FS1474-4422%2809%2970150-4) | 2009 | 12.5191 | **2012** | 2016 | ▂▂▂▂▂▂▂▂▂▂▂▂▂▂▂▂▂▂▂▂▂▂▃▃▃▃▃ |
| BURKE JW, 2009, VISUAL COMPUT, V25, P1085, [DOI](http://dx.doi.org/10.1007%2FS00371-009-0387-4) | 2009 | 11.8697 | **2012** | 2016 | ▂▂▂▂▂▂▂▂▂▂▂▂▂▂▂▂▂▂▂▂▂▂▃▃▃▃▃ |
| SAPOSNIK G, 2010, STROKE, V41, P1477, [DOI](http://dx.doi.org/10.1161%2FSTROKEAHA.110.584979) | 2010 | 40.8356 | **2012** | 2016 | ▂▂▂▂▂▂▂▂▂▂▂▂▂▂▂▂▂▂▂▂▂▂▃▃▃▃▃ |
| GORINI A, 2010, ANN GEN PSYCHIATR, V9, P, [DOI](http://dx.doi.org/10.1186%2F1744-859X-9-30) | 2010 | 6.2137 | **2012** | 2016 | ▂▂▂▂▂▂▂▂▂▂▂▂▂▂▂▂▂▂▂▂▂▂▃▃▃▃▃ |
| MIRELMAN A, 2011, J GERONTOL A-BIOL, V66, P234, [DOI](http://dx.doi.org/10.1093%2FGERONA%2FGLQ201) | 2011 | 13.6682 | **2012** | 2016 | ▂▂▂▂▂▂▂▂▂▂▂▂▂▂▂▂▂▂▂▂▂▂▃▃▃▃▃ |
| MCLAY RN, 2011, CYBERPSYCH BEH SOC N, V14, P223, [DOI](http://dx.doi.org/10.1089%2FCYBER.2011.0003) | 2011 | 8.9431 | **2012** | 2016 | ▂▂▂▂▂▂▂▂▂▂▂▂▂▂▂▂▂▂▂▂▂▂▃▃▃▃▃ |
| BOWMAN DA, 2007, COMPUTER, V40, P36, [DOI](http://dx.doi.org/10.1109%2FMC.2007.257) | 2007 | 13.9958 | **2012** | 2016 | ▂▂▂▂▂▂▂▂▂▂▂▂▂▂▂▂▂▂▂▂▂▂▃▃▃▃▃ |
| KIM JH, 2009, AM J PHYS MED REHAB, V88, P693, [DOI](http://dx.doi.org/10.1097%2FPHM.0B013E3181B33350) | 2009 | 14.5605 | **2012** | 2016 | ▂▂▂▂▂▂▂▂▂▂▂▂▂▂▂▂▂▂▂▂▂▂▃▃▃▃▃ |
| MALONE HR, 2010, NEUROSURGERY, V67, P1105, [DOI](http://dx.doi.org/10.1227%2FNEU.0B013E3181EE46D0) | 2010 | 12.157 | **2012** | 2016 | ▂▂▂▂▂▂▂▂▂▂▂▂▂▂▂▂▂▂▂▂▂▂▃▃▃▃▃ |
| CAMEIRAO MS, 2012, STROKE, V43, P2720, [DOI](http://dx.doi.org/10.1161%2FSTROKEAHA.112.653196) | 2012 | 8.1288 | **2012** | 2016 | ▂▂▂▂▂▂▂▂▂▂▂▂▂▂▂▂▂▂▂▂▂▂▃▃▃▃▃ |
| ADAMS R, 2009, CHILD NEUROPSYCHOL, V15, P120, [DOI](http://dx.doi.org/10.1080%2F09297040802169077) | 2009 | 4.9935 | **2012** | 2016 | ▂▂▂▂▂▂▂▂▂▂▂▂▂▂▂▂▂▂▂▂▂▂▃▃▃▃▃ |
| HARVEY CD, 2009, NATURE, V461, P941, [DOI](http://dx.doi.org/10.1038%2FNATURE08499) | 2009 | 10.2387 | **2012** | 2016 | ▂▂▂▂▂▂▂▂▂▂▂▂▂▂▂▂▂▂▂▂▂▂▃▃▃▃▃ |
| GRAVES LEF, 2010, J PHYS ACT HEALTH, V7, P393 | 2010 | 7.4345 | **2012** | 2016 | ▂▂▂▂▂▂▂▂▂▂▂▂▂▂▂▂▂▂▂▂▂▂▃▃▃▃▃ |
| LEMOLE GM, 2007, NEUROSURGERY, V61, P142, [DOI](http://dx.doi.org/10.1227%2F01.NEU.0000255485.65450.49) | 2007 | 12.3896 | **2012** | 2016 | ▂▂▂▂▂▂▂▂▂▂▂▂▂▂▂▂▂▂▂▂▂▂▃▃▃▃▃ |
| JOO LY, 2010, J REHABIL MED, V42, P437, [DOI](http://dx.doi.org/10.2340%2F16501977-0528) | 2010 | 12.9273 | **2012** | 2016 | ▂▂▂▂▂▂▂▂▂▂▂▂▂▂▂▂▂▂▂▂▂▂▃▃▃▃▃ |
| PARK KM, 2011, PSYCHIAT RES, V189, P166, [DOI](http://dx.doi.org/10.1016%2FJ.PSYCHRES.2011.04.003) | 2011 | 8.1288 | **2012** | 2016 | ▂▂▂▂▂▂▂▂▂▂▂▂▂▂▂▂▂▂▂▂▂▂▃▃▃▃▃ |
| SAPOSNIK G, 2011, STROKE, V42, P1380, [DOI](http://dx.doi.org/10.1161%2FSTROKEAHA.110.605451) | 2011 | 33.3318 | **2012** | 2016 | ▂▂▂▂▂▂▂▂▂▂▂▂▂▂▂▂▂▂▂▂▂▂▃▃▃▃▃ |
| BOTELLA C, 2012, CYBERPSYCH BEH SOC N, V15, P78, [DOI](http://dx.doi.org/10.1089%2FCYBER.2011.0140) | 2012 | 5.2808 | **2012** | 2016 | ▂▂▂▂▂▂▂▂▂▂▂▂▂▂▂▂▂▂▂▂▂▂▃▃▃▃▃ |
| SHARPLES S, 2008, DISPLAYS, V29, P58, [DOI](http://dx.doi.org/10.1016%2FJ.DISPLA.2007.09.005) | 2008 | 8.5612 | **2012** | 2016 | ▂▂▂▂▂▂▂▂▂▂▂▂▂▂▂▂▂▂▂▂▂▂▃▃▃▃▃ |
| GIL-GOMEZ JA, 2011, J NEUROENG REHABIL, V8, P, [DOI](http://dx.doi.org/10.1186%2F1743-0003-8-30) | 2011 | 16.1039 | **2012** | 2016 | ▂▂▂▂▂▂▂▂▂▂▂▂▂▂▂▂▂▂▂▂▂▂▃▃▃▃▃ |
| AGMON M, 2011, J GERIATR PHYS THER, V34, P161, [DOI](http://dx.doi.org/10.1519%2FJPT.0B013E3182191D98) | 2011 | 12.3577 | **2013** | 2016 | ▂▂▂▂▂▂▂▂▂▂▂▂▂▂▂▂▂▂▂▂▂▂▂▃▃▃▃ |
| FLYNN SHERYL, 2007, J NEUROL PHYS THER, V31, P180, [DOI](http://dx.doi.org/10.1097%2FNPT.0B013E31815D00D5) | 2007 | 15.0579 | **2013** | 2016 | ▂▂▂▂▂▂▂▂▂▂▂▂▂▂▂▂▂▂▂▂▂▂▂▃▃▃▃ |
| MOUAWAD MR, 2011, J REHABIL MED, V43, P527, [DOI](http://dx.doi.org/10.2340%2F16501977-0816) | 2011 | 13.1947 | **2013** | 2016 | ▂▂▂▂▂▂▂▂▂▂▂▂▂▂▂▂▂▂▂▂▂▂▂▃▃▃▃ |
| KILTENI K, 2012, PLOS ONE, V7, P, [DOI](http://dx.doi.org/10.1371%2FJOURNAL.PONE.0040867) | 2012 | 12.8341 | **2013** | 2016 | ▂▂▂▂▂▂▂▂▂▂▂▂▂▂▂▂▂▂▂▂▂▂▂▃▃▃▃ |
| MCGAGHIE WC, 2010, MED EDUC, V44, P50, [DOI](http://dx.doi.org/10.1111%2FJ.1365-2923.2009.03547.X) | 2010 | 9.3995 | **2013** | 2016 | ▂▂▂▂▂▂▂▂▂▂▂▂▂▂▂▂▂▂▂▂▂▂▂▃▃▃▃ |
| CROCHET P, 2011, ANN SURG, V253, P1216, [DOI](http://dx.doi.org/10.1097%2FSLA.0B013E3182197016) | 2011 | 10.4923 | **2013** | 2016 | ▂▂▂▂▂▂▂▂▂▂▂▂▂▂▂▂▂▂▂▂▂▂▂▃▃▃▃ |
| COOK DA, 2011, JAMA-J AM MED ASSOC, V306, P978, [DOI](http://dx.doi.org/10.1001%2FJAMA.2011.1234) | 2011 | 16.6475 | **2013** | 2016 | ▂▂▂▂▂▂▂▂▂▂▂▂▂▂▂▂▂▂▂▂▂▂▂▃▃▃▃ |
| LANGE B, 2012, DISABIL REHABIL, V34, P1863, [DOI](http://dx.doi.org/10.3109%2F09638288.2012.670029) | 2012 | 10.4532 | **2013** | 2016 | ▂▂▂▂▂▂▂▂▂▂▂▂▂▂▂▂▂▂▂▂▂▂▂▃▃▃▃ |
| ALARAJ ALI, 2011, SURG NEUROL INT, V2, P52, [DOI](http://dx.doi.org/10.4103%2F2152-7806.80117) | 2011 | 9.2829 | **2013** | 2016 | ▂▂▂▂▂▂▂▂▂▂▂▂▂▂▂▂▂▂▂▂▂▂▂▃▃▃▃ |
| ESCULIER JF, 2012, J REHABIL MED, V44, P144, [DOI](http://dx.doi.org/10.2340%2F16501977-0922) | 2012 | 10.7108 | **2013** | 2016 | ▂▂▂▂▂▂▂▂▂▂▂▂▂▂▂▂▂▂▂▂▂▂▂▃▃▃▃ |
| DELORME S, 2012, NEUROSURGERY, V71, P32, [DOI](http://dx.doi.org/10.1227%2FNEU.0B013E318249C744) | 2012 | 13.5687 | **2013** | 2016 | ▂▂▂▂▂▂▂▂▂▂▂▂▂▂▂▂▂▂▂▂▂▂▂▃▃▃▃ |
| DIESEN DL, 2011, J SURG EDUC, V68, P282, [DOI](http://dx.doi.org/10.1016%2FJ.JSURG.2011.02.007) | 2011 | 10.4923 | **2013** | 2016 | ▂▂▂▂▂▂▂▂▂▂▂▂▂▂▂▂▂▂▂▂▂▂▂▃▃▃▃ |
| CLARK RA, 2010, GAIT POSTURE, V31, P307, [DOI](http://dx.doi.org/10.1016%2FJ.GAITPOST.2009.11.012) | 2010 | 12.9533 | **2013** | 2016 | ▂▂▂▂▂▂▂▂▂▂▂▂▂▂▂▂▂▂▂▂▂▂▂▃▃▃▃ |
| RIVA GIUSEPPE, 2011, STUD HEALTH TECHNOL INFORM, V163, P493 | 2011 | 11.921 | **2013** | 2016 | ▂▂▂▂▂▂▂▂▂▂▂▂▂▂▂▂▂▂▂▂▂▂▂▃▃▃▃ |
| OPRIS D, 2012, DEPRESS ANXIETY, V29, P85, [DOI](http://dx.doi.org/10.1002%2FDA.20910) | 2012 | 21.6811 | **2013** | 2016 | ▂▂▂▂▂▂▂▂▂▂▂▂▂▂▂▂▂▂▂▂▂▂▂▃▃▃▃ |
| LARSEN CR, 2012, ACTA OBSTET GYN SCAN, V91, P1015, [DOI](http://dx.doi.org/10.1111%2FJ.1600-0412.2012.01482.X) | 2012 | 13.3105 | **2013** | 2016 | ▂▂▂▂▂▂▂▂▂▂▂▂▂▂▂▂▂▂▂▂▂▂▂▃▃▃▃ |
| LAVER K, 2012, EUR J PHYS REHAB MED, V48, P523 | 2012 | 15.6937 | **2013** | 2016 | ▂▂▂▂▂▂▂▂▂▂▂▂▂▂▂▂▂▂▂▂▂▂▂▃▃▃▃ |
| BOHIL CJ, 2011, NAT REV NEUROSCI, V12, P752, [DOI](http://dx.doi.org/10.1038%2FNRN3122) | 2011 | 30.6009 | **2013** | 2016 | ▂▂▂▂▂▂▂▂▂▂▂▂▂▂▂▂▂▂▂▂▂▂▂▃▃▃▃ |
| LAVER KE, 2011, COCHRANE DB SYST REV, V, P, [DOI](http://dx.doi.org/10.1002%2F14651858.CD008349.PUB2) | 2011 | 17.6016 | **2013** | 2016 | ▂▂▂▂▂▂▂▂▂▂▂▂▂▂▂▂▂▂▂▂▂▂▂▃▃▃▃ |
| MCGAGHIE WC, 2011, ACAD MED, V86, P706, [DOI](http://dx.doi.org/10.1097%2FACM.0B013E318217E119) | 2011 | 8.8458 | **2013** | 2016 | ▂▂▂▂▂▂▂▂▂▂▂▂▂▂▂▂▂▂▂▂▂▂▂▃▃▃▃ |
| DEUTSCH JE, 2011, TOP STROKE REHABIL, V18, P701, [DOI](http://dx.doi.org/10.1310%2FTSR1806-701) | 2011 | 8.4762 | **2013** | 2016 | ▂▂▂▂▂▂▂▂▂▂▂▂▂▂▂▂▂▂▂▂▂▂▂▃▃▃▃ |
| PLANCHER G, 2010, NEUROPSYCHOLOGY, V24, P379, [DOI](http://dx.doi.org/10.1037%2FA0018680) | 2010 | 4.7846 | **2013** | 2016 | ▂▂▂▂▂▂▂▂▂▂▂▂▂▂▂▂▂▂▂▂▂▂▂▃▃▃▃ |
| DUQUE G, 2013, CLIN INTERV AGING, V8, P257, [DOI](http://dx.doi.org/10.2147%2FCIA.S41453) | 2013 | 8.7145 | **2014** | 2016 | ▂▂▂▂▂▂▂▂▂▂▂▂▂▂▂▂▂▂▂▂▂▂▂▂▃▃▃ |
| MIRELMAN A, 2010, GAIT POSTURE, V31, P433, [DOI](http://dx.doi.org/10.1016%2FJ.GAITPOST.2010.01.016) | 2010 | 9.6077 | **2014** | 2016 | ▂▂▂▂▂▂▂▂▂▂▂▂▂▂▂▂▂▂▂▂▂▂▂▂▃▃▃ |
| SHIBAN Y, 2013, BEHAV RES THER, V51, P68, [DOI](http://dx.doi.org/10.1016%2FJ.BRAT.2012.10.007) | 2013 | 8.0205 | **2014** | 2016 | ▂▂▂▂▂▂▂▂▂▂▂▂▂▂▂▂▂▂▂▂▂▂▂▂▃▃▃ |
| TUROLLA A, 2013, J NEUROENG REHABIL, V10, P, [DOI](http://dx.doi.org/10.1186%2F1743-0003-10-85) | 2013 | 10.8967 | **2014** | 2016 | ▂▂▂▂▂▂▂▂▂▂▂▂▂▂▂▂▂▂▂▂▂▂▂▂▃▃▃ |
| AMERICAN PSYCHIATRIC ASSOCIATION, 2013, DIAGN STAT MAN MENT, V, P | 2013 | 17.9986 | **2014** | 2016 | ▂▂▂▂▂▂▂▂▂▂▂▂▂▂▂▂▂▂▂▂▂▂▂▂▃▃▃ |
| RENDON AA, 2012, AGE AGEING, V41, P549, [DOI](http://dx.doi.org/10.1093%2FAGEING%2FAFS053) | 2012 | 15.117 | **2014** | 2016 | ▂▂▂▂▂▂▂▂▂▂▂▂▂▂▂▂▂▂▂▂▂▂▂▂▃▃▃ |
| ZENDEJAS B, 2013, ANN SURG, V257, P586, [DOI](http://dx.doi.org/10.1097%2FSLA.0B013E318288C40B) | 2013 | 12.9318 | **2014** | 2016 | ▂▂▂▂▂▂▂▂▂▂▂▂▂▂▂▂▂▂▂▂▂▂▂▂▃▃▃ |
| ANDERSON PL, 2013, J CONSULT CLIN PSYCH, V81, P751, [DOI](http://dx.doi.org/10.1037%2FA0033559) | 2013 | 9.8054 | **2014** | 2016 | ▂▂▂▂▂▂▂▂▂▂▂▂▂▂▂▂▂▂▂▂▂▂▂▂▃▃▃ |
| GALLAGHER AG, 2013, ANN SURG, V257, P1025, [DOI](http://dx.doi.org/10.1097%2FSLA.0B013E318284F658) | 2013 | 10.2024 | **2014** | 2016 | ▂▂▂▂▂▂▂▂▂▂▂▂▂▂▂▂▂▂▂▂▂▂▂▂▃▃▃ |
| CRASKE MG, 2008, BEHAV RES THER, V46, P5, [DOI](http://dx.doi.org/10.1016%2FJ.BRAT.2007.10.003) | 2008 | 6.0872 | **2014** | 2016 | ▂▂▂▂▂▂▂▂▂▂▂▂▂▂▂▂▂▂▂▂▂▂▂▂▃▃▃ |
| HOFFMAN HG, 2011, ANN BEHAV MED, V41, P183, [DOI](http://dx.doi.org/10.1007%2FS12160-010-9248-7) | 2011 | 10.1535 | **2014** | 2016 | ▂▂▂▂▂▂▂▂▂▂▂▂▂▂▂▂▂▂▂▂▂▂▂▂▃▃▃ |
| BANAKOU D, 2013, P NATL ACAD SCI USA, V110, P12846, [DOI](http://dx.doi.org/10.1073%2FPNAS.1306779110) | 2013 | 8.5658 | **2014** | 2016 | ▂▂▂▂▂▂▂▂▂▂▂▂▂▂▂▂▂▂▂▂▂▂▂▂▃▃▃ |
| CRASKE MG, 2014, BEHAV RES THER, V58, P10, [DOI](http://dx.doi.org/10.1016%2FJ.BRAT.2014.04.006) | 2014 | 4.3544 | **2014** | 2016 | ▂▂▂▂▂▂▂▂▂▂▂▂▂▂▂▂▂▂▂▂▂▂▂▂▃▃▃ |
| CHANG YJ, 2011, RES DEV DISABIL, V32, P2566, [DOI](http://dx.doi.org/10.1016%2FJ.RIDD.2011.07.002) | 2011 | 14.2738 | **2014** | 2016 | ▂▂▂▂▂▂▂▂▂▂▂▂▂▂▂▂▂▂▂▂▂▂▂▂▃▃▃ |
| KIM EK, 2012, J PHYS THER SCI, V24, P901 | 2012 | 5.6803 | **2014** | 2016 | ▂▂▂▂▂▂▂▂▂▂▂▂▂▂▂▂▂▂▂▂▂▂▂▂▃▃▃ |
| CLARK RA, 2012, GAIT POSTURE, V36, P372, [DOI](http://dx.doi.org/10.1016%2FJ.GAITPOST.2012.03.033) | 2012 | 8.7145 | **2014** | 2016 | ▂▂▂▂▂▂▂▂▂▂▂▂▂▂▂▂▂▂▂▂▂▂▂▂▃▃▃ |
| PLANCHER G, 2012, NEUROPSYCHOLOGIA, V50, P592, [DOI](http://dx.doi.org/10.1016%2FJ.NEUROPSYCHOLOGIA.2011.12.013) | 2012 | 11.6912 | **2014** | 2016 | ▂▂▂▂▂▂▂▂▂▂▂▂▂▂▂▂▂▂▂▂▂▂▂▂▃▃▃ |
