# Supplementary material for: The Past, Present, and Future of Virtual and Augmented Reality Research: A Network and Cluster Analysis of the Literature
Source: Front Psychol. 2018 Nov 6;9:2086. doi: 10.3389/fpsyg.2018.02086 (PMC6232426; doi:10.3389/fpsyg.2018.02086)
Supplement: Supplementary file 1 [file Data_Sheet_1.ZIP › Cluster_Category_WoS.docx]

| **ClusterID** | **Size** | **Silhouette** | **mean(Year)** | **Label (TFIDF)** | **Label (LLR)** | **Label (MI)** |
| --- | --- | --- | --- | --- | --- | --- |
| 0 | 27 | 0.523 | 1996 | (9.73) robotic system; (8.62) catheter; (8.62) freedom; (8.01) dof; (8.01) poster | virtual reality application (26.67, 1.0E-4); research (26.11, 1.0E-4); design (22.17, 1.0E-4); | advanced open source |
| 1 | 26 | 0.656 | 1997 | (10.17) feasibility; (10.01) neurosurgery; (9.22) motor function; (8.51) systematic review; (7.88) clinical article | patient (53.32, 1.0E-4); neurosurgery (45.25, 1.0E-4); parkinsons disease (33.49, 1.0E-4); | accuracy |
| 2 | 21 | 0.695 | 1992 | (6.93) barrier; (5.98) ancient ayutthaya; (5.98) thailand; (5.98) rubble; (5.59) frozen shoulder | virtual information desk (15.76, 1.0E-4); barrier (15.76, 1.0E-4); virtual environment (10.62, 0.005); | fiction |
| 3 | 19 | 0.57 | 1995 | (7.88) social anxiety; (6.93) polish isaf soldier; (6.93) meta-analysis; (6.93) social anxiety disorder; (6.53) systematic review | result (13.2, 0.001); virtual reality exposure therapy (13.2, 0.001); social anxiety disorder (13.2, 0.001); | anxiety disorder |
| 4 | 16 | 0.736 | 1993 | (8.01) poster; (7.88) nanomanipulator; (7.88) vrdd; (7.88) applying virtual reality visualization; (7.88) molecular structure | virtual environment (18.93, 1.0E-4); virtual reality technique (14.73, 0.001); perception (14.73, 0.001); | anxiety disorder |
| 5 | 7 | 0.967 | 1991 | (7.88) multisensory control; (5.59) using action observation; (5.59) differential neural activation pattern; (5.59) small group meeting; (5.59) grid cell | size (20.03, 1.0E-4); multisensory control (20.03, 1.0E-4); surgeon (10, 0.005); | auditory information |
